# Inhibitors of Fumarylacetoacetate Hydrolase Domain Containing Protein 1 (FAHD1)

**DOI:** 10.3390/molecules26165009

**Published:** 2021-08-18

**Authors:** Alexander K. H. Weiss, Richard Wurzer, Patrycia Klapec, Manuel Philip Eder, Johannes R. Loeffler, Susanne von Grafenstein, Stefania Monteleone, Klaus R. Liedl, Pidder Jansen-Dürr, Hubert Gstach

**Affiliations:** 1 Research Institute for Biomedical Aging Research, University of Innsbruck, Rennweg 10, A-6020 Innsbruck, Austria; 2 Department of Organic Chemistry, Faculty of Chemistry, University of Vienna, Währinger Straße 38, A-1090 Vienna, Austria; GHB@gmx.net; 3 Campus Tulln, University of Applied Sciences Wiener Neustadt, Konrad-Lorenz-Straße 10, A-3430 Tulln an der Donau, Austria; patrycia.klapec@gmx.at (P.K.); manuel.eder01@gmail.com (M.P.E.); 4 Institute of General, Inorganic and Theoretical Chemistry, University of Innsbruck, Innrain 58, A-6020 Innsbruck, Austria; johannes.r.loeffler@gmail.com (J.R.L.); susanne.grafenstein@gmail.com (S.v.G.); stefania.monteleone01@universitadipavia.it (S.M.); Klaus.Liedl@uibk.ac.at (K.R.L.); 5 Center for Molecular Biosciences Innsbruck (CMBI), University of Innsbruck, Innrain 58, A-6020 Innsbruck, Austria; 6 Department of Pharmaceutical Sciences, Pharmaceutical Chemistry Division, Faculty of Life Sciences, University of Vienna, Althanstraße 14, UZ2 E349, A-1090 Vienna, Austria

**Keywords:** FAHD1, enzyme inhibitor, mitochondria, ODx, oxaloacetate

## Abstract

*FAH domain containing protein 1* (FAHD1) acts as oxaloacetate decarboxylase in mitochondria, contributing to the regulation of the tricarboxylic acid cycle. Guided by a high-resolution X-ray structure of FAHD1 liganded by oxalate, the enzymatic mechanism of substrate processing is analyzed in detail. Taking the chemical features of the FAHD1 substrate oxaloacetate into account, the potential inhibitor structures are deduced. The synthesis of drug-like scaffolds afforded first-generation FAHD1-inhibitors with activities in the low micromolar IC_50_ range. The investigations disclosed structures competing with the substrate for binding to the metal cofactor, as well as scaffolds, which may have a novel binding mode to FAHD1.

## 1. Introduction

*FAH domain containing protein 1* (FAHD1) acts as bifunctional *oxaloacetate* (OAA) *decarboxylase* (ODx) [1] and *acylpyruvate hydrolase* (ApH) [2] in mitochondria. FAHD1 belongs to the broad FAH superfamily of enzymes [3,4]. While its ApH activity is only of minor biological relevance, FAHD1 is involved as ODx in the regulation of the tricarboxylic acid (TCA) cycle flux [5,6]. OAA is not only required for the central citrate synthase reaction in the TCA cycle but also acts as a competitive inhibitor of succinate dehydrogenase and as a cataplerotic metabolite. Our working model refers to a *mitochondrial dysfunction associated senescence* (MiDAS)-like phenotype [6], where the mitochondrial OAA levels are tightly regulated by FAHD1 activity. In particular, we investigate the role of FAHD1 in human cell metabolism, focusing on metabolic shifts in human cell lines upon FAHD1 knockdown, and we assess the dependence of these cells on FAHD1 for proliferation and survival. Successful experiments will put FAHD1 inhibition into the therapeutic context, to be tested in future efficacy studies in human tissue culture and xenograft models. We anticipate that the elucidation of the role of FAHD1 as a therapeutic target in the treatment of pathologies associated with mitochondrial dysfunction will stimulate translational research with the aim to contribute to new therapeutic strategies for human malignancies.

In the following, we discuss the substrate processing of FAHD1 based on structural information gained from the available X-ray structures of FAHD1. By a theoretical analysis, also taking into consideration the chemical features of the FAHD1 substrates (oxaloacetate and acylpyruvates), valuable proposals for the synthesis of inhibitors could be deduced. Their synthesis afforded the first inhibitors of FAHD1.

The information obtained by comparing the unliganded with the liganded protein models enabled the structural definition of the FAHD1 catalytic center in high resolution. X-ray data suggests a bidentate-binding mode of the 1,2-dicarbonyl motif of the inhibitor to the Mg cofactor and provides a rationale for closure of the lid domain and induction of a catalytically competent dyad (His30-Glu33) [7,8]. However, the structure model of FAHD1 liganded by oxalate does not tell us which residues are key in the enzymatic process, and the essential residues involved in driving the enzymatic reaction remain to be elucidated. Furthermore, no information about lid opening, product release and restauration of the native FAHD1 can be deduced from this data. To gain further mechanistic insights, it is therefore worth to address the central chemical step of both FAHD1 functions (ODx- and ApH) by a theoretical approach.

### 1.1. The C–C Bond Cleavage Mechanism in ODx- and ApH-Activity of FAHD1

**Figure 1:**
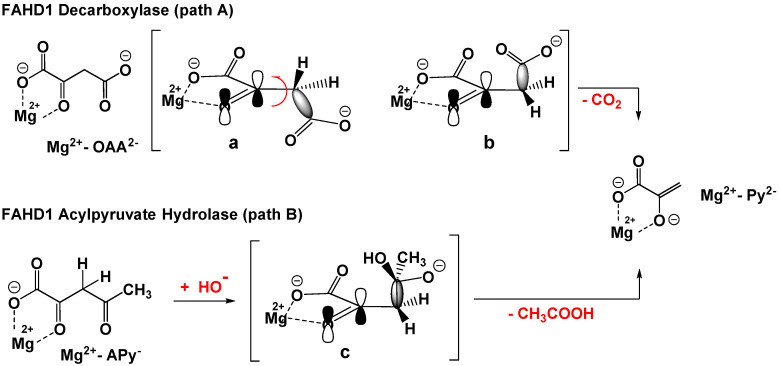
Orbital model for the ODx and ApH reactions catalyzed by FAHD1. Path A: ODx. Mg^2+^-OAA^2−^ depicted in two conformations: not competent (**a**) and competent conformations (**b**) for decarboxylation and the formation of the pyruvate enolate complex Mg^2+^-Py^2−^ (bent arrow indicates bond rotation); C–C bond cleavage under CO_2_ evolution to the Mg^2+^-complexed enolate of pyruvate (Mg^2+^-APY^2−^). Path B: ApH. Nucleophilic addition of hydroxyl to the 2-keto form of OAA^2−^; (**c**) intermediate oxyanion already in a productive conformation is shown; C–C bond cleavage under the extrusion of acetic acid and formation of the pyruvate enolate complex Mg^2+^-Py^2−^.

**Figure 2:**
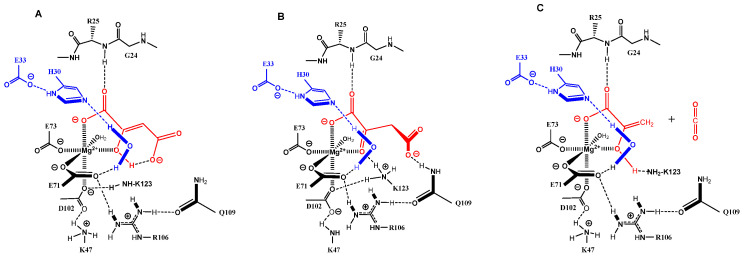
Mg-bound enol form of OAA (red, (**A**)); Q109 promoted the conformational control of ketonized OAA (red, (**B**)); quench of the primary enolate of pyruvate by K123 to pyruvate–enol and CO_2_ release (red, (**C**)); blue: catalytic dyad E33-H30 and captured water molecule; dotted black lines: hydrogen bonding; filled bonds are in front and point towards the viewer).

Both the hydrolase and the decarboxylase mechanisms share a common strategic chemical step, namely the scission of an equivalent carbon–carbon single bond in OAA and Ap, respectively. From the experimental data, it can be deduced that the substrates start initial binding to the cofactor in their enol form: Enolized oxaloacetate, as well as acylpyruvates, are planar molecules due to intramolecular resonance-stabilized hydrogen bonding (a comprehensive literature overview of features of oxaloacetate is provided in the Supporting Materials). The C–C σ-bond has no orbital overlap with the C–C double bond of the enol (Figure 1a). The Mg-bound enol of oxaloacetate is therefore not capable for decarboxylation. FAHD1 has to provide residues to isomerize the enol first into the keto form and, subsequently, to adjust a conformation of the molecule that supports a parallel alignment of the σ-bond to the π-bond of the carbonyl (Figure 1b and Figure 2B). This adds an isomerase activity as a third function to FAHD1.

A major conclusion of the theoretical analysis of the C–C bond cleavage in the FAHD1 mechanisms is, although the substrates, as well as the mechanisms behind are different, that both pathways lead to the same primary cleavage product, namely pyruvate enolate complexed to Mg^2+^. It must be taken into account that a pyruvate–enolate complex reveals a more than ten thousand-fold greater stability constant compared to simple carboxylic acids [9], implying a block of lid opening and product release. For the ODx, as well as ApH mechanisms, a quench of the primary enolate to the final enol of pyruvate is proposed to prevent inhibition by the product.

In the ApH mechanism, the generation of the nucleophilic hydroxyl from a conserved (and isolated) water molecule can be performed by the dyad of His-Glu present in reasonable proximity to the catalytically important water in the substrate pocket of FAHD1. The attack of the nucleophile on the *pro*-chiral carbonyl center of acylpyruvates (C^4^) follows strictly along the trajectories defined by the orientation of the acyl carbonyl group in the FAHD1 catalytic center [10]. The site of a nucleophilic attack will be under stereospecific control by the enzyme residues interacting with the bound substrate. The striking similarity of both mechanisms is that the oxyanion formed along the acylpyruvase pathway appears at the identical position as the negatively charged carboxylate oxygen in the ODx path. The difference between ODx and ApH mechanisms is the leaving molecule after C–C bond cleavage: carbon dioxide from ODx but acetic acid from the ApH mechanism. The quench of the enolate and lid opening in the ApH mechanism would be supported by the acetic acid.

The ketonization of the OAA enol form (Figure 2A) needs a proton transfer mediated by residues of the catalytic cavity. An enol–hydroxy function shows a more than ten thousand-fold increase in acidity when complexed to magnesium [11]. Lysine K123, which can adopt an equlibrium protonation state in the K43-D102-K123 network, would be able to deprotonate the enol and transfer the proton through the 4-carboxylate to the C^3^-centered carbanion. However, ketonization alone is not sufficient for decarboxylation. The 2-keto OAA has to adopt a conformation that is not in an energy minimum. A further residue in the catalytic cavity of FAHD1 must exist for control of the conformation of the bound OAA keto form. Through ketonization, the molecule gains rotational freedom around the C^2^–C^3^ bond and can achieve the parallel orbital orientation of the C^3^–C^4^ bond to the carbonyl π*-orbital by assistance of a residue provided by FAHD1. We assign the carbamoyl group of residue Q109, which is part of the hydrogen bond network E71-R106, the strategic role for the conformational control of bound substrates (Figure 2B). The energy needed for the C^3^–C^4^ bond rotation of OAA has been calculated to be 2.4 kcal/mol [12]. The importance of the glutamine residue in the catalytic mechanism has been shown for Cg1458, the prokaryotic analog of FAHD1. Mutation of the corresponding residue almost abolished Cg1458-ODx activity (98%) [13].

Persistent formation of the extremely stable Mg^2+^–enolate complex [9] can be prohibited in an equilibrium protonation of enolate oxygen through the ammonium group of K123. The products of OAA processing by FAHD1 would be pyruvate enol and carbon dioxide (Figure 2C). However, pyruvate enol in the bidentate-binding mode to Mg^2+^ still supports the strong hydrogen bonding of the 1-carboxylate to NH of liganded G24. Consequently, the lid of FAHD1 would not open for product release (equivalent to inhibition by the product). At this point of the discussion, it must be added that pyruvate enol is a high-energy molecule storing ~ 7 kcal/moL of free energy relative to the keto form [14]. Taking the energy reservoir present in the pyruvate enol form into account, we propose once more a ketonization of the enol as an additional step prior to the lid opening. The necessary proton transfer could be supported by the conserved water molecule positioned by the E33-H30 dyad or through the D102-K123 network. In the case of the water molecule, the produced hydroxyl would be competent to displace the pyruvate from the magnesium, destroying the stabilization of liganded G24, which would revert to the conformation adopted in the unliganded structure. The lid could open under release of pyruvate as final product. The catalytic center would be restored for the next catalytic cycle upon exposure to the mitochondrial environment (remark: the conserved water molecule would show amphoteric behavior: acidic in the ODx mechanism but nucleophilic in the hydrolase mechanism).

### 1.2. Design Steps of First Generation of FAHD1 Inhibitors

**Figure 3:**
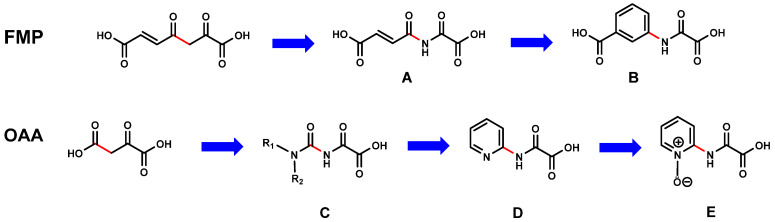
The design principle of the 1st-generation FAHD1 inhibitors. Upper lane: deduction of oxalamic acid (**B**) starting from substrate fumarylpyruvate (FMP); bottom lane: deduction of *N*-pyridyl-oxalamic acid (**D**) and corresponding 1-oxide congener (**E**) starting from oxaloacetate (OAA); (red: initial C–C bond to be cleaved in FMP and OAA and position of the corresponding bonds in the designed structures (**A**–**E**)).

The analysis of the proposed FAHD1 mechanisms suggests that replacement of the cleavable carbon–carbon bond in FAHD1 substrates by an uncleavable bond, while keeping the Mg^2+^-binding motif should lead to structures with inhibitory potential (Figure 3).

Exchange of the 3-methylene carbon by a nitrogen in fumarylpyruvate (FMP) leads to a flexible and reactive molecule **A** (depicted in Figure 3 and, also, for the following). The planar enone moiety present in FMP and **A** can be mimicked with an aromatic ring. The resulting molecule **B** is resonance-stabilized, capable of Mg^2+^ binding, and keeps the carboxy function in a similar position as in FMP, respectively. An equivalent consideration as described above can be applied to oxaloacetate (OAA). In case of OAA, the nitrogen exchange of the methylene group leads to a carbamic acid, which is not stable. Substitution by a further nitrogen changes the molecule to a reactive acyl-urea derivative **C**. As a mimetic of the urea substructure in **C**, a pyridine ring system can be introduced concomitant with conformational stabilization (**D**). Oxidation of the pyridine moiety to *N*-oxide **E** results in a structure presenting negative charge density in the space where substrate OAA^2−^ positions the carboxyl anion and acylpyruvates their oxyanion intermediate. The deduced scaffolds **B**, **D** and **E** have molecular weights far below 300 and would leave generous room for further SAR studies and optimization of the pharmacological parameters. The proposed molecules **B**, **D** and **E** belong to the class of oxalamic acid derivatives. Such molecules are known and easily accessible by proven experimental procedures.

## 2. Results and Discussion

### 2.1. N-Aryl-Oxalamic Acids as FAHD1 Inhibitors

**Figure 4:**
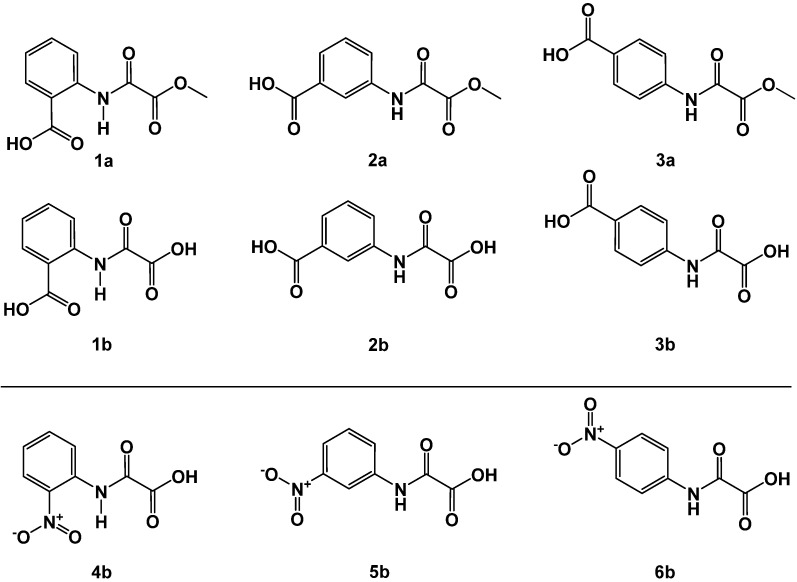
Oxalamic acids methyl esters **1a**–**3a** and corresponding acids **1b**–**3b** (**upper panel**); o-, m- and *p*-nitro-substituted *N*-phenyl-oxalamic acids **4b**–**6b** (**lower panel**).

First, we synthesized exemplaric known compounds of scaffold **B** (Figure 3) derived from anilines functionalized with polar carboxy or nitro groups, which present the charge density into the space of the oxyanion hole in the FAHD1 catalytic cavity. The acylation of amino-substituted benzoic acids yields oxalamic acid methyl esters (**1a**–**3a**) in high yields and purity. Analogously, the acylation of nitro-substituted anilines afforded after saponification oxalamic acids **4b**–**6b**. The compounds obtained from the carboxy series revealed instantly a good-to-moderate inhibition of FAHD1-ODx activity (Figure 4 and Table 1).

In the series of methyl esters **1a**–**3a**, we observed a remarkable change in activities as a function of the position of the carboxy substituent. The *o*-substituted **1a** shows only a moderate inhibition (IC_50_ = 47 µM), whereas the *m*-substituted congener **2a** was already comparable with the only known FAHD1 inhibitor oxalate (IC_50_ 13 µM vs. 7 µM). The *p*-carboxy-substituted derivative **3a** revealed a weak activity.

**1b** belongs (accordingly the design steps shown in Figure 3) to a fumarylpyruvate (FMP) mimetic. The *m*-phenyl carboxy group is presented at a similar position as in the FAHD1 substrate FMP, creating an additional interaction not accessible by *o*- and *p*-substituted congeners **2a** and **3a**, respectively. The *o*-carboxy derivative **1a** can be termed as a close mimetic of oxaloacetate. However, a six-membered hydrogen bonding between amide NH and oxygen of the *o*-carboxylate creates a resonace stabilized flat structure that impairs the presentation of negative charge density to the oxyanion hole of FAHD1. This conformational penalty is obviously romoved upon saponification to the acid **1b**, most probably due to electronic repulsion of the corboxy functions. Saponification of the FMP analog **2a** to the corresponding acid **2b** dit not change the inhibitory activity, suggesting that most of the binding energy of this derivative is carried by the *m*-substituent. The *p*-carboxy derivatives **3a** and **3b** present the *N*-aryl substituent in a position not covered by substrates OAA or FMP, which explains their reduced activity.

The 2-oxalylamino-benzoic acid **1b** is a known competitive inhibitor of protein-tyrosine phosphatase *β* [15]. The molecule behaved as a phosphate mimetic and has been used as a “minimal unit” mimetic for advanced and selective inhibitors of this enzyme, but **1b** apparently could also serve well for FAHD1 inhibitors. The low molecular weight of **1b** enables a broad derivatization for the establishment of a structure–activity relationship (SAR) for FAHD1. Somewhat surprising was that oxalamic acid methyl esters **1a**–**3a** kept active. Especially *meta*-substituted **2a** was equally potent copmpared to the *ortho*-carboxylate presenting **1b**. This finding is important, because acid functions are known to impair the cellular uptake. Ester derivatives could act as prodrugs, facilitating membrane crossing (for additional ester derivatives, see Section 2.3). We investigated further substituents on the *N*-aryl scaffold, such as hydroxy-, methoxy or halogen groups in various positions, but none of the synthesized compounds improved the IC_50_ below 30 µM (selected results are provided in the Supporting Materials).

**Figure 5:**
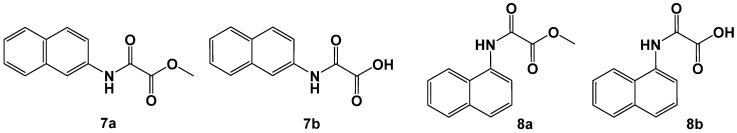
*N*-*β*-naphthyl-oxalamic acid methyl ester (**7a**) and -acid (**7b**) and *N*-*α*-naphthyl oxalamic acid analogs (**8a**, **8b**).

In the next step, we investigated *α*- and *β*-naphthylamines as examples for annulated ring systems. It was found that only *β*-substitution is appropriate for good inhibitors, wheras the *α*-naphthyl congeners sustain a dramatic loss of activity (Figure 5 and Table 2).

*N*-*β*-naphthyl-oxalamic acid methyl ester (**7a**) and -acid (**7b**) share a similarity with the *m*-carboxyl substituted derivatives shown in Figure 4 but still miss the charge presentation. The ester-acid pair **7a** and **7b** is a rare example where the saponification of the ester to the acid led to a significant loss of activity. The results warrant further investigations of annulation with heterocycles and substitution of the naphthyl moiety by polar functional groups.

Discouraging results were obtained from investigation of compounds where the *N*-aryl ring was spacered by an alkyl (e.g., *N*-benzyl or *N*-cycloalkyl motifs). Such derivatives revealed IC_50_ > 100 µM (for examples, see the Supporting Materials).

### 2.2. N-Pyridyl Substituted Oxalamic Acid Derivatives and Their N-Oxides as FAHD1 Inhibitors

In the following, we gave focus to synthesis of pyridine scaffolds (**D**) and their *N*-oxides (**E**), which were deduced from the OAA substrate (Figure 3). Heterocyclic *N*-oxides have been investigated in numerous medicinal chemistry projects and revealed anticancer, anti-inflammatory, antibacterial, anti-HIV and antiparasitic activities, among many others [16] Additionally, the *N*-oxide moiety serves as an important molecular recognition motif in biological interactions.

**Figure 6:**
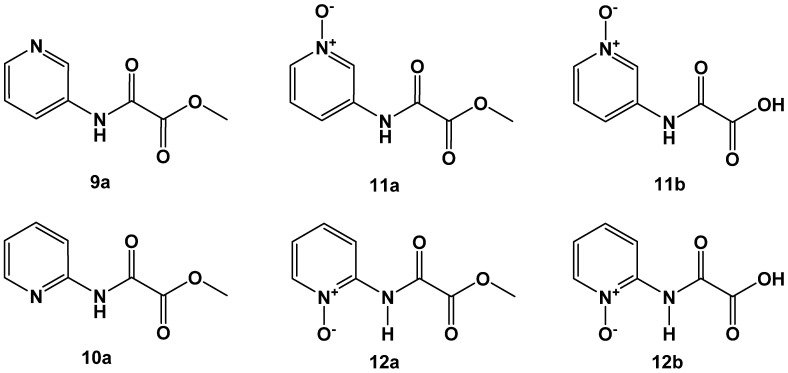
Oxalamic acid derived from 3-aminopyridine (**upper lane**) and 2-aminopyridine building blocks (**lower lane**).

We first synthesized 3-aminopyridine and 2-aminopyridine oxalamic acid methyl esters **9a** and **10a** (Figure 6). Clean oxidation of the heterocyclic ring nitrogen afforded the corresponding *N*-oxides **11a** and **12a**, which were saponified to the acids **11b** and **12b**. The compounds showed FAHD1 inhibitory activity, as proposed by the design strategy (Table 3). Derivatives **9a**, **11a** and **11b** obtained from the 3-aminopyridine building block turned out to be much superior in FAHD1 inhibitory potency compared to the 2-aminopyridine congeners **10a**, **12a** and **12b**. The 3-aminopyridine-derived compounds can deliver negative charge density towards the space of the oxyanion hole of FAHD1, stabilizing the binding of the inhibitor. In contrast, the 2-aminopyridine congeners present the charge density opposite to the oxyanion hole. Rotation around the C–N bond cannot correct for this obstacle. There is no evidence for a tautomerism from the NMR-spectra of the 2-aminopyridine-derived compounds.

**Figure 7:**
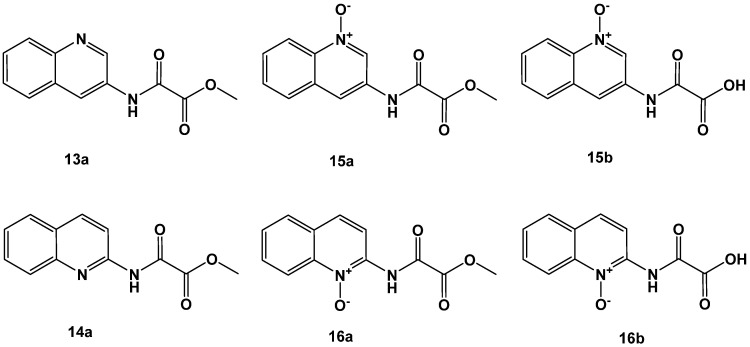
2-amino-(lower lane) and 3-amino-quinoline derivatives (upper lane).

To study the effect of annulation of the pyridine ring, 2-amino or 3-aminoquinoline building blocks were applied in the synthesis. The modification was especially beneficial for the 2-aminoquinolines (**14a**, **16a** and **16b**; Figure 7 and Table 4) compared to the nonanullated 2-aminopyridines (**10a**, **12a** and **12b**; Figure 6). The 3-aminoquinolines kept good activity with the 3-aminoquinoline *N*-oxide **15b** as the most active representative.

### 2.3. Variations Affecting the 1,2-Dicarbonyl Motif in Oxalamic Acid Derivatives

**Figure 8:**
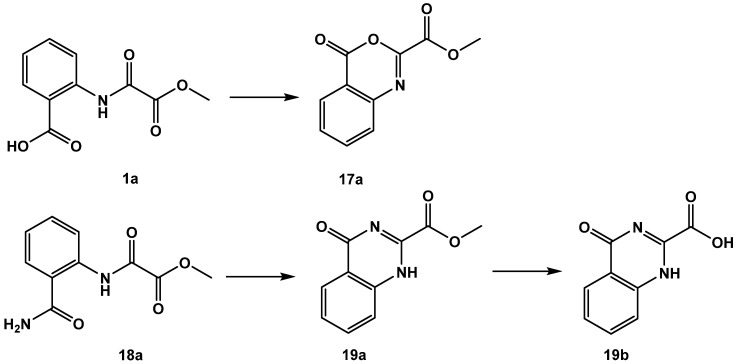
Products obtained from reactions under participation of the 1,2-dicarbonyl motif.

As disclosed by the single molecule X-ray diffraction analysis of FAHD1 with bound oxalate inhibitor, the 1,2-dicarbonyl motif is an essential structural element for bidentate binding to the magnesium cofactor. We explored several modifications in this motif. The oxalamic acid derivative **1a** was cyclized at an elevated temperature or upon treatment with acetic acid anhydride to a 4-oxo-4*H*-benzo[*d*]-[1,3]oxazine derivative **17a** [17]. Analogously, the *ortho*-amide-bearing methyl ester **18a** was cyclized to methyl 4-oxo-1,4-dihydroquinazoline-2-carboxylate **19a** [18]. The latter one was saponified to the acid congener **19b** (Figure 8). All these small molecules inhibited FAHD1 ODx activity in the low µM IC_50_ range (Table 5). It must be considered that the highly active **17a** is a reactive molecule able to acylate nucleophiles (but, so far, we have no evidence that **17a** is modifying residues in the catalytic center of FAHD1).

**Figure 9:**
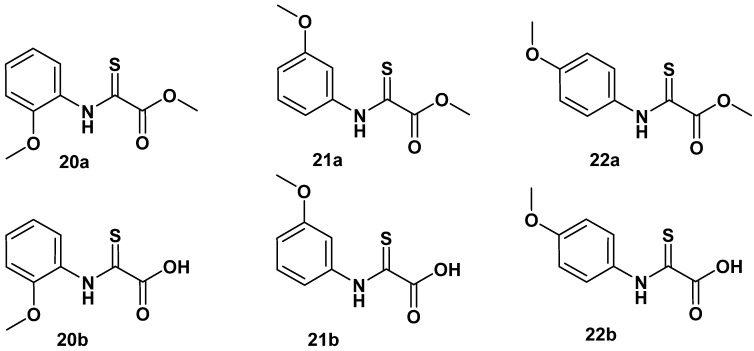
Selective exchange of 2-oxo function with the Lawesson reagent to 2-thio derivatives.

Formation of cyclic **17a** is propagated by a nucleophilic attack of 2-amide carbonyl oxygen on the activated *ortho*-carboxyl group of **1a** [17]**,** disclosing a significant polarization of the 2-amide function towards the oxygen atom, which supports strong bidentate binding to the enzyme cofactor and gains stability towards hydrolysis of the amide bond in oxalamic acids. The nucleophilic property of the 2-oxygen in oxalamic acid derivatives inspired us to investigate the reaction with a Lawesson reagent (CAS RN 19172-47-5). We found a clean specific exchange of the 2-carbonyl in oxalamic acid esters to the 2-thio congeners **20a**–**22a**. Saponification of the methyl esters afforded the acid derivatives **20b**–**22b** (Figure 9). 

However, these transformations did not improve the inhibitory activity, as summarized in Table 6.

**Figure 10:**
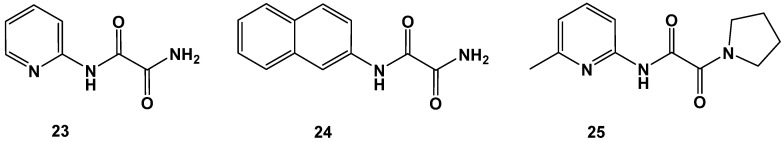
Examples of oxalamic acid diamides (**23–25**).

Change of oxalamic esters to oxalamic acid amides was also not a favorable transformation, as revealed by derivatives **23–25**. *C*-terminal cycloalkyl amide **25** was an inferior compound (Figure 10 and Table 7).

**Figure 11:**
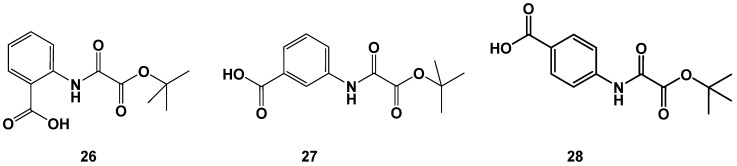
Identification of highly active *tert*. butyl esters **26** and **28**.

A surprising result became visible when we tested a variation in the terminal ester functionality. Change of methyl esters to *tert*. butyl esters in the series of *ortho*-, *meta-* and *para*-aminobenzoic acids yielded two highly potent inhibitors **26** and **28**, whereas the *meta*-substitution (**27**) was not well-tolerated (Figure 11 and Table 8). Comparison of the *tert.* butyl esters **26–28** with the corresponding methyl esters **1a**–**3a** (see Figure 5 and Table 1) reveals that the modification is beneficial for the methyl esters with lower activity. **1a** gained a four-fold and **3a** even a six-fold increase in activity, whereas the highly active *m*-carboxy-substituted **2a** sustains a twenty-fold loss in activity upon the change from a methyl to *tert.* butyl ester.

However, the catalytic center of FAHD1 (as derived from the oxalate ligated FAHD1 X-ray structure) does not feature enough space for the accommodation of a hydrophobic and bulky *tert*. butyl residue. Especially the activity increase of the *p*-carboxy-substituted **28**, which carries the charge presenting substituent at an unfavorable position (s. **3a**, Figure 4), points towards a drastic reorganization within the catalytic center of FAHD1 upon the binding of these structures. These findings need further investigations. The derivatizations of *tert*. butyl esters were extended from the aminobenzoic acids to other scaffolds and resulted in additional compounds with high inhibitory activity (see the Supporting Materials).

Starting from these observations, and despite the initially negative experience made with variations in the terminal amide region, we explored symmetric oxalamic acid diamides for FAHD1 inhibition.

### 2.4. N,N′-Diaryl-Oxalamic Acid Diamides

**Figure 12:**
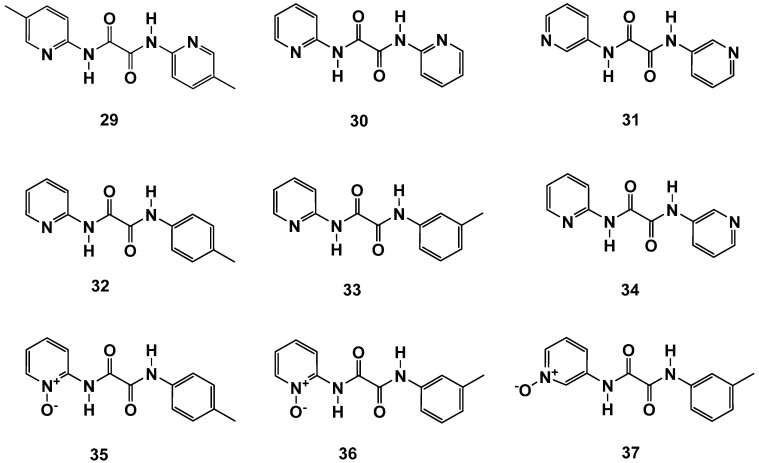
*N*,*N*′-diaryl-oxalamic acid diamides with C2 symmetry and broken symmetry.

Surprisingly, the synthesis starting with 2-aminopyridine delivered highly active C_2_-symmetrical compounds (e.g., **29** and **30**; Figure 12 and Table 9). Symmetry was not a prerequisite for high activity, as demonstrated by compounds **32** and **33**. The C_2_-symmetrical combination of *N*,*N*′-bis-3-aminopyridyl led to a significant loss of activity (**31**). The combination of *N*-2-aminopyridyl with *N*′-3-aminopyridyl (**37**) even resulted in a total loss of activity. These results point towards a so-far-undisclosed specific interaction of these compounds within FAHD1, which deserves further investigations. The *N*,*N*′-diamide scaffold cannot be compared with the compounds described in Section 2.2, which are capable of bidentate binding to the Mg cofactor of FAHD1. The *N,N*′-diamide molecules adopt a planar structure with the 1,2-carbonyls in *s-trans* conformation [19] (rotational barrier ~ 12 kcal/mol), which does not support bidentate Mg complexation. The interpretation of the activities may become even more complex when taking into account, that the oxalic acid diamides present recognition motifs for *β*-sheets or backbone amide bonds (present, e.g., in the exposed FAHD1 lid domain).

We also synthesized novel *N*-oxides of *N*,*N*′-diamides (**35–37**) and achieved good results with 2-aminopyridine-based compounds **35** and **36**, whereas the 3-aminopyridyl containing derivative **37** was found to be inactive. Obviously, the *N*′-*m*-toluidine amide in **37** destroys the activity of acid congener **11b** (Figure 6 and Table 3), which represents one of the most active compounds found in this study. In contrast, the 2-aminopyridyl-derived *N*′-*m*-toluidine amide **36** gained significant activity compared to its acid congener **12b**. The so-far-collected results for the *N*,*N*′-diamide scaffold deserve a broad extension of SAR studies with this novel FAHD1 inhibitor scaffold.

*N*,*N*′-dipyridyl-containing compounds could also be oxidized to corresponding *bis*-*N*,*N*′-oxides, but the compounds obtained were unfortunately not soluble.

Taking together, the data for *tert*. butyl esters and the *N*,*N*′-diaryl-oxalamic acid diamides, an inhibition mode different to oxalate, can be assumed for these scaffolds. The discovery of the scaffold expands the initially applied design strategy significantly. To clarify the binding mode of these molecules, we initiated already co-crystallization experiments to create a solid basis for the future design of second-generation FAHD1 inhibitors to be active in the nanomolar regime.

## 3. Materials and Methods

Solvents, reagents and building blocks were purchased from various suppliers and used without further purification. Ethyl acetate and *n*-hexane (mixture of isomers) were distilled prior use in chromatographic separations.

### 3.1. Reaction Monitoring and Purification of Compounds

#### 3.1.1. Thin-Layer Chromatography (TLC)

Reaction monitoring was performed by thin-layer chromatography (TLC) on Merck silica gel 60-F_254_ glass plates or on Macherey & Nagel POLYGRAM SIL G/UV 254 aluminium foils. The plates were developed with mixtures of hexane/ethyl acetate and methanol/ethyl acetate. Compound spots were visualized by UV (254 nm) irradiation or in TLC chamber containing iodine adsorbed on silica gel.

#### 3.1.2. Middle Pressure Liquid Chromatography (MPLC)

Purification of compounds was performed by middle-pressure liquid chromatography (MPLC) on silica gel 60 from Merck (0.040–0.063 µm, 240–400 mesh). The unique home built MPLC system was provided by MoleculeCrafting.HuGs e.U. (Hubert Gstach) and consisted of columns, an FMI pump (Fluid Metering, Inc., Syosset, NY, USA) and an Amersham Superfrac fraction collector.

### 3.2. Analytical Characterization

#### 3.2.1. High-Resolution Mass Spectroscopy (HRMS)

HRMS measurements were performed on Bruker maxis HD with ESI-q-TOF setup via direct infusion with a flow rate of 180 µL × h^−1^. ESI parameter: End Plate Offset 500 V, capillary 1500 V, nebulizer 0.4 bar, dry gas 4.0 L × min^−1^ and dry temperature 200 °C. Tune parameter: Transfer Funnel 1 RF 300.0 Vpp, isCID Energy 0.0 eV, Multipole RF 100.0 Vpp, Quadrupole Ion Energy 2.0 eV and Low Mass m/z 70.00.

#### 3.2.2. Melting Points

Melting points (Mp) were determined with a Bausch & Lomb microscope equipped with a Kofler melting stage and are uncorrected.

#### 3.2.3. Nuclear Magnetic Resonance Spectroscopy (NMR)

NMR spectra were recorded at the NMR Centre of the Faculty of Chemistry, University of Vienna, either on a DRX 600 WB spectrometer (resonance frequencies 600.25 MHz for ^1^H, 150.93 MHz for ^13^C) or on an Avance III HDX 700 spectrometer equipped with a quadruple QCIF cryoprobe (700.40 MHz for ^1^H, 176.12 MHz for ^13^C), both Bruker instruments (Bruker BioSpin, Rheinstetten, Germany).

The software used for the processing of 1D- (^1^H, ^13^C) and 2D- (COSY, HMBC and HSQC) NMR spectra was SpinWorks 4.2.0.0 (copyright 2015, Kirk Marat, University of Manitoba). Coupling constants (*J*) are given in Hertz (Hz) and refer to the first order interpretation (apparent coupling constants *J*_app_ are provided). Assignment of resonances was performed with COSY, HSQC and HMBC, respectively. ^x^*J* refers to homonuclear HH-coupling over x bonds. ^x^*J*_CF_ refers to heteronuclear CF coupling over x bonds. Solvents used for NMR spectroscopy: CDCl_3_, chloroform-d_1_ (CAS RN 865-49-6), was filtered through basic, activated aluminium oxide (Sigma Aldrich) prior to use; DMSO-d_6_, hexadeuterio dimethyl sulfoxide (CAS RN 2206-27-1) was stored over a molecular sieve (4 Å). Chemical shift calibration [20]: CDCl_3_, ^1^H δ = 7.26, ^13^C δ = 77.16; DMSO-d_6_, ^1^H δ = 2.50, ^13^C δ = 39.52 ppm. NMR characterization: br, broad; multiplicity: m, multiplet; s, singlet; d, doublet; t, triplet; q, quaternary or quartet; m [e.g., t, dd, q, tt and sext] notation in brackets describes the overall appearance of the signal pattern; 2D NMR techniques used for the assignment of ^1^H and ^13^C resonance signals: HSQC, Heteronuclear Single Quantum Coherence; HMBC, Heteronuclear Multiple Bond Correlation; COSY, Correlation Spectroscopy.

### 3.3. Inhibitor Assay on the 96-Well Plate

The target inhibitor class features UV absorption in the region around 255 nm, where oxaloacetate is absorbing as well, while the absorbance of the product pyruvate is negligible at this wavelength. It can be observed that lower concentrations of OAA display an initial raise of the OD 255 nm signal. The steady state is typically reached at about 5 min. This observation can be attributed to an increase of the OAA-enol form, indicating that not all OAA introduced into the assay mixture was already in the enol form. The assay starts with 100% bound enol form of OAA. From our literature survey of metal catalyzed OAA decarboxylation, we can conclude that the rate determining step of the decarboxylation is slow ketonization. The enol form of OAA is not prone to decarboxylation (as suggested by theory). We speculate that our designed inhibitors compete with the transient enol form of OAA.

In a direct assay on the 96-well plate (Table 10), ODx activity of FAHD1 and its inhibition is performed as follows: We used a freshly prepared 20-mM OAA solution in ddH_2_O and any dilutions of an original 100-mM inhibitor solution (dilution “0”), which we refer to as dilution “0” to “6” that cover the range of mM down to nM inhibition. We used a 40-µM solution of recombinant protein [21], which was freshly prepared from working solutions of about 1 µg/µL, as determined via the Pierce™ BCA Protein Assay Kit (Thermo). Three rows on a 96-well plate (wells labeled 1–12) are used for one inhibitor in order to have triplets (*n* = 3). Well 1 was used to check for the auto-decarboxylation of OAA. Well 2 was used to check for possible reactions of the inhibitor with OAA. Well 3 was used to check for FAHD1 stability in the presence of higher inhibitor concentrations. Well 4 was used to check for the inhibitor stability and OD signal over time. Well 5 was used to measure the uninhibited FAHD1-specific activity. Wells 6–12 were used for titrations of the inhibitor.

### 3.4. General Synthetic Procedures

**Scheme 1:**
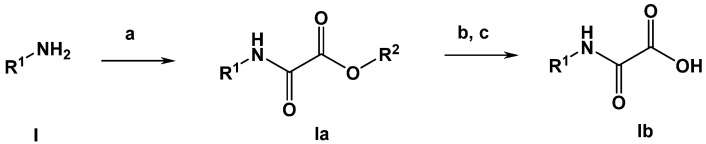
General synthesis of oxalamic acid derivatives (R^2^: methyl or tert. -butyl). Conditions: (**a**) methyl or tert. butyl 2-chloro-2-oxoacetate, triethylamine, DCM or DMF; (**b**) 2*N* NaOH, ethanol, 2*N* HCl or (**c**) K_2_CO_3_, H_2_O-MeOH; 2*N* HCl for **4b**–**6b**.

Oxalamic acid derivatives can be obtained by kown methods. The generally applid reaction path is depicted in Scheme 1.

#### 3.4.1. General Synthesis of Oxalamic Acid Derivatives

To a solution of the desired amine (0.5 mmol) in *N*,*N*-dimethylformamide (dichloromethane for anthranilic acid) (10 mL) was added 70 µL of triethylamine (0.5 mmol). The solution was cooled to 0 °C. To the stirred mixture, a solution of methyl 2-chloro-2-oxoacetate (CAS RN. 5781-53-3; 1.1 equivalents) in 10 mL of dichloromethane was added dropwise within 10 min. The reaction mixture was stirred at room temperature for 3 h. Subsequently, the reaction mixture was diluted with 50 mL of ethyl acetate. The organic layer was extracted three times with 20 mL of HCl (2*N*), dried over Na_2_SO_4_ and evaporated under reduced pressure. The crude product was purified by column chromatography on silica gel using ethyl acetate/n-hexane (2:1) as the eluent. The crystalline product obtained was finally dried in vacuo.

**Scheme 2:**
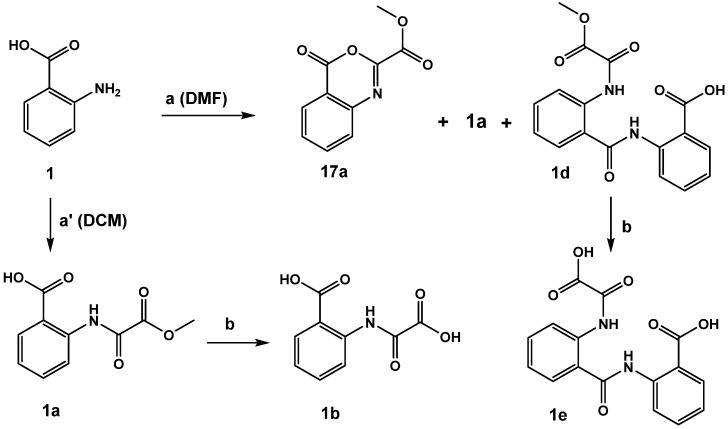
Conditions: (**a**) methyl 2-chloro-2-oxoacetate, triethylamine, DCM or DMF. (**b**) 2*N* NaOH, ethanol, 2*N* HCl.

The acylation of anthranilic acid **1** turned out to be solvent-dependent (Scheme 2). Reaction performed in DCM led in 98% yield to the desired methyl ester product **1a**, wherase the reaction in DMF resulted in a complex mixture containing the desired product **1a** as a minor component, methyl 4-oxo--benzo[*d*][1,3]oxazine-2-carboxylate (**17a**) as the major product and little of 2-(2-(2-methoxy-2-)-benzamido)benzoic acid **1d**. By-product **1d** formed upon reaction of **17a** with unreacted starting material **1**. Saponification of **1d** yielded the corresponding diacid **1e** (both compounds revealed moderate inhibitory activity).

#### 3.4.2. Saponification of Methyl Ester Derivatives

To a solution of a methyl ester derivativ in 5 mL of methanol was added NaOH (2*N*, 2eq.). The resulting mixture was stirred for 10 min and finally quenched with HCl (2*N*). The mixture obtained was extracted three times with ethyl acetate. The organic phases were combined, dried over Na_2_SO_4_ and evaporated. The crystalline product obtained was dried in vacuo.

#### 3.4.3. General Synthesis of 2-Thiooxoacetate Derivatives

**Scheme 3:**
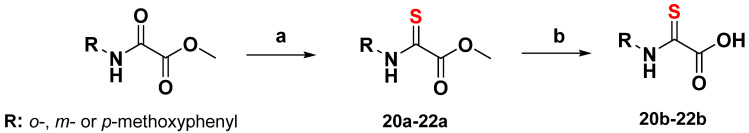
Conditions: (**a**) Lawesson’s reagent, toluene; (**b**) 2*N* NaOH, ethanol; 2*N* HCl.

Lawesson’s reagent (CAS RN 19172-47-5) (0.20 mmol) was added to a solution of a methyl ester derivate **(S2**, **S3**, or **S4**) (0.40 mmol) in toluene (2 mL). The reaction mixture was stirred at 75 °C for 7 h. The crude mixture was concentrated under reduced pressure, and the product was purified by column chromatography (n-hexane/ethyl acetate 10:1). The crystalline product (**20a**, **21a**, **22a**) was dried in vacuo before being dissolved in ethanol (5 mL) and saponified with NaOH (2*N*, 2eq.). The resulting mixture was stirred for 3 h and, finally, quenched with HCl (2*N*). The product obtained was extracted several times with ethyl acetate. The organic layers were combined, dried over Na_2_SO_4_ and evaporated. The crystalline product (**20b**, **21b** and **22b**) obtained was dried in vacuo (yield: 90–95%) (Scheme 3).

#### 3.4.4. Synthesis of Pyridine and Quinoline N-Oxides

To a solution of the corresponding oxalamic acid ester (methyl or *tert.* butyl) in dichloromethane, 1.1 equivalents of *m*-chloroperoxybenzoic acid (*m*-CPBA) were added. The reaction mixture was stirred overnight at room temperature. The solvent was removed under reduced pressure. To the obtained reaction mixture, cold di-isopropyl ether (DIPE) was added. The obtained heterogeneous slurry was vigorously stirred for 2 h. Subsequently, the formed crystals were collected by filtration and washed three times with cold DIPE. The crystals were dried under reduced pressure. (Note: some of the compound’s form defined crystalline 1:1 clathrate with 3-chlorobenzoic acid).

#### 3.4.5. Synthesis of Oxalamic Acid Amides

**Scheme 4:**
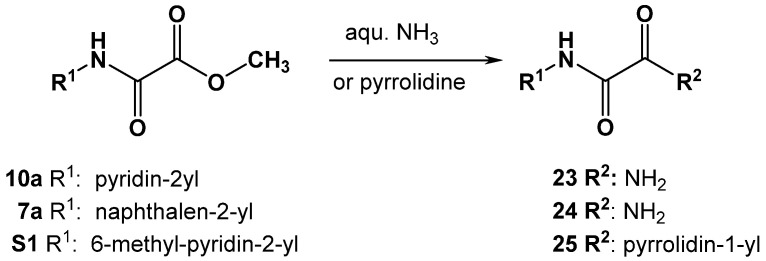
Synthesis of oxalamic acid amide derivatives **23**, **24** and **25**.

Oxalamic acid amides were prepared by aminolysis of *N*-aryl oxalamic acid methyl esters (Scheme 4).

To a solution of oxalamic acid methyl ester (**10a**, **7a** or **S1**) in 5 mL of toluene, the corresponding amine was added (aqueous ammonia for **23**, **24** or 1.1 equivalents of pyrrolidine for **25**). The reaction mixture was stirred at room temperature in a sealed vessel. The reaction status was monitored by TLC. Toluene was removed under reduced pressure. Subsequently, the obtained oily residue was diluted with ethyl acetate. The organic phase was washed three times with deionized water. The organic layer was dried over Na_2_SO_4_ and evaporated under reduced pressure. The crystalline product (**23–25**) obtained was dried in vacuo (yields: 45–60%).

#### 3.4.6. Synthesis of *N*,*N*′-Diaryl-Oxalamic Acid Diamides

**Scheme 5:**
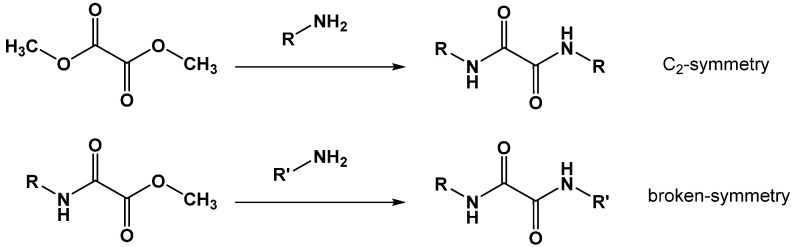
Synthesis of oxalamic acid amide derivatives **29–37**.

*N*,*N*′-diaryl-oxalamic acid diamides with C_2_-symmetry can be achieved in one step by reacting an amine scaffold with oxalic acid dimethyl ester (DIMOX) (method A). Nonsymmetric *N*,*N*′-diaryl-oxalamic acid diamides are best obtained by aminolysis of *N*-aryl-oxalamic acid methyl esters with an *N*′-amine building block (method B) (Scheme 5).

##### Synthesis of Oxalic Acid Diamides by Aminolysis of DIMOX

Typical example for method A: Synthesis of *N^1^,N^2^*-di(pyridin-2-yl)oxalamide (**30**).

Two equivalents of pyridin-2-amine and one equivalent of dimethyl oxalate (DIMOX) were dissolved in DMF by slight heating. The homogeneous solution obtained was heated to 120 °C and kept under stirring in a nitrogen atmosphere overnight. The reaction mixture was cooled to room temperature. Subsequently, the reaction mixture was diluted with ethyl acetate. The organic phase was washed three times with brine, dried over Na_2_SO_4_ and filtered. The solvent was removed on a rotary evaporator under reduced pressure. The pure product **30** was obtained by MPLC separation (stationary phase: silica gel; eluent: appropriate mixture of *n*-hexane and ethyl acetate); Yield: 44% (the reactions did not go to full completion; for the analytical characterization of compound **30**, see the Supplementary Materials).

##### Synthesis of Oxalic Acid Diamides by Aminolysis of Methyl Esters

Typical example for method B: Synthesis of *N^1^*-(pyridin-2-yl)-*N^2^*-(m-tolyl)oxalamide (**33**).

Methyl ester **10** and 1.1 eq. of *m*-toluidine were dissolved in DMF. The solution obtained was heated to 110 °C and stirred under nitrogen atmosphere overnight. The reaction mixture was cooled to room temperature and diluted with ethyl acetate. The organic phase was washed three times with brine, dried over Na_2_SO_4_ and filtered. The solvent was removed on a rotary evaporator under reduced pressure. The products were purified by MPLC separation (stationary phase: silica gel; eluent: appropriate mixture of *n*-hexane and ethyl acetate). Yield: 50% (for the analytical characterization of compound **33**, see the Supporting Materials).

## 4. Conclusions

We addressed the central chemical step of both FAHD1 functions (ODx- and ApH) by a theoretical approach. The results obtained from this approach verified a mechanism-based design concept of first-generation FAHD1 inhibitors. Replacement of the common scissile C–C bond in FAHD1 substrates by a simple carbon–nitrogen bond, together with the negative charge density presentation in the appropriate space of the catalytic cavity, turned out to be successful for the identification of the first generation of FAHD1 inhibitors. As a surprising result from the synthesis efforts, we identified the molecules that suggested a novel binding mode for inhibition of FAHD1. Valuable proposals for the synthesis of inhibitors could be deduced, and co-crystallization and STD-NMR experiments with selected compounds have been initiated.

We anticipate that a successful elucidation of the role of FAHD1 as a therapeutic target will stimulate translational research, with the aim to contribute to new therapeutic strategies for human malignancies. The current attempts to develop small molecules with the ability to modulate FAHD1 catalytic activity aim at translational strategies to fine-tune FAHD1 activity in physiological and pathological conditions. We will continue to synthesize and analyze FAHD1 novel and more potent inhibitor candidates, starting from the structure of existing inhibitors with the one-digit micromolar functional IC_50_ described in this work. After screening for binding affinity, the most promising molecules will be used for the first experiments with selected human cell lines expressing FAHD1, which will be treated with the most active inhibitors (and their methyl esters to enhance cell permeability) in different concentrations. We anticipate that the inhibition of FAHD1 over longer periods will induce the mitochondrial dysfunction in human cells. Such a model would provide a new important research tool and serve as the basis for new therapeutic molecules for human malignancies.

## Figures and Tables

**Table 1:** Carboxy substituted *N*-aryl oxalamic acid derivatives **1a**–**3b**. Nitro-substituted *N*-aryl oxalamic acids **4b**–**6b**. Data fit with GRAPHPAD PRISM 5. Standard error of the fit given in µM. IC_50_ diagrams, including the fit and standard deviations of the individual experiments (*n* = 3), are depicted in the Supplementary Materials.

Compound ID	IC_50_ ± Std. Error (µM)
**1a**	47 ± 1
**2a**	13 ± 1
**3a**	85 ± 2
**1b**	11 ± 1
**2b**	14 ± 1
**3b**	32 ± 2
**4b**	29 ± 2
**5b**	31 ± 2
**6b**	789 ± 6
**OAA**	6 ± 2

**Table 2:** *N*-*β*-naphthyl oxalamic acid derivatives (**7a**, **7b**). *N*-*α*-naphthyl oxalamic acid derivatives (**8a**, **8b**). Data fit with GRAPHPAD PRISM 5. Standard error of the fit given in µM. IC_50_ diagrams including the fit and standard deviations of the individual experiments (*n* = 3) are depicted in the Supplementary Materials.

Compound ID	IC_50_ ± Std. Error (µM)
**7a**	11 ± 1
**7b**	26 ± 2
**8a**	280 ± 2
**8b**	158 ± 1
**OAA**	6 ± 2

**Table 3:** *N*-(pyridine-3-yl)oxalamic acid derivatives **9a**–**11b**. *N*-(pyridine-2-yl) oxalamic acid derivatives **10a**–**12b**. Data fit with GRAPHPAD PRISM 5. Standard error of the fit given in µM. IC_50_ diagrams, including the fit and standard deviations of the individual experiments (*n* = 3), are depicted in the Supplementary Materials.

Compound ID	IC_50_ ± Std. Error (µM)
**9a**	27 ± 2
**10a**	249 ± 1
**11a**	29 ± 2
**11b**	4 ± 2
**12a**	168 ± 1
**12b**	65 ± 1
**OAA**	6 ± 2

**Table 4:** *N*-(quinolin-3-yl)oxalamic acid derivatives **13a**, **15a** and **15b**. *N*-(quinolin-2-yl)oxalamic acid derivatives **14a**, **16a** and **16b**. R Data fit with GRAPHPAD PRISM 5. Standard error of the fit given in µM. IC_50_ diagrams, including the fit and standard deviations of the individual experiments (*n* = 3), are depicted in the Supplementary Materials.

Compound ID	IC_50_ ± Std. Error (µM)
**13a**	33 ± 2
**14a**	33 ± 2
**15a**	31 ± 2
**15b**	8 ± 1
**16a**	51 ± 1
**16b**	31 ± 2
**OAA**	6 ± 2

**Table 5:** Modifications of the 1,2-dicarbonyl motif. 4-oxo-4*H*-benzo[*d*]-[1,3]oxazine **17a** obtained from **1a**. Derivatives **19a** and **19b** obtained from the reaction of the anthranilic acid amide derivative **18a**. Data fit with GRAPHPAD PRISM 5. Standard error of the fit given in µM. IC_50_ diagrams, including the fit and standard deviations of the individual experiments (*n* = 3), are depicted in the Supplementary Materials.

Compound ID	IC_50_ ± Std. Error (µM)
**1a**	47 ± 1
**17a**	11 ± 2
**18a**	59 ± 2
**19a**	43 ± 2
**19b**	30 ± 1
**OAA**	6 ± 2

**Table 6:** *N*-(*o*-, *m*-, *p*-methoxyphenyl)-2-thio oxalamic acid methyl esters (**20a**–**22a** and the corresponding acids **21b**–**22b**. Data fit with GRAPHPAD PRISM 5. Standard error of the fit given in µM. IC_50_ diagrams, including the fit and standard deviations of the individual experiments (*n* = 3), are depicted in the Supplementary Materials.

Compound ID	IC_50_ ± Std. Error (µM)
**20a**	-
**21a**	85 ± 3
**21b**	63 ± 2
**22a**	130 ± 1
**22b**	167 ± 1
**20b**	295 ± 2
**OAA**	6 ± 2

**Table 7:** Pyridin-2-yl (**23**), naphthyl-2-yl (**24**) and pyrrolidine-1-yl (**25**) oxalamic acid amides. Data fit with GRAPHPAD PRISM 5. Standard error of the fit given in µM. IC_50_ diagrams, including the fit and standard deviations of the individual experiments (*n* = 3), are depicted in the Supplementary Materials.

Compound ID	IC_50_ ± Std. Error (µM)
**23**	137 ± 3
**24**	107 ± 1
**25**	324 ± 2
**OAA**	6 ± 2

**Table 8:** *N*-(*o*-, *m*- or *p*-carboxy-phenyl) oxalamic acid *tert.* butyl esters. Data fit with GRAPHPAD PRISM 5. Standard error of the fit given in µM. IC_50_ diagrams, including the fit and standard deviations of the individual experiments (*n* = 3), are depicted in the Supplementary Materials.

Compound ID	IC_50_ ± Std. Error (µM)
**26**	12 ± 3
**27**	261 ± 2
**28**	15 ± 2
**OAA**	6 ± 2

**Table 9:** C_2_-symmetric *N^1^*,*N^2^*-bis(pyridin-2-yl or -3-yl) oxalamides (**29–31**). *N^1^*,N^2^- diaryl oxalamic acid amides with broken symmetry (**32–34**). Pyridine 1-oxide derivatives (**35–37**) obtained from the compounds with broken symmetry. Data fit with GRAPHPAD PRISM 5. Standard error of the fit given in µM. IC_50_ diagrams, including the fit and standard deviations of the individual experiments (*n* = 3), are depicted in the Supplementary Materials.

Compound ID	IC_50_ ± Std. Error (µM)
**29**	3 ± 2
**30**	8 ± 2
**31**	131 ± 1
**32**	3 ± 2
**33**	8 ± 2
**34**	inactive
**35**	17 ± 1
**36**	14 ± 2
**37**	inactive
**OAA**	6 ± 2

**Table 10:** Pipetting scheme for a direct ODx assay on the 96-well plate, to screen for IC_50_ values of FAHD1 in vitro.

Well Nr.	Buffer	FAHD1 (40 µM) (2 µM Final; ~5 µg)	DMSO (1% Final)	Inhibitor (in DMSO) (1% Final DMSO)	OAA (20 mM) (1 mM Final)
1	94 µL	---	1 µL	---	5 µL
2	94 µL	---	---	1 µL of dilution “0”	5 µL
3	94 µL	5 µL	---	1 µL of dilution “0”	---
4	99 µL	---	---	1 µL of dilution “0”	---
5	89 µL	5 µL	1 µL	---	5 µL
6	89 µL	5 µL	---	1 µL of dilution “6”	5 µL
7	89 µL	5 µL	---	1 µL of dilution “5”	5 µL
8	89 µL	5 µL	---	1 µL of dilution “4”	5 µL
9	89 µL	5 µL	---	1 µL of dilution “3”	5 µL
10	89 µL	5 µL	---	1 µL of dilution “2”	5 µL
11	89 µL	5 µL	---	1 µL of dilution “1”	5 µL
12	89 µL	5 µL	---	1 µL of dilution “0”	5 µL

## Data Availability

Data is contained within the article or supplementary material.

## Supplementary Materials

The following are available online. A comprehensive discussion of the “many faces” of FAHD1 substrate oxaloacetate (OAA), additional compounds (S5-S22) investigated for FAHD1 inhibition (Figure S4), all analytical data for compounds synthesized, as well as titration curves for compounds discussed in the manuscript.

## Sample Availability

Samples of the compounds described in the manuscript and in supplementary materials are available from the corresponding authors.
