# Supplementary material for: Inhibitors of Fumarylacetoacetate Hydrolase Domain Containing Protein 1 (FAHD1)
Source: Molecules. 2021 Aug 18;26(16):5009. doi: 10.3390/molecules26165009 (PMC8398924; doi:10.3390/molecules26165009)
Supplement: Supplementary file 1 [file molecules-26-05009-s001.zip › molecules-1308499-SI.pdf]

molecules

Supporting Materials

Inhibitors of Fumarylacetoacetate Hydrolase Domain Containing Protein 1 (FAHD1)

Alexander K. H. Weiss*, Richard Wurzer, Patrycia Klapec, Manuel Philip Eder, Johannes R. Loeffler, Susanne von Grafenstein, Stefania Monteleone, Klaus R. Liedl, Pidder Jansen-Dürr*, Hubert Gstach*

Content

1. Compounds described in the manuscript
2. Starting materials and intermediates used in synthesis
3. Additional compounds from SAR
4. A comprehensive discussion of the "many faces" of substrate oxaloacetate (OAA)
5. Additional compounds investigated for FAHD1 inhibition (Figure S4)
6. Analytical data
7. Graphical representation of NMR-spectra
8. FAHD1-inhibitor titration curves
9. References provided in SM

- **Compounds described in the manuscript:**

2-(2-methoxy-2-oxoacetamido)benzoic acid **(1a)** (RN: 174575-83-8)

2-(carboxyformamido)benzoic acid **(1b)** (RN: 5651-01-4)

2-(2-(2-methoxy-2-oxoacetamido)benzamido)benzoic acid **(1d)**

2-(2-(carboxyformamido)benzamido)benzoic acid **(1e)**

3-(2-methoxy-2-oxoacetamido)benzoic acid **(2a)**

3-(Carboxyformamido)benzoic acid **(2b)** (RN: 58465-46-6)

4-(2-methoxy-2-oxoacetamido)benzoic acid **(3a)** (RN: 1086273-37-1)

4-(carboxyformamido)benzoic acid **(3b)** (RN: 14121-56-3)

Methyl 2-((2-nitrophenyl)amino)-2-oxoacetate **(4a)**

2-((2-nitrophenyl)amino)-2-oxoacetic acid **(4b)** (RN: 77901-51-0)

Methyl 2-((3-nitrophenyl)amino)-2-oxoacetate **(5a)** (RN: 1237427-49-4)

2-((3-nitrophenyl)amino)-2-oxoacetic acid**(5b)** (RN: 6274-26-6)

Methyl 2-((4-nitrophenyl)amino)-2-oxoacetate **(6a)** (RN: 82633-22-5)

2-((4-nitrophenyl)amino)-2-oxoacetic acid **(6b)** (RN: 103-94-6)

Methyl 2-(naphthalen-2-ylamino)-2-oxoacetate **(7a)** (RN: 20518-40-5)

2-(naphthalen-2-ylamino)-2-oxoacetic acid **(7b)** RN: 81682-60-2)

Methyl 2-(naphthalen-1-ylamino)-2-oxoacetate **(8a)** (RN: 254751-07-0

2-(naphthalen-1-ylamino)-2-oxoacetic acid **(8b)** (RN: 21660-76-4)

Methyl 2-oxo-2-(pyridin-3-ylamino)acetate **(9a)** (RN: 480452-68-4)

3-(2-Methoxy-2-oxoacetamido)pyridine 1-oxide **(11a)**

3-(Carboxyformamido)pyridine 1-oxide **(11b)**

Methyl 2-oxo-2-(pyridin-2-ylamino)acetate **(10a)**

2-(2-Methoxy-2-oxoacetamido)pyridine 1-oxide **(12a)**

2-(Carboxyformamido)pyridine 1-oxide **(12b)**

Methyl 2-oxo-2-(quinolin-3-ylamino)acetate **(13a)**

Methyl 2-oxo-2-(quinolin-2-ylamino)acetate **(14a)**

3-(2-Methoxy-2-oxoacetamido)quinoline 1-oxide **(15a)**

3-(Carboxyformamido)quinoline 1-oxide **(15b)**

2-(2-Methoxy-2-oxoacetamido)quinoline 1-oxide **(16a)**

2-(Carboxyformamido)quinoline 1-oxide **(16b)**

Methyl 4-oxo-4*H*-benzo[d][1,3]oxazine-2-carboxylate **(17a)** (RN: 3603-20-1)

Methyl 2-((2-carbamoylphenyl)amino)-2-oxoacetate **(18a)** (RN: 69065-88-9)

Methyl 4-oxo-1,4-dihydroquinazoline-2-carboxylate **(19a)** (RN: 63569-82-4)

4-oxo-1,4-dihydroquinazoline-2-carboxylic acid **(19b)** (RN: 29113-34-6)

Methyl 2-((2-methoxyphenyl)amino)-2-thioxoacetate **(20a)**

2-((2-methoxyphenyl)amino)-2-thioxoacetic acid **(20b)** (RN: 7267-58-5)

Methyl 2-((3-methoxyphenyl)amino)-2-thioxoacetate **(21a)**

2-((3-methoxyphenyl)amino)-2-thioxoacetic acid **(21b)** (RN: 276693-17-5)

Methyl 2-((4-methoxyphenyl)amino)-2-thioxoacetate **(22a)**

2-((4-methoxyphenyl)amino)-2-thioxoacetic acid **(22b)** (RN: 946-61-2)

N^1-(pyridin-2-yl)oxalamide **(23)** (RN: 52781-00-7)

N^1-(naphthalen-2-yl)oxalamide **(24)** **(**RN: 21775-72-4)

N-(6-methylpyridin-2-yl)-2-oxo-2-(pyrrolidin-1-yl)acetamide **(25)** (RN:1210298-59-1)

2-(2-*tert*-Butoxy-2-oxoacetamido)benzoic acid **(26)**

3-(2-*tert*-Butoxy-2-oxoacetamido)benzoic acid **(27)**

4-(2-*tert*-Butoxy-2-oxoacetamido)benzoic acid **(28)** (RN: 614760-53-1)

N^1,N^2-bis(5-methylpyridin-2-yl)oxalamide **(29)** (RN: 349401-68-9)

N^1,N^2-di(pyridin-2-yl)oxalamide **(30)** (RN: 20172-97-8)

N,*N′*-Di-pyridin-3-yl-oxalamide **(31)** (RN: 39642-61-0)

N-Pyridin-2-yl-*N′*-p-tolyl-oxalamide **(32)** (RN: 301344-55-8)

N-Pyridin-2-yl-*N′*-m-tolyl-oxalamide **(33)**

N-Pyridin-3-yl-*N′*-pyridin-2-yl-oxalamide **(34)** (RN: 1796089-38-7)

N-(1-Oxy-pyridin-2-yl)-*N′*-*m*-tolyl-oxalamide **(35)**

N-(1-Oxy-pyridin-2-yl)-*N′*-*p*-tolyl-oxalamide **(36)**

N-(1-Oxy-pyridin-3-yl)-*N′*-*m*-tolyl-oxalamide (**37**)

- **Starting materials and intermediates used in synthesis:**

Methyl 2-((6-methylpyridin-2-yl)amino)-2-oxoacetate (**S1**) (RN: 1566684-50-1)

Methyl 2-((2-methoxyphenyl)amino)-2-oxoacetate (**S2**) (RN: 113449-16-4)

Methyl 2-((3-methoxyphenyl)amino)-2-oxoacetate (**S3**) (RN: 103448-86-8)

Methyl 2-((4-methoxyphenyl)amino)-2-oxoacetate (**S4**) (RN: 24439-54-1)

- Additional compounds from SAR:

2-oxo-2-(o-tolylamino)acetic acid (**S5**) (RN: 406190-09-8)

2-(((1R,2S)-2-hydroxy-2,3-dihydro-1*H*-inden-1-yl)amino)-2-oxoacetic acid (**S6**) (RN: 1849465-46-8)

Methyl 2-(*tert*-butylamino)-2-oxoacetate (**S7**) (RN: 1450740-20-1)

2-(benzylamino)-2-oxoacetic acid (**S8**) (RN: 6345-08-0)

Methyl 2-(((1S,2R)-1-hydroxy-1-phenylpropan-2-yl)amino)-2-oxoacetate (**S9**) (RN: 1218912-11-8)

2-((4-fluorophenyl)amino)-2-oxoacetic acid (**S10**) (RN: 69066-43-9)

2-((2-fluorophenyl)amino)-2-oxoacetic acid (**S11)** (RN: 84944-15-0)

2-((2-bromophenyl)amino)-2-oxoacetic acid (**S12**) (RN: 868565-59-7)

2-((3-bromophenyl)amino)-2-oxoacetic acid (**S13**) (RN: 946744-52-1)

2-((2,4-dichlorophenyl)amino)-2-oxoacetic acid (**S14**) (RN: 17772-30-4)

2-((3-hydroxyphenyl)amino)-2-oxoacetic acid (**S15**) (RN: 38188-60-2)

2-((2-hydroxyphenyl)amino)-2-oxoacetic acid (**S16**) (RN: 89942-67-6)

2-((2-methoxyphenyl)amino)-2-oxoacetic acid (**S17**) (RN: 57727-23-8)

2-((4-methoxyphenyl)amino)-2-oxoacetic acid (**S18**) (RN: 41374-62-3)

2-(2-(*tert*-butoxy)-2-oxoacetamido)-5-methylpyridine 1-oxide (**S19**)

tert-butyl 2-((5-methylpyridin-2-yl)amino)-2-oxoacetate (**S20**)

5-bromo-2-(2-(*tert*-butoxy)-2-oxoacetamido)pyridine 1-oxide (**S21**)

2-(2-(*tert*-butoxy)-2-oxoacetamido)-6-(methoxycarbonyl)pyridine 1-oxide (**S22**)

- **A comprehensive discussion of the "many faces" of FAHD1 substrate oxaloacetate (OAA)**

The structural status of oxaloacetate is mainly dependent on the environmental pH value. OAA may add water to the 2-keto group and form a hydrate (only at low pH). The methylene protons in OAA are acidic due to the flanking 1,3-keto-groups. The molecule can tautomerize into enol forms, which may exist on their part in *E*- and *Z*-configuration. Each of the different OAA-forms will show an individual chemical reactivity. FAHD1 is operative in mitochondria where OAA will be fully deprotonated to the di-anion at the prevailing pH of 7.9 in the mitochondrial matrix (Figure S1)[1]. The pH-dependent protonation states of possible OAA-species in aqueous solution can be calculated (https://chemicalize.com/#/calculation). At physiolocical pH the doubly deprotonated OAA^{2-} species predominates. At lower pH, the C^1-carboxylate is more easily deprotonated than the C^4-carboxylate which reflects the binding affinity of OAA^- towards the Mg^{2+} ion in the catalytic cavity of FAHD1.

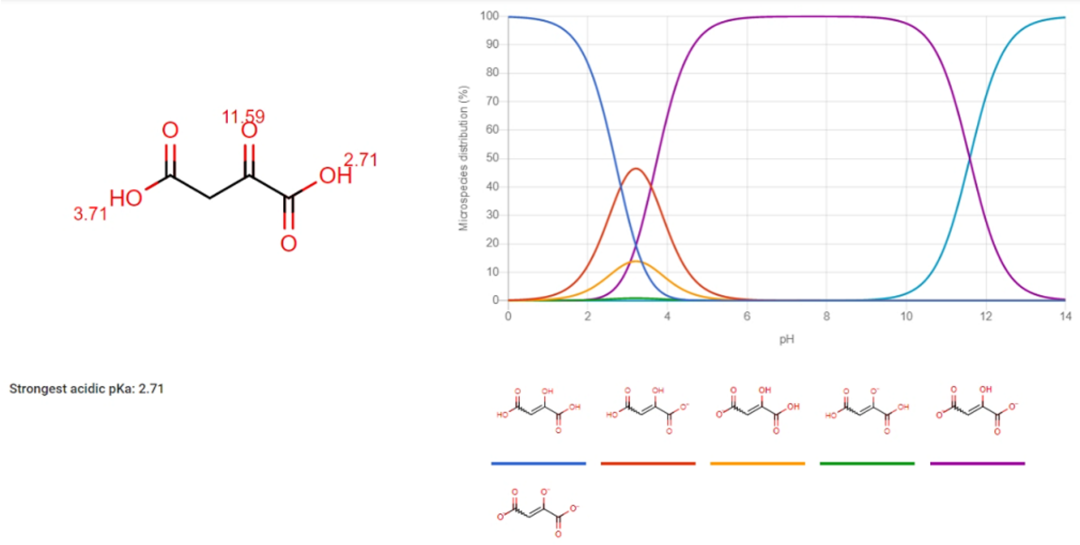

Figure S1. Calculated pH-dependent protonation states of OAA-species.

It has been shown that OAA exists in solution and in solid state in (*Z*)-enol form[2] OAA is unstable in solution and starts slowly to decarboxylate following a first-order kinetics[3]. In organic solvents such as ether OAA is also mainly in the enol form (~90 %)[4]. In a 0.1 M tris buffer at physiological pH value of 7.4 the keto-OAA is predominant (74.3 %), but the enolic species (B′) contributes significantly (17.8 %) while the hydrate (D′) is a minor component (7.8 %). Decreasing the pH to 2.0 leads to an increase of the hydrate (54.9 %) in expense of the keto form[5]. The three titration dissociations constants of OAA have been determined: 2.22 (pKa 1-COOH), 3.89 (pKa 4-COOH), and 12.18 (pKa enolic OH), respectively. The pKa(1) and pKa (2) are slightly higher in the enol form[6,7]. A striking observation is reported for the dissociation of the third proton from enolic oxaloacetic acid. Upon complexation to magnesium the dissociation constant increases more than ten-thousand-fold[6], enabling the deprotonation of the enol function (e.g. by an amino residue). For comparison: without Mg pKa of enol-OH = 12.18 but complexed to Mg pKa = 8.11. Additionally, the enol form is stabilized by the magnesium. Investigations of complexes between magnesium and enolate OAA^{3-} revealed for a 1:1 complex a stability constant of logK = 6.27 which is also ten-thousand-fold greater than any reported logK for Mg^{2+} ions with simple organic acid ligands[8], such as the acid ligands complexing magnesium in the catalytic center of FAHD1.

Another important result was provided by investigation of metal-ion catalyzed decarboxylation of dimethyl-oxaloacetic acid (Figure S2.**A**), a molecule which cannot adopt an enol form. Enolization was found to be not a prerequisite for metal catalyzed decarboxylation and that the first product was the enol form of α-ketoisovaleric acid[9] (**B**). Furthermore, Steinberger and Westheimer demonstrated that decarboxylation of **C** to **D** was not catalyzed by metals[9] Similar to mono anion, thermal decarboxylation of mono ethyl ester (**C**) yields the enol form of ethyl α-ketoisovalerate (**D**)[10]

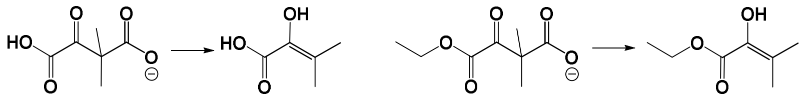

Figure S2. Decarboxylation of dimethyl-oxaloacetic acid mono anion **A** and corresponding mono ethyl ester **C**: primary products are enol forms **B** and **D**

From the Steinberger and Westheimer experiments it could be concluded that the 4-carboxylate group is the source of carbon dioxide. Furthermore, UV/Vis spectroscopy revealed that when OAA is exposed to divalent metal ions a rapid, strong increase of absorption is recorded, at a wavelength where α-keto acids have little absorption. The authors assigned the new absorbing species to an enolic metal complex of structure **B** shown in Figure S2. The presence of an enol was verified by iodine titration experiments. But was chelated **B** the structure from which carbon dioxide originates? Two possible Mg^{2+}- OAA^{2-} chelates are depicted in Figure 4.

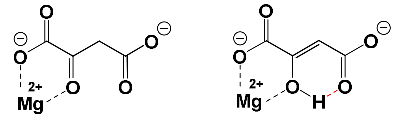

Figure S3. Metal chelated oxaloacetate di-anion: 2-keto form (**1**), enol form (**2**); hydrogen bonding: dashed red bond.

Considering that 3,3-dimethyl oxaloacetic acid (see Figure S2, **A**) cannot form an enolic complex of type **2** (Figure S3, **2**) but nevertheless decarboxylates metal catalyzed. This suggests complex **1** as the most likely active species in the metal catalyzed decarboxylation of OAA. Additionally, complex **1** has rotational freedom around two C-C bonds (C^2-C^3 and C^3-C^4). But the enol-complex **2** is a flat molecule with resonance stabilized intramolecular hydrogen bond between enolic hydrogen and oxygen of the 4-carboxylate (red bond in Figure S3, **2**). The C^3-C^4 σ-bond has no orbital overlap with the π-bond present in the enol. Decarboxylation of complex **2** would produce a non-stabilized sp^2-centered carbanion. Gelles and Hay provided esperimental evidence that ketonic chelate compounds are a kinetically active species in decarboxylation. They demonstrated in spectrophotometric studies also the presence of enolic chelates which did not decarboxylate[11]. Therefore complex **2** is unlikely the source of carbon dioxide. But the conformationally flexible oxaloacetate in complex **1** can circumvent the obstacle of missing orbital overlap through rotational freedom around the C^2-C^3 bond such positioning the C^3-C^4 σ-bond in parallel alignment with the π*-orbital of the 2-keto group (see Figure 1 in manuscript). The demanded conformation for decarboxylation of complex **1** is adjusted by FAHD1.

Summary and working hypothesis: OAA binds initially in the enol-form to the Mg^{2+} co-factor followed by slow ketonization of the substrate. The necessary deprotonation of the enol is supported by the K123 residue of FAHD1. The driving force is the highly increased acidity of the Mg^{2+} bound enolic hydroxyle group. The conformation of the such formed 2-keto OOA Mg^{2+} complex gets under control of FAHD1 residues R106-Q109 which finally ensures parallel alignment of the orbitals involved in C-C bond cleavage.

- **Additional compounds investigated for FAHD1 inhibition (Figure S4)**

ID	Structure	CAS RN	IC_{50} (µM)
	***N*-alkyl, *N*-arylalkyl**		
**S5**	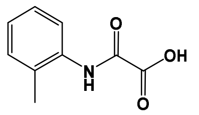	406190-09-8	**108**
**S6**	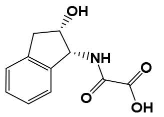	1849465-46-8	**250**
**S7**	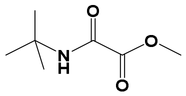	1450740-20-1	**inactive**
**S8**	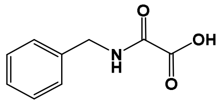	6345-08-0	**552**
**S9**	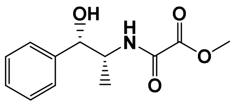	1218912-11-8	**inactive**

ID	Structure	CAS RN	IC_{50} (µM)
	**Hydroxy and methoxy substitution**		
**S15**	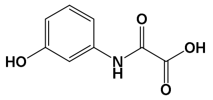	38188-60-2	**35**
**S16**	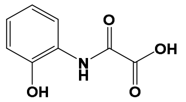	89942-67-6	**212**
**S17**	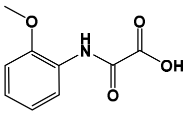	57727-23-8	**121**
**S18**	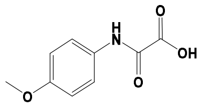	41374-62-3	**155**

ID	Structure	CAS RN	IC_{50} (µM)
	**Halogen substitution**		
**S10**	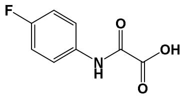	69066-43-9	**140**
**S11**	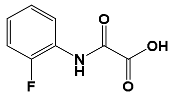	84944-15-0	**inactive**
**S12**	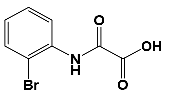	868565-59-7	**34**
**S13**	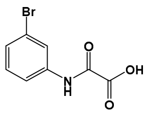	946744-52-1	**75**
**S14**	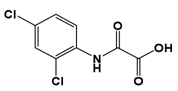	17772-30-4	**134**

ID	Structure	CAS RN	IC_{50} (µM)
	***tert.* Butyl ester derivatives**		
**S19**	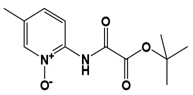	none	**3**
**S20**	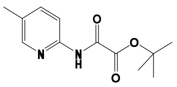	none	**5**
**S21**	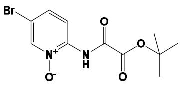	none	**13**
**S22**	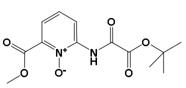	none	**14**

- **Analytical data of synthesized compounds**

NMR assignment, HRMS and melting points

2-(2-methoxy-2-oxoacetamido)benzoic acid (1a)

(RN: 174575-83-8); Mp: 184-185°C; HRMS: calc. [$C_{10}H_9NO_5$]: 223.05; found: 246.0365 $[M+Na]^+$.

^{1}H NMR (600.25 MHz, DMSO-d$_6$, 25°C): δ = 3.87 (s, 3H, OC*H*$_3$), 7.23-7.28 (m[dt], 1H, *H*-4 aniline), 7.64-7.69 (m[dt], 1H, *H*-5 aniline), 8.05 (dd, ^3J = 7.9, ^4J = 1.6, 1H, *H*-3 aniline), 8.59 (dd, ^3J = 8.5, ^4J = 0.9, 1H, *H*-6 aniline), 12.58 (s, 1H, N*H*), 13.84 (br, s, 1H, COO*H*); ^{13}C{^{1}H}NMR (150.93 MHz, DMSO-d$_6$, 25°C): δ = 53.62 (OCH$_3$), 117.03 (*C*-2 aniline), 119.67 (*C*-6 aniline), 124.08 (*C*-4 aniline), 131.44 (*C*-3 aniline), 134.41 (*C*-5 aniline), 139.29 (*C*-1 aniline), 154.22 (NHCO), 160.53 (*C*OOMe), 169.24 (*C*OOH).

2-(carboxyformamido)benzoic acid (1b)

(RN: 5651-01-4); Mp:206-208 ^{0}C; HRMS: calc. [$C_9H_7NO_5$]: 209.0324; found: 210.034 [M+H]+.

^{1}H NMR (600.25 MHz, DMSO-d$_6$, 25 °C): δ = 3.0-5.0 (NHCOCOO*H* in exchange with water), 7.24 (t[ddd], $^3J_1$ = $^3J_2$ = 7.7, ^4J = 1.1, 1H, *H*-5), 7.66 (t[ddd], $^3J_1$ = 7.2, $^3J_2$ = 8.0, ^4J = 1.6, 1H, *H*-4), 8.04 (dd, $^3J_2$ = 7.9, ^4J = 1.6, *H*-6), 8.62 (dd, $^3J_2$ = 8.3, ^4J = 0.8, 1H, *H*-3), 12.50 (s, sh, 1H, N*H*COCOOH), 12.8-15.0 (br, 1H, 1-COO*H*). ^{13}C{^{1}H}NMR (150.93 MHz, DMSO-d$_6$, 25 °C): δ = 117.04 (C_q-1), 119.61 (*C*-3), 123.94 (*C*-5), 131.46 (*C*-6), 134.36 (*C*-4), 139.43 (C_q-2), 156.02 (NH*C*OCOOH), 161.45 (NHCO*C*OOH), 169.08 (1-*C*OOH).

2-(2-(2-methoxy-2-oxoacetamido)benzamido)benzoic acid (1d)

(RN: none); Mp: 200-202 °C (MeOH); HRMS: calc. [$C_{17}H_{14}N_2O_6$]: 342.09; found: 343.0925 [M+H]+.

^{1}H NMR (600.25 MHz, DMSO-d$_6$, 25°C): δ = 3.85 (s, 3H, OC*H*$_3$), 7.27 (dt, ^3J = 7.6, ^4J = 1.1, 1H, *H*-5 aryl B), 7.38 (dt, ^3J = 7.6, ^4J = 1.1, 1H, *H*-5 aryl A), 7.66 (m[t], 1H, *H*-4 aryl A), 7.69 (m[t], 1H, *H*-4 aryl B), 7.94 (dd, ^3J = 7.9, ^4J = 1.3, 1H, *H*-6 aryl A), 8.05 (dd, ^3J = 8.0, ^4J = 1.6, 1H, *H*-6 aryl B), 8.44 (dd, ^3J = 8.4, ^4J = 0.7, 1H, *H*-3 aryl B), 8.54 (dd, ^3J = 8.4, ^4J = 0.8, 1H, *H*-3 aryl A), 12.06 (s, 1H, N*H*CO), 12.14 (s, 1H, N*H*COCOOMe), (COO*H* in exchange with water in DMSO). ^{13}C{1H}NMR (150.93 MHz, DMSO-d$_6$, 25°C): δ = 53.57 (OCH$_3$), 117.89 (*C*-1 aryl B), 120.85 (*C*-3 aryl B), 121.34 (*C*-3 aryl A), 122.83 (*C*-1 aryl A), 123.79 (*C*-5 aryl B), 124.79 (*C*-5 aryl A), 127.96 (*C*-6 aryl A), 131.25 (*C*-6 aryl B), 132.92 (*C*-4 aryl A), 134.18 (*C*-4 aryl B), 137.34 (*C*-2 aryl A), 140.14 (*C*-2 aryl B), 154.27 (NH*C*OCOOMe), 160.62 (*C*OOMe), 166.41 (NH*C*O), 169.72 (*C*OOH); aryl A: aryl bearing the oxalyl head; aryl B: aryl bearing the COOH function.

2-(2-(carboxyformamido)benzamido)benzoic acid (1e)

(RN: none); Mp: 215-217°C; HRMS: calc. [$C_{16}H_{12}N_2O_6$]: 328.07; found: 351.0691$[M+Na]^+$.

^{1}H NMR (600.25 MHz, DMSO-d$_6$, 25°C): δ = 7.27 (d, ^3J = 7.7, 1H, *H*-5 aryl B), 7.37 (d, ^3J = 7.7, 1H, *H*-5 aryl A), 7.67 (m[t], 1H, *H*-4 aryl A), 7.70 (m[t], 1H, *H*-4 aryl B), 7.94 (dd, ^3J = 7.9, ^4J = 1.3, 1H, *H*-6

aryl A), 8.05 (dd, ^3J = 7.9, ^4J = 1.6, 1H, *H*-6 aryl B), 8.51 (d, ^3J = 8.2, 1H, *H*-3 aryl B), 8.53 (d, ^3J = 8.4, 1H, *H*-3 aryl A), 12.06 (s, 1H, N*H*CO), 12.13 (s, 1H, N*H* A). ^{13}C{1H}NMR (150.93 MHz, DMSO-d$_6$, 25°C): δ = 117.92 (*C*-1 aryl B), 120.87 (*C*-3 aryl B), 121.04 (*C*-3 aryl A), 122.55 (*C*-1 aryl A), 123.77 (*C*-5 aryl B), 124.57 (*C*-5 aryl A), 127.92 (*C*-6 aryl A), 131.25 (*C*-6 aryl B), 132.94 (*C*-4 aryl A), 134.18 (*C*-4 aryl B), 137.65 (*C*-2 aryl A), 140.15 (*C*-2 aryl B), 155.90 (NH*C*O B), 161.49 (*C*OOH A), 166.47 (NH*C*O), 169.71 (*C*OOH B); aryl A: aryl bearing the oxalyl part; aryl B: aryl bearing the COOH function.

3-(2-methoxy-2-oxoacetamido)benzoic acid (2a)

(RN: none); Mp: 192-193 °C.

^{1}H NMR (600.25 MHz, DMSO-d$_6$, 25°C): δ = 3.86 (s, 3H, OC*H*$_3$), 7.48 (t, ^3J = 7.9, 1H, *H*-5 aryl), 7.72 (m[d], ^3J = 7.8, 1H, *H*-6 aryl), 7.94 (m[d], ^3J = 8.2, 1H, *H*-4 aryl), 8.41 (t, ^4J = 1.9, 1H, *H*-2 aryl), 10.98 (s, 1H, N*H*); ^{13}C{1H}NMR (150.93 MHz, DMSO-d$_6$, 25°C): δ = 53.27 (OCH$_3$), 121.28 (*C*-2 aryl), 124.71 (*C*-4 aryl), 125.57 (*C*-6 aryl), 129.10 (*C*-5 aryl), 131.44 (*C*-1 aryl), 137.78 (*C*-3 aryl), 155.47 (NH*C*O), 160.86 (*C*OOMe), 167.02 (*C*OOH).

3-(Carboxyformamido)benzoic acid (2b)

(RN: 58465-46-6); Mp: 210-211 ^{0}C; HRMS: calc. [$C_9H_7NO_5$]: 209.0324; found: 232.0216 [M+Na]$^+$.

^{1}H NMR (600.25 MHz, DMSO-d$_6$, 25 °C): δ = 7.47 (t, $^3J_1$ = $^3J_2$ = 8.0, 1H, *H*-5), 7.70 (dt, ^3J = 7.7, $^4J_1$ = $^4J_2$ = 1.0, 1H, *H*-6), 7.95 (ddd, ^3J = 8.0, $^4J_1$ = 0.7, $^4J_2$ = 1.8, 1H, *H*-5), 8.44 (t, $^4J_1$ = $^4J_2$ = 1.7, 1H, *H*-2), 10.91 (s, 1H, N*H*COCOOH), 13.09 (br, COO*H*); ^{13}C{^{1}H}NMR (150.93 MHz, DMSO-d$_6$, 25 °C): δ = 121.13 (*C*-2), 124.59 (*C*-4), 125.38 (*C*-6), 129.02 (*C*-5), 131.39 (*C*-1), 138.01 (*C*-3), 157.11 (NH*C*OCOOH), 161.98 (NHCO*C*OOH), 167.08 (1-*C*OOH)

4-(2-methoxy-2-oxoacetamido)benzoic acid (3a)

(RN: 1086273-37-1); Mp: 251-253°C; HRMS: calc. [$C_{10}H_9NO_5$]: 223.05; found: 224.0561[M+H]$^+$.

^{1}H NMR (600.25 MHz, DMSO-d$_6$, 25°C): δ = 3.86 (s, 3H, OC*H*$_3$), 7.88 (m[d], 2H, *H*-2, *H*-6 aniline), 7.93 (m[d], 2H, *H*-3, *H*-5 aniline), 11.06 (s, 1H, N*H*), 12.84 (br, s, 1H, COO*H*); ^{13}C{1H}NMR (150.93 MHz, DMSO-d6, 25°C): δ = 53.30 (OCH$_3$), 119.90 (*C*-2, *C*-6 aniline), 126.66 (*C*-4 aniline), 130.28 (*C*-3, *C*-5 aniline), 141.51 (*C*-1 aniline), 155.59 (NH*C*O), 160.76 (*C*OOMe), 166.82 (*C*OOH).

4-(carboxyformamido)benzoic acid (3b)

(RN: 14121-56-3); Mp: 87-92°C; HRMS: calc. [$C_9H_7NO_5$]: 209.03; found: 232.0209 [M+Na]$^+$.

^{1}H NMR (600.25 MHz, DMSO-d$_6$, 25°C): δ = 7.88 (m, 2H, *H*-2, *H*-6 aniline), 7.92 (m, 2H, *H*-3, *H*-5 aniline), 10.99 (s, 1H, N*H*), COO*H* in rapid exchange; ^{13}C{^{1}H}NMR (150.93 MHz, DMSO-d$_6$, 25°C): δ = 119.75 (*C*-2, *C*-6 aniline), 126.48 (*C*-4 aniline), 130.27 (*C*-3, *C*-5 aniline), 141.75 (*C*-1 aniline), 157.38 (NH*C*O), 161.87 (*C*OOH), 166.85 (Ar-*C*OOH).

Methyl 2-((2-nitrophenyl)amino)-2-oxoacetate (4a)

(RN: none); Mp: 162-167°C; HRMS: calc. [$C_9H_8N_2O_5$]: 224.04; found: 247.0324 $[M+Na]^+$.

^{1}H NMR (600.25 MHz, DMSO-d$_6$, 25°C): δ = 3.89 (s, 3H, OC*H*$_3$), 7.45 (dt, ^3J = 7.9, ^4J = 1.2, 1H, *H*-4 aniline), 7.80 (dt, ^3J = 7.9, ^4J = 1.4, 1H, *H*-5 aniline), 8.06 (dd, ^3J = 8.2, ^4J = 1.2, 1H, *H*-6 aniline), 8.12 (dd, ^3J = 8.2, ^4J = 1.4, 1H, *H*-3 aniline), 11.39 (s, 1H, N*H*); ^{13}C{^{1}H}NMR (150.93 MHz, DMSO-d$_6$, 25°C): δ = 53.67 (OCH$_3$), 124.47 (*C*-6 aniline), 125.51 (*C*-3 aniline), 126.08 (*C*-4 aniline), 130.79 (*C*-1 aniline), 134.95 (*C*-5 aniline), 140.76 (*C*-2 aniline), 154.90 (NHCO), 160.24 (*C*OOMe).

2-((2-nitrophenyl)amino)-2-oxoacetic acid (4b)

(RN: 77901-51-0); Mp: 148-151°C; HRMS: calc. [$C_9H_6N_2O_5$]: 210.03; found: 211.0394 $[M+H]^+$.

^{1}H NMR (600.25 MHz, DMSO-d$_6$, 25°C): δ = 7.42 (t, ^3J = 7.7, 1H, *H*-4 aniline), 7.80 (dt, ^3J = 7.6, 1H, *H*-5 aniline), 8.13 (d, ^3J = 8.2, 1H, *H*-6 aniline), 8.17 (d, ^3J = 8.2, 1H, *H*-3 aniline), 11.40 (s, 1H, N*H*), COO*H* in rapid exchange; ^{13}C{^{1}H}NMR (150.93 MHz, DMSO-d$_6$, 25°C): δ = 123.79 (*C*-3 aniline), 125.56 (*C*-6 aniline), 125.62 (*C*-4 aniline), 131.40 (*C*-1 aniline), 135.09 (*C*-5 aniline), 140.07 (*C*-2 aniline), 156.63 (NH*C*O), 161.19 (*C*OOH).

Methyl 2-((3-nitrophenyl)amino)-2-oxoacetate (5a)

(RN: 1237427-49-4); Mp: 169-172°C; HRMS: calc. [$C_9H_8N_2O_5$]: 224.04; found: 247.0326 $[M+Na]^+$

^{1}H NMR (600.25 MHz, DMSO-d$_6$, 25°C): δ = 3.87 (s, 3H, OC*H*$_3$), 7.65 (t, ^3J = 8.1, 1H, *H*-5 aniline), 7.99 (m, 1H, *H*-4 aniline), 8.16 (m, 1H, *H*-6 aniline), 8.74 (m, 1H, *H*-2 aniline), 11.27 (s, 1H, N*H*); ^{13}C{^{1}H}NMR (150.93 MHz, DMSO-d$_6$, 25°C): δ = 53.39 (OCH$_3$), 114.71 (*C*-2 aniline), 119.24 (*C*-4 aniline), 126.49 (*C*-6 aniline), 130.24 (*C*-5 aniline), 138.74 (*C*-1 aniline), 147.88 (*C*-3 aniline), 155.56 (NHCO), 160.43 (*C*OOMe).

2-((3-nitrophenyl)amino)-2-oxoacetic acid (5b)

(RN: 6274-26-6); Mp: 155-162°C; HRMS: calc. [$C_9H_6N_2O_5$]: 210.03; found: 211.0394 $[M+H]^+$.

^{1}H NMR (600.25 MHz, DMSO-d$_6$, 25°C): δ = 7.66 (t, ^3J = 8.1, 1H, *H*-5 aniline), 8.00 (m, 1H, *H*-4 aniline), 8.17 (m, 1H, *H*-6 aniline), 8.78 (m, 1H, *H*-2 aniline), 11.20 (s, 1H, N*H*), 14.41 (br, s, 1H, COO*H*); ^{13}C{^{1}H}NMR (150.93 MHz, DMSO-d$_6$, 25°C): δ = 114.54 (*C*-2 aniline), 119.06 (*C*-4 aniline), 126.38 (*C*-6 aniline), 130.21 (*C*-5 aniline), 138.96 (*C*-1 aniline), 147.91 (*C*-3 aniline), 157.34 (NHCO), 161.55 (*C*OOH).

Methyl 2-((4-nitrophenyl)amino)-2-oxoacetate (6a)

(RN: 82633-22-5); Mp: 232-234°C; HRMS: calc. [$C_9H_8N_2O_5$]: 224.04; found: 247.0322$[M+Na]^+$

^{1}H NMR (600.25 MHz, DMSO-d$_6$, 25°C): δ = 3.87 (s, 3H, OC*H*$_3$), 8.03 (m, 2H, *H*-2, *H*-6 aniline), 8.25 (m, 2H, *H*-3, *H*-5 aniline), 11.33 (s, 1H, N*H*); ^{13}C{^{1}H}NMR (150.93 MHz, DMSO-d$_6$, 25°C): δ = 53.41 (OCH$_3$), 120.41 (*C*-2, *C*-6 aniline), 124.77 (*C*-3, *C*-5 aniline), 143.33 (*C*-4 aniline), 143.66 (*C*-1 aniline), 155.75 (NHCO), 160.37 (*C*OOMe).

2-((4-nitrophenyl)amino)-2-oxoacetic acid (6b)

(RN: 103-94-6); Mp: 205-212°C; HRMS: calc. [$C_9H_6N_2O_5$]: 210.03; found: 211.0394 $[M+H]^+$.

^{1}H NMR (600.25 MHz, DMSO-d$_6$, 25°C): δ = 8.05 (d, ^3J = 9.2, 2H, *H*-2, *H*-6 aniline), 8.26 (d, ^3J = 9.2, 2H, *H*-3, *H*-5 aniline), 11.26 (s, 1H, N*H*), 14.49 (br, s, 1H, COO*H*); ^{13}C{^{1}H}NMR (150.93 MHz, DMSO-d$_6$, 25°C): δ = 120.24 (*C*-2, *C*-6 aniline), 124.78 (*C*-3, *C*-5 aniline), 143.20 (*C*-4 aniline), 143.90 (*C*-1 aniline), 157.61 (NHCO), 161.53 (COOH).

Methyl 2-(naphthalen-2-ylamino)-2-oxoacetate (7a)

(RN: 20518-40-5) Mp: 133-136°C; HRMS: calc. [$C_{13}H_{11}NO_3$]: 229.0739; found: 252.0642 $[M+Na]^+$.

^{1}H NMR (600.25 MHz, CDCl$_3$, 25 °C): δ = 3.99 (s, 3H, OC*H*$_3$), 7.45 (t, ^3J = 7.5, 1H, *H*-6 naphthyl), 7.49 (t, ^3J = 7.5, 1H, *H*-7 naphthyl), 7.57 (dd, ^3J = 8.8, ^4J = 2.1, 1H, *H*-3 naphthyl), 7.80 (d, ^3J = 8.0, 1H, *H*-4 naphthyl), 7.84 (d, ^3J = 8.5, 2H, *H*-5, *H*-8 naphthyl), 8.33 (d, ^4J = 1.8, 1H, *H*-1 naphthyl), 9.04 (s, 1H, N*H*); ^{13}CNMR (150.93 MHz, CDCl$_3$, 25 °C): δ = 54.25 (OCH$_3$),117.33 (*C*-1 naphthyl), 119.37 (*C*-3 naphthyl), 125.84 (*C*-6 naphthyl), 126.95 (*C*-7 naphthyl), 127.77 (*C*-4 naphthyl), 128.01 (*C*-8 naphthyl), 129.27 (*C*-5 naphthyl), 131.29 (*C*-4a naphthyl), 133.70 (*C*-2 or *C*-8a naphthyl), 133.74 (*C*-2 or *C*-8a naphthyl), 153.81 (NHCO), 161.61 (*C*OOMe).

2-(naphthalen-2-ylamino)-2-oxoacetic acid (7b)

RN: 81682-60-2); Mp: 150-155°C; HRMS: calc. [$C_{12}H_9NO_3$]: 215.06; found: 238.0639 $[M+Na]^+$.

^{1}H NMR (600.25 MHz, DMSO-d$_6$, 25 °C): δ = 7.44 (t, ^3J = 7.5, 1H, *H*-6 naphthyl), 7.49 (t, ^3J = 7.0, 1H *H*-7 naphthyl), 7.81 (dd, ^3J = 8.8, *J* = 1.9, 1H, *H*-3 naphthyl), 7.85 (dd[t], ^3J = 7,7, 2H, *H*-5, *H*-8 naphthyl), 7.88 (d, ^3J = 8.8, 1H, *H*-4 naphthyl), 8.43 (s, 1H, *H*-1 naphthyl), 10.90 (s, 1H, N*H*), 14.29 (br, s, 1H, COO*H*); ^{13}C{^{1}H}NMR (150.93 MHz, DMSO-d$_6$, 25 °C): δ = 116.87 (*C*-1 naphthyl), 120.56 (*C*-3 naphthyl), 125.25 (*C*-6 naphthyl), 126.57 (*C*-7 naphthyl), 127.52 (*C*-5 naphthyl), 127.59 (*C*-8 naphthyl), 128.40 (*C*-4 naphthyl), 130.34 (*C*-4a naphthyl), 133.13 (*C*-8a naphthyl), 135.38 (*C*-2 naphthyl), 157.32 (NHCO), 162.21 (*C*OOH).

Methyl 2-(naphthalen-1-ylamino)-2-oxoacetate (8a)

(RN: 254751-07-0); Mp: 75-76°C.

^{1}H NMR (600.25 MHz, DMSO-d$_6$, 25°C): δ = 3.87 (s, 3H, OC*H*$_3$), 7.48-7.55 (m, 1H, *), 7.83-7.87 (m, 2H, *), 7.91-7.95 (m, 4H, *), 10.90 (s, 1H, N*H*); ^{13}C{1H}NMR (150.93 MHz, DMSO-d$_6$, 25°C): δ = 54.19 (OCH$_3$), 123.42 (*), 124.33 (*), 126.41 (*), 127.29 (*), 127.35 (*), 128.12 (*), 129.02 (*), 129.15 (*), 132.44 (*), 134.46 (*), 157.67 (NHCO), 161.82 (*C*OOMe); *Assignment not possible due to mixture of conformers.

2-(naphthalen-1-ylamino)-2-oxoacetic acid (8b)

(RN: 21660-76-4); Mp: 176-177°C.

^{1}H NMR (600.25 MHz, DMSO-d$_6$, 25°C): δ = 7.48-7.56 (m, 4H, *), 7.81-7.86 (m, 2H, *), 7.91-7.95 (m, 1H, *) 10.73 (s, 1H, N*H*), COO*H* in rapid exchange; ^{13}C{1H}NMR (150.93 MHz, DMSO-d$_6$, 25°C): δ = 123.27 (*), 123.94 (*), 126.44 (*), 127.26 (*), 127.31 (*), 127.83 (*), 129.01 (*), 129.05 (C_q*), 132.72 (C_q*),

134.46 (C_q*), 159.46 (NH*C*O), 163.06 (*C*OOH); *Assignment not possible due to mixture of conformers.

Methyl 2-oxo-2-(pyridin-3-ylamino)acetate (9a)

(RN: 480452-68-4); Mp: 118-121 ^{0}C; HRMS: calc. [$C_8H_8N_2O_3$]: 180.0535; found: 203.0427 [M+Na]$^+$.

^{1}H NMR (600.25 MHz, CDCl$_3$, 25 °C): δ = 3.92 (s, 3H, NHCOCOOC*H*$_3$), 7.28 (dd, $^3J_1$ = 8.3, $^3J_2$ = 4.8, 1H, *H*-5), 8.20 (ddd, ^3J = 8.3, $^4J_1$ = 2.2, $^4J_2$ = 1.2, 1H, *H*-4), 8.38 (dd, ^3J = 4.8, ^4J = 1.2, 1H, *H*-6), 8.67 (d, ^4J = 2.4, 1H, *H*-2), 9.04 (s, br, 1H, N*H*COCOOCH$_3$); ^{13}C{^{1}H}NMR (150.93 MHz, CDCl$_3$, 25 °C): δ = 54.39 (NHCOCOO*C*H$_3$), 123.98 (*C*-5), 127.22 (*C*-4), 133.30 (C_q-3), 141.49 (*C*-2), 146.72 (*C*-6), 154.29 (NH*C*OCOOCH$_3$), 161.03 (NHCO*C*OOCH$_3$).

3-(2-Methoxy-2-oxoacetamido)pyridine 1-oxide (11a)

Mp: 184-191 ^{0}C; HRMS: calc. [$C_8H_8N_2O_4$]: 196.0484; found: 219.0376 [M+H]$^+$.

^{1}H NMR (600.25 MHz, DMSO-d$_6$, 25 °C): δ = 3.86 (s, 3H, NHCOCOOC*H*$_3$), 7.41 (dd, $^3J_1$ = 8.5, $^3J_2$ = 6.5, 1H, *H*-5), 7.72 (ddd, ^3J = 8.5, $^4J_1$ = 1.7, $^4J_2$ = 0.7, 1H, *H*-4), 8.04 (ddd, ^3J =6.5, $^4J_1$ = 1.7, $^4J_2$ = 0.7, 1H, *H*-6), 8.73 (dd[t], $^4J_1$ = 1.7, $^4J_2$ = 1.7, 1H, *H*-2), 11.21 (s, sh, 1H, N*H*COCOOCH$_3$); ^{13}C{^{1}H}NMR (150.93 MHz, DMSO-d$_6$, 25 °C): δ = 53.50 (NHCOCOO*C*H$_3$),117.04 (*C*-4), 126.31 (*C*-5), 131.12 (*C*-2), 134.98 (*C*-6), 137.07 (C_q-3), 155.57 (NH*C*OCOOCH$_3$), 160.09 (NHCO*C*OOCH$_3$).

3-(Carboxyformamido)pyridine 1-oxide (11b)

Mp: 236 ^{0}C; HRMS: calc. [$C_7H_6N_2O_4$]: 182.0328; found: 183.0400 [M+H]$^+$.

^{1}H NMR (600.25 MHz, DMSO-d$_6$, 25 °C): δ= 7.41 (dd, $^3J_1$ = 8.5, $^3J_2$ = 6.5, 1H, *H*-5), 7.74 (ddd, ^3J = 8.5, $^4J_1$ = 1.6, $^4J_2$ = 0.6, 1H, *H*-4), 8.04 (ddd, ^3J = 6.5, $^4J_1$ = 1.5, $^4J_2$ = 0.7, 1H, *H*-6), 8.76 (m[t], br, 2x ^4J = 1.6, 1H, *H*-2), 11.13 (s, sh, 1H, N*H*COCOOH), 14.6 (s, vbr, 1H, NHCOCOO*H*); ^{13}C{^{1}H}NMR (150.93 MHz, DMSO-d$_6$, 25 °C): δ = 117.13 (*C*-4), 126.31 (*C*-5), 130.98 (*C*-2), 134.82 (*C*-6), 137.27 (C_q-3), 157.39 (NH*C*OCOOH), 161.21 (NHCO*C*OOH).

Methyl 2-oxo-2-(pyridin-2-ylamino)acetate (10a)

(RN: 54166-60-8); Mp: 103-104 ^{0}C.

^{1}H NMR (600.25 MHz, CDCl$_3$, 25 °C): d = ^3J = 8.4, ^4J = 1.8, 1H, *H*-4), 3.98 (s, 3H, NHCOCOOC*H*$_3$), 7.13 (ddd, $^3J_1$ = 4.9, $^3J_2$ = 7.4, ^4J = 0.8, 1H, *H*-5), 7.76 (ddd, $^3J_1$ = 7.5, $^3J_2$ = 7.8, ^4J = 1.8, 1H, *H*-4), 8.24 (d, br, ^3J = 8.3, 1H, *H*-3), 8.35 (d, br, ^3J = 4.8, 1H, *H*-6), 9.41 (s, br, 1H, N*H*COCOOCH$_3$); ^{13}C{^{1}H}NMR (150.93 MHz, CDCl$_3$, 25 °C): d = 54.26 (NHCOCOO*C*H$_3$), 114.40 (*C*-3), 121.17 (*C*-5), 138.73 (*C*-4), 148.51 (*C*-6), 149.93 (C_q-2), 154.10 (NH*C*OCOOCH$_3$), 160.78 (NHCO*C*OOCH$_3$).

2-(2-Methoxy-2-oxoacetamido)pyridine 1-oxide (12a)

Mp: 209-210 ^{0}C;

^{1}H NMR (600.25 MHz, DMSO-d$_6$, 25 °C): δ = 3.89 (NHCOCOOC*H*$_3$), 7.26 (ddd, $^3J_1$ = $^3J_2$ = 7.5, ^4J = 1.8, 1H, *H*-5), 7.52 (ddd, $^3J_1$ = $^3J_2$ = 7.5, ^4J = 1.4, 1H, *H*-4), 8.27 (dd, ^3J = 8.3, ^4J = 1.8, 1H, *H*-3), 8.47 (m[dd], ^3J = 6.5, ^4J = 1.3, 1H, *H*-6), 11.13 (s, br, 1H, N*H*COCOOCH$_3$); ^{13}C{^{1}H}NMR (150.93 MHz, DMSO-d$_6$, 25 °C): δ = 53.81 (NHCOCOO*C*H$_3$), 114.25 (*C*-3), 120.74 (*C*-5), 127.81 (*C*-4), 137.57 (*C*-6), 142.29 (C_q-2), 154.43 (NH*C*OCOOCH$_3$), 159.47 (NHCO*C*OOCH$_3$).

2-(Carboxyformamido)pyridine 1-oxide (12b)

(RN: none); Mp: 198 ⁰C (sharp, dec.); HRMS: calc. [$C_7H_6N_2O_4$]: 182.0328; found: 183.0400 $[M+H]^+$.

^{1}H NMR (600.25 MHz, DMSO-d$_6$, 25 °C): δ = 7.24 (ddd[t], 1H, $^3J_1$ = 8.1, $^3J_1$ = 6.5, ^4J = 1.7, 1H, *H*-5), 7.50 (ddd[t], 1H, $^3J_1$ = 8.0, $^3J_1$ = 8.0, ^4J = 1.1, 1H, *H*-4), 8.27 (dd, ^3J = 8.3, ^4J = 1.7, 1H, *H*-3), 8.46 (dd, ^3J = 6.5, ^4J = 0.7, 1H, *H*-6), 11.15 (s, vbr, 1H, N*H*COCOOH), (NHCOCOO*H* in exchange with H_2O-DMSO-d$_6$); ^{13}C{^{1}H}NMR (150.93 MHz, DMSO-d$_6$, 25 °C): δ = 114.03 (*C*-3), 120.57 (*C*-5), 127.81 (*C*-4), 137.57 (*C*-6), 142.46 (*C$_q$*-2), 155.94 (NHCOCOOH), 160.45 (NHCOCOOH).

Methyl 2-oxo-2-(quinolin-3-ylamino)acetate (13a)

(RN: 1710262-07-9); Mp: 181-182 ⁰C (ethyl acetate); HRMS: calc. [C12H10N2O3]: 230.0691; found: 231.0764 [M+H]+.

^{1}H NMR (600.25 MHz, DMSO-d$_6$, 25 °C): δ = 3.89 (s, 3H, NHCOCOOC*H*$_3$), 7.59 (ddd, $^3J_1$ = 8.0, $^3J_2$ = 6.9, ^4J = 1.1, 1H, *H*-6), 7.69 (ddd, $^3J_1$ = 8.3, $^3J_2$ = 6.7, ^4J = 1.1, 1H, *H*-7), 7.96, 7.98 (2xd [t] , br, 2H, *H*-5, *H*-8), 8.78 (d, ^4J = 2.4, 1H, *H*-4), 9.14 (d, ^4J = 2.4, 1H, *H*-2), 11.29 (s, br, 1H, N*H*COCOOCH$_3$); ^{13}C{^{1}H}NMR (150.93 MHz, DMSO-d$_6$, 25 °C): δ = 53.38 (NHCOCOOCH$_3$), 124.17 (C-4), 127.24 (C-6), 127.46 (*C*$_q$-4a), 128.00 (*C*-5), 128.63 (*C*-8), 128.64 (*C*-7), 131.36 (*C*$_q$-3), 144.81 (*C*$_q$-8a), 145.25 (*C*-2), 155.82 (NHCOCOOCH$_3$), 160.60 (NHCOCOOCH$_3$).

Methyl 2-oxo-2-(quinolin-2-ylamino)acetate (14a)

(RN: none); Mp: 131-134 ⁰C; HRMS: calc. [$C_{12}H_{10}N_2O_3$], 230.0691; found: 253.0584 $[M+Na]^+$.

^{1}H NMR (600.25 MHz, CDCl$_3$, 25 °C): δ = 4.00 (s, 3H, NHCOCOOC*H*$_3$), 7.50 (m[t], br, $^3J_{app}$ = 7.5, 1H, *H*-6), 7.70 (m[t; ddd], br, $^3J_{app}$ = 7.6, 1H, *H*-7), 7.81 (d, br, ^3J = 8.0, 1H, *H*-5), 7.88 (d, ^3J = 8.5, 1H, *H*-8), 8.23 (d, ^3J = 9.0, 1H, *H*-4), 8.42 (d, ^3J = 9.0, 1H, *H*-3), 9.59 (s, br, 1H, N*H*COCOOCH$_3$); ^{13}C{^{1}H}NMR (150.93 MHz, CDCl$_3$, 25 °C): δ = 54.33 (NHCOCOOCH$_3$), 113.90 (*C*-3), 126.05 (*C*-6), 126.88 (*C*$_q$-4a), 127.75 (*C*-8), 127.95 (*C*-5), 130.48 (*C*-7), 139.13 (*C*-4), 146.80 (*C*$_q$-8a), 149.29 (*C*$_q$-2), 154.43 (NHCOCOOCH$_3$), 160.80 (NHCOCOOCH$_3$).

3-(2-Methoxy-2-oxoacetamido)quinoline 1-oxide (15a)

(RN: none); Mp: 219-220 ⁰C (methanol); HRMS: calc. [$C_{12}H_{10}N_2O_4$]: 246.0641; found: 269.0533 $[M+Na]^+$.

^{1}H NMR (600.25 MHz, DMSO-d$_6$, 25 °C): δ = 3.89 (s, 3H, NHCOCOOC*H*$_3$), 7.69 (m[ddd], 1H, *H*-6), 7.72 (m[ddd], 1H, *H*-7), 8.05 (dd, ^3J = 8.0, ^4J = 1.2, 1H, *H*-5), 8.39 (s, br, 1H, *H*-4), 8.44 (m[d], 1H, *H*-8), 8.97 (s, br, 1H, *H*-2), 11.27 (s, br, 1H, N*H*COCOOCH$_3$); ^{13}C{^{1}H}NMR (150.93 MHz, DMSO-d$_6$, 25 °C): δ = 53.49 (NHCOCOOCH$_3$), 114.39 (*C*-4), 118.67 (*C*-8), 128.64 (*C*-5), 129.32 (*C*-6), 129.35 (*C*-7), 129.55 (*C*$_q$-4a), 129.90 (*C*-2), 132.18 (*C*$_q$-3), 138.30 (*C*$_q$-8a), 155.66 (NHCOCOOCH$_3$), 160.24 (NHCOCOOCH$_3$).

3-(Carboxyformamido)quinoline 1-oxide (15b)

(RN: none); Mp: 236-237 ⁰C (disintegration)

^{1}H NMR (600.25 MHz, DMSO-d$_6$, 25 °C): δ = 7.70 (ddd[t], 1H, *H*-6), 7.73 (ddd[t], 1H, *H*-7), 8.06 (dd, ^3J = 7.9, ^4J = 1.4, 1H, *H*-5), 8.43 (d, ^4J = 1.3, 1H, *H*-4), 8.45 (d, br, ^3J = 8.4, 1H, *H*-8), 9.00 (d, ^4J = 1.5, 1H, *H*-2), 11.23 (s, sh, 1H, N*H*COCOOH), (NHCOCOO*H* in exchange with H_2O in DMSO-d$_6$);

$^{13}C\{^1H\}$NMR (150.93 MHz, DMSO-d$_6$, 25 °C): δ = 114.42 (*C*-4), 118.70 (*C*-8), 128.67 (*C*-5), 129.34 (*C*-6), 129.40 (*C*-7), 129.64 (*C*$_q$-4a), 130.01 (*C*-2), 132.43 (*C*$_q$-3), 138.19 (*C*$_q$-8a), 157.46 (NHCOCOOH), 161.38 (NHCOCOOH).

2-(2-Methoxy-2-oxoacetamido)quinoline 1-oxide (16a)

(RN: none)

^{1}H NMR (600.25 MHz, CDCl$_3$, 25 °C): δ = 4.05 (s, 3H, NHCOCOOC*H*$_3$), 7.64 (m[t], ^3J = 7.4, 1H, *H*-6), 7.84 (m[t], ^3J = 7.7, 1H, *H*-7), 7.88 (d, ^3J = 9.4, 1H, *H*-4), 7.90 (d, ^3J = 8.6, 1H, *H*-5), 8.60 (d, ^3J = 9.22, 1H, *H*-3), 8.69 (d, ^3J = 8.72, 1H, *H*-8),11.60 (s, br, 1H, N*H*COCOOCH$_3$); $^{13}C\{^1H\}$NMR (150.93 MHz, CDCl$_3$, 25 °C): δ = 54.46 (NHCOCOOCH$_3$), 112.90 (*C*-3), 119.14 (*C*-8), 126.59 (*C*$_q$-4a), 127.81 (*C*-6), 128.22 (*C*-4), 128.39 (*C*-5), 131.63 (*C*-7), 139.50 (*C*$_q$-8a), 140.65 (*C*$_q$-2), 154.76 (NHCOCOOCH$_3$), 159.45 (NHCOCOOCH$_3$).

2-(Carboxyformamido)quinoline 1-oxide (16b)

(RN: none); Mp: 119.5-125 ^{0}C; HRMS: calc. [$C_{11}H_8N_2O_4$]: 232.0484; found: 255.0376 $[M+Na]^+$.

^{1}H NMR (600.25 MHz, DMSO-d$_6$, 25 °C): δ = 7.69 (ddd[t], ^3J = 7.8 , 1H, *H*-6), 7.88 (ddd[t], ^3J =7.5, 1H, *H*-7), 8.09 (d, ^3J = 8.3, 1H, *H*-4), 8.11 (d, ^3J = 9.3 , 1H, *H*-5), 8.46 (d, ^3J = 9.1 , 1H, *H*-3), 8.49 (d, ^3J = 8.6 , 1H, *H*-8), 10.8-12.4 (s, br, 1H, N*H*COCOOH), (NHCOCOO*H* in exchange with H_2O in DMSO-d$_6$); $^{13}C\{^1H\}$NMR (150.93 MHz, DMSO-d$_6$, 25 °C): δ = 112.24 (*C*-3), 118.27 (*C*-8), 126.02 (*C*$_q$-4a), 127.44 (*C*-6), 127.48 (*C*-4),

128.83 (*C*-5), 131.44 (*C*-7), 138.75 (*C*$_q$-8a), 140.25 (*C*$_q$-2), 156.41 (NHCOCOOH), 160.52 (NHCOCOOH).

Methyl 4-oxo-4*H*-benzo[d][1,3]oxazine-2-carboxylate (17a)

(RN: 3603-20-1); Mp: 173-175°C; HRMS: calc. [$C_{10}H_7NO_4$]: 205.04; found: 206.0447 $[M+H]^+$.

^{1}H NMR (600.25 MHz, DMSO-d$_6$, 25°C): δ = 3.94 (s, 3H, OC*H*$_3$), 7.77 (dt, ^3J = 7.6, ^4J = 1.0, *H*-5 benzoxazine), 7.83 (dd, ^3J = 8.0, ^4J = 0.7, *H*-8 benzoxazine), 8.02 (dt, ^3J = 7.8, ^4J = 1.4, *H*-7 benzoxazine), 8.20 (dd, ^3J = 7.8, ^4J = 1.2, *H*-5 benzoxazine); $^{13}C\{1H\}$NMR (150.93 MHz, DMSO-d$_6$, 25°C): δ = 53.58 (OCH$_3$), 118.37 (*C*-4a benzoxazine), 128.05 (*C*-8 benzoxazine), 128.27 (*C*-5 benzoxazine), 130.81 (*C*-6 benzoxazine), 137.12 (*C*-7 benzoxazine), 144.68 (*C*-8a benzoxazine), 146.99 (*C*-2 benzoxazine), 158.08 (*C*OOMe), 158.15 (*C*-4 benzoxazine).

Methyl 2-((2-carbamoylphenyl)amino)-2-oxoacetate (18a)

(RN: 69065-88-9); Mp: 164-166°C.

HRMS: calc. [$C_{10}H_{10}N_2O_4$]: 222.06; found: 223.0614 $[M+H]^+$.

^{1}H NMR (600.25 MHz, DMSO-d$_6$, 25°C): δ = 3.85 (s, 3H, OC*H*$_3$), 7.24 (dt, ^3J = 7.7, ^4J = 1.1, 1H, *H*-4 aryl), 7.57 (dt, ^3J = 7.8, ^4J = 1.3, 1H, *H*-5 aryl), 7.82 (s, 1H, N*H*$_2$), 7.87 (dd, ^3J = 8.0, ^4J = 1.3, 1H, *H*-3 aryl), 8.36 (s, 1H, N*H*), 8.52 (dd, ^3J = 8.4, ^4J = 0.9, 1H, *H*-6 aryl), 13.04 (s, 1H, N*H*$_2$); $^{13}C\{1H\}$NMR (150.93 MHz, DMSO-d$_6$, 25°C): δ = 53.66 (OCH$_3$), 120.20 (*C*-6 aryl), 120.47 (*C*-2 aryl), 124.07 (*C*-4 aryl), 128.91 (*C*-3 aryl), 132.67 (*C*-5 aryl), 138.14 (*C*-1 aryl), 154.24 (NHCO), 160.71 (*C*OOMe), 170.51 (*C*OONH$_2$).

Methyl 4-oxo-1,4-dihydroquinazoline-2-carboxylate (19a)

(RN: 63569-82-4); Mp: 201-204°C; Lit.: 202-203°C; HRMS: calc. [$C_{10}H_8N_2O_3$]: 204.05; found: 205.0584 $[M+H]^+$.

^{1}H NMR (600.25 MHz, $CDCl_3$, 25°C): δ = 3.95 (s, 3H, OC*H*$_3$), 7.22 (dt, ^3J = 7.7, ^4J = 1.0, *H*-6 quinazoline), 7.59 (m[d], 1H, *H*-5 quinazoline), 7.60 (m[dt], 1H, *H*-7 quinazoline), 8.39 (dd, ^3J = 8.9, ^4J = 1.0, 1H, *H*-8 quinazoline), 9.32 (br, s, 1H, N*H*); ^{13}C{^{1}H}NMR (150.93 MHz, $CDCl_3$, 25°C): δ = 54.54 (OCH_3), 103.18 (*C*-4a quinazoline), 115.68 (*C*-8a quinazoline), 121.14 (*C*-8 quinazoline), 125.67 (*C*-6 quinazoline), 132.75 (*C*-5 quinazoline), 134.48 (*C*-7 quinazoline), 138.71 (*C*-4 quinazoline), 154.04 (*C*-2 quinazoline), 160.44 (*C*OOMe).

4-oxo-1,4-dihydroquinazoline-2-carboxylic acid (19b)

(RN: 29113-34-6); Mp: 213-215°C; HRMS: calc. [$C_9H_6N_2O_3$]: 190.04; found: 213.0379.

^{1}H NMR (600.25 MHz, DMSO-d$_6$, 25°C): δ = 7.43 (dt, ^3J = 7.7, ^4J = 0.9, 1H, *H*-6 quinazoline), 7.64 (d, ^3J = 8.2, 1H, *H*-8 quinazoline), 7.74 (dt, ^3J = 7.9, ^4J = 1.5, 1H, *H*-7 quinazoline), 7.87 (dd, ^3J = 7.9, ^4J = 1.3, 1H, *H*-5 quinazoline), 10.89 (s, 1H, N*H*), COO*H* in rapid exchange; ^{13}C{1H}NMR (150.93 MHz, DMSO-d$_6$, 25°C): δ = 108.23 (*C*-4a quinazoline), 116.57 (*C*-4 quinazoline), 125.89 (*C*-8 quinazoline), 126.85 (*C*-6 quinazoline), 133.29 (*C*-5 quinazoline), 134.03 (*C*-7 quinazoline), 138.93 (*C*-8a quinazoline), 157.30 (*C*OOH), 161.46 (*C*-2 quinazoline).

Methyl 2-((2-methoxyphenyl)amino)-2-thioxoacetate (20a)

(RN: none); Mp: 94-96°C; HRMS: calc. [$C_{10}H_{11}NO_3S$]: 225.05; found: 248.0353 $[M+Na]^+$.

^{1}H NMR (600.25 MHz, $CDCl_3$, 25°C): δ = 3.95 (s, 3H, Ar-OC*H*$_3$), 3.99 (s, 3H, OC*H*$_3$), 6.97 (dd, ^3J = 8.2, ^4J = 1.0, 1H, *H*-3 aniline), 7.02 (dt, ^3J = 7.7, ^4J = 1.0, 1H, *H*-5 aniline), 7.22-7.27 (m[dt], ^3J = 8.0, ^4J = 1.4, 1H, *H*-4 aniline, $CDCl_3$), 9.26 (dd, ^3J = 8.2, ^4J = 1.4, 1H, *H*-6 aniline), 11.29 (br, s, 1H, N*H*); ^{13}C{^{1}H}NMR (150.93 MHz, $CDCl_3$, 25°C): δ = 54.99 (OCH_3), 56.16 (Ar-OCH_3), 110.44 (*C*-3 aniline), 120.39 (*C*-6 aniline), 120.54 (*C*-5 aniline), 127.60 (*C*-4, *C*-1 aniline[127.54]), 149.83 (*C*-2 aniline), 160.42 (*C*OOMe), 176.95 (NHCS).

2-((2-methoxyphenyl)amino)-2-thioxoacetic acid (20b)

(RN: 7267-58-5); Mp: 133-135°C; HRMS: calc. [$C_9H_9NO_3S$]: 211.03; found: 234.0196 $[M+Na]^+$.

^{1}H NMR (600.25 MHz, DMSO-d$_6$, 25°C): δ = 3.84 (s, 3H, OC*H*$_3$), 7.00 (dt, ^3J = 7.7, ^4J = 1.2, 1H, *H*-5 aniline), 7.16 (dd, ^3J = 8.4, ^4J = 1.0, 1H, *H*-3 aniline), 7.31 (dt, ^3J = 7.9, ^4J = 1.6, 1H, *H*-4 aniline), 8.07 (dd, ^3J = 8.0, ^4J = 1.4, 1H, *H*-6 aniline), 11.73 (s, 1H, N*H*), COO*H* in rapid exchange; ^{13}C{1H}NMR (150.93 MHz, DMSO-d$_6$, 25°C): δ = 55.90 (OCH_3), 111.97 (*C*-6 aniline), 120.07 (*C*-5 aniline), 124.35 (*C*-3 aniline), 126.60 (*C*-1 aniline), 128.26 (*C*-4 aniline), 151.97 (*C*-2 aniline), 162.88 (*C*OOH), 186.40 (NHCS).

Methyl 2-((3-methoxyphenyl)amino)-2-thioxoacetate (21a)

(RN: none) Mp: 86-89°C; HRMS: calc. [$C_{10}H_{11}NO_3S$]: 225.05; found: 248.0353 $[M+Na]^+$.

^{1}H NMR (600.25 MHz, $CDCl_3$, 25°C): δ = 3.83 (s, 3H, OC*H*$_3$), 3.99 (s, 3H, OC*H*$_3$), 6.86 (dd, ^3J = 8.1, ^4J = 2.0, 1H, *H*-4 aniline), 7.33 (t, ^3J = 8.0, 1H, *H*-5 aniline), 7.36 (d, ^3J = 8.2, 1H, *H*-6 aniline), 7.89 (t, ^4J = 2.1, 1H, *H*-2 aniline), 10.57 (s, 1H, N*H*); ^{13}C{^{1}H}NMR (150.93 MHz, $CDCl_3$, 25°C): δ = 55.18 (OCH_3),

55.62 (OCH_3), 107.26 (*C*-2 aniline), 113.52 (*C*-4 aniline), 114.29 (*C*-6 aniline), 129.99 (*C*-5 aniline), 138.88 (*C*-1 aniline), 160.13 (*C*-3 aniline), 160.24 (*C*OOMe), 178.50 (NHCS).

2-((3-methoxyphenyl)amino)-2-thioxoacetic acid (21b)

(RN: 276693-17-5); Mp: 97-98°C; HRMS: calc. [$C_9H_9NO_3S$]: 211.03; found: 234.0196 $[M+Na]^+$.

^{1}H NMR (600.25 MHz, DMSO-d$_6$, 25°C): δ = 3.75 (s, 3H, OC*H*$_3$), 6.87 (dd, ^3J = 8.3, ^4J = 2.5, 1H, *H*-4 aniline), 7.34 (t, ^3J = 8.1, 1H, *H*-5 aniline), 7.47 (dd, ^3J = 8.0, ^4J = 2.0, 1H, *H*-6 aniline), 7.68 (t, ^4J = 2.1, 1H, *H*-2 aniline), 12.27 (s, 1H, N*H*), COO*H* in rapid exchange; ^{13}C{1H}NMR (150.93 MHz, DMSO-d$_6$, 25°C): δ = 55.24 (OCH_3), 108.37 (*C*-2 aniline), 112.24 (*C*-4 aniline), 114.94 (*C*-6 aniline), 129.59 (*C*-5 aniline), 139.45 (*C*-1 aniline), 159.22 (*C*-3 aniline), 163.91 (*C*OOH), 187.53 (NHCS).

Methyl 2-((4-methoxyphenyl)amino)-2-thioxoacetate (22a)

(RN: none); Mp: 134-143°C; HRMS: calc. [$C_{10}H_{11}NO_3S$]: 225.05; found: 248.0353 $[M+Na]^+$.

^{1}H NMR (600.25 MHz, $CDCl_3$, 25°C): δ = 3.83 (s, 3H, OC*H*$_3$), 3.98 (s, 3H, Ar-OC*H*$_3$), 6.94 (m, 2H, *H*-3, *H*-5 aniline), 7.90 (m, 2H, *H*-2, *H*-6 aniline), 10.51 (s, 1H, N*H*); ^{13}C{^{1}H}NMR (150.93 MHz, $CDCl_3$, 23°C): δ = 55.07 (Ar-OCH_3), 55.64 (OCH_3), 114.27 (*C*-3, *C*-5 aniline), 123.74 (*C*-2, *C*-6 aniline), 130.90 (*C*-1 aniline), 158.53 (*C*-4 aniline), 160.52 (*C*OOMe), 177.73 (NHCS).

2-((4-methoxyphenyl)amino)-2-thioxoacetic acid (22b)

(RN: 946-61-2); Mp: 139-140°C; HRMS: calc. [$C_9H_9NO_3S$]: 211.03; found: 234.0196 $[M+Na]^+$.

^{1}H NMR (600.25 MHz, DMSO-d$_6$, 25°C): δ = 3.77 (s, 3H, OC*H*$_3$), 6.98 (d, ^3J = 8.8, 2H, *H*-3, *H*-5 aniline), 7.83 (d, ^3J = 8.4, 2H, *H*-2, *H*-6 aniline), 12.19 (s, 1H, N*H*), COO*H* in rapid exchange; ^{13}C{^{1}H}NMR (150.93 MHz, DMSO-d$_6$, 25°C): δ = 55.35 (OCH_3), 113.78 (*C*-3, *C*-5 aniline), 124.41 (*C*-2, *C*-6 aniline), 131.37 (*C*-1 aniline), 157.51 (*C*-4 aniline), 164.00 (*C*OOH), 186.19 (NHCS).

N1-(pyridin-2-yl)oxalamide (23)

(RN: 52781-00-7); Mp: 156-157 ^{0}C (chloroform)

^{1}H NMR (600.25 MHz, $CDCl_3$, 25 °C): δ = 6.11 (s, br, 1H, NHCOCON*H*$_a$H$_b$), 7.12 (dd, $^3J_1$ = 7.2, $^3J_2$ = 5.2, 1H, *H*-5), 7.44 (s, br, 1H, NHCOCONH$_a$*H*$_b$), 7.76 (ddd, $^3J_1$ = 7.2, $^3J_2$ = 7.2, ^4J = 1.5, 1H, *H*-4), 8.20 (m[d]), 1H, *H*-3), 8.37 (m[dd]), 1H, *H*-6), 9.72 (s, br, 1H, N*H*COCONH$_a$H$_b$); ^{13}C{^{1}H}NMR (150.93 MHz, $CDCl_3$, 25 °C): δ = 114.10 (*C*-3), 121.01 (*C*-5), 138.55 (*C*-4), 148.63 (*C*-6), 149.94 (C_q-2), 157.58 ($NHCOCONH_2$), 161.53 $NHCOCONH_2$).

***N*¹-(naphthalen-2-yl)oxalamide (24)**

(RN: 21775-72-4); Mp: 143-147°C; HRMS: calc. [$C_5H_5NO_3$]: 214.07; found: $[M+Na]^+$.

^{1}H NMR (600.25 MHz, DMSO-d$_6$, 25°C): δ = 7.44 (dt, ^3J = 7.4, ^4J = 1.1, 1H, *H*-6 naphthyl), 7.49 (dt, ^3J = 7.4, ^4J = 1.1, 1H, *H*-7 naphthyl), 7.83 (d, ^3J = 8.2, 1H, *H*-8 naphthyl), 7.86 (d, ^3J = 8.1, 1H, *H*-5 naphthyl), 7.88 (m[d], 2H, *H*-3, *H*-4 naphthyl), 8.04 (s, 1H, N*H*$_2$), 8.34 (s, 1H, N*H*$_2$), 8.47 (s, 1H, *H*-1 naphthyl), 10.77 (br, s, 1H, N*H*); ^{13}C{^{1}H}NMR (150.93 MHz, DMSO- d$_6$, 25°C): δ = 116.76 (*C*-1 naphthyl), 120.65 (*C*-3 naphthyl), 125.19 (*C*-6 naphthyl), 126.53 (*C*-7 naphthyl), 127.52 (*C*-5 naphthyl), 127.56 (*C*-8 naphthyl), 128.35 (*C*-4 naphthyl), 130.31 (*C*-4a naphthyl), 133.14 (*C*-8a naphthyl), 135.37 (*C*-2 naphthyl), 159.15 ($COONH_2$), 162.17 (NHCO).

N-(6-methylpyridin-2-yl)-2-oxo-2-(pyrrolidin-1-yl)acetamide (25)

(RN: 1210298-59-1; no references); Mp: 111-112.5 ^{0}C; HRMS: calc. [$C_{12}H_{15}N_3O_2$]: 233.1164; found: 234.1237 $[M+H]^+$.

^{1}H NMR (600.25 MHz, $CDCl_3$, 25 °C): δ = 1.87 (m[q], 2H, $^3J_{1-4}$ = 6.8, 2H, *H*-4′), 1.98 (m[q], 2H, $^3J_{1-4}$ = 6.8, 2H, *H*-3′), 2.47 (s, 3H, 6-C*H*$_3$), 3.61 (t, $^3J_1$ = $^3J_2$ = 7.0, 2H, *H*-5′), 4.04 (t, $^3J_1$ = $^3J_2$ = 6.8, 2H, *H*-2′), 6.93 (d, ^3J = 7.4, 1H, *H*-5), 7.60 (t, $^3J_1$ = $^3J_2$ = 7.7, 1H, *H*-4), 7.99 (d, ^3J = 8.1, 1H, *H*-3), 9.80 (s, 1H, N*H*COCON); $^{13}C\{^1H\}$NMR (150.93 MHz, $CDCl_3$, 25 °C): δ = 23.55 (*C*H$_2$-4′), 24.24 (6-*C*H$_3$), 27.03 (*C*H$_2$-3′), 48.43 (*C*H$_2$-5′), 49.00 (*C*H$_2$-2′), 110.69 (*C*-3), 120.01 (*C*-5), 138.58 (*C*-4), 149.77 (*C*$_q$-6), 157.56 (*C*$_q$-2), 158.63 (NH*C*OCON), 158.73 (NHCO*C*ON).

2-(2-*tert*-Butoxy-2-oxoacetamido)benzoic acid (26)

(RN: none); Mp: 139-142 ^{0}C (at 142 ^{0}C dec.); HRMS: calc. [$C_{13}H_{15}NO_5$]: 265.0950; found: 288.0842 $[M+H]^+$.

^{1}H NMR (600.25 MHz, $CDCl_3$, 25 °C): δ = 1.63 (2, 9H, NHCOCOOC(C*H*$_3$)$_3$), 7.23 (m[ddd], $^3J_1$ = $^3J_2$ = 7.7, ^4J = 0.8, 1H, *H*-5), 7.67 (m[ddd], $^3J_1$ = $^3J_2$ = 7.9, ^4J = 1.4, 1H, *H*-4), 8.20 (dd, ^3J = 8.0, ^4J = 1.4, 1H, *H*-6), 8.81 (m[d], ^3J = 8.4, 1H, *H*-3), 9.2-11.0 (br, 1H, COO*H*), 12.32 (s, 1H, N*H*COCOOC(CH$_3$)$_3$); $^{13}C\{^1H\}$NMR (150.93 MHz, $CDCl_3$, 25 °C): δ =27.87 (NHCOCOOC(*C*H$_3$)$_3$), 85.32 (NHCOCOO*C*(CH$_3$)$_3$), 115.20 (*C*$_q$-1), 120.74 (*C*-3), 124.22 (*C*-5), 32.24 (*C*-6), 136.00 (*C*-4), 140.45 (*C*$_q$-2), 155.86 (NH*C*OCOOC(CH$_3$)$_3$), 158.85 (NHCO*C*OOC(CH$_3$)$_3$).

3-(2-*tert*-Butoxy-2-oxoacetamido)benzoic acid (27)

(RN: none); Mp: 148 ^{0}C (dec.); HRMS: calculated [$C_{13}H_{15}NO_5$]: 265.0950; found: 288.0842 $[M+Na]^+$.

^{1}H NMR (600.25 MHz, DMSO-d$_6$, 25 °C): δ = 1.53 (s, 9H, NHCOCOOC(C*H*$_3$)$_3$), 7.45 (t, $^3J_1$ = $^3J_2$ = 7.7, 1H, *H*-5), 7.71 (d, br, ^3J = 7.8, 1H, *H*-6), 7.91 (d, br, ^3J = 7.9, 1H, *H*-4), 8.34 (s[t], br, 1H, *H*-2), 10.81 (s, br, 1H, N*H*COCOOC(CH$_3$)$_3$), (COO*H* in exchange with water in DMSO-d$_6$); $^{13}C\{^1H\}$NMR (150.93 MHz, DMSO-d$_6$, 25 C): = 27.45 (NHCOCOOC(*C*H$_3$)$_3$), 83.81 (NHCOCOO*C*(CH$_3$)$_3$), 121.28 (*C*-2), 124.19 (*C*-4), 125.45 (*C*-6), 128.90 (*C*-5), 132.64 (C$_q$-1), 137.70 (C$_q$-3), 156.59 (NH*C*OCOOC(CH$_3$)$_3$), 159.82 (NHCO*C*OOC(CH$_3$)$_3$), 167.38 (*C*OOH)

4-(2-*tert*-Butoxy-2-oxoacetamido)benzoic acid (28)

(RN: 614760-53-1); Mp: oil; HRMS: calc. [$C_{13}H_{15}NO_5$]: 265.0950; found: 288.0842 $[M+Na]^+$.

^{1}H NMR (600.25 MHz, DMSO-d$_6$, 25 °C): δ = 1.53 (s, 9H, NHCOCOOC(C*H*$_3$)$_3$); 7.84, 7.85, (m, AA′, 2H, *H*-3,5 phenyl), 7.92, 7.93 (m, BB′, 2H, *H*-2,6 phenyl), 10.94 (N*H*COCOOC(CH$_3$)$_3$), (COO*H* in exchange with water in DMSO-d$_6$); $^{13}C\{^1H\}$NMR (150.93 MHz, DMSO-d$_6$, 25 °C): δ = 27.46 (NHCOCOOC(*C*H$_3$)$_3$), 84.00 (NHCOCOO*C*(CH$_3$)$_3$), 119.86 (*C*-3,5 phenyl), 126.57 (*C*$_q$-1), 130.33 (*C*-2,6 phenyl), 141.62 (*C*$_q$-4), 156.75 (NH*C*OCOOC(CH$_3$)$_3$), 159.73 (NHCO*C*OOC(CH$_3$)$_3$), 166.88 (phenyl-1-*C*OOH).

***N*^{1},*N*^{2}-bis(5-methylpyridin-2-yl)oxalamide (29)**

(RN: 349401-68-9); Mp: 230-235°C; HRMS: calc. [$C_{14}H_{14}N_4O_2$]: 270.1117; found: 271.1190 $[M+H^+]^+$.

^{1}H NMR (600.25 MHz, $CDCl_3$, 25 °C): δ = 2.33 (s, 6H, 2x 5-C*H*$_3$), 7.58 (dd, ^3J = 8.5, ^4J = 2.1, 2H, 2x *H*-3), 8.15 (d, ^3J = 8.5, 2H, 2x *H*-4), 8.20 (s, br, 2H, 2x *H*-6), 9.73 (s, br, 2H, 2x N*H*COCON*H*);

$^{13}C\{^1H\}$NMR (150.93 MHz, $CDCl_3$, 25 °C): δ = 18.12 (5-CH3), 113.68 (*C*-3), 130.64 (C_q-5), 139.02 (*C*-6), 147.74 (*C*-4), 148.54 (C_q-2), 157.42 (NHCOCONH).

N^1,N^2-di(pyridin-2-yl)oxalamide (30)

(RN: 20172-97-8); Mp: 163-164 ^{0}C.

^{1}H NMR (600.25 MHz, $CDCl_3$, 25 °C): δ = 7.14 (ddd, $^3J_1$ = 7.3, $^3J_2$ = 5.0, ^4J = 1.8, 1H, 2x *H*-5), 7.78 (ddd, $^3J_1$ = $^3J_2$ = 7.8, ^4J = 1.8, 1H, 2x *H*-4), 8.27 (d, ^3J = 7.8, 1H, 2x *H*-3), 8.39 (m[d], ^3J = 4.9, 2H, 2x *H*-6); 9.81 (s, 2H, N*H*COCON*H*); $^{13}C\{^1H\}$NMR (150.93 MHz, $CDCl_3$, 25 °C): δ = 114.27 (*C*-3), 121.13 (*C*-5), 138.65 (*C*-4), 148.64 (*C*-6), 149.93 (C_q-2), 157.51 (NHCOCONH).

***N*,*N′*-Di-pyridin-3-yl-oxalamide (31)**

(RN: 39642-61-0); Mp: 250-255°C; HRMS: calc. [$C_{12}H_{10}N_4O_2$]: 242.24; found: 243.0876 [M+H$^+$]$^+$.

^{1}H NMR (600.25 MHz, DMSO-d$_6$, 25 °C): δ = 7.44 (m, ^4J = 4.3, 1H ,2x *H*-5), 8.24 (ddd, ^3J = 8.4, $^4J_1$ = 3.3, $^4J_2$ = 1.7, 1H, 2x *H*-4), 8.38 (dd, ^3J = 4.8, ^4J = 0.9, 1H, 2x *H*-6), 9.05 (d, ^4J = 2.2, 1H, 2x *H*-2), 11.16 (s, 2H, 2x N*H*COCOON*H*); $^{13}C\{^1H\}$NMR (150.93 MHz, $CDCl_3$, 25 °C): δ = 123.55 (*C*-5), 127.59 (*C*-4), 134.37 (C_q-3), 142.28 (*C*-2), 145.52 (*C*-6), 158.58 (NHCOCONH).

***N*-Pyridin-2-yl-*N′*-*p*-tolyl-oxalamide (32)**

(RN: 301344-55-8); Mp: 176-178°C; HRMS: calc. [$C_{14}H_{14}N_3O_3$]: 255.28; found: 256.1083 [M+H$^+$]$^+$.

1H NMR (600.25 MHz, $CDCl_3$, 25 °C): δ = 2.35 (s, 3H, 4-C*H*$_3$), 7.19 (m[d]; AA′ – part of AA′BB′ – spin system ^3J = 8.3, *H*-2, *H*-5), 7.56 (m[d]; BB′ – part of AA′BB′ spin system ^3J = 8.2 , *H*-3, *H*-6), 7.14 ([m]t, $^3J_1$ = 6.1, $^3J_2$ = 6.1, 1H, *H*-5\`), 7.77 (t, br, $^3J_1$ = 7.8, $^3J_2$ = 7.8, 1H, *H*-4\`), 8.24 (d, br, $^3J_1$ = 8.3, 2H, *H*-3\`), 8.39(d, br, $^3J_1$ = 4.9, 2H, *H*-6\`), 9.26 (s, br, 1H, N*H*COCOONH), 9.88 (s, br, 1H, NHCOCOON*H*); $^{13}C\{^1H\}$NMR (150.93 MHz, $CDCl_3$, 25 °C): δ = 21.18 (4-*C*H$_3$), 119.97 (*C*-3), 129.98 (*C*-5), 133.85 (C_q-4), 135.46 (C_q-1), 114.16 (*C*-3\`), 121.10 (*C*-5\`), 138.53 (*C*-4\`), 148.54 (*C*-6\`), 149.71 (C_q-2\`), 156.77 (NH*C*OCOONH), 158.39 (NHCO*C*OONH).

***N*-Pyridin-2-yl-*N′*-*m*-tolyl-oxalamide (33)**

(RN: 920210-16-8; no reference); Mp: 134-135°C; HRMS: calc. [$C_9H_{10}N_2O_3$]: 194.0691; found: 217.0584 [M+Na$^+$]$^+$

1H NMR (600.25 MHz, $CDCl_3$, 25 °C): δ = 2.35 (s, 3H, 3-C*H*$_3$), 7.01 (d, ^3J = 7.5, *H*-4), 7.25 (t, $^3J_1$ = 7.8, $^3J_2$= 7.8, 1H, *H*-5) 7.47 (d, ^3J = 8.2, 1H, *H*-6), 7.50 (s, 1H, *H*-2), 9.33 (s, br, 1H, N*H*COCOONH), 9.93 (s, br, 1H, NHCOCOON*H*), 7.12 ([m]ddd, $^3J_1$ = 6.2, $^3J_2$ = 4.0, ^4J = 1.0, 1H, *H*-5\`), 7.74 (ddd, $^3J_1$ = 8.0, $^3J_2$ = 6.0, $^4J$ = 1.9, 1H, *H*-4\`), 8.23 (d, br, ^3J = 8.4, 1H, *H*-3\`), 8.38 (ddd, br, $^3J_10$ = 4.8, $^3J_2$ = 1.9, $^4J$ = 0.8, 1H , *H*-6\`); $^{13}C\{^1H\}$NMR (150.93 MHz, $CDCl_3$, 25 °C): δ= 114.12 (*C*-3\`), 120.19 (*C*-5\`), 138.49 (*C*-4\`), 148.66 (*C*-6\`), 149.96 (C_q-2\`), 157.06 (NH*C*OCOOCH$_3$), 158.19 (NHCO*C*OOCH$_3$), 21.63 (3-*C*H$_3$), 117.18 (*C*-2), 121.06 (*C*-6), 126.55 (*C*-4), 128.81 (*C*-5), 136.25 (C_q-3), 139.30 (C_q-1).

***N*-Pyridin-3-yl-*N′*-pyridin-2-yl-oxalamide (34)**

(RN: 1796089-38-7); Mp. 163-165°C; HRMS: calc. [$C_{12}H_{10}N_4O_2$]: 242.24; found: 243.0879 [M+H$^+$]$^+$.

^{1}H NMR (600.25 MHz, $CDCl_3$, 25 °C): δ = 7.14 (ddd, $^3J_1$ = 4.9, $^3J_2$ = 7.0, ^4J = 0.5, 1H, *H*-5), 7.78 (ddd, $^3J_1$ = 7.5, $^3J_2$ = 7.8, ^4J = 1.8, 1H, *H*-4), 8.27 (d, br, ^3J = 8.3, 1H, *H*-3), 8.39 (d, br, ^3J = 4.8, 1H, *H*-6), 9.81 (s, br, 1H, N*H*COCOOCH$_3$), 7.34 (m, ^4J = 4.6, 1H, *H*-5\`) , 8.23 (d, $^4J$ = 8.3, 1H, *H*-4\`), 8.46 (dd, ^3J = 4.8, ^4J = 1.3, 1H, *H*-6\`), 8.80 (d, $^4J$ = 2.7, 1H, *H*-2\`); ^{13}C{^{1}H}NMR (150.93 MHz, $CDCl_3$, 25 °C): δ= 114.40 (*C*-3), 121.17 (*C*-5), 138.73 (*C*-4), 148.51 (*C*-6), 149.93 (C_q-2), 157.26 (NH*C*OCOOCH$_3$), 158.00 (NHCO*C*OOCH$_3$), 124 (*C*-5\`), 127 (*C*-4\`), 133 (C_q-3\`) , 141 (*C*-2\`), 146 (*C*-6\`).

N-(1-Oxy-pyridin-2-yl)-*N'*-*m*-tolyl-oxalamide (35)

(RN: none); Mp: 253-255°C; HRMS: calc. [$C_{14}H_{13}N_3O_3$]: 271.28; found: 294.0850 [M+Na$^+$]$^+$.

^{1}H NMR (600.25 MHz, DMSO- d$_6$, 25 °C): δ = 2.32 (s, 3H, 3-C*H*$_3$), 7.01 (d, ^3J = 7.6, *H*-4), 7.27 (t, ^3J = 2.7, 1H, *H*-5), 7.28 (s, 1H, *H*-2), 7.65 (d, ^3J = 8.3, 1H, *H*-6), 10.95 (s, br, 1H, N*H*COCOONH), 11.46 (s, br, 1H, NHCOCOON*H*), 7.54 (m[ddd], $^3J_1$ = 8.7, $^3J_2$ = 8.1, ^4J = 1.1, 1H, *H*-5\`), 7.71 (s, br, 1H, *H*-4\`), 8.34 (dd, ^3J = 8.4, ^4J = 1.9, 1H, *H*-6\`), 8.48 (dd, $^3J$ = 6.6, $^4J$ = 1.2, 1H, *H*-3\`); ^{13}C{^{1}H}NMR (150.93 MHz, DMSO-d6, 25°C): δ= 21.19 (3-*C*H$_3$), 117.90 (*C*-6), 121.26 (*C*-5), 125.57 (*C*-4), 127.65 (*C*-2), 137.18 (C_q-3), 137.63 (C_q-1), 156.91 (NH*C*OCOONH), 158.02 (NHCO*C*OONH), 120.46 (*C*-4\`), 128.60 (*C*-5\`), 138.01 (*C*-3\`), 113.90 (*C*-6\`), 142.34 (C_q-2\`).

***N*-(1-Oxy-pyridin-2-yl)-*N'*-*p*-tolyl-oxalamide (36)**

(RN: none); Mp: nd (contains residual 3-chlorobenzoic acid); HRMS: calc. [$C_{14}H_{13}N_3O_3$]: 271,2780 ; found: 272.1029 [M+H]$^+$.

^{1}H NMR (600.25 MHz, DMSO- d$_6$, 25 °C): δ = 2.31 (s, 3 H, 4-C*H*3), 7.00 (d, br, 1 H), 7.26 (t, br, 1 H), 7.54 (d, br, 2 H), 7.65 (d, br, 1H), 7.68 – 7.72 (m, br, 2 H), 7.88 – 7.91 (m, br, 3 H), 8.34 (dd, 1 H), 8.48 (d, br, 1H), 10,95 (s, br, 1H), 11.46 (s, br, 1H), 13.00 – 13.6 (br, 1H).^{13}C{^{1}H}NMR (150.93 MHz, DMSO-d6, 25 °C): δ = 21.20 (4-CH$_3$), 114.01 (CH), 118.00 (CH), 120.66 (CH), 121.26 (CH), 125.72 (CH), 127.66 (CH), 127.92 (CH), 128.61 (CH), 128.83 (CH), 130.66 (CH), 132.71 (CH), 132.95 (C_q), 133.35 (C_q), 137.20 (C_q), 137.64 (CH), 138.03 (C_q), 142.37 (C_q), 156.92 (C_q), 158.08 (C_q),166.08 (C_q).

***N*-(1-Oxy-pyridin-3-yl)-*N'*-*m*-tolyl-oxalamide (37)**

(RN: none); Mp: 231-232 °C; HRMS: calc. [$C_{14}H_{13}N_3O_3$]: 271.28; found: 272.1026 [M+H$^+$]$^+$.

^{1}H NMR (600.25 MHz, DMSO- d$_6$, 25 °C): δ = 2.31 (s, 3H, 3-C*H*$_3$), 6.99 (d, ^3J = 7.5, *H*-4), 7.26 (t, $^3J_1$ = 7.7, $^3J_2$= 7.7, 1H, *H*-5) 7.66 (d, ^3J = 8.8, 1H, *H*-6), 7.67 (s, 1H, *H*-2), 10.80 (s, br, 1H, N*H*COCOONH), 11.26 (s, br, 1H, NHCOCOON*H*), 7.43 (m[dd], $^3J_1$= 8.5, $^3J_2$= 7.7, ^4J = 1.3, 1H, *H*-3\`), 7.82 (dd, $^3J$ = 8.5, $^4J$ = 1.7, 1H, *H*-6\`), 8.05 (dd, ^3J = 6.5, ^4J = 1.6, 1H, *H*-2\`), 8.85 (t, $^3J_1$ = 1.6, $^3J_2$= 1.6, 1H, *H*-4\`); ^{13}C{^{1}H}NMR (150.93 MHz, DMSO-d$_6$, 25 °C): δ= 21.24 (3-*C*H$_3$), 116.80 (*C*-6), 121.15 (*C*-2), 125.35 (*C*-4), 128.58 (*C*-5), 136.97 (C_q-3), 137.94 (C_q-1), 157.62 (NH*C*OCOONH), 159.41 (NHCO*C*OONH), 126.00 (*C*-3\`), 117.60 (*C*-6\`), 131.16 (*C*-4\`), 137.15 (*C*-2\`).

Methyl 2-((6-methylpyridin-2-yl)amino)-2-oxoacetate (S1)

(RN: 1566684-50-1; no references); Mp: 68-69 °C; HRMS: calc. [$C_9H_{10}N_2O_3$]: 194.0691; found: 217.0584 [M+Na]$^+$; [FAHD1: IC_{50} = 33 μM].

^{1}H NMR (600.25 MHz, $CDCl_3$, 25 °C): δ = 2.48 (s, 3H, C*H*$_3$-6), 3.96 (s, 3H, NHCOCOOC*H*$_3$), 6.98 (d, ^3J = 7.5, 1H, *H*-5), 7.64 (t, ^3J = 7.8, 1H, *H*-4), 8.04 (d, ^3J = 8.1, 1H, *H*-3), 9.32 (s, br, 1H, N*H*COCOOCH$_3$); ^{13}C{^{1}H}NMR (150.93 MHz, $CDCl_3$, 25 °C): δ = 24.15 (CH$_3$-6), 54.22 (COOCH$_3$), 111.23 (*C*-3), 120.72 (*C*-5), 138.96 (*C*-4), 149.12 (*C*$_q$-6), 154.00 (NHCOCOOCH$_3$), 157.61 (*C*$_q$-2), 160.76 (NHCOCOOCH$_3$).

Methyl 2-((2-methoxyphenyl)amino)-2-oxoacetate (S2)

(RN: 113449-16-4); Mp: 85-87°C; HRMS: calc. [$C_{10}H_{11}NO_4$]: 209.07; found: 210.0773 [M+H]$^+$.

^{1}H NMR (600.25 MHz, $CDCl_3$, 25°C): δ = 3.91 (s, 3H, Ar-OC*H*$_3$), 3.96 (s, 3H, OC*H*$_3$), 6.91 (dd, ^3J = 8.3, ^4J = 1.0, 1H *H*-3 aniline), 6.99 (dt, ^3J = 7.9, ^4J = 1.0, 1H *H*-5 aniline), 7.13 (dt, ^3J = 7.9, ^4J = 1.5, 1H *H*-4 aniline), 8.40 (dd, ^3J = 8.3, ^4J = 1.5, 1H *H*-6 aniline), 9.47 (s, 1H, N*H*); ^{13}C{^{1}H}NMR (150.93 MHz, $CDCl_3$, 25°C): δ = 54.05 (OCH$_3$), 55.90 (Ar-OCH$_3$), 110.22 (*C*-3 aniline), 120.05 (*C*-6 aniline), 121.19 (*C*-5 aniline), 125.47 (*C*-4 aniline), 126.10 (*C*-1 aniline), 148.56 (*C*-2 aniline), 153.51 (NH*C*O), 161.45 (*C*OOMe).

Methyl 2-((3-methoxyphenyl)amino)-2-oxoacetate (S3)

(RN: 103448-86-8); Mp: 70-72°C; HRMS: calc. [$C_{10}H_{11}NO_4$]: 209.07; found: 210.0773 [M+H]$^+$.

^{1}H NMR (600.25 MHz, $CDCl_3$, 23°C): δ = 3.81 (s, 3H, Ar-OC*H*$_3$), 3.96 (s, 3H, OC*H*$_3$), 6.74 (dd, ^3J = 8.3, ^4J = 2.1, 1H *H*-4 aniline), 7.12 (dd, ^3J = 8.3, ^4J = 2.1, 1H *H*-6 aniline), 7.26 (t, ^3J = 8.3, 1H, *H*-5 aniline), 7.36 (t, ^4J = 2.1, 1H, *H*-2 aniline), 8.86 (s, 1H, N*H*); ^{13}C{^{1}H}NMR (150.93 MHz, $CDCl_3$, 23°C): δ = 54.21 (OCH$_3$), 55.47 (Ar-OCH$_3$), 105.72 (*C*-2 aniline), 111.52 (*C*-4 aniline), 112.18 (*C*-6 aniline), 130.08 (*C*-5 aniline), 137.48 (*C*-1 aniline), 153.68 (NHCO), 160.33 (*C*-3 aniline), 161.54 (-*C*OOMe).

Methyl 2-((4-methoxyphenyl)amino)-2-oxoacetate (S4)

(RN: 24439-54-1); Mp: 140-141°C; HRMS: calc. [$C_{10}H_{11}NO_4$]: 209.07; found: 210.0773 [M+H]$^+$.

^{1}H NMR (600.25 MHz, $CDCl_3$, 25°C): δ = 3.80 (s, 3H, Ar-OC*H*$_3$), 3.95 (s, 3H, OC*H*$_3$), 6.89 (d, ^3J = 9.0, 2H, *H*-3, *H*-5 aniline), 7.56 (d, ^3J = 9.0, 2H, *H*-2, *H*-6 aniline), 8.81 (s, 1H, N*H*); ^{13}C{^{1}H}NMR (150.93 MHz, $CDCl_3$, 25°C): δ = 54.12 (OCH$_3$), 55.59 (Ar-OCH$_3$), 114.45 (*C*-3, *C*-5 aniline), 121.55 (*C*-2, *C*-6 aniline), 129.50 (*C*-1 aniline), 153.47 (NHCO), 157.32 (*C*-4 aniline), 161.74 (COOMe).

2-oxo-2-(*o*-tolylamino)acetic acid (S5)

(RN: 406190-09-8); Mp: 179-180°C; HRMS: calc. [$C_9H_9NO_3$]: 179.0582; found: [M+Na]$^+$.

^{1}H NMR (600.25 MHz, $CDCl_3$, 25°C): δ = 2.35 (s, 3H, 2-C*H*$_3$ of tolyl), 7.18 (m[t], 1H, *H*-5 phenyl), 7.25 (m[d], 1H, *H*-3 phenyl), 7.27 (m[t], 1H, *H*-4 phenyl), 7.94 (m[d], 1H, *H*-3 phenyl), 8.20-8.80 (br, 1H, COO*H*), 8.99 (s, br, 1H, N*H*COCOOCH$_3$); ^{13}C{^{1}H}NMR (150.93 MHz, $CDCl_3$, 25°C): δ = 17.54 (2-CH$_3$-phenyl), 121.83 (*C*-6 tolyl), 126.85 (*C*-4 tolyl), 127.27 (*C*-5 tolyl), 129.01 (*C*$_q$-2 tolyl), 131.02 (*C*-3 tolyl), 133.61 (*C*$_q$-1 tolyl), 155.13 (COCOOH), 160.48 (COCOOH).

2-(((1*R*,2*S*)-2-hydroxy-2,3-dihydro-1H-inden-1-yl)amino)-2-oxoacetic acid (S6)

(RN: 1849465-46-8); Mp: 204-205°C; HRMS: calc. [$C_{11}H_{11}NO_4$]: 221.07; found: 244.0580 [M+Na]$^+$.

^{1}H NMR (600.25 MHz, DMSO-d$_6$, 25°C): δ = 2.85 (m[dd], 1H, ^2J = 16.1, ^3J = 1.5, H_{eq} indene), 3.10 (m[dd], 1H, ^2J = 16.2, ^3J = 5.1, H_{ax} indene), 4.48 (m[dt], 1H *H*-2 indene), 5.18 (m[q], 1H, *H*-1 indene), 5.38 (br, s, 1H, O*H*), 7.16-7.20 (m, 2H, *H*-5 indene, *H*-7 indene), 7.20-7.24 (m, 1H, *H*-6 indene), 7.25 (m[d], 1H *H*-4 indene), 8.18 (d, 1H, ^3J = 8.6, N*H*), 14.07 (br, s, 1H, COO*H*); ^{13}C{^{1}H}NMR (150.93 MHz, DMSO-d$_6$, 25°C): δ = 39.76 (*C*-3 indene), 56.90 (*C*-1 indene), 71.63 (*C*-2 indene), 124.22 (*C*-4, *C*-5, *C*-6, *C*-7 indene), 125.02 (*C*-4, *C*-5, *C*-6, *C*-7 indene), 126.53 (*C*-4, *C*-5, *C*-6, *C*-7 indene), 127.72 (*C*-4, *C*-5, *C*-6, *C*-7 indene), 140.79 (*C*-4a indene), 141.15 (*C*-7a indene), 158.19 (NHCO), 162.02 (COOH).

Methyl 2-(*tert*-butylamino)-2-oxoacetate (S7)

(RN: 1450740-20-1 (patent); Mp: oil; HRMS: calc. [$C_7H_{13}NO_3$]: 159.0895; found: 182.0788 [M+Na]$^+$.

^{1}H NMR (600.25 MHz, $CDCl_3$, 25 ^{0}C): δ = 1.39 (s, 9H, (C*H*$_3$)$_3$CNH), 3.86 (s, 3H, (COOC*H*$_3$), 6.96 (s, br, 1H, (CH$_3$)$_3$CN*H*); ^{13}C{^{1}H}NMR (150.93 MHz, $CDCl_3$, 25 ^{0}C): δ = 28.29 ((CH$_3$)$_3$CNH), 52.06 ((CH$_3$)$_3$CNH), 53.70 (COOCH$_3$), 155.35 (NHCOCOOCH$_3$), 161.96 (NHCOCOOCH$_3$).

2-(benzylamino)-2-oxoacetic acid (S8)

(RN: 6345-08-0): Mp: 126-127°C; HRMS: calc. [$C_9H_9NO_3$]: 179.06; found: 202.1647 [M+Na]$^+$.

^{1}H NMR (600.25 MHz, $CDCl_3$, 25°C): δ = 4.52 (d, ^{3}J = 6.2, 2H, phenyl-C*H*$_2$), 7.27-7.30 (m, 2H, *H*-2,6 benzyl), 7.31-7.35 (m, 1H, *H*-4 benzyl), 7.34-7.39 (m, 2H, *H*-3,5 benzyl), 7.68 (s, br, 1H, N*H*CO), 7.80-8.20 (br, COO*H*); ^{13}C{^{1}H}NMR (150.93 MHz, $CDCl_3$, 25°C): δ = 47.02 (phenyl-*C*H$_2$), 128.10 (*C*-2,6 benzyl), 128.44 (*C*-4 benzyl), 129.15 (*C*-3,5 benzyl), 135.85 (*C*-1 benzyl), 157.48 (NHCO), 159.97 (COOH).

Methyl 2-(((1*S*,2*R*)-1-hydroxy-1-phenylpropan-2-yl)amino)-2-oxoacetate (S9)

(RN: none); Mp: 112-115°C; HRMS: calc. [$C_{12}H_{15}NO_4$]: 237.10; found: 260.0941[M+Na]$^+$.

^{1}H NMR (600.25 MHz, $CDCl_3$, 25°C): δ = 1.04 (d, ^3J = 6.8, 3H, CHC*H*$_3$), 2.83 (d, ^3J = 3.0, 1H, O*H*), 3.88 (s, 3H, OC*H*$_3$), 4.29 (m, 1H, C*H*CH$_3$), 4.92 (s, 1H, C*H*OH), 7.29 (m, 1H, *H*-4, aryl), 7.35 (m[d], 4H, *H*-2, 3, 5, 6 aryl), 7.41 (br, d, ^3J = 8.3, 1H, N*H*); ^{13}C{^{1}H}NMR (150.93 MHz, $CDCl_3$, 25°C): δ = 13.60 (CHCH$_3$), 51.40 (CHCH$_3$), 53.76 (OCH$_3$), 75.43 (CHOH), 126.12 (*C*-2, 3, 5, 6 aryl), 127.96 (*C*-4 aryl), 128.54 (*C*-2, 3, 5, 6 aryl), 140.40 (*C*-1 aryl), 156.16 (NHCO), 161.21 (COOMe).

2-((4-fluorophenyl)amino)-2-oxoacetic acid (S10)

(RN: 69066-43-9); Mp: 155-159°C; HRMS: calc. [$C_8H_6FNO_3$]: 183.03; found: 206.0232 [M+Na]$^+$.

^{1}H NMR (600.25 MHz, DMSO-d$_6$, 25°C): δ = 7.13-7.23 (m, br, 2H, *H*-3, *H*-5 aniline), 7.75-7.83 (m, br, 2H, *H*-2, *H*-6 aniline), 10.79 (s, 1H, N*H*), 14.24 (br, s, 1H, COO*H*); ^{13}C{^{1}H}NMR (150.93 MHz, DMSO-d$_6$, 25°C): δ = 115.40 (d, ^2J = 22.3, *C*-3, *C*-5 aniline), 122.21 (d, ^3J = 7.9, *C*-2, *C*-6 aniline), 134.17 (*C*-1 aniline), 156.88 (NHCO), 158.75 (d, ^1J = 241.1, *C*-4 aniline), 162.09 (COOH).

2-((2-fluorophenyl)amino)-2-oxoacetic acid (S11)

(RN: 84944-15-0); Mp: 162-165°C; HRMS: calc. [$C_8H_6FNO_3$]: 183.03; found: 206.0224 [M+Na]$^+$.

^{1}H NMR (600.25 MHz, DMSO-d$_6$, 25°C): δ = 7.18-7.24 (m, 1H, *H*-6 aniline), 7.24-7.33 (m, 2H, *H*-4, *H*-5 aniline), 7.60-7.69 (m, 1H, *H*-3 aniline), 10.36 (s, 1H, N*H*), 14.12 (br, s, 1H, COO*H*); ^{13}C{^{1}H}NMR (150.93 MHz, DMSO-d$_6$, 25°C): δ = 115.86 (d, ^{2}J = 19.5, *C*-3 aniline), 124.41 (*C*-1 aniline), 124.50 (d, ^{4}J = 3.7, *C*-5 aniline), 125.73 (*C*-6 aniline), 127.28 (d, ^{3}J = 7.7, *C*-4 aniline), 154.95 (d, ^{1}J = 248.8, *C*-2 aniline), 157.31 (NHCO), 161.78 (COOH).

2-((2-bromophenyl)amino)-2-oxoacetic acid (S12)

(RN: 868565-59-7); Mp: 123-125°C: HRMS: calc. [$C_8H_6BrNO_3$]: 242.95; found: 265.9431 [M+Na]$^+$.

^{1}H NMR (700.40 MHz, DMSO-d$_6$, 25°C): δ = 7.20 (dt, ^{3}J = 7.6, ^{2}J = 1.2, 1H, *H*-4 aniline), 7.43 (dd, ^{3}J = 8.1, ^{2}J = 1.3, 1H, *H*-5 aniline), 7.71 (dt, ^{3}J = 7.6, ^{2}J = 1.2, 1H, *H*-3 aniline), 7.81 (dd, ^{3}J = 8.1, ^{2}J = 1.3, 1H, *H*-6 aniline), 10.14 (s, 1H, N*H*), 14.29 (br, s, 1H, COO*H*); ^{13}C{^{1}H}NMR (176.11 MHz, DMSO-d$_6$, 25°C): δ = 117.37 (*C*-2 aniline), 125.26 (*C*-6 aniline), 127.65 (*C*-4 aniline), 128.44 (*C*-5 aniline), 132.76 (*C*-3 aniline), 134.99 (*C*-1 aniline), 156.55 (NHCO), 161.68 (COOH).

2-((3-bromophenyl)amino)-2-oxoacetic acid (S13)

(RN: 946744-52-1); Mp: 146-150°C; HRMS: calc. [$C_8H_6BrNO_3$]: 242.95; found: 265.9430 [M+Na]$^+$.

^{1}H NMR (600.25 MHz, DMSO-d$_6$, 25°C): δ = 7.31 (m, 2H, *H*-4, *H*-5 aniline), 7.76 (m, 1H, *H*-6 aniline), 8.07 (s, 1H, *H*-2 aniline), 10.84 (s, 1H, N*H*), 14.36 (br, s, 1H, COO*H*); ^{13}C{^{1}H}NMR (150.93 MHz, DMSO-d$_6$, 25°C): δ = 119.09 (*C*-6 aniline), 121.46 (*C*-3 aniline), 122.53 (*C*-2 aniline), 127.06 (*C*-4 aniline), 130.74 (*C*-5 aniline), 139.47 (*C*-1 aniline), 157.86 (NHCO), 161.83 (COOH).

2-((2,4-dichlorophenyl)amino)-2-oxoacetic acid (S14)

(RN: 17772-30-4); Mp: 125-128°C; HRMS: calc. [$C_8H_5Cl_2NO_3$]: 232.96; found: 255.9541 [M+Na]$^{+.}$

^{1}H NMR (600.25 MHz, DMSO-d$_6$, 25°C): δ = 7.48 (dd, ^{3}J = 8.7, ^{4}J = 2.3, 1H, *H*-5 aniline), 7.74 (d, ^{4}J = 2.3, 1H, *H*-3 aniline), 7.80 (d, ^{3}J = 8.7, 1H, *H*-6 aniline), 10.26 (s, 1H, N*H*), 13.95 (br, s, 1H, COO*H*); ^{13}C{^{1}H}NMR (150.93 MHz, DMSO-d$_6$, 25°C): δ = 126.55 (*C*-6 aniline), 127.96 (*C*-5 aniline), 128.06 (*C*-4 aniline), 129.13 (*C*-3 aniline), 130.58 (*C*-2 aniline), 132.88 (*C*-1 aniline), 156.97 (NHCO), 161.50 (COOH).

2-((3-hydroxyphenyl)amino)-2-oxoacetic acid (S15)

(RN: 38188-60-2); Mp: 212-214°C; HRMS: calc. [$C_8H_7NO_4$]: 181.04; found: 204.0280 [M+Na]$^+$.

^{1}H NMR (700.40 MHz, DMSO-d$_6$, 25°C): δ = 6.53 (d, ^{3}J = 7.8, 1H, *H*-4 aniline), 7.10 (t, ^{3}J = 7.9, 1H, *H*-5 aniline), 7.14 (d, ^{3}J = 8.3, 1H *H*-6 aniline), 7.31 (d, ^{4}J = 2.0, 1H, *H*-2 aniline), 9.48 (s, 1H, NH), 10.54 (br, s, 1H, ArO*H*), 14.12 (br, s, 1H, COO*H*); ^{13}C{^{1}H}NMR (176.11 MHz, DMSO-d$_6$, 25°C): δ = 107.40 (*C*-2 aniline), 111.12 (*C*-6 aniline), 111.71 (*C*-4 aniline), 129.42 (*C*-5 aniline), 138.69 (*C*-1 aniline), 156.86 (NHCO), 157.56 (*C*-3 aniline), 162.26 (COOH).

2-((2-hydroxyphenyl)amino)-2-oxoacetic acid (S16)

(RN: 89942-67-6); Mp: 250-254°C; HRMS: calc. [$C_8H_7NO_4$]: 181.04; found: 204.0275 $[M+Na]^+$.

^{1}H NMR (700.40 MHz, DMSO-d$_6$, 25°C): δ = 6.83 (dt, ^3J = 7.6, ^4J = 1.1, 1H, *H*-5 aniline), 6.92 (dd, ^3J = 8.1, ^{4}J = 1.2, 1H, *H*-3 aniline), 7.00 (dt, ^3J = 7.7, ^4J = 1.5, 1H, *H*-4 aniline), 8.02 (dd, ^3J = 7.9, ^4J = 1.2, 1H, *H*-6 aniline), 9.59 (s, 1H, N*H*), 10.26 (br, s, 1H, ArO*H*), 14.38 (br, s, 1H, COO*H*); ^{13}C{^{1}H}NMR (176.11 MHz, DMSO-d$_6$, 25°C): 115.04 (*C*-3 aniline), 119.24 (*C*-5 aniline), 120.22 (*C*-6 aniline), 124.99 (*C*-1 aniline), 125.36 (*C*-4 aniline), 147.20 (*C*-2 aniline), 155.39 (NH*C*O), 161.91 (*C*OOH).

2-((2-methoxyphenyl)amino)-2-oxoacetic acid (S17)

(RN: 57727-23-8); Mp: 148-152°C; HRMS: calc. [$C_9H_9NO_4$]: 195.05; found: 196.0618 $[M+H]^+$.

^{1}H NMR (600.25 MHz, DMSO-d$_6$, 25°C): δ = 3.88 (s, 3H, Ar-OC*H*$_3$), 6.98 (t, ^3J = 7.7, 1H *H*-5 aniline), 7.11 (d, ^3J = 7.1, 1H *H*-3 aniline), 7.16 (t, ^3J = 7.9, 1H *H*-4 aniline), 8.08 (d, ^3J = 7.8, 1H *H*-6 aniline), 9.63 (s, 1H, N*H*), 14.48 (br, s, 1H, COO*H*); ^{13}C{^{1}H}NMR (150.93 MHz, DMSO-d$_6$, 25°C): δ = 55.98 (Ar-OCH$_3$), 111.24 (*C*-3 aniline), 120.15 (*C*-6 aniline), 120.59 (*C*-5 aniline), 125.50 (*C*-4 aniline), 125.79 (*C*-1 aniline), 149.09 (*C*-2 aniline), 155.72 (NH*C*O), 161.90 (*C*OOH).

2-((4-methoxyphenyl)amino)-2-oxoacetic acid (S18)

(RN: 41374-62-3); Mp: 155-160°C; HRMS: calc. [$C_9H_9NO_4$]: 195.05; found: 196.0616 $[M+H]^+$

^{1}H NMR (600.25 MHz, DMSO-d$_6$, 25°C): δ = 3.74 (s, 3H, Ar-OC*H*$_3$), 6.92 (d, ^3J = 9.0, 2H, *H*-3, *H*-5 aniline), 7.69 (d, ^3J = 9.0, 2H, *H*-2, *H*-6 aniline), 10.61 (s, 1H, N*H*), 14.14 (br, s, 1H, COO*H*); ^{13}C{^{1}H}NMR (150.93 MHz, DMSO-d$_6$, 25°C): δ = 55.22 (Ar-OCH$_3$), 113.87 (*C*-3, *C*-5 aniline), 121.80 (*C*-2, *C*-6 aniline), 130.82 (*C*-1 aniline), 156.11 (NH*C*O), 156.47 (*C*-4 aniline), 162.34 (*C*OOH).

2-(2-(*tert*-butoxy)-2-oxoacetamido)-5-methylpyridine 1-oxide (S19)

(RN: none; 1:1 clathtrate with 3-chlorobenzoic acid); Mp1 (plates): 157-158 ^{0}C (thermal *t*-butyl cleavage), formation of needles; Mp2: 200-202 ^{0}C); HRMS: calc. [$C_{12}H_{16}N_2O_4$]: 252.1110; found: 275.1002 $[M+Na]^+$.

Parent compound **S19**: ^{1}H NMR (600.25 MHz, CDCl$_3$, 25 °C): δ = 1.60 (s, 9H, NHCOCOOC(C*H*$_3$)$_3$), 2.34 (s, 3H, C*H*$_3$-5), 7.29 (dd, 1H, *H*-4), 8.31 (s, br, 1H, *H*-6), 8.36 (d, ^3J= 6.7, 1H, *H*-3), 11.14 (s, br, 1H, N*H*COCOOC(CH$_3$)$_3$); ^{1}H resonances of complexed 3-chlorobenzoic acid: 7.38 (t, $^3J_1$= $^3J_2$= 7.8, 1H, *H*-5′), 7.53 (ddd, ^3J= 7.9, $^4J_1$= 2.0, $^4J_2$ = 1.0, 1H, *H*-4′), 7.96 (dt, ^3J= 7.9, $^4J_1$= $^4J_2$= 1.2, 1H, *H*-6′), 8.05 (m[t]), $^4J_1$= 2.0, $^4J_2$= 1.0, 1H, *H*-2′), 8.5-10.5 (vbr, 1H, COO*H*);

Parent compound **S19**: ^{13}C{^{1}H}NMR (150.93 MHz, CDCl$_3$, 25 °C): δ = 18.21 (*C*H$_3$-5), 27.81 (NHCOCOOC(*C*H$_3$)$_3$), 86.04 (NHCOCOO*C*(CH$_3$)$_3$), 114.86 (*C*-3), 130.94 (C_q-5), 131.03 (*C*-4), 137.68 (*C*-6), 140.86 (C_q-2), 155.42 (NHCO*C*OOC(CH$_3$)$_3$), 157.67 (NH*C*OCOOC(CH$_3$)$_3$); ^{13}C resonances of complexed 3-chlorobenzoic acid: 128.23 *C*-6′), 129.80 (*C*-2′), 130.20 (*C*-5′), 132.09 (C_q-1′), 133.29 (*C*-4′), 134.59 (C_q-3′), 169.00 (*C*OOH).

***tert*-butyl 2-((5-methylpyridin-2-yl)amino)-2-oxoacetate (S20)**

(RN: none); Mp: < 50 ^{0}C; HRMS: calc. [$C_{12}H_{16}N_2O_3$]: 236.1161; found: 237.1234 $[M+H]^+$.

^{1}H NMR (600.25 MHz, $CDCl_3$, 25 °C): δ = 1.58 (s, 9H, COO(C*H*$_3$)$_3$), 2.30 (s, 3H, C*H*$_3$-5), 7.54 (dd, ^3J = 8.4, ^4J = 2.1, 1H, *H*-4), 8.12 (d, ^3J = 8.4, 1H, *H*-3), 8.14 (m[dq]), 1H, *H*-6), 9.33 (s, sh, 1H, N*H*COCOOC(CH$_3$)$_3$); ^{13}C{^{1}H}NMR (150.93 MHz, $CDCl_3$, 25 °C): δ = 18.02 (5-*C*H$_3$), 27.81 (COOC(*C*H$_3$)$_3$), 85.29 (COO*C*(CH$_3$)$_3$), 113.71 (*C*-3), 130.39 (*C*$_q$-5), 139.13 (*C*-4), 147.94 (*C*$_q$-2), 148.30 (*C*-6), 155.15 (NH*C*OCOOC(CH$_3$)$_3$), 159.12 (NHCO*C*OOC(CH$_3$)$_3$).

5-bromo-2-(2-(*tert*-butoxy)-2-oxoacetamido)pyridine 1-oxide (S21)

(RN: none; 1:1 clathrate with 3-chlorobenzoic acid); Mp: 166-187 °C; HRMS: calc. [$C_{11}H_{13}BrN_2O_4$]: 316.0059; found: 338.9951 $(M+Na)^+$.

Parent compound **S21**: ^{1}H NMR (600.25 MHz, DMSO-d$_6$, 25 °C): δ = 1.61 (s, 9H, NHCOCOOC(C*H*$_3$)$_3$), 7.59 (dd, ^3J = 9.1, ^4J = 1.8, 1H, *H*-4), 8.40 (d, ^3J = 9.1, 1H, *H*-3), 8.58 (d, ^4J = 1.8, 1H, *H*-6), 11.09 (s, br, 1H, N*H*COCOOC(CH$_3$)$_3$); ^{1}H resonances of complexed 3-chlorobenzoic acid: δ = 7.41 (t, $^3J_1 = {}^3J_2$ = 7.9, 1H, *H′*-5), 7.56 (m[dt]), ^3J = 7.8, $^4J_1$ = 1.8, $^4J_2$ = 0.9, 1H, *H′*-4), 7.98 (dt, ^3J = 7.7, $^4J_1 = {}^4J_2$ = 1.2, 1H, *H′*-6), 8.07 (m[t]), $^4J_1$ = 2.0, $^4J_2$ = 1.0, 1H, *H′*-2), 9.2-10.0 (br, 1H, COO*H*);

Parent compound **S21**: ^{13}C{^{1}H}NMR (150.93 MHz, DMSO-d$_6$, 25 °C): δ = 27.80 (NHCOCOOC(*C*H$_3$)$_3$), 86.47 (NHCOCOO*C*(CH$_3$)$_3$), 113.51 (*C*$_q$-5), 115.41 (*C*-3), 132.62 (*C*-4), 139.06 (*C*-6), 142.40 (*C*$_q$-2), 155.46 (NH*C*OCOOC(CH$_3$)$_3$), 157.40 (NHCO*C*OOC(CH$_3$)$_3$); ^{13}C resonances of complexed 3-chlorobenzoic acid: δ = 128.36 (*C*-6′), 129.91 (*C*-2′), 130.31 (*C*-5′), 131.46 (*C*$_q$-1′), 133.71 (*C*-4′), 134.73 (*C*$_q$-3′), 169.87 (*C*OOH).

2-(2-(*tert*-butoxy)-2-oxoacetamido)-6-(methoxycarbonyl)pyridine 1-oxide (S22)

(RN: none); Mp: oil; HRMS: calc. [$C_{13}H_{16}N_2O_6$]: 296.1008; found: 319.0901 $(M+Na)^+$.

^{1}H NMR (600.25 MHz, $CDCl_3$, 25 °C): δ = 1.58 (s, 9H, NHCOCOOC(C*H*$_3$)$_3$), 3.99 (s, 3H, 6-COOC*H*$_3$), 7.38-7.43 (m, 2H, AB-part of ABX, *H*-4,5), 8.49-8.56 (m, 1H, X-part of ABX, *H*-3), 11.29 (s, br, N*H*COCOOC(CH$_3$)$_3$); ^{13}C{^{1}H}NMR (150.93 MHz, $CDCl_3$, 25 °C): δ = 27.75 (NHCOCOOC(*C*H$_3$)$_3$), 53.63 (6-COO*C*H$_3$), 86.23 (NHCOCOO*C*(CH$_3$)$_3$), 116.48 (*C*-3), 120.79 (*C*-5), 127.38 (*C*-4), 139.79 (*C*$_q$-6), 144.12 (*C*$_q$-2), 155.58 (NH*C*OCOOC(CH$_3$)$_3$), 157.01 (NHCO*C*OOC(CH$_3$)$_3$), 161.46 (6-*C*OOCH$_3$).

- **Graphical representation of NMR-spectra**

2-(2-methoxy-2-oxoacetamido)benzoic acid (1a)

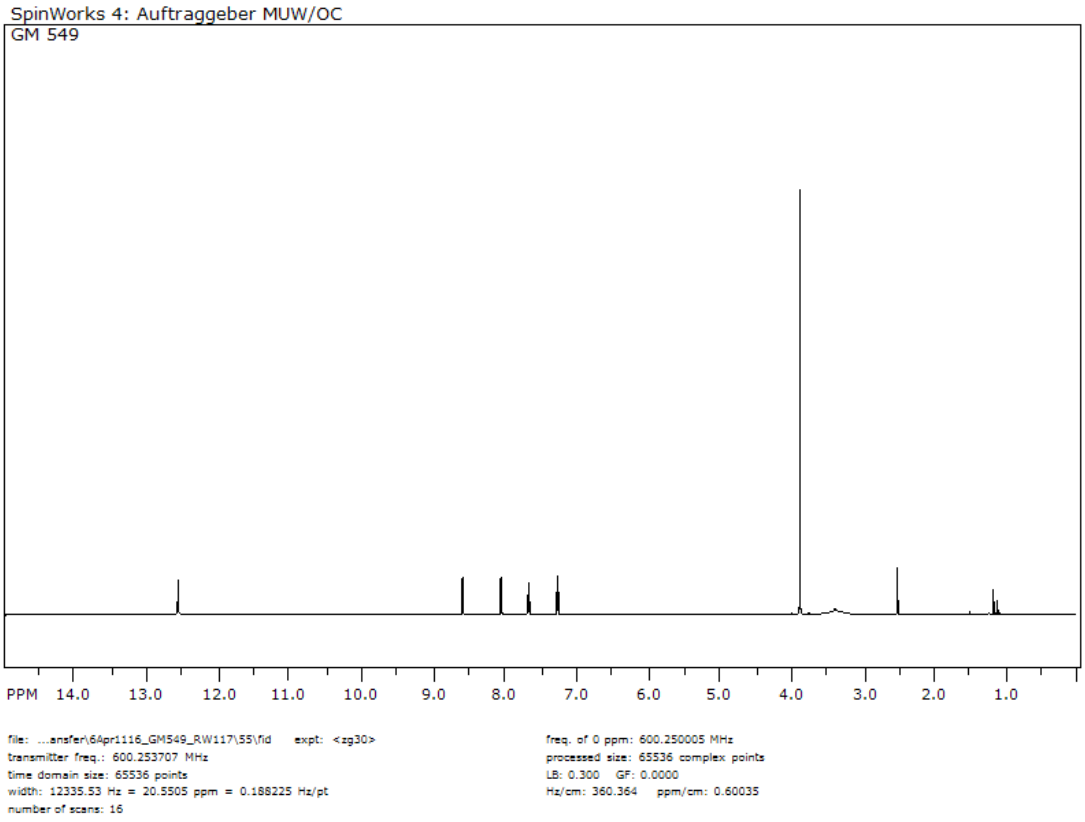

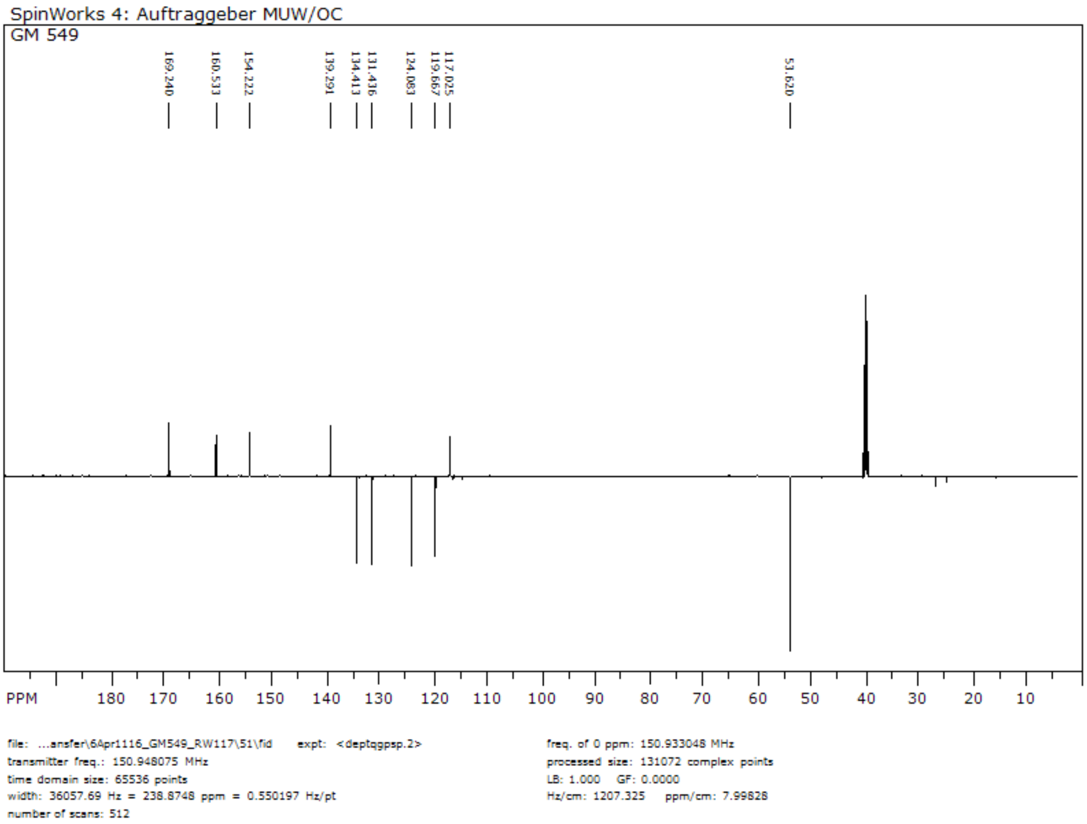

2-(carboxyformamido)benzoic acid (1b)

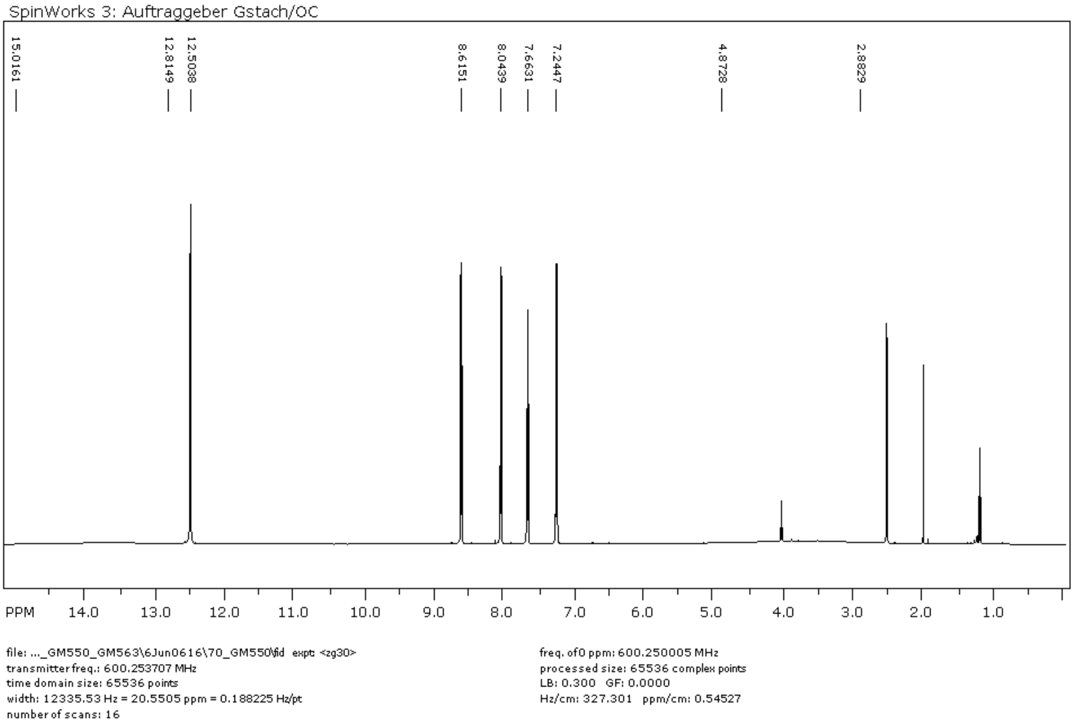

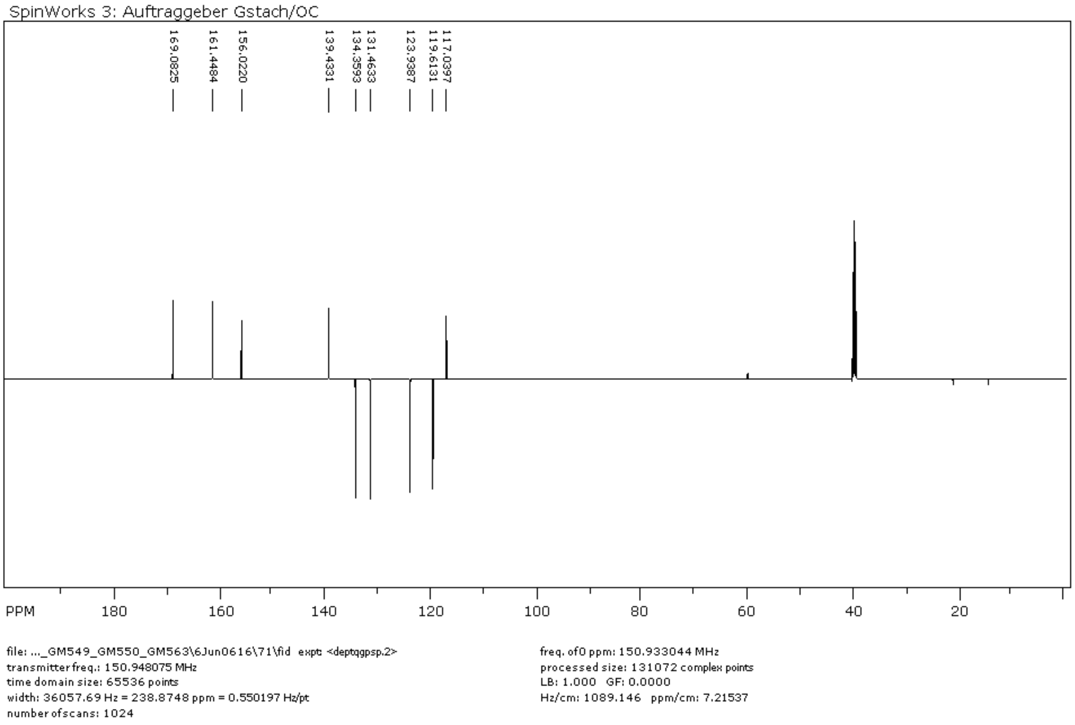

2-(2-(2-methoxy-2-oxoacetamido)benzamido)benzoic acid (1d)

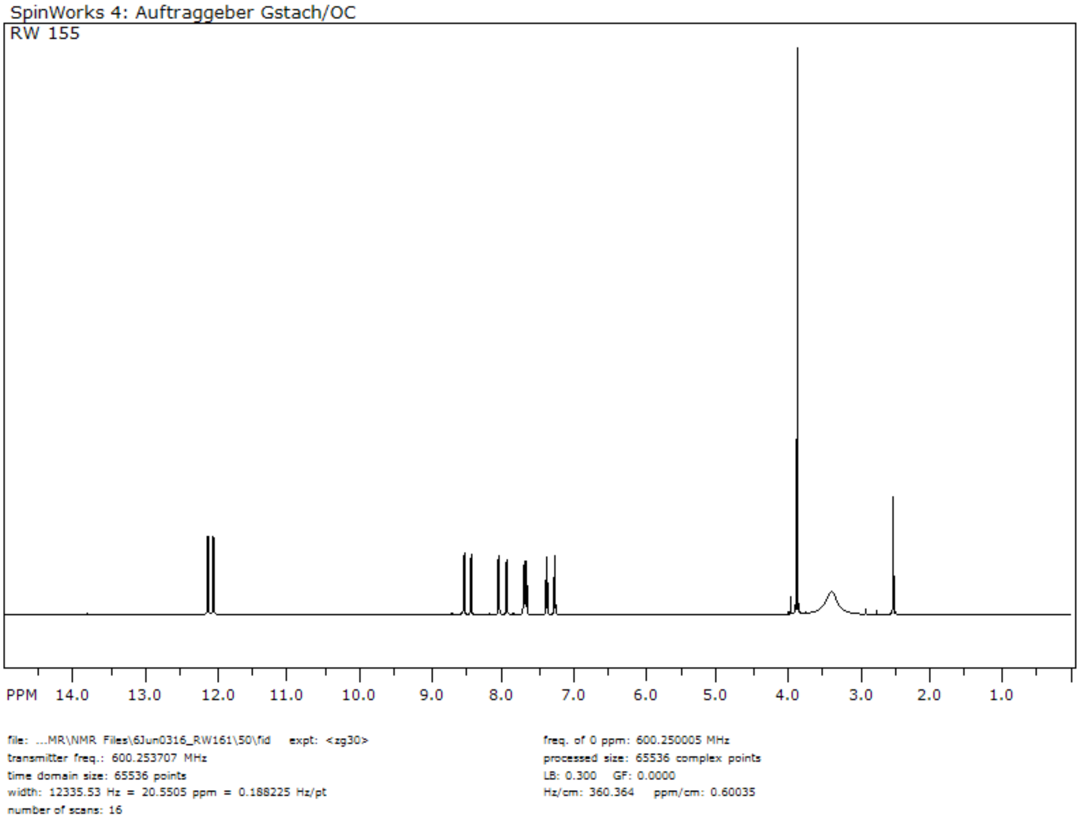

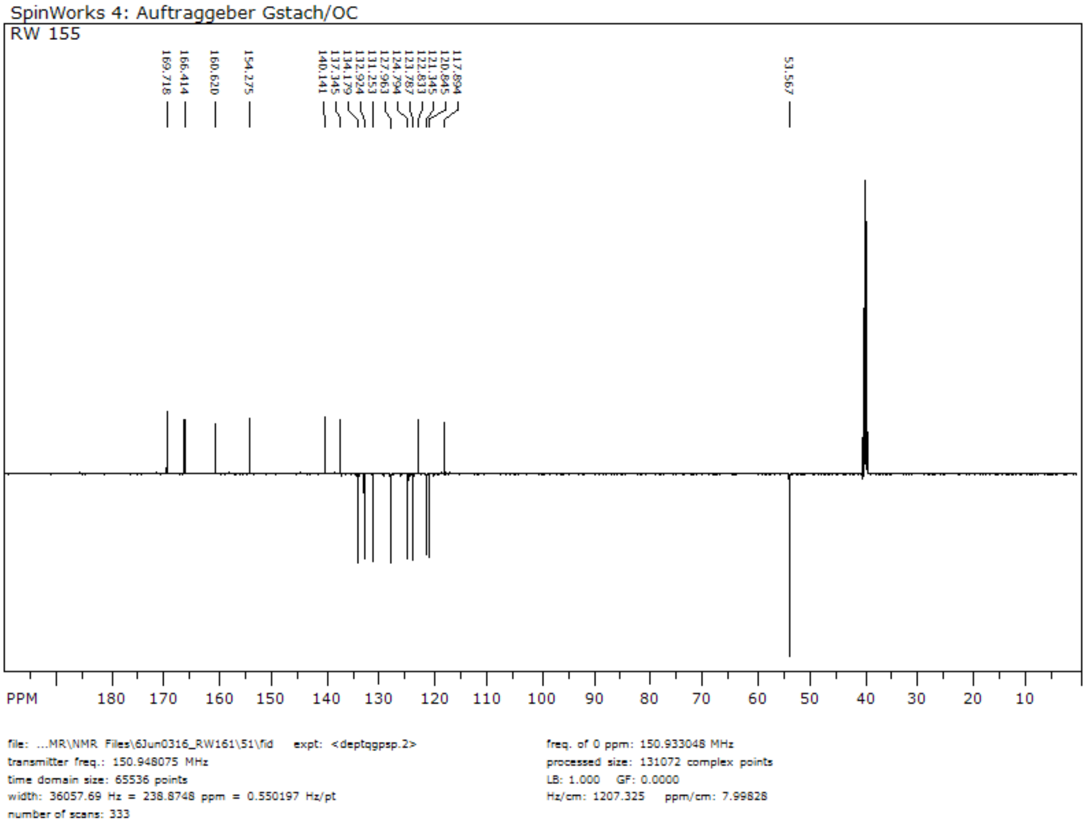

2-(2-(carboxyformamido)benzamido)benzoic acid (1e)

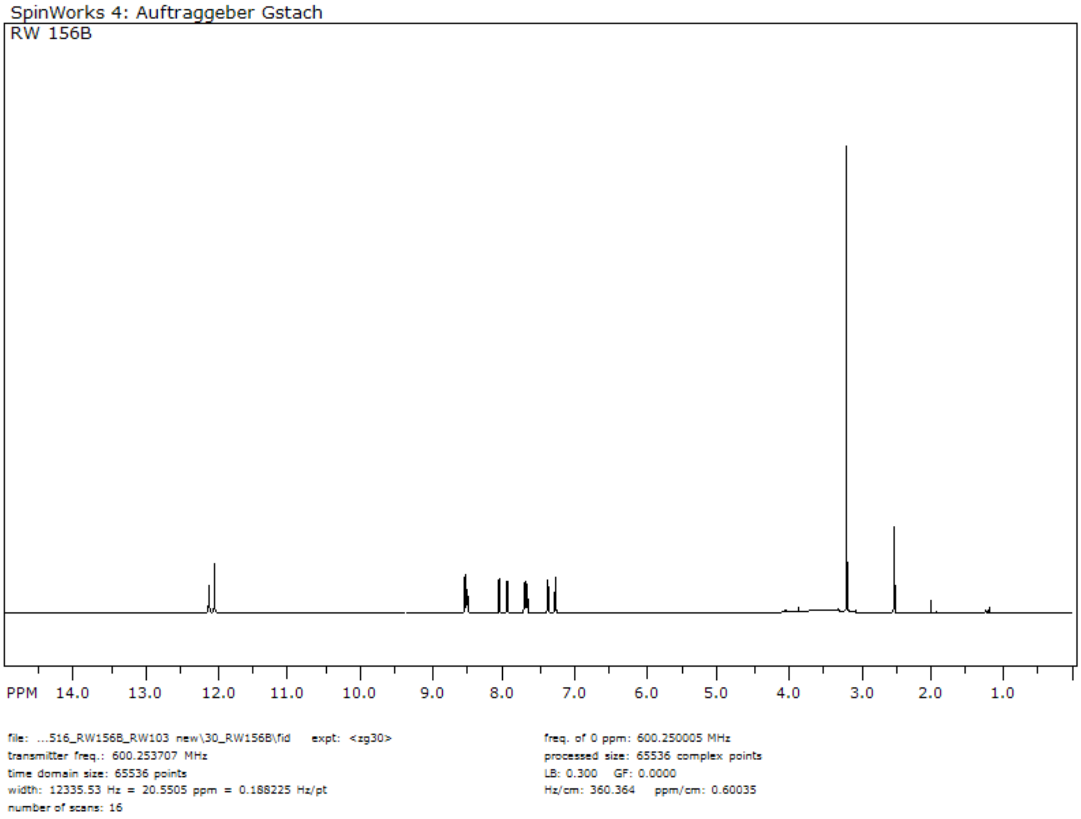

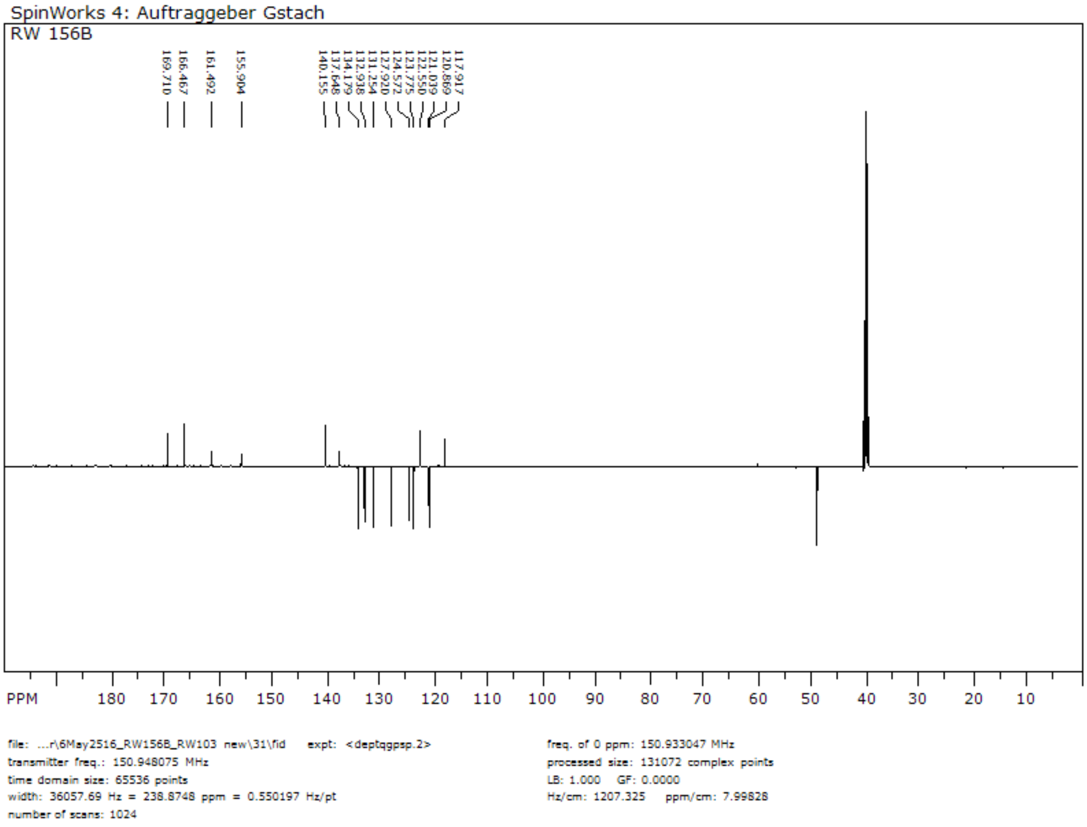

3-(2-methoxy-2-oxoacetamido)benzoic acid (2a)

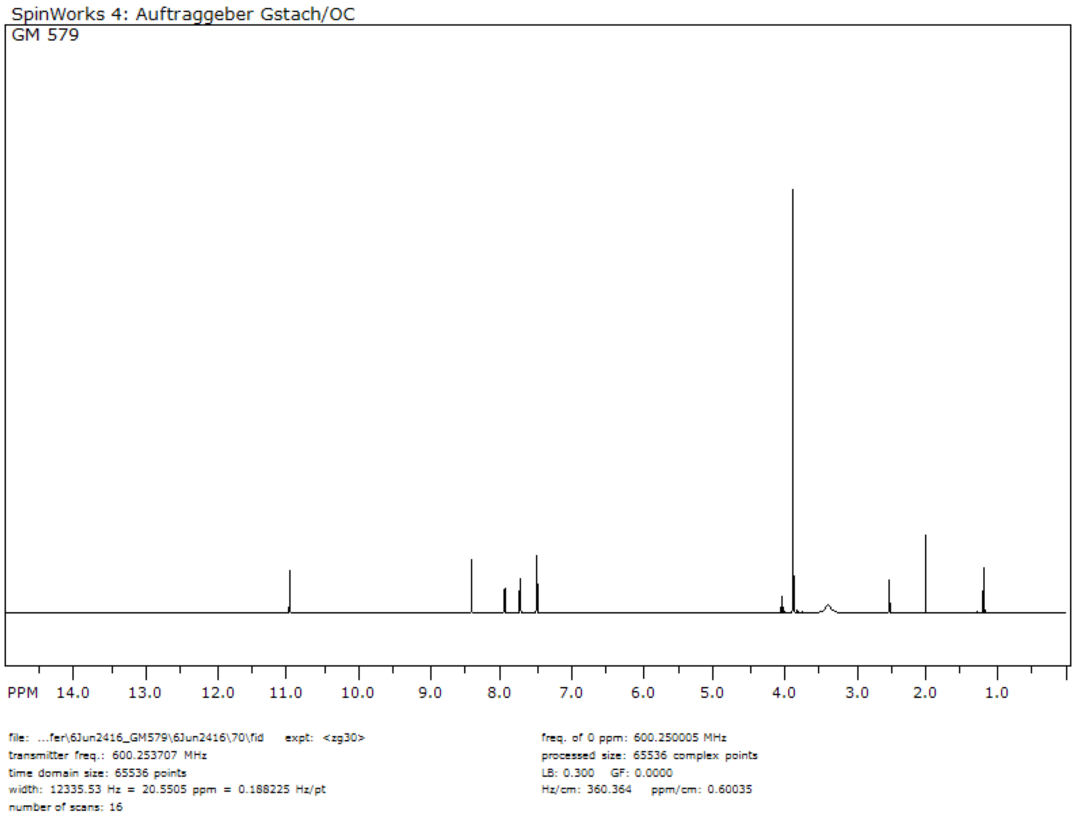

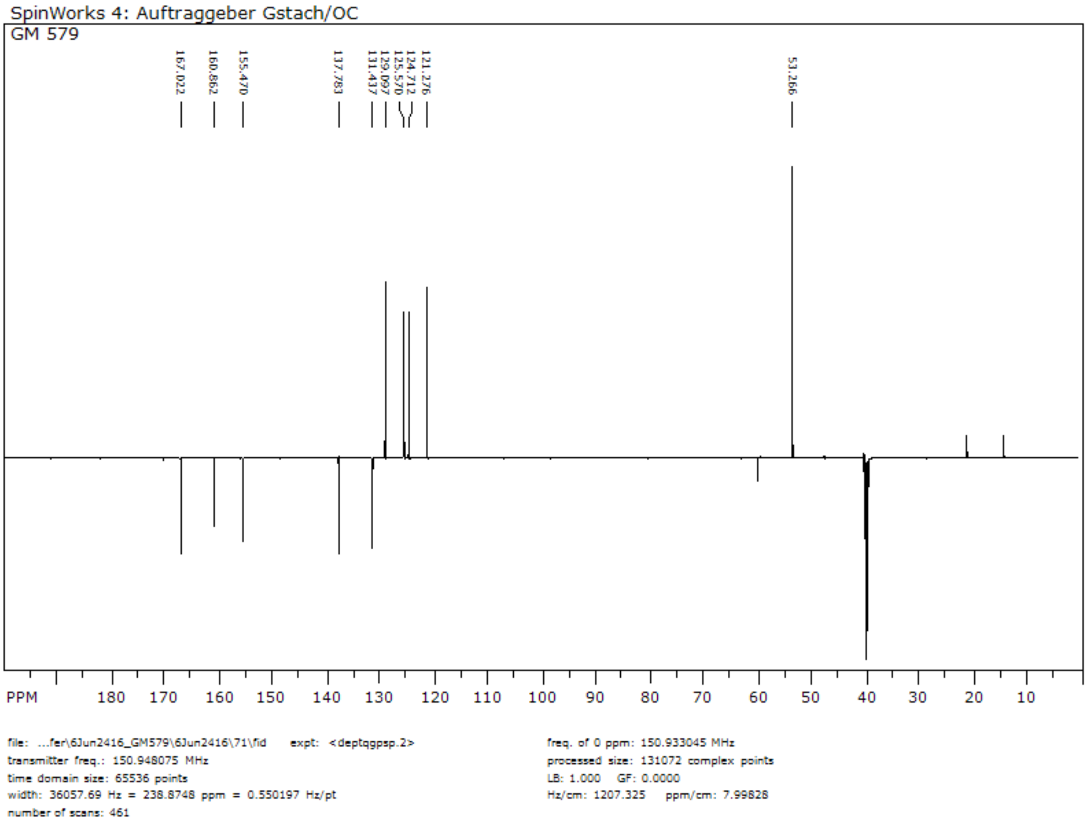

3-(Carboxyformamido)benzoic acid (2b)

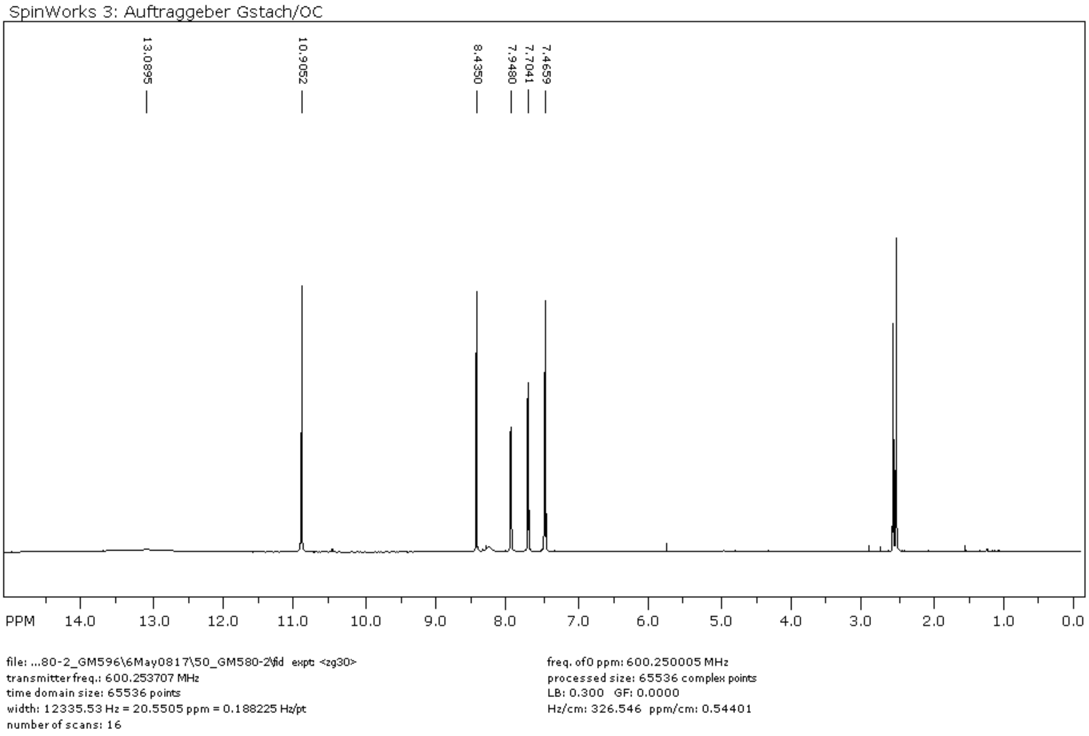

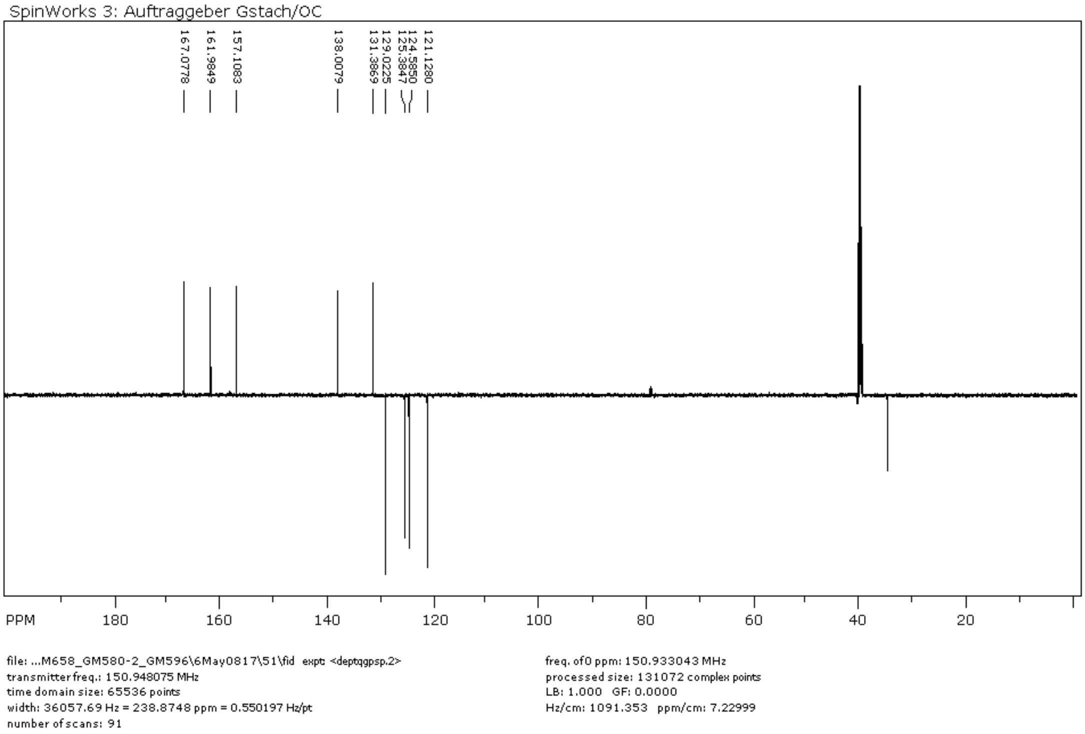

4-(2-methoxy-2-oxoacetamido)benzoic acid (3a)

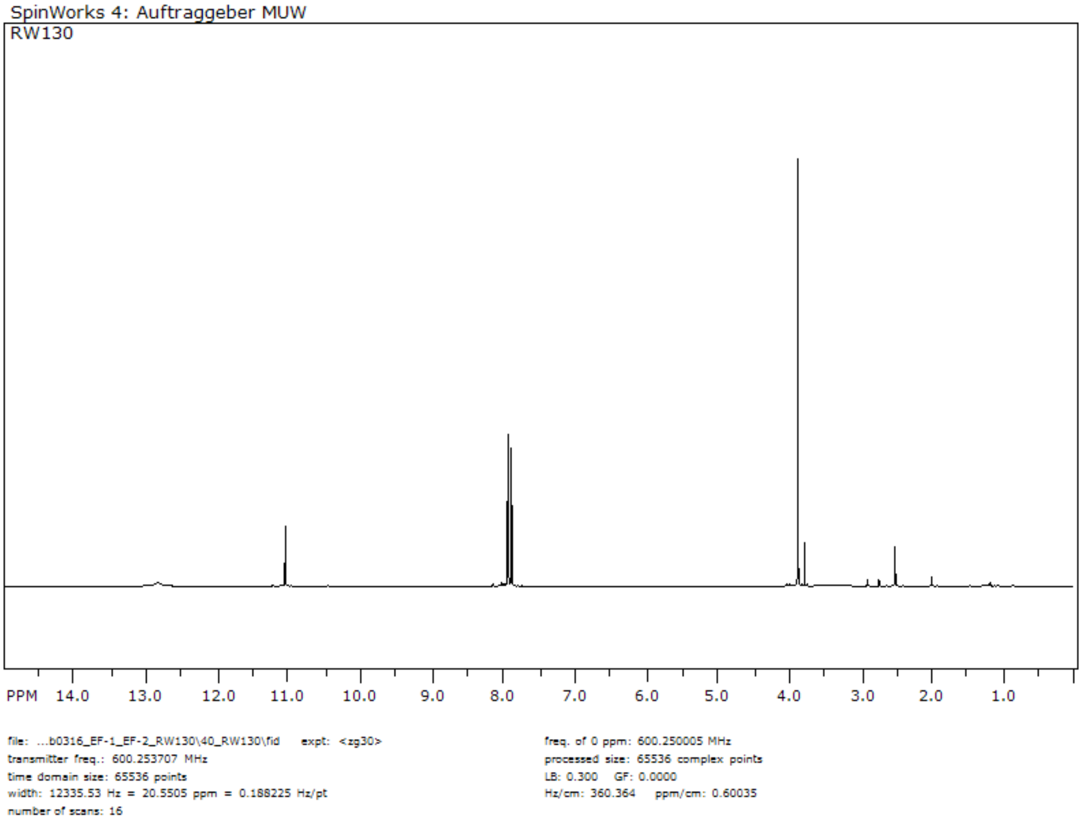

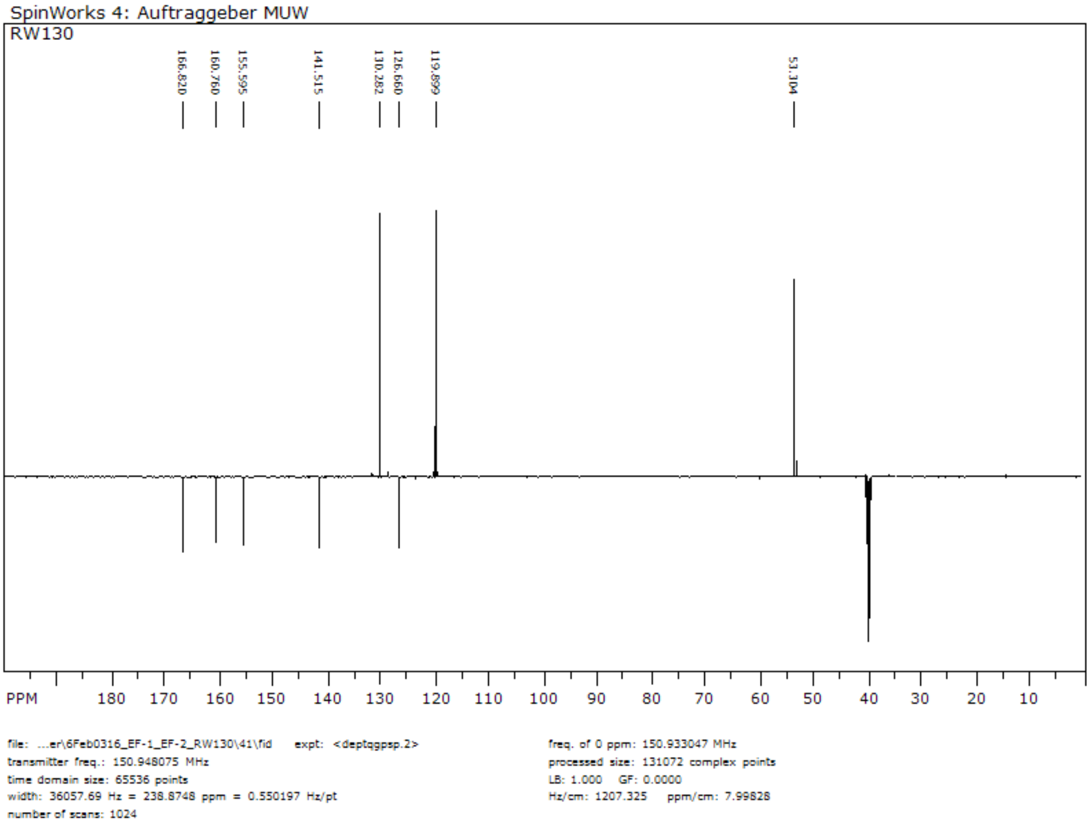

4-(carboxyformamido)benzoic acid (3b)

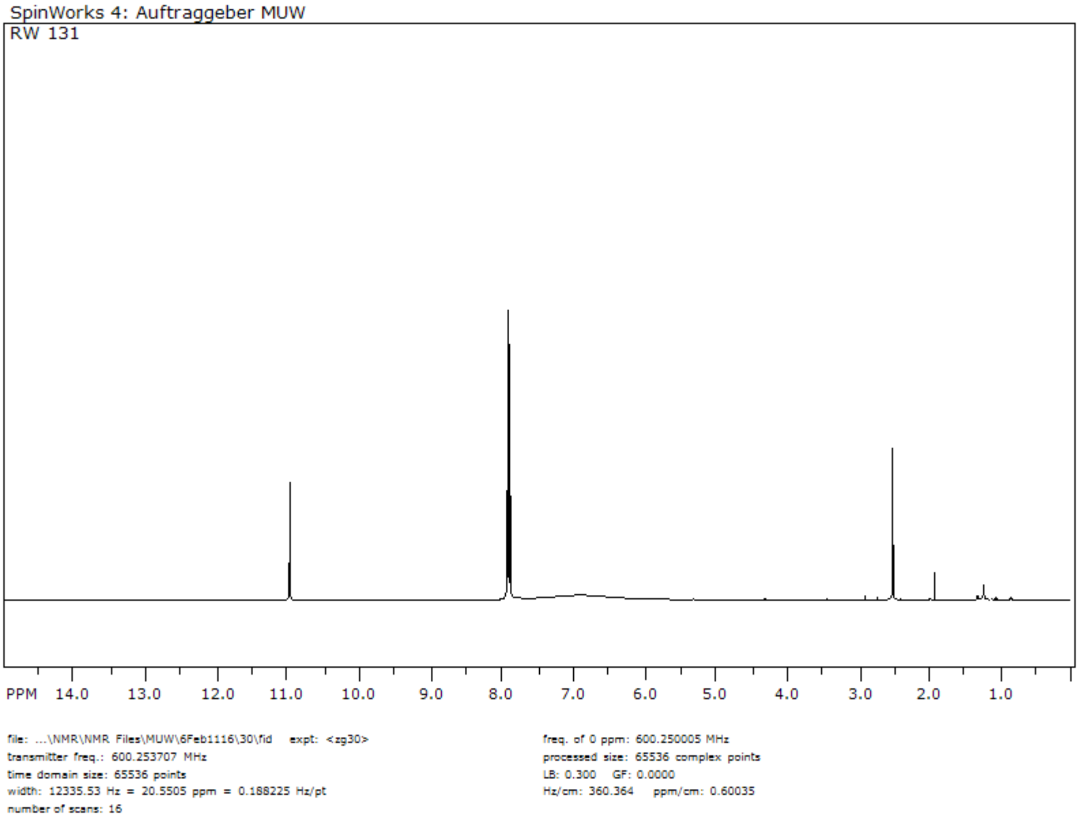

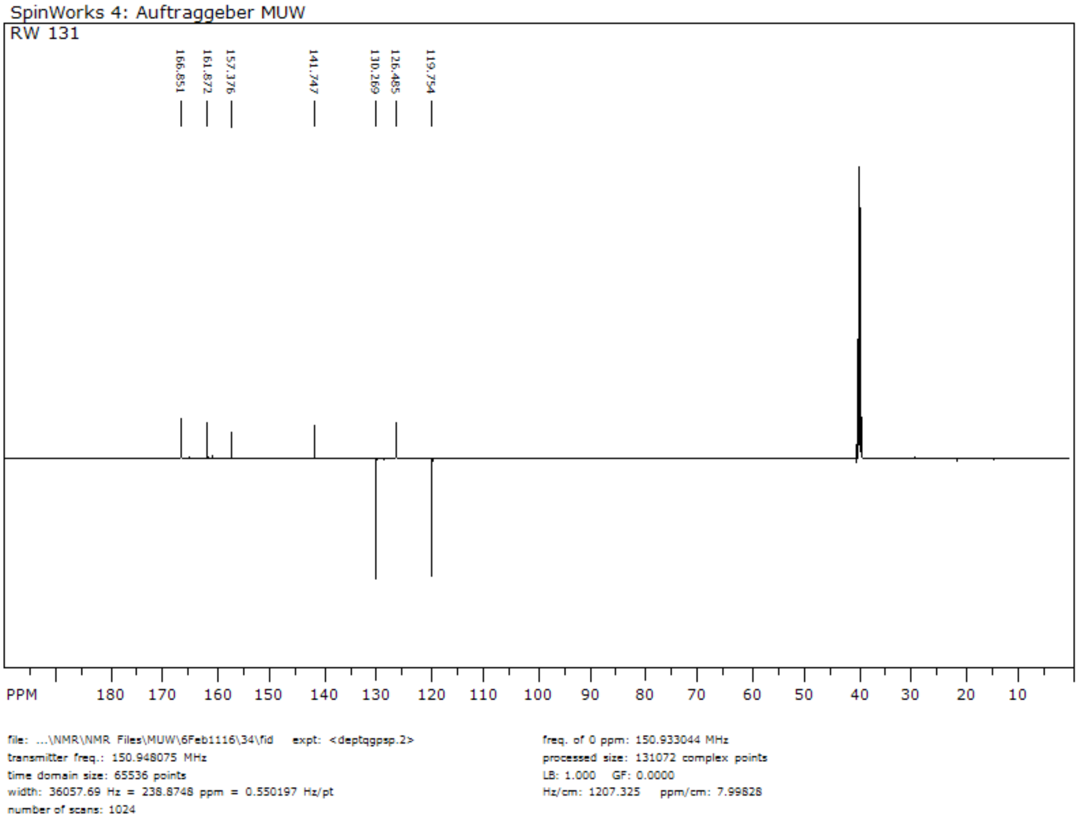

Methyl 2-((2-nitrophenyl)amino)-2-oxoacetate (4a)

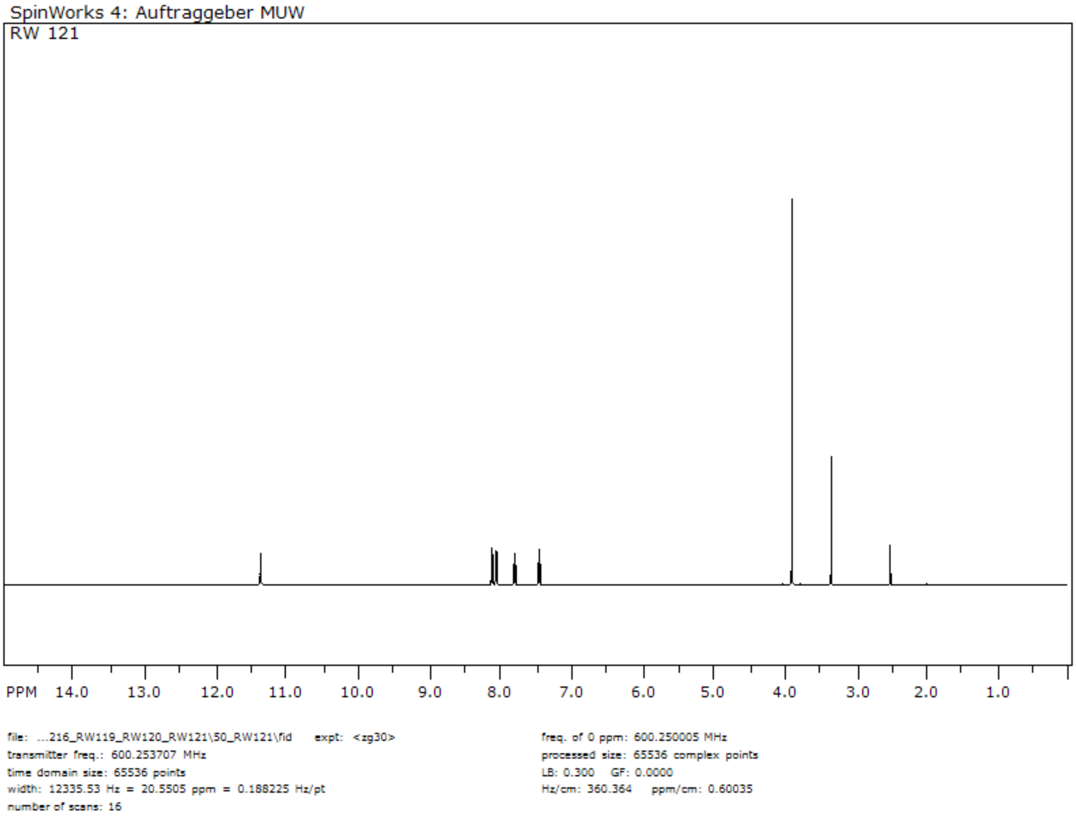

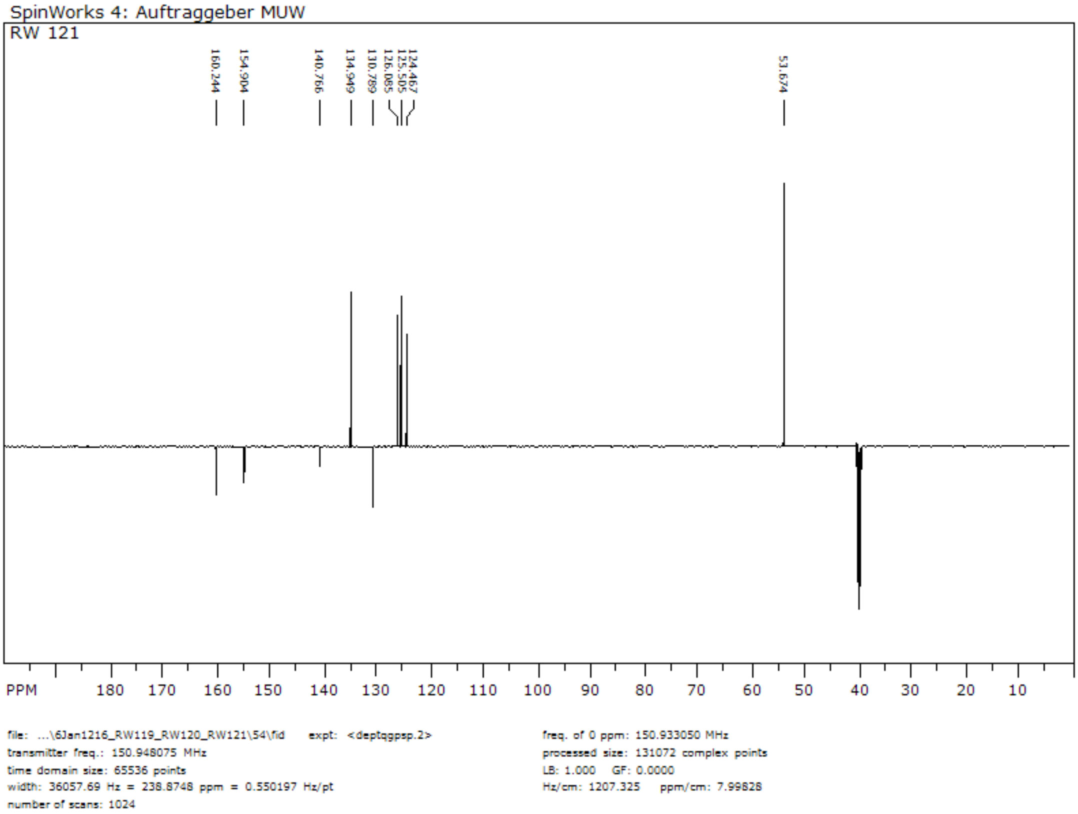

2-((2-nitrophenyl)amino)-2-oxoacetic acid (4b)

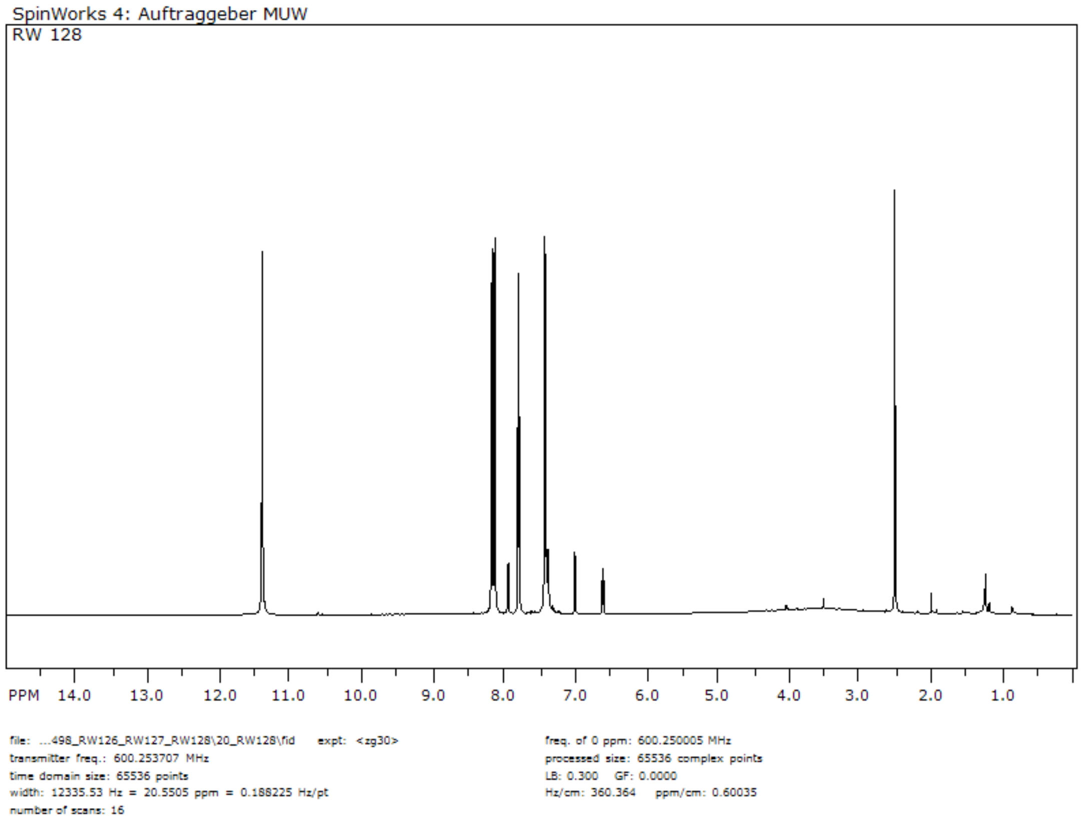

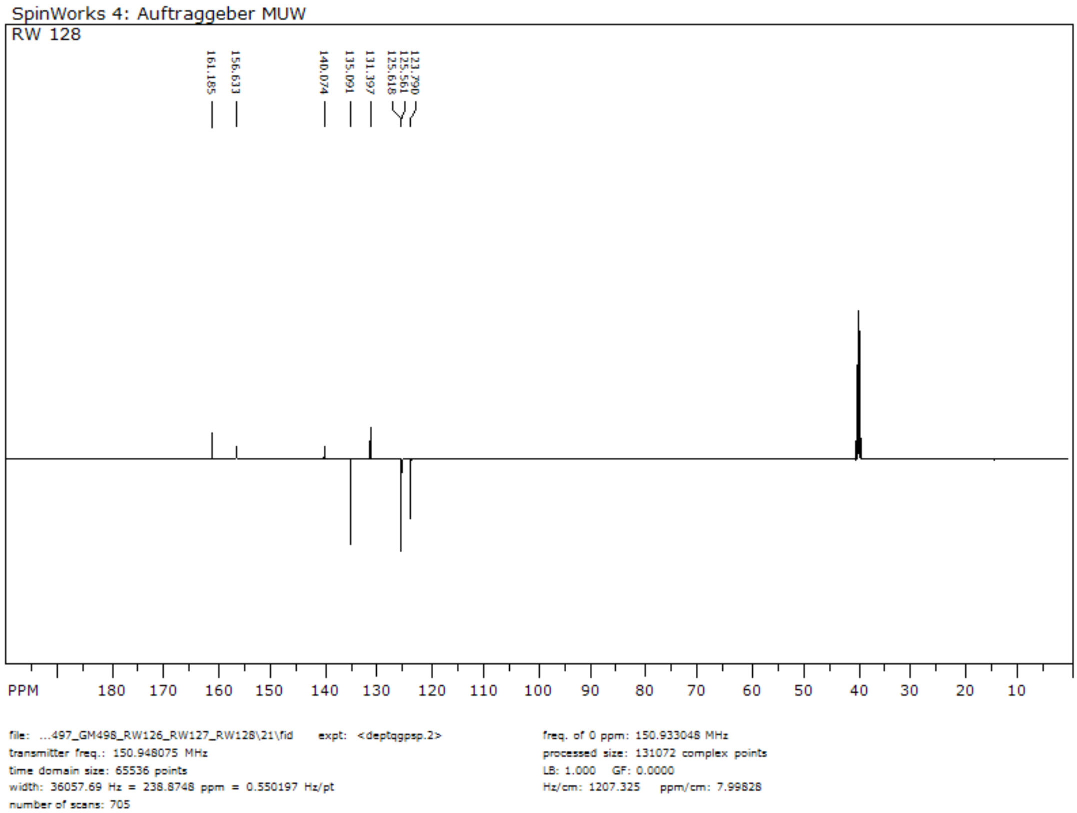

Methyl 2-((3-nitrophenyl)amino)-2-oxoacetate (5a)

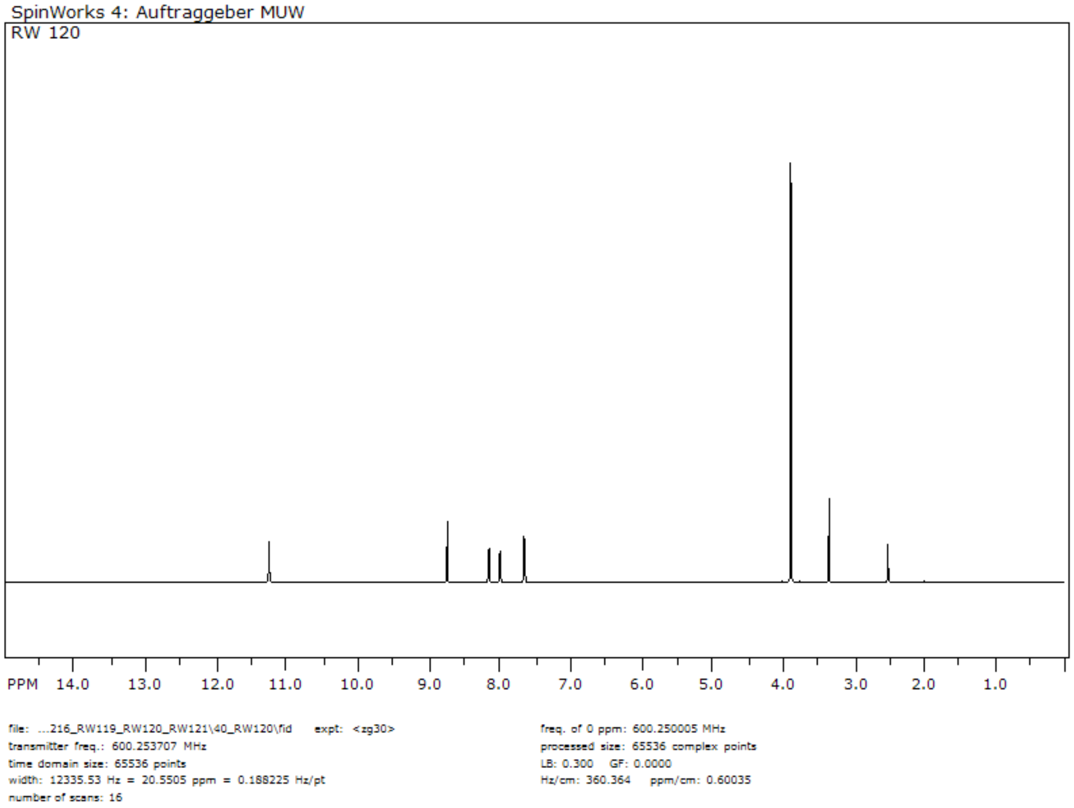

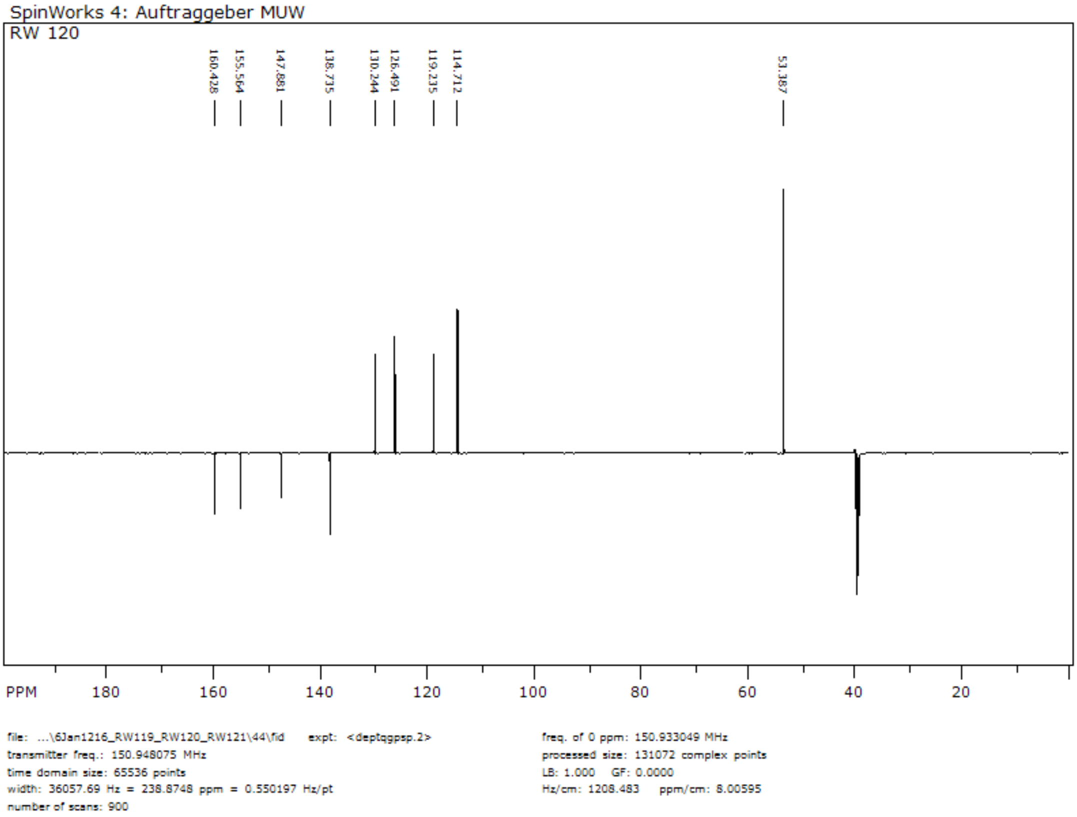

2-((3-nitrophenyl)amino)-2-oxoacetic acid (5b)

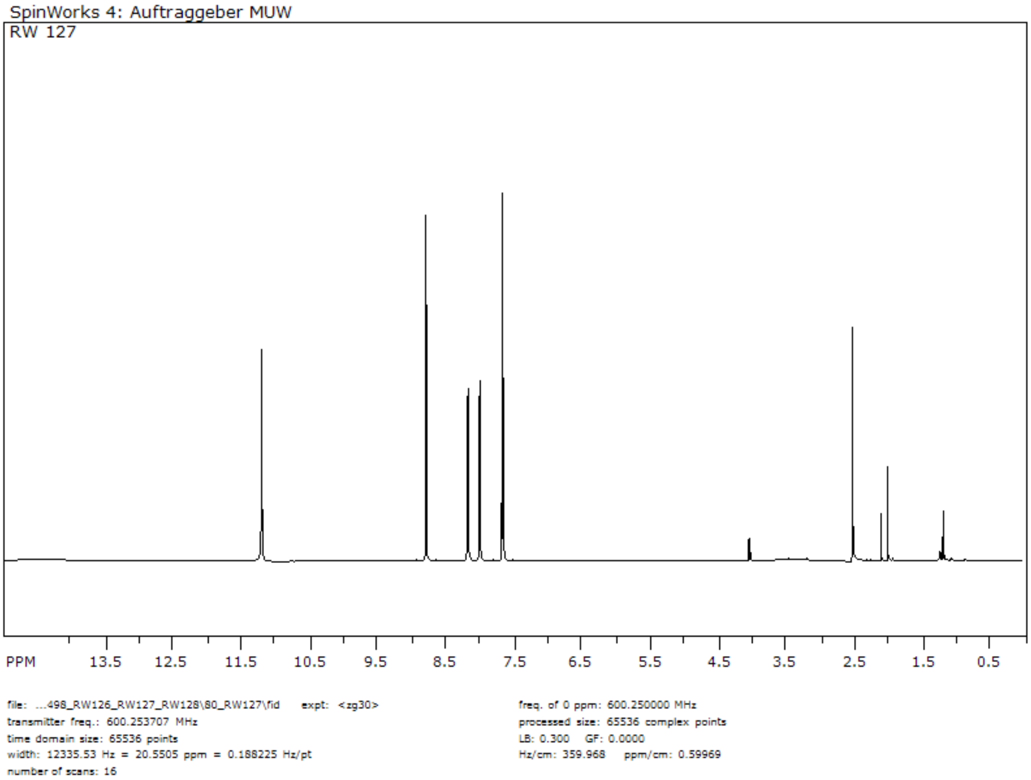

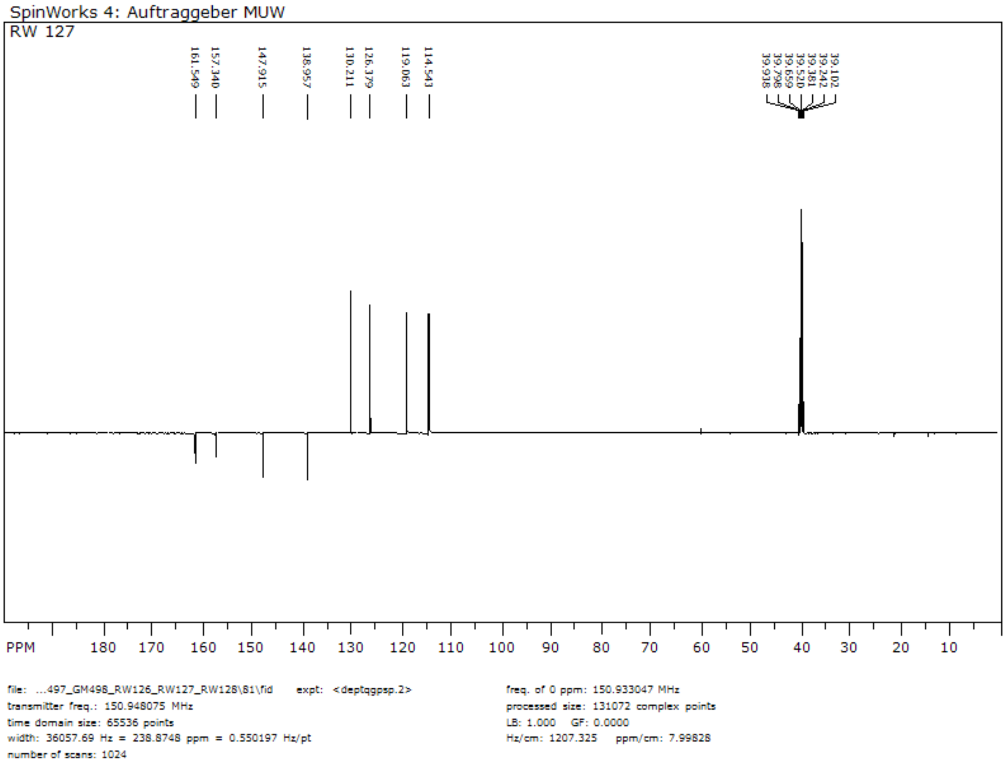

Methyl 2-((4-nitrophenyl)amino)-2-oxoacetate (6a)

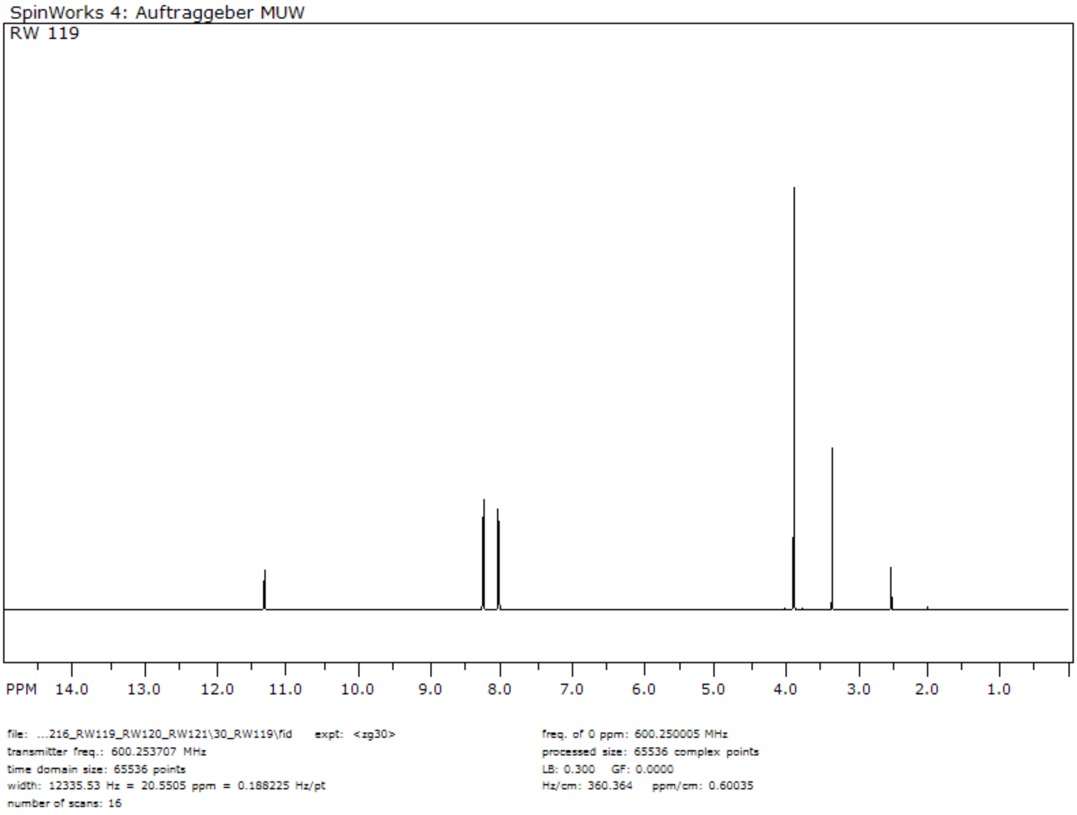

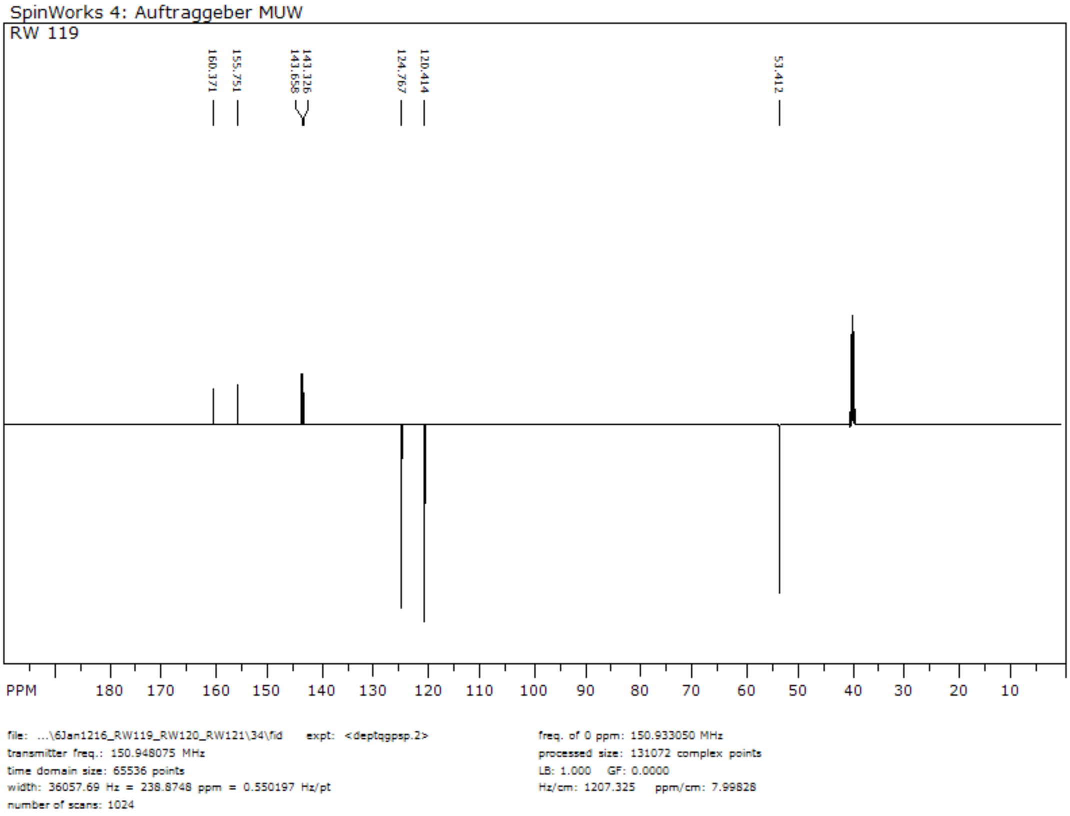

2-((4-nitrophenyl)amino)-2-oxoacetic acid (6b)

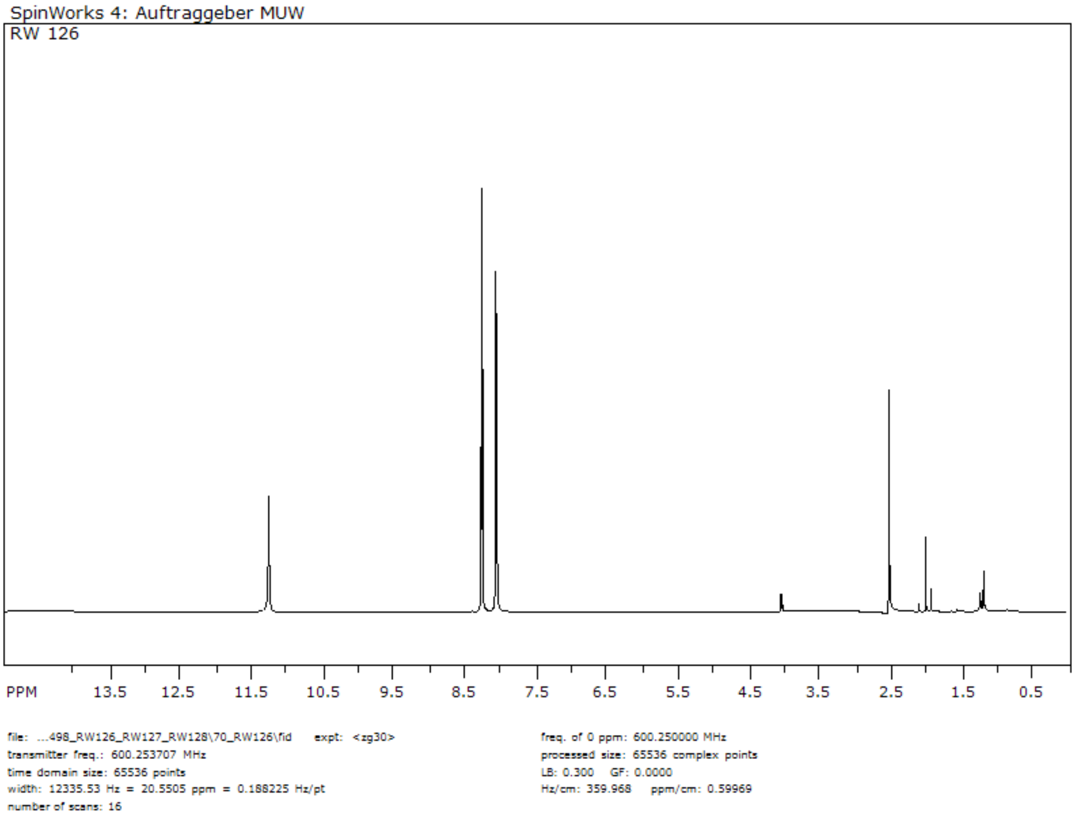

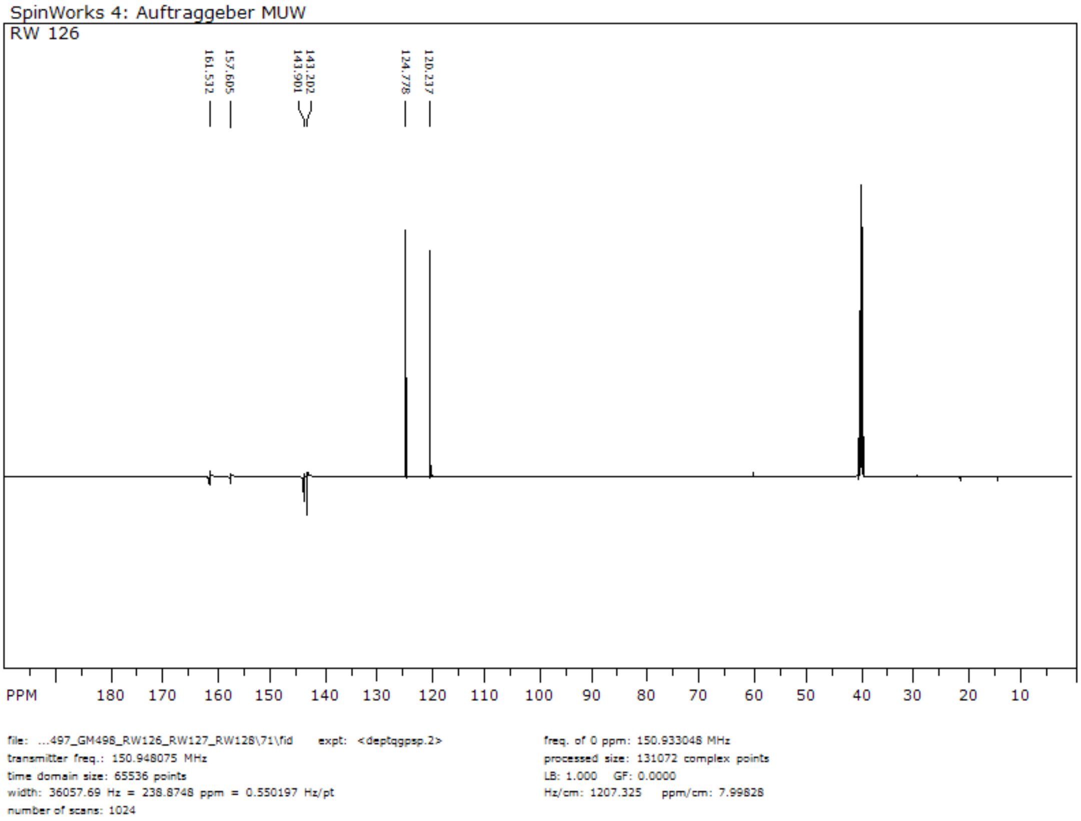

Methyl 2-(naphthalen-2-ylamino)-2-oxoacetate (7a)

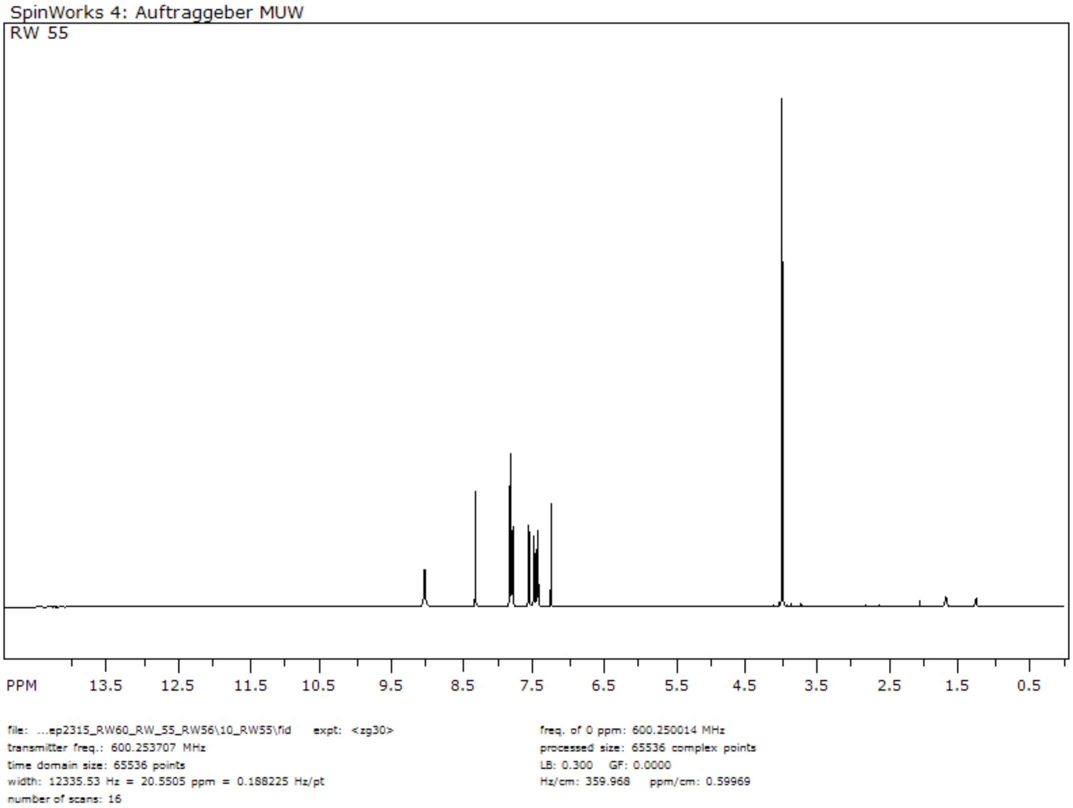

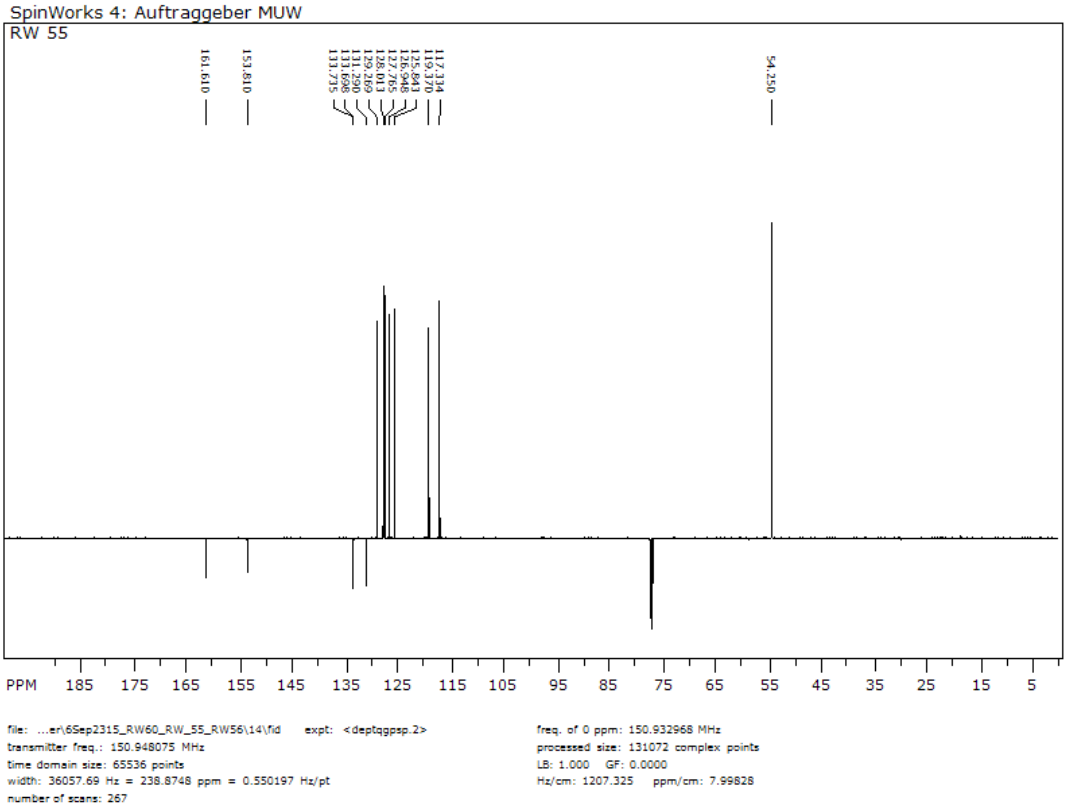

2-(naphthalen-2-ylamino)-2-oxoacetic acid (7b)

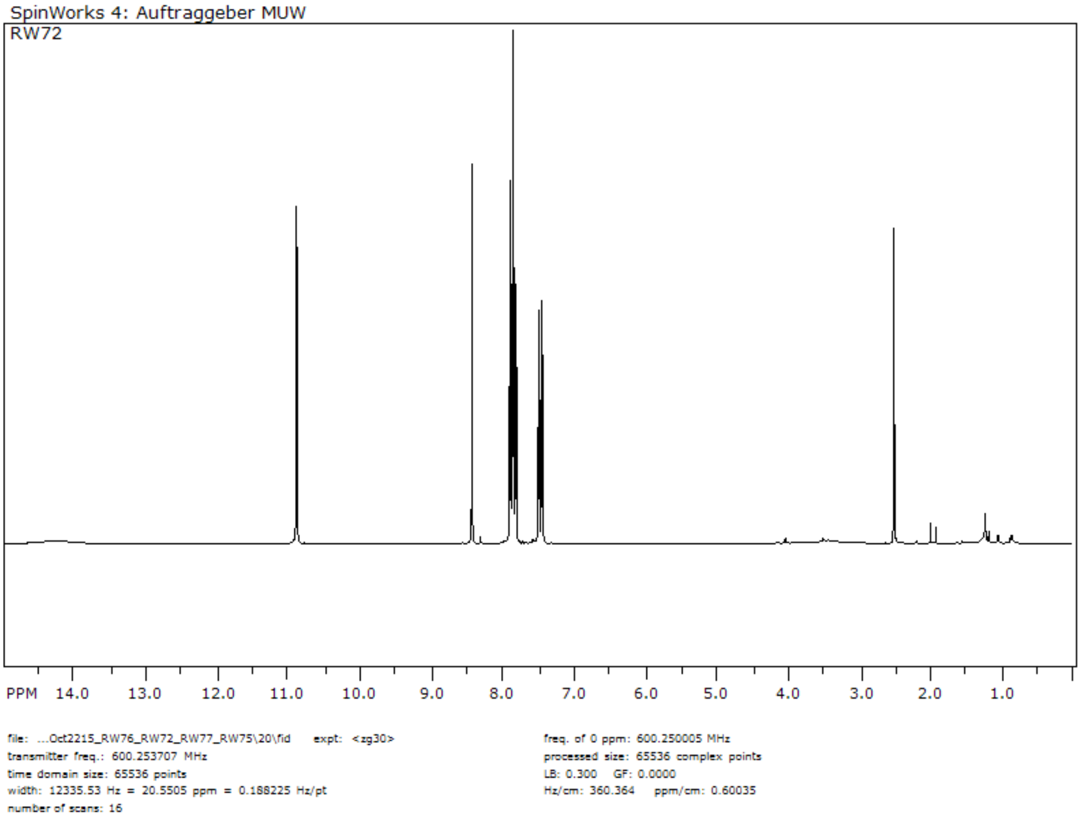

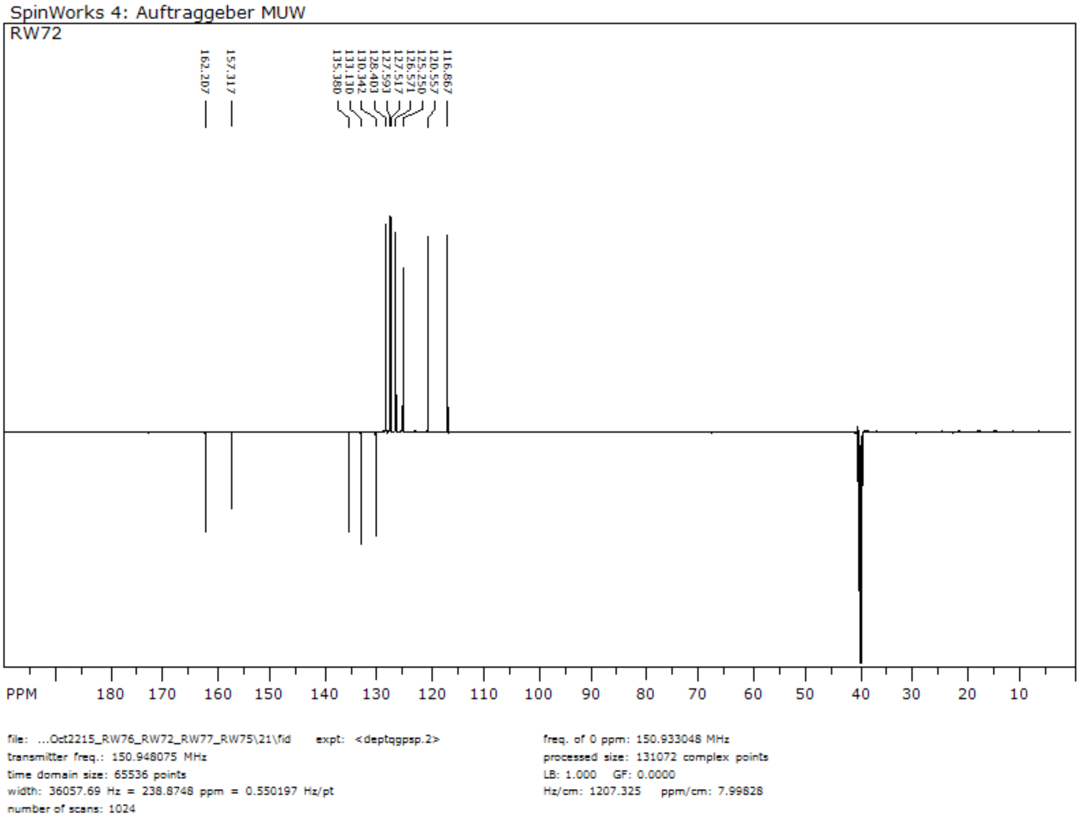

Methyl 2-(naphthalen-1-ylamino)-2-oxoacetate (8a)

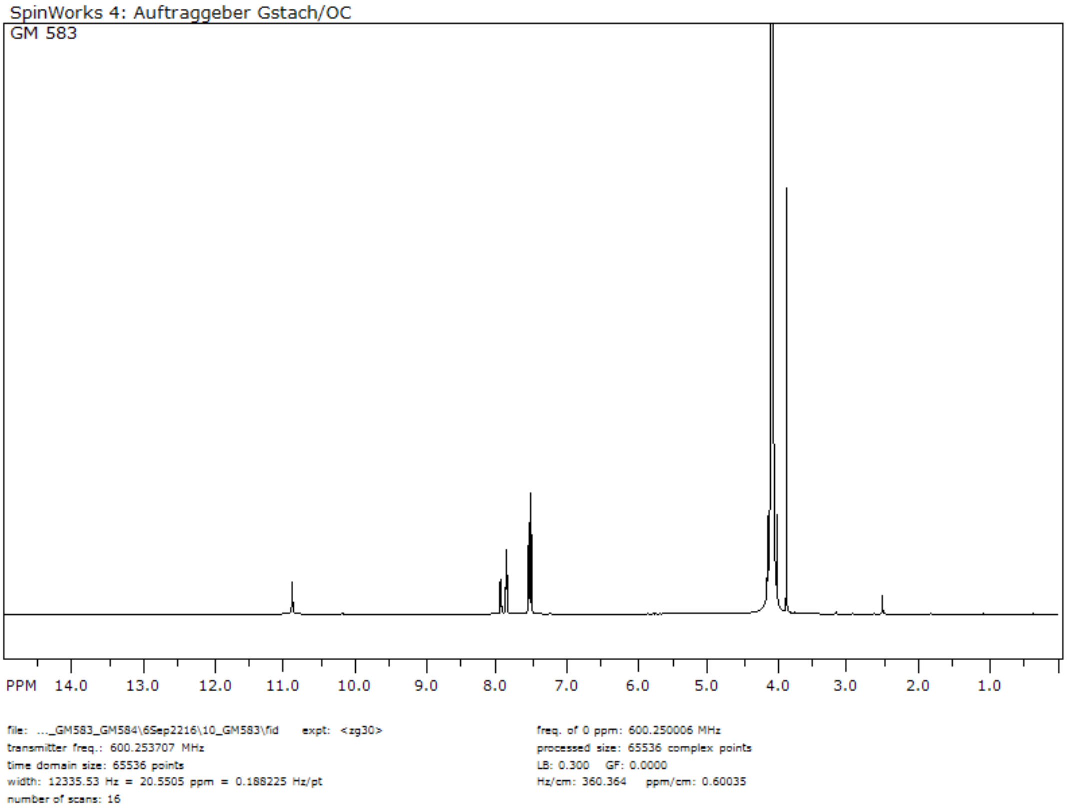

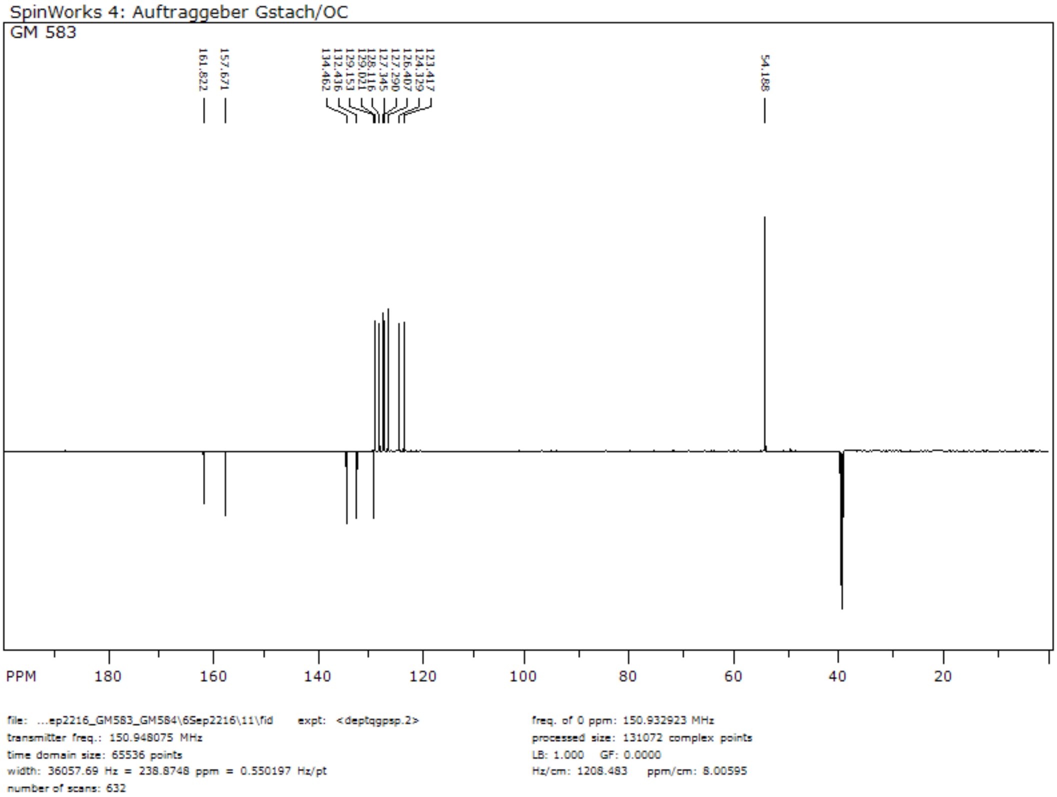

2-(naphthalen-1-ylamino)-2-oxoacetic acid (8b)

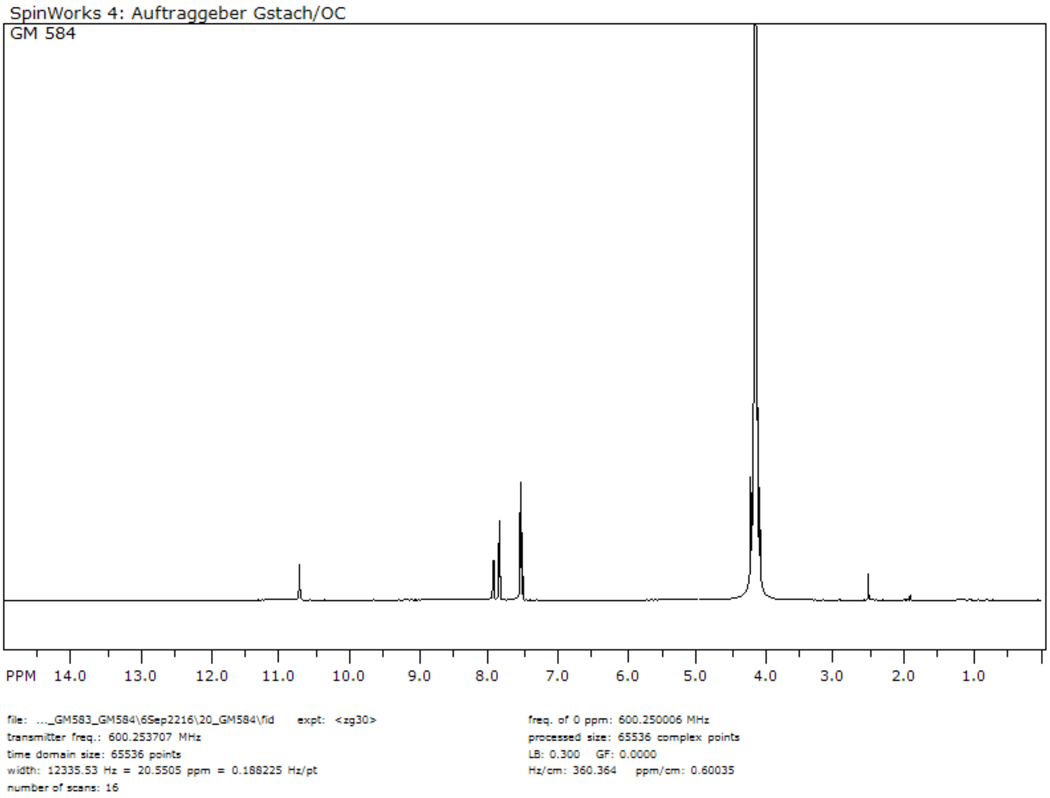

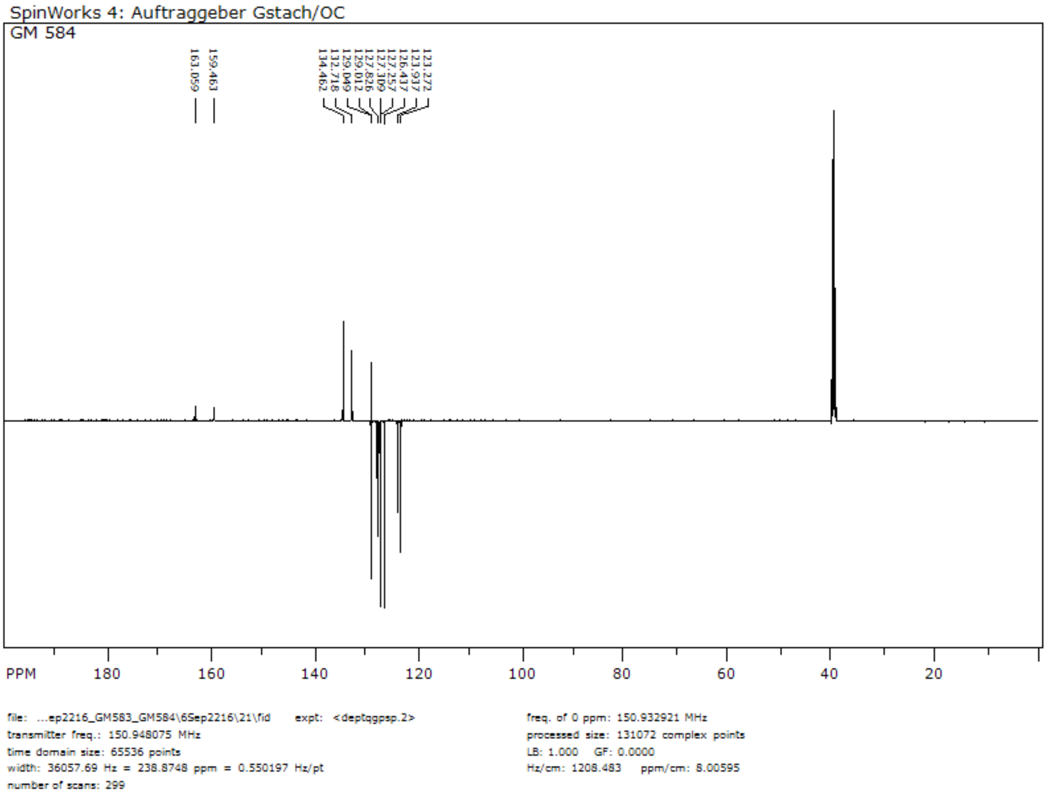

Methyl 2-oxo-2-(pyridin-3-ylamino)acetate (9a)

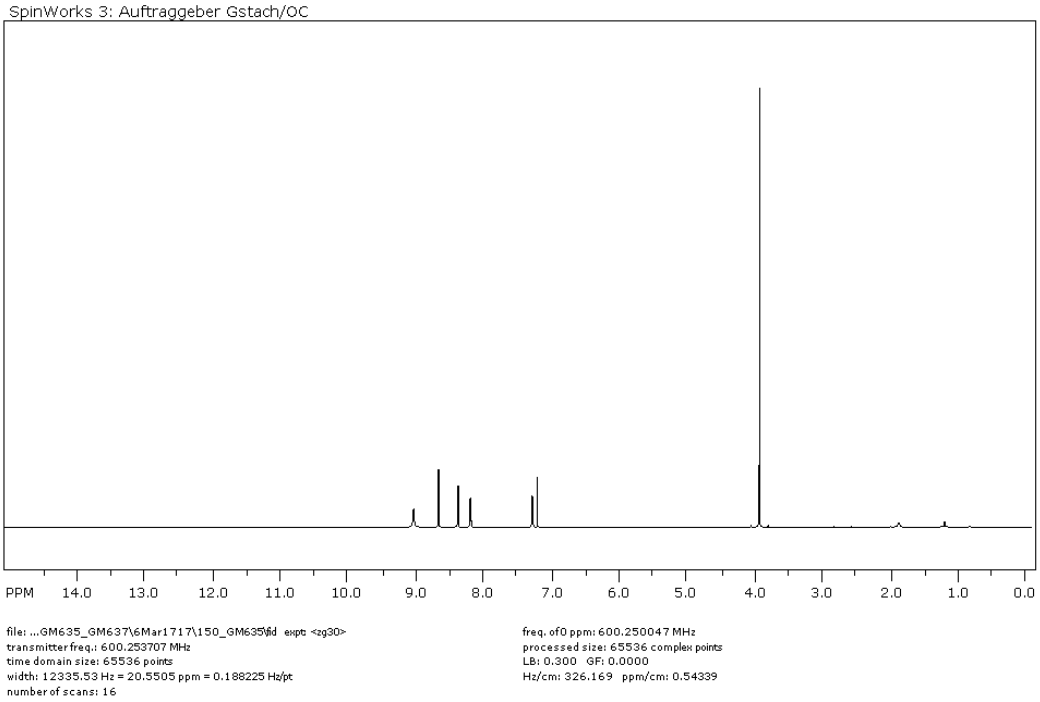

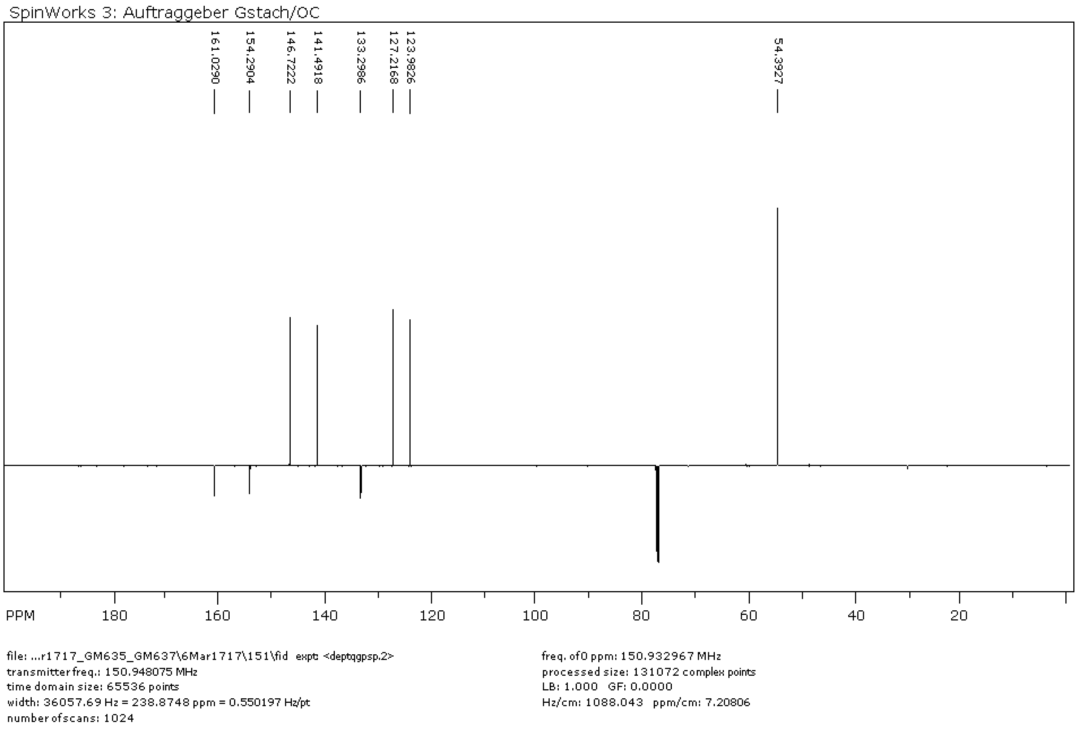

3-(2-Methoxy-2-oxoacetamido)pyridine 1-oxide (11a)

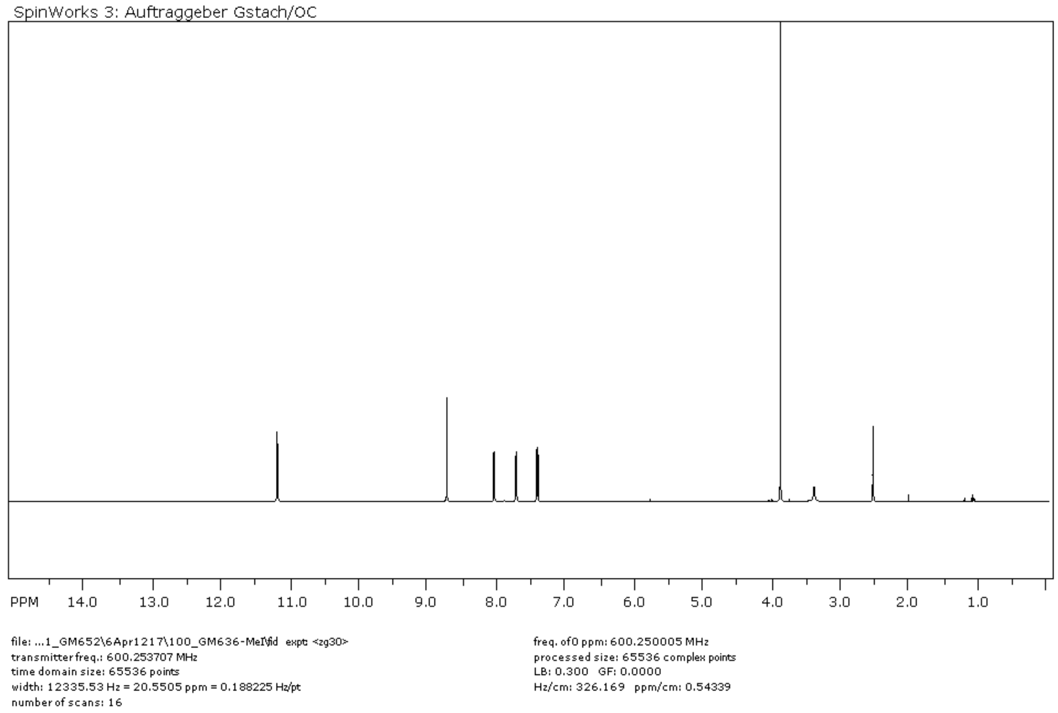

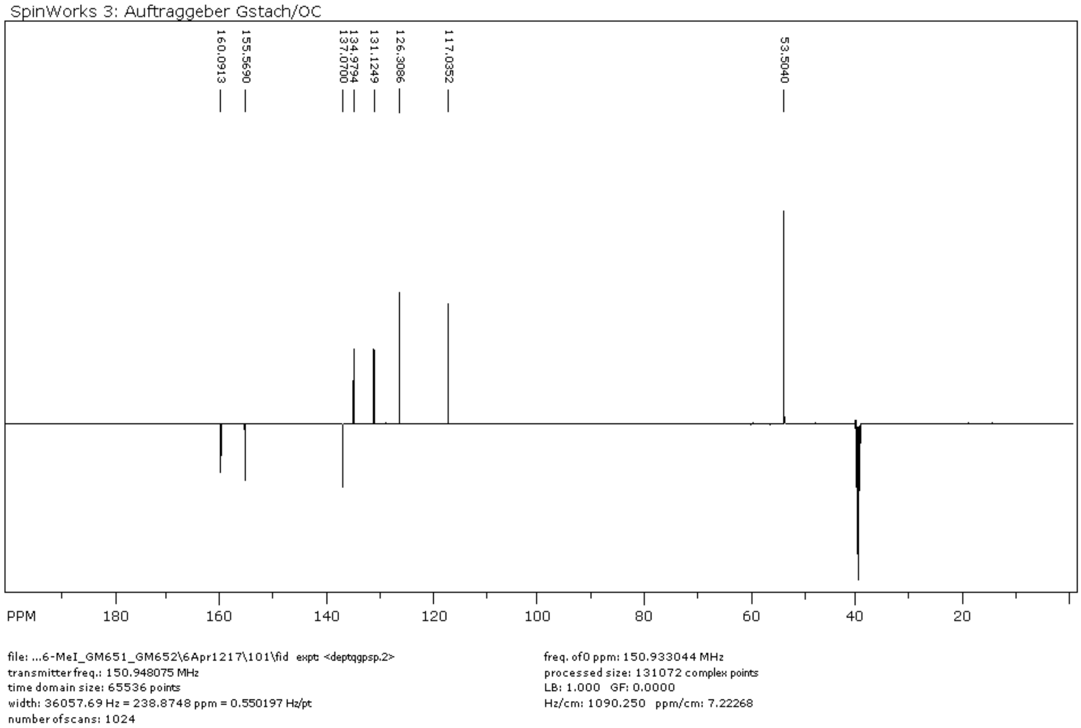

3-(Carboxyformamido)pyridine 1-oxide (11b)

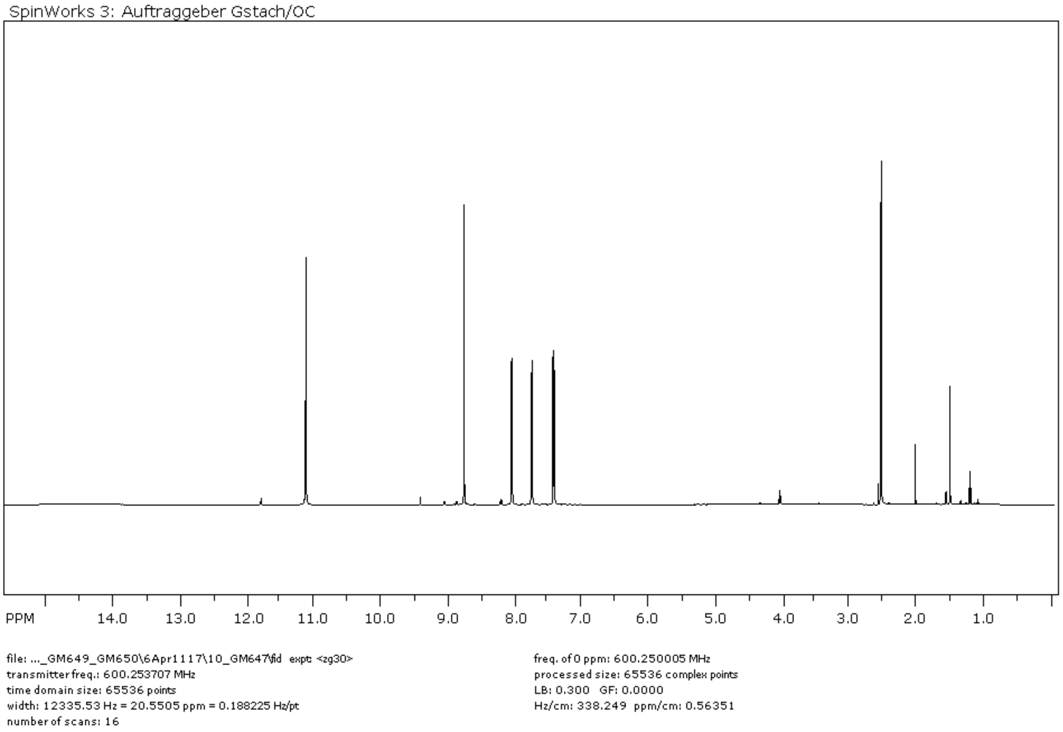

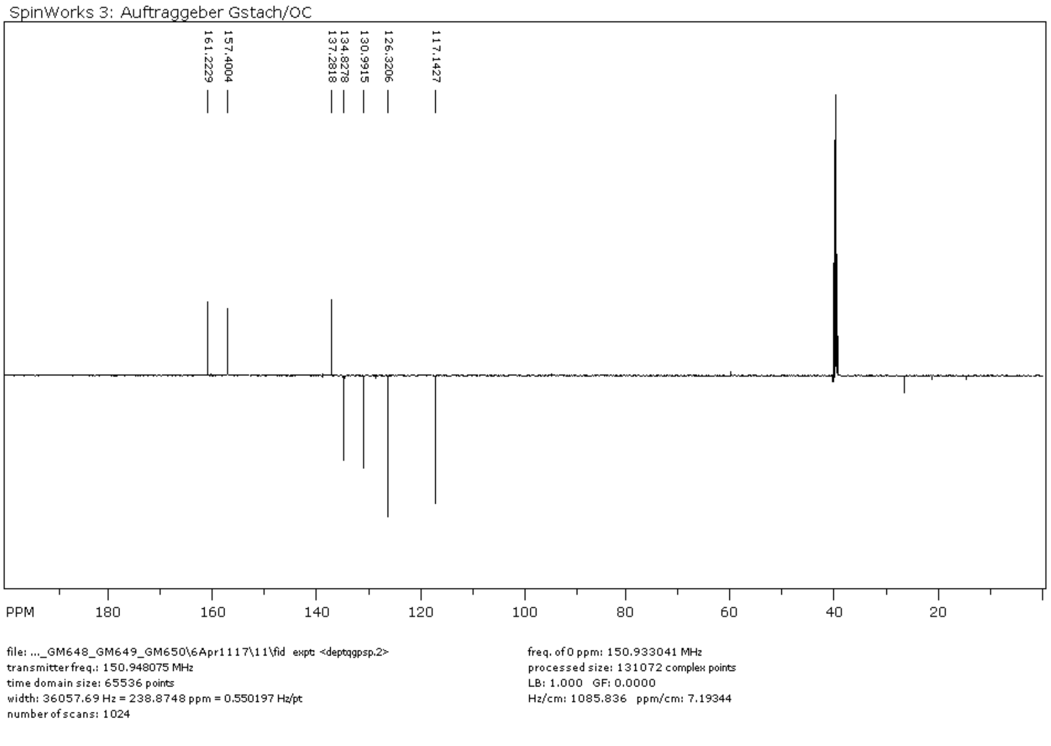

Methyl 2-oxo-2-(pyridin-2-ylamino)acetate (10a)

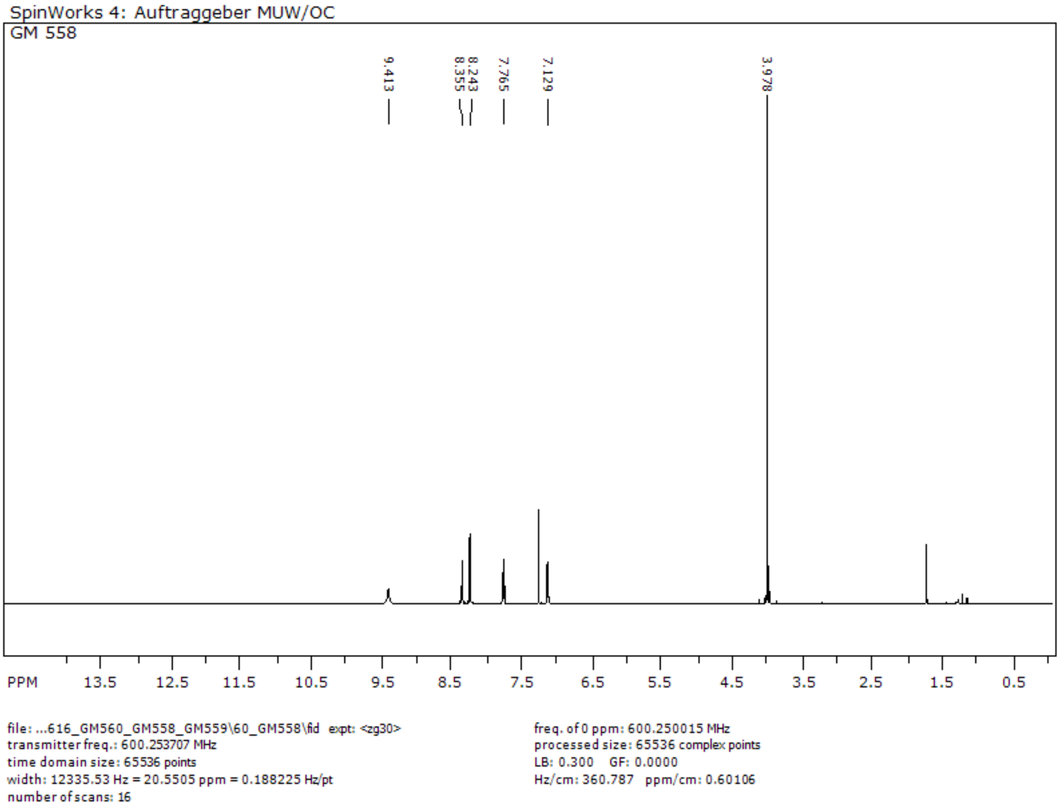

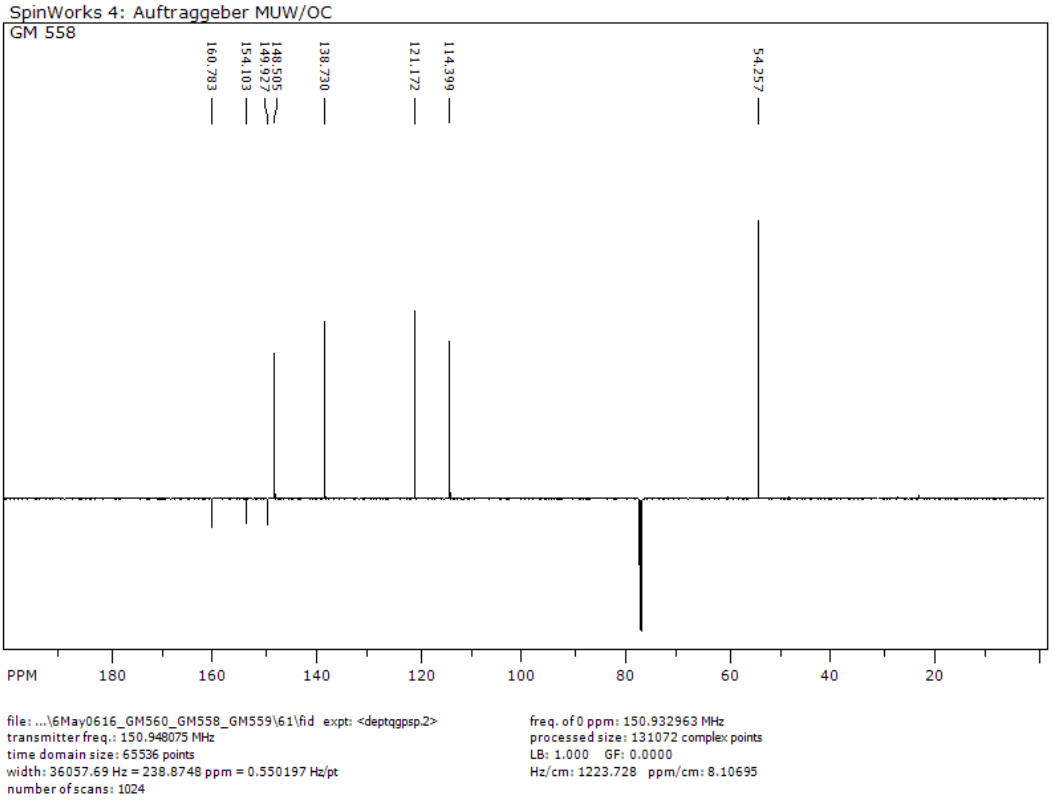

2-(2-Methoxy-2-oxoacetamido)pyridine 1-oxide (12a)

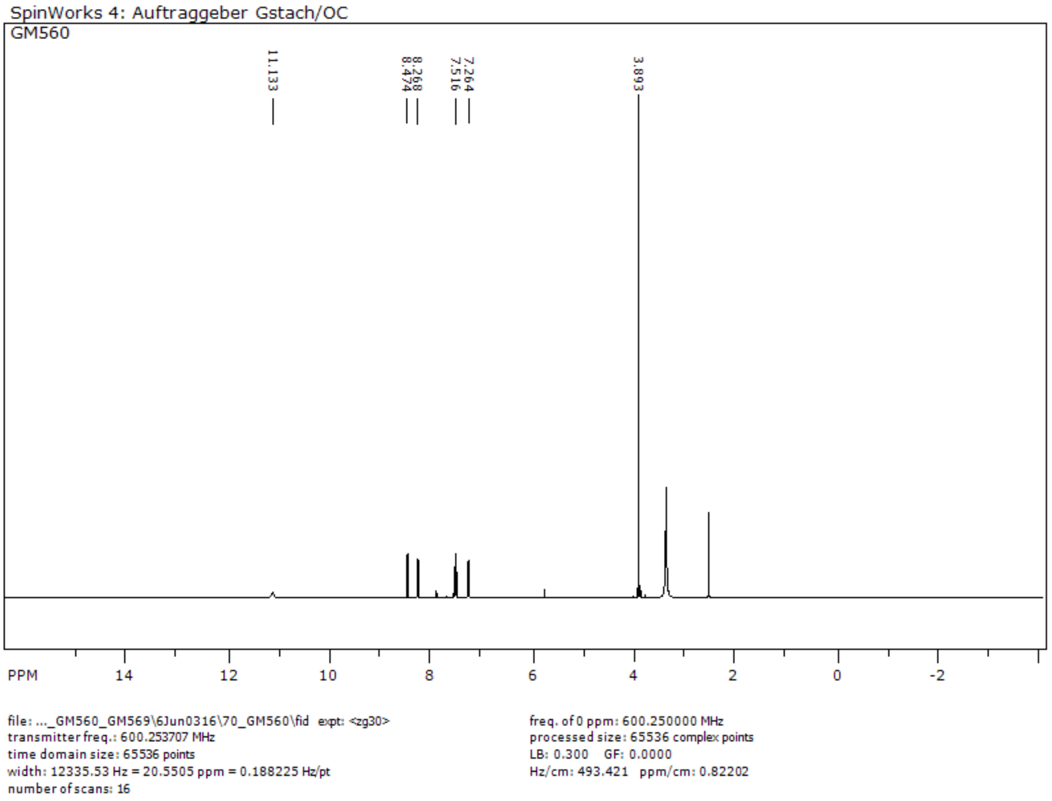

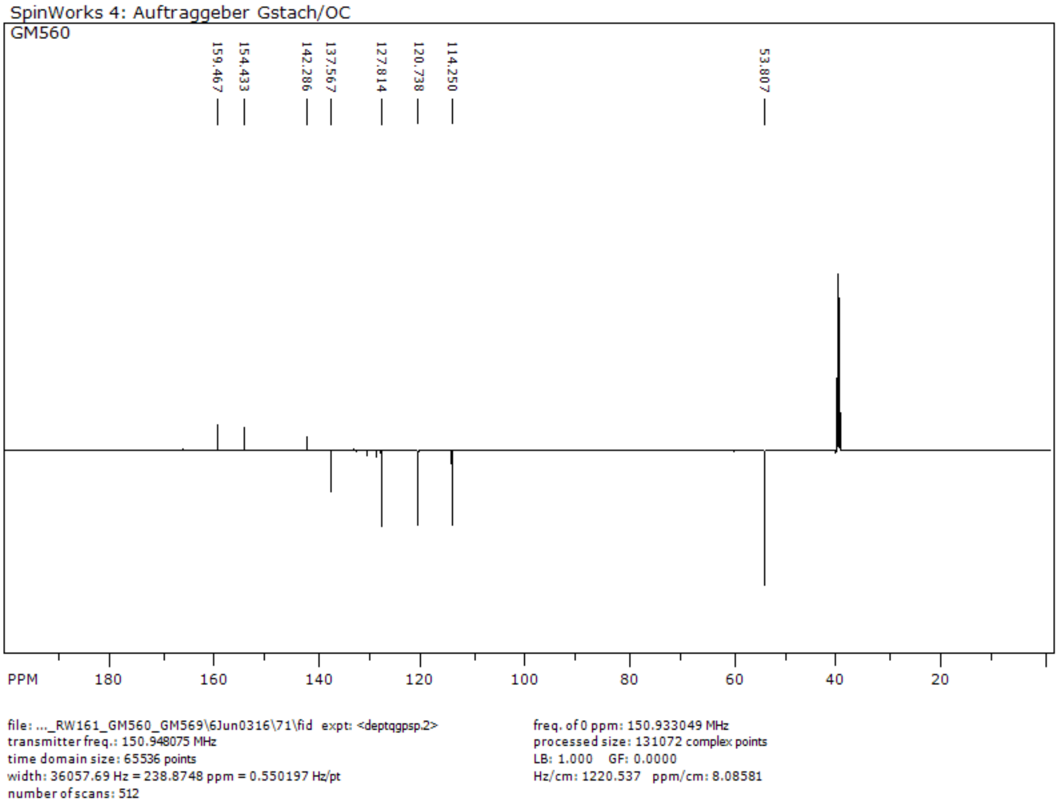

2-(Carboxyformamido)pyridine 1-oxide (12b)

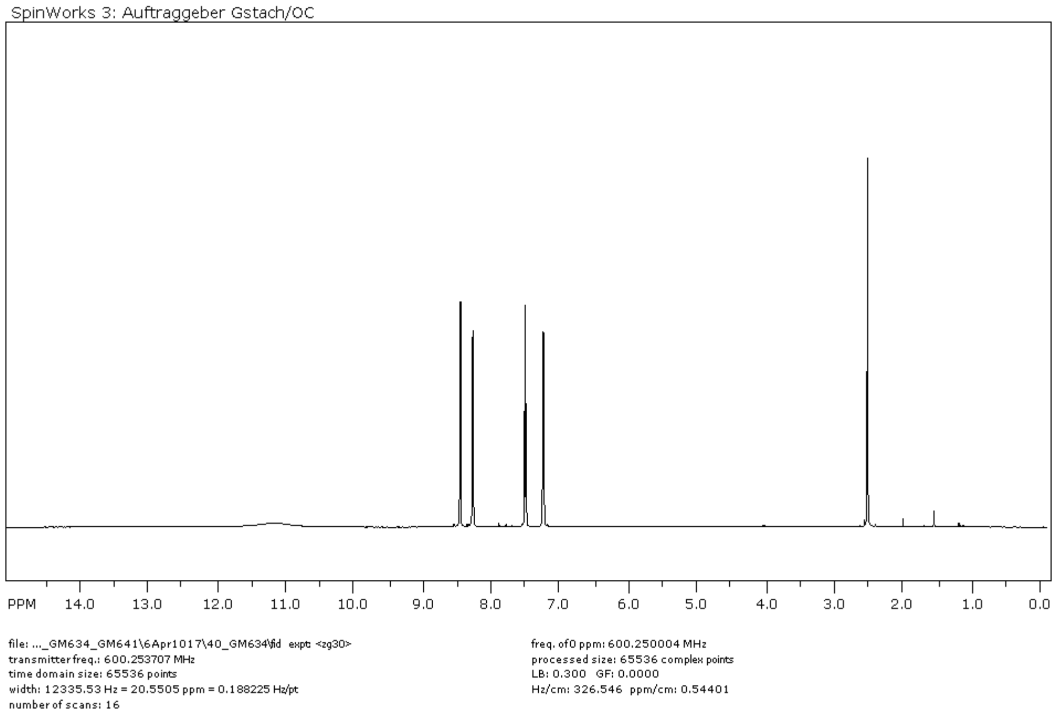

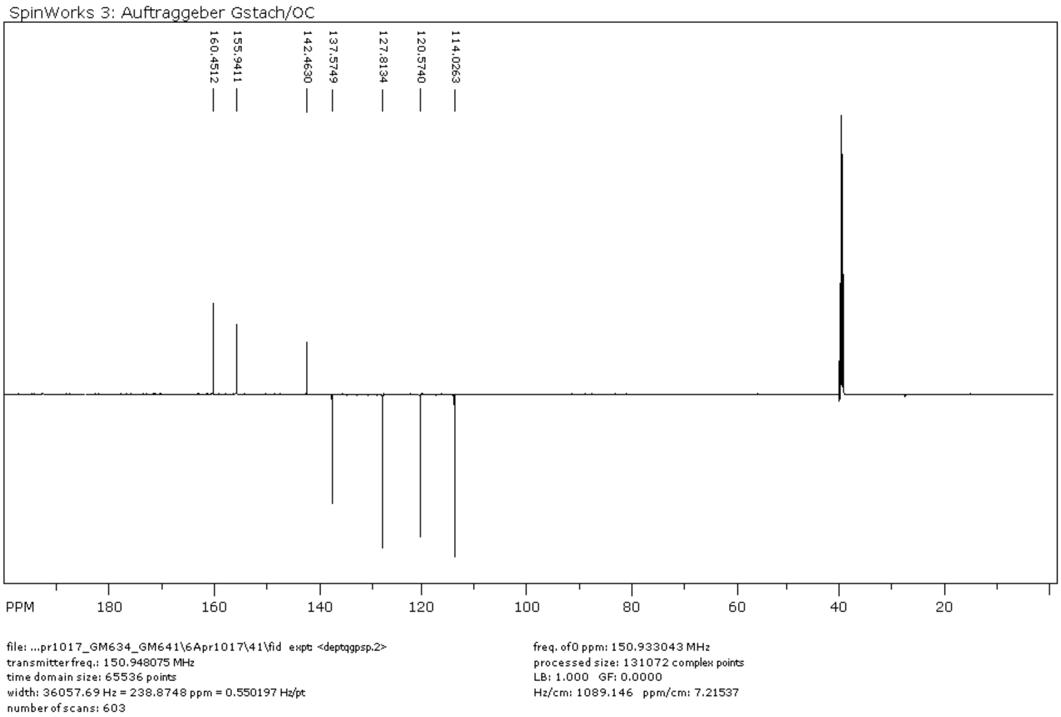

Methyl 2-oxo-2-(quinolin-3-ylamino)acetate (13a)

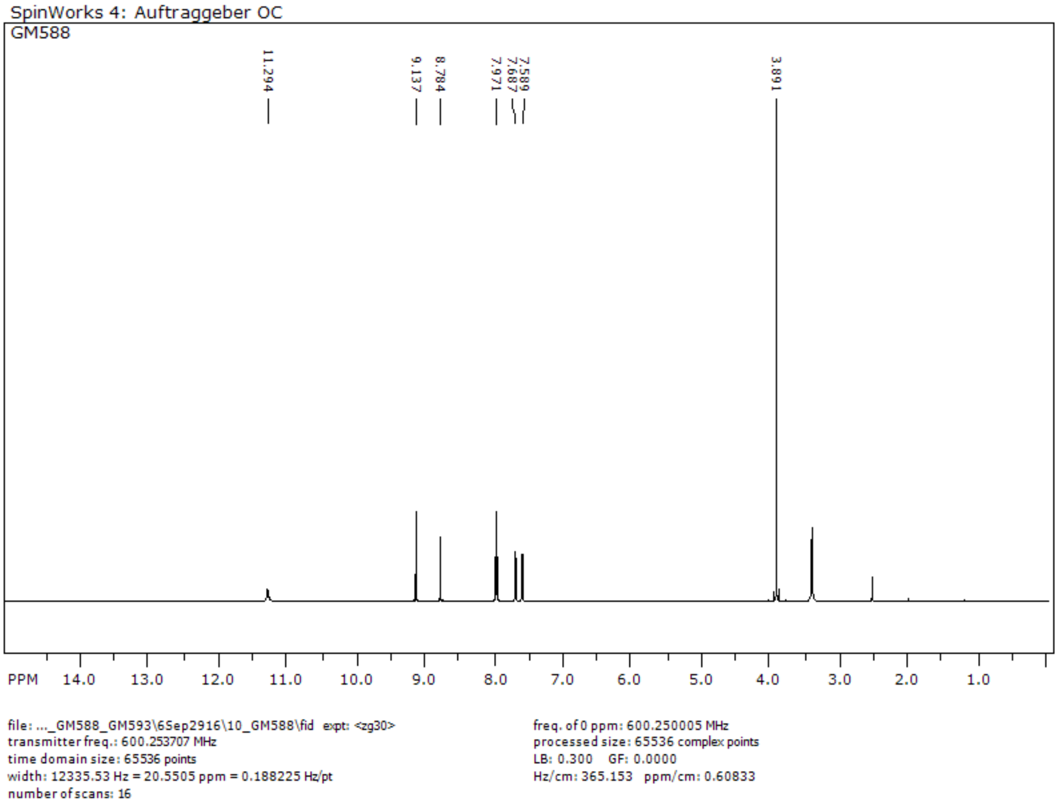

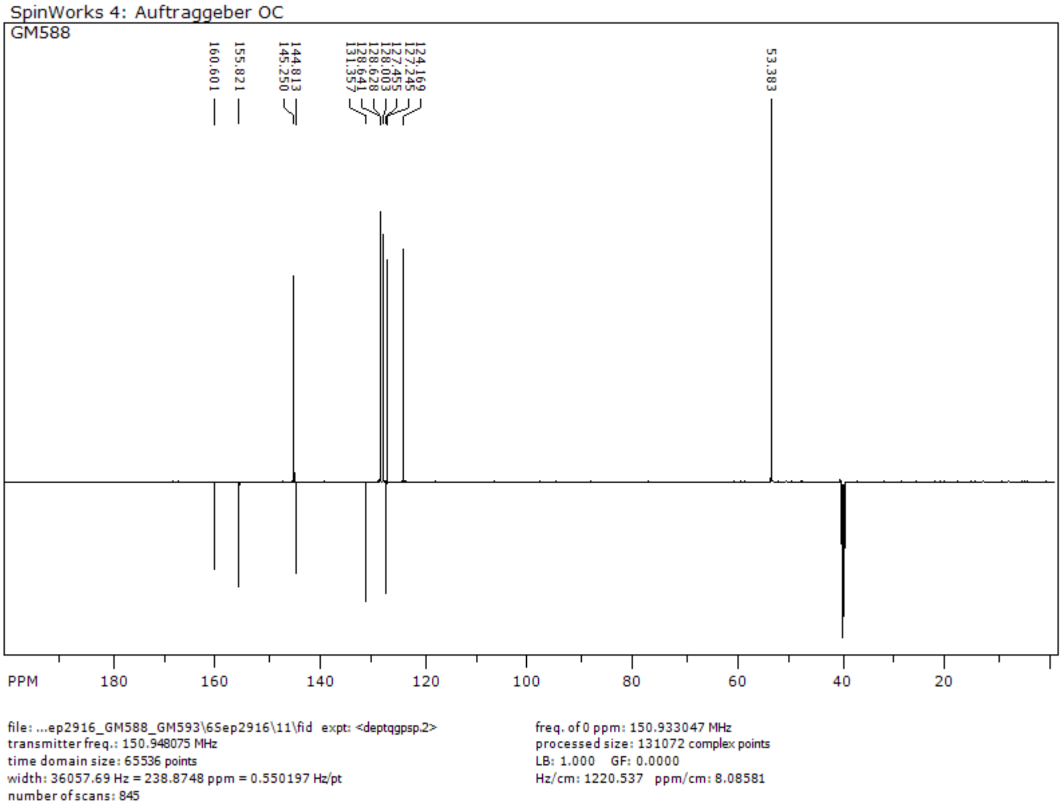

Methyl 2-oxo-2-(quinolin-2-ylamino)acetate (14a)

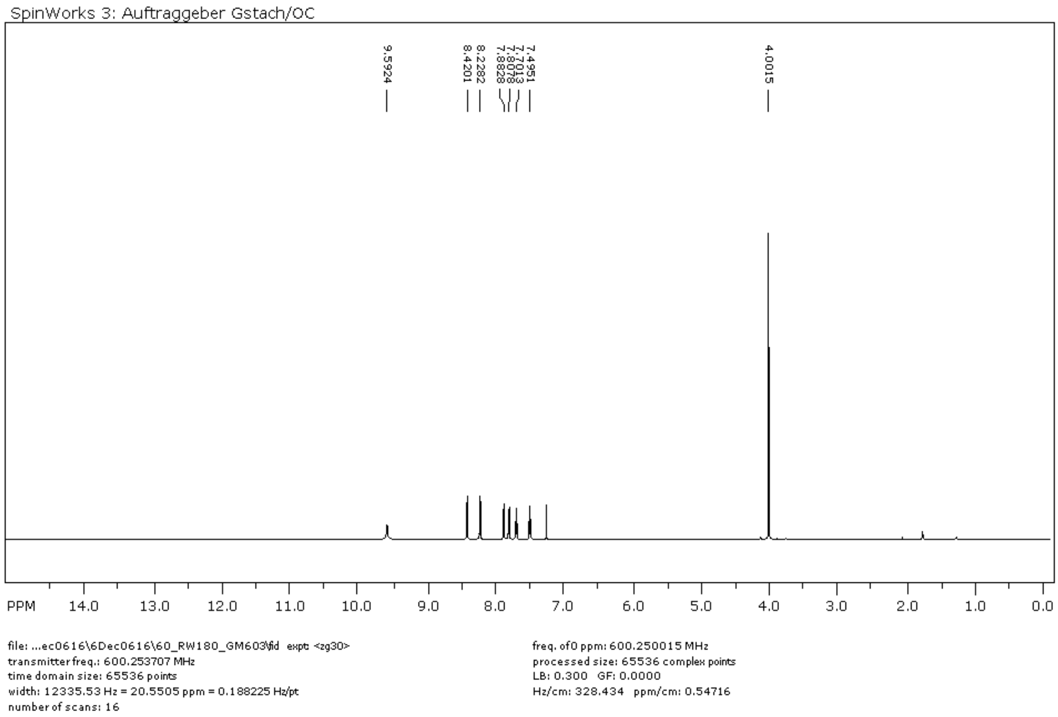

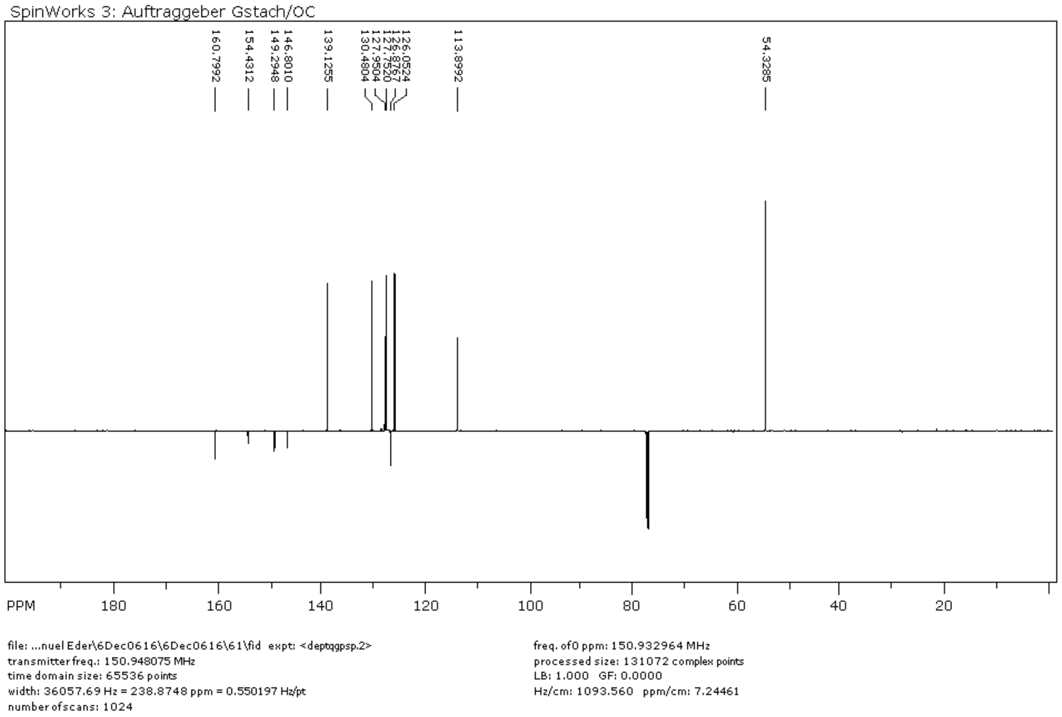

3-(2-Methoxy-2-oxoacetamido)quinoline 1-oxide (15a)

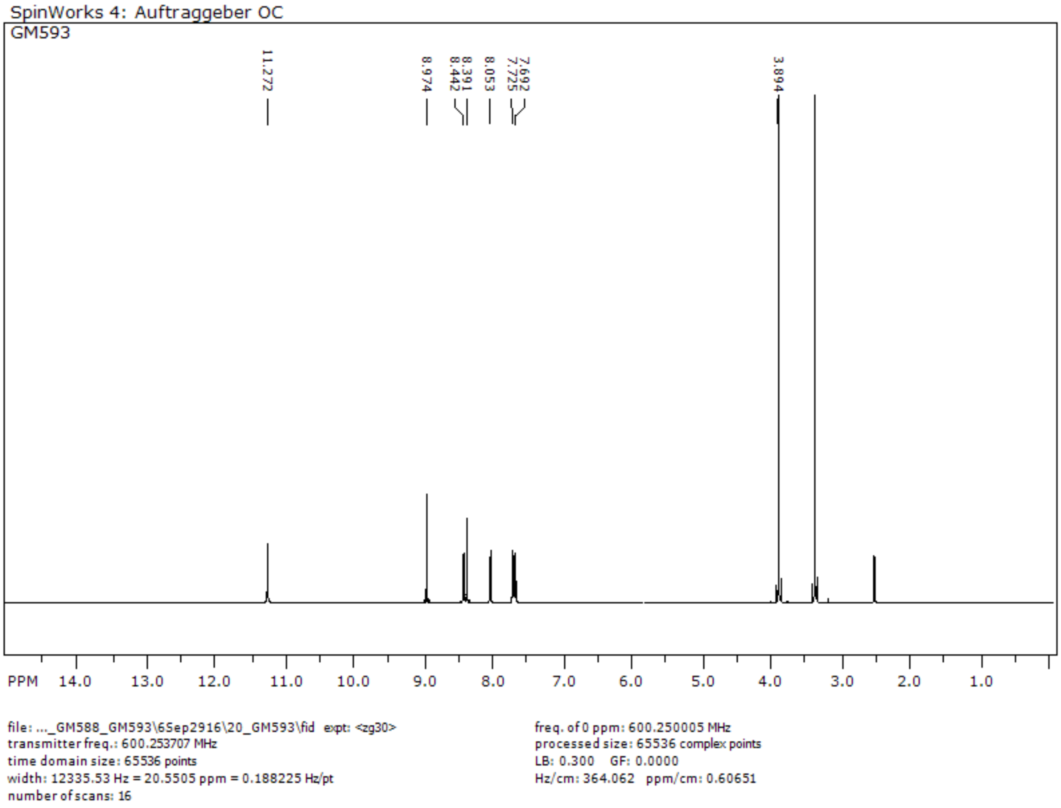

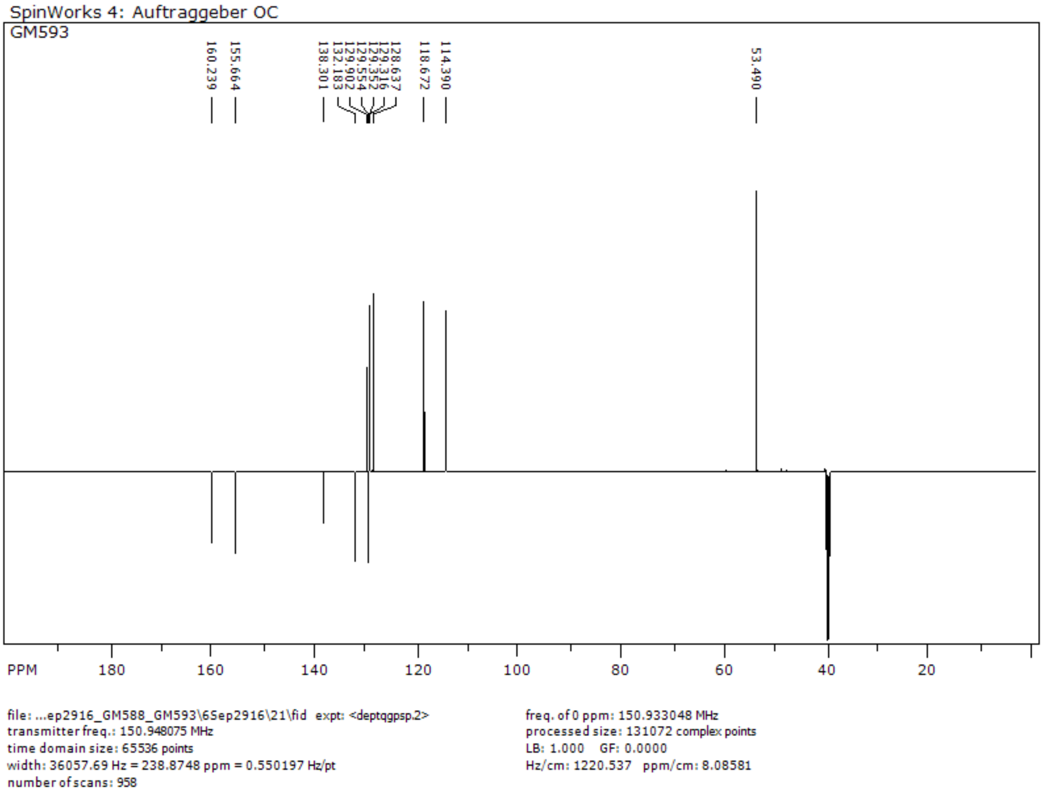

3-(Carboxyformamido)quinoline 1-oxide (15b)

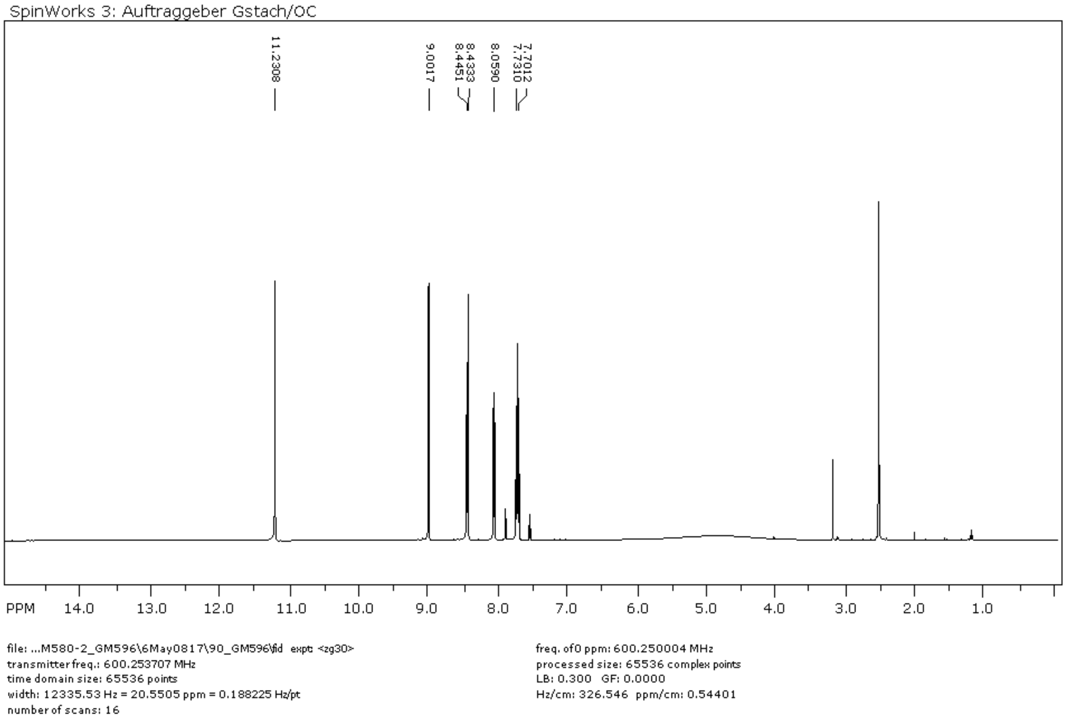

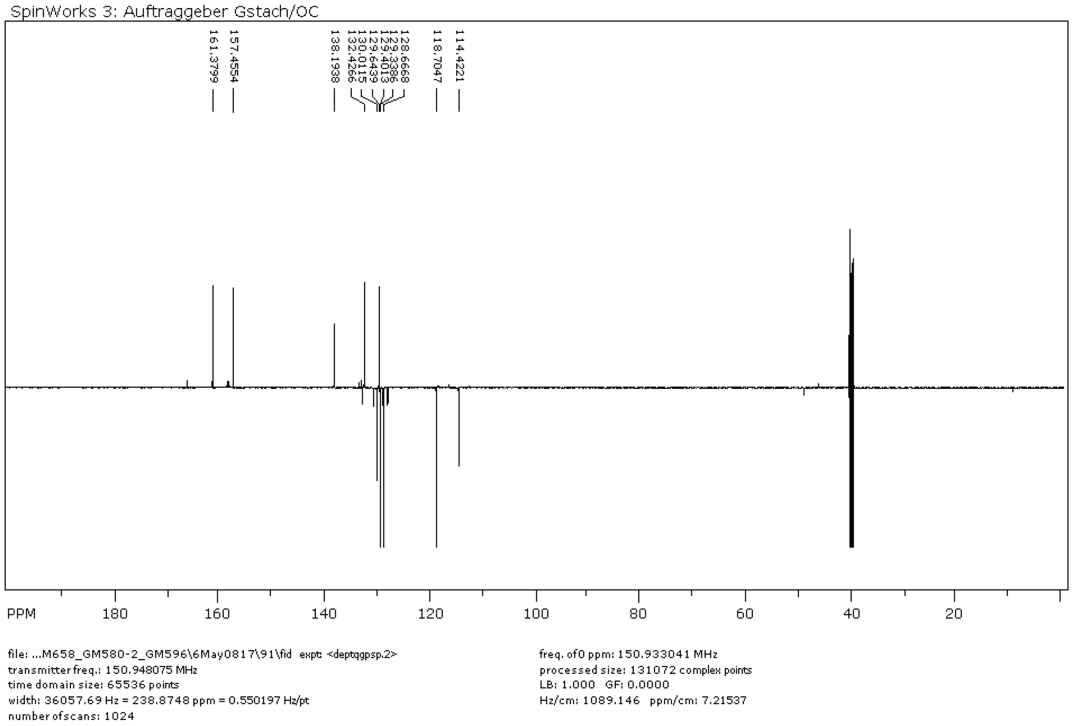

2-(2-Methoxy-2-oxoacetamido)quinoline 1-oxide (16a)

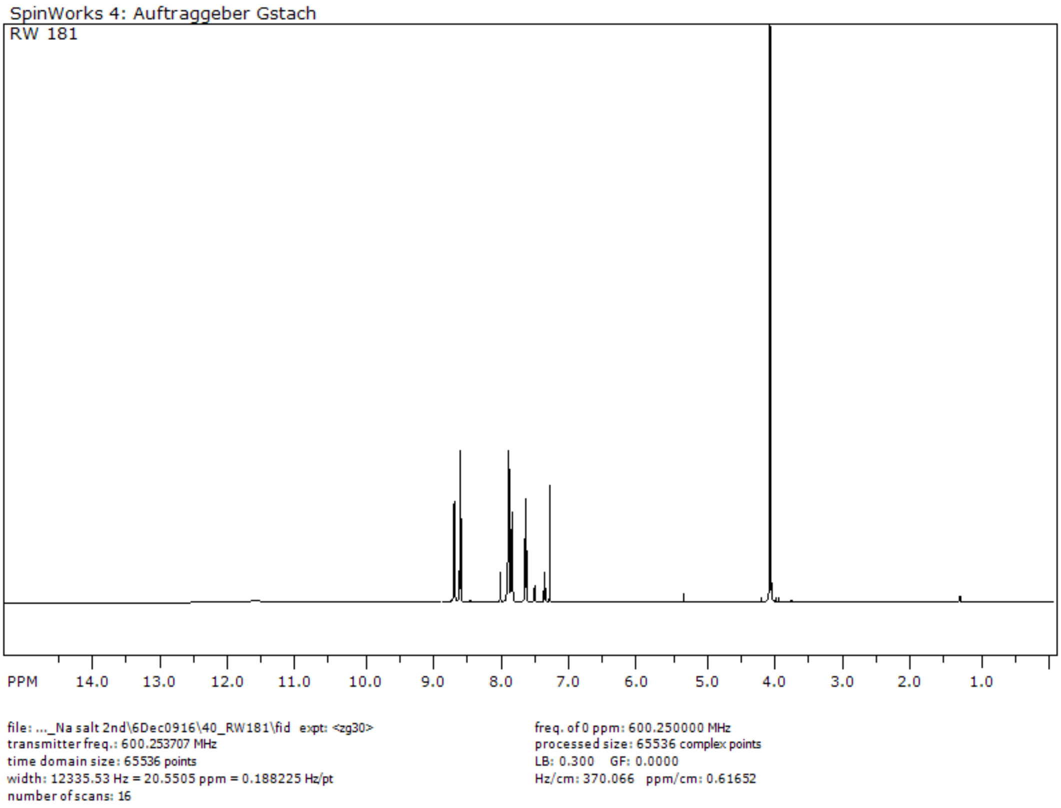

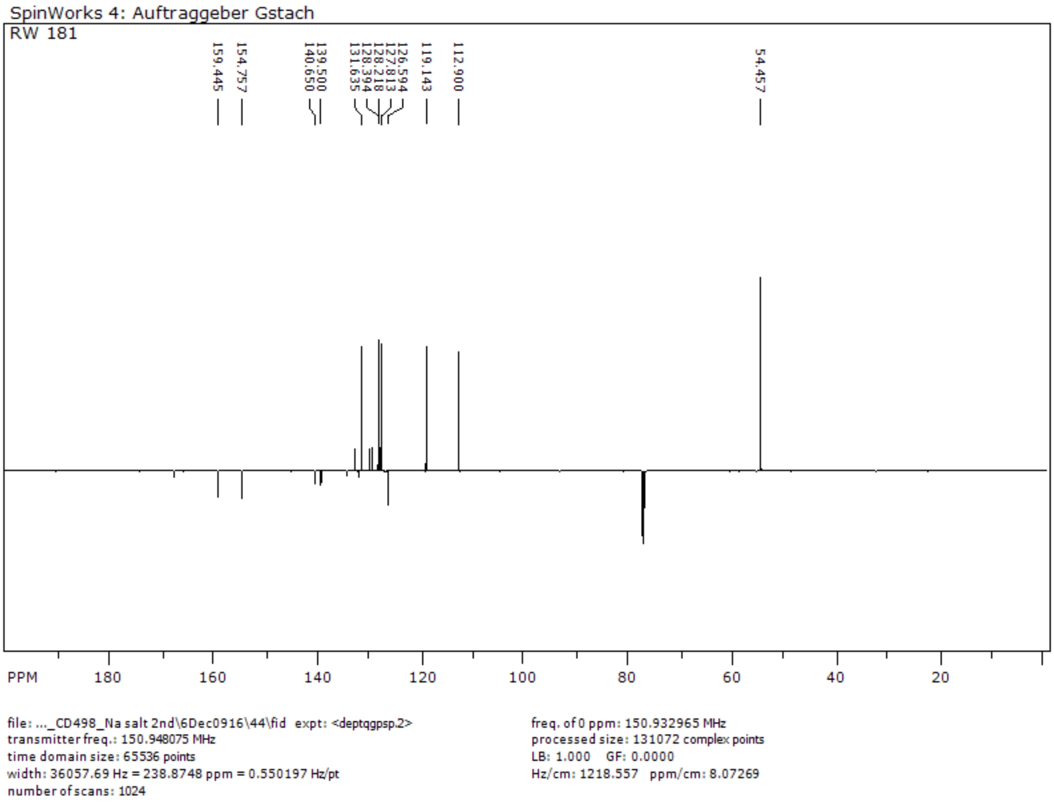

2-(Carboxyformamido)quinoline 1-oxide (16b)

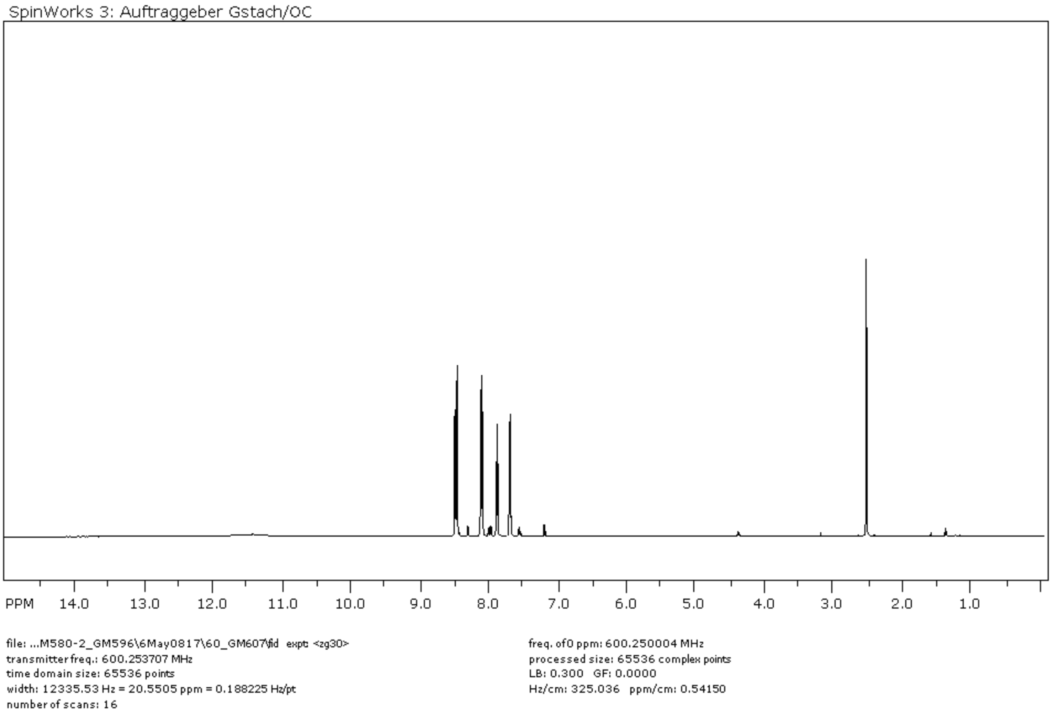

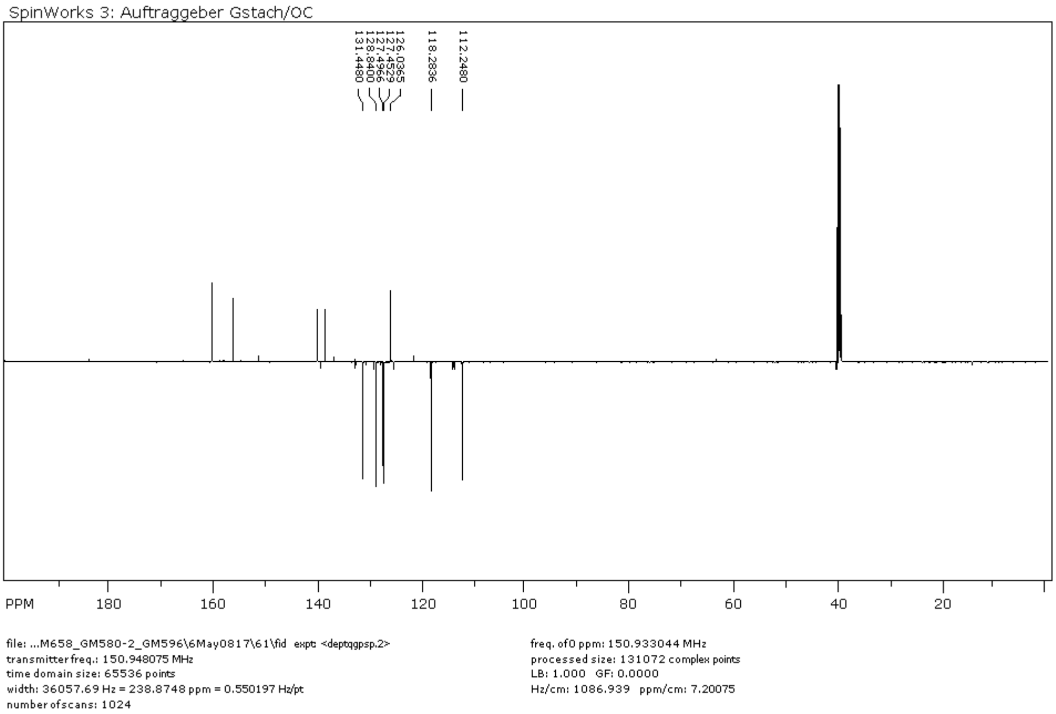

Methyl 4-oxo-4*H*-benzo[*d*][1,3]oxazine-2-carboxylate (17a)

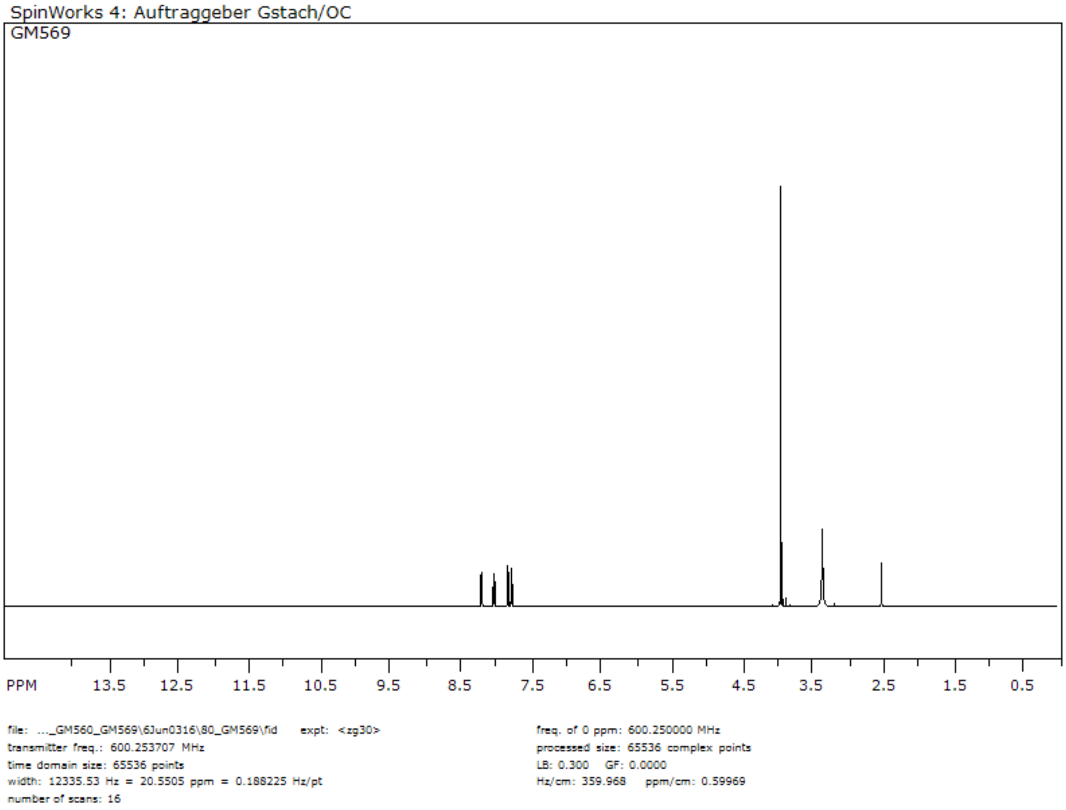

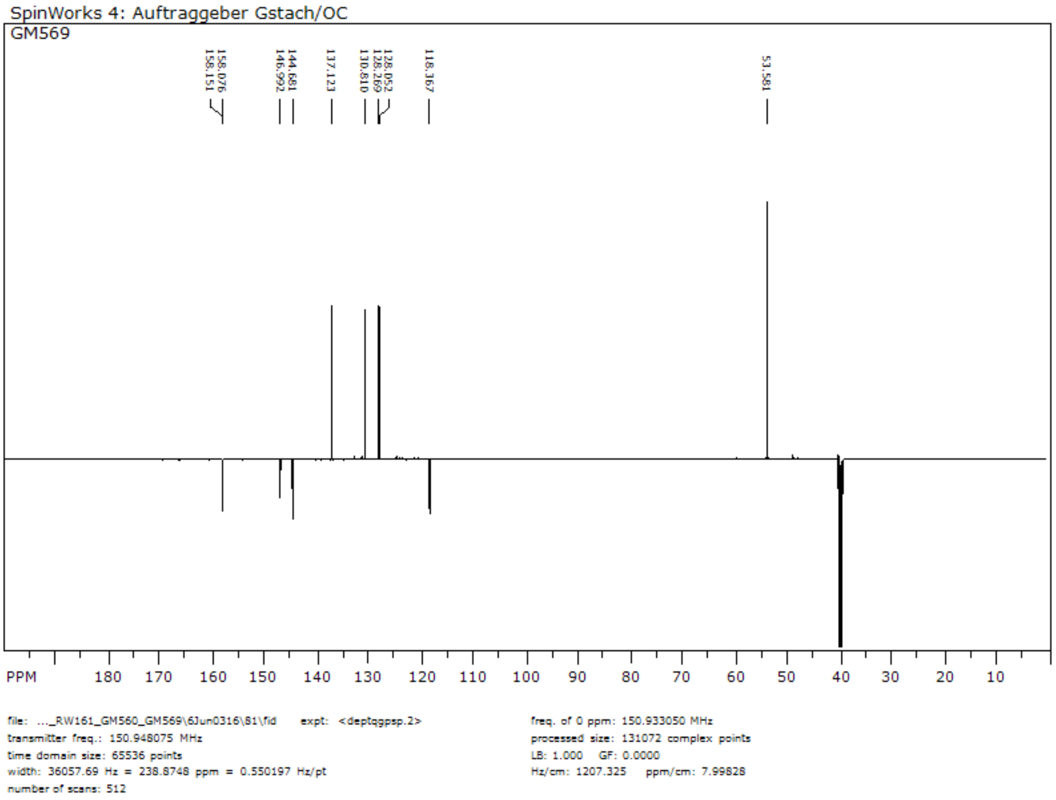

Methyl 2-((2-carbamoylphenyl)amino)-2-oxoacetate (18a)

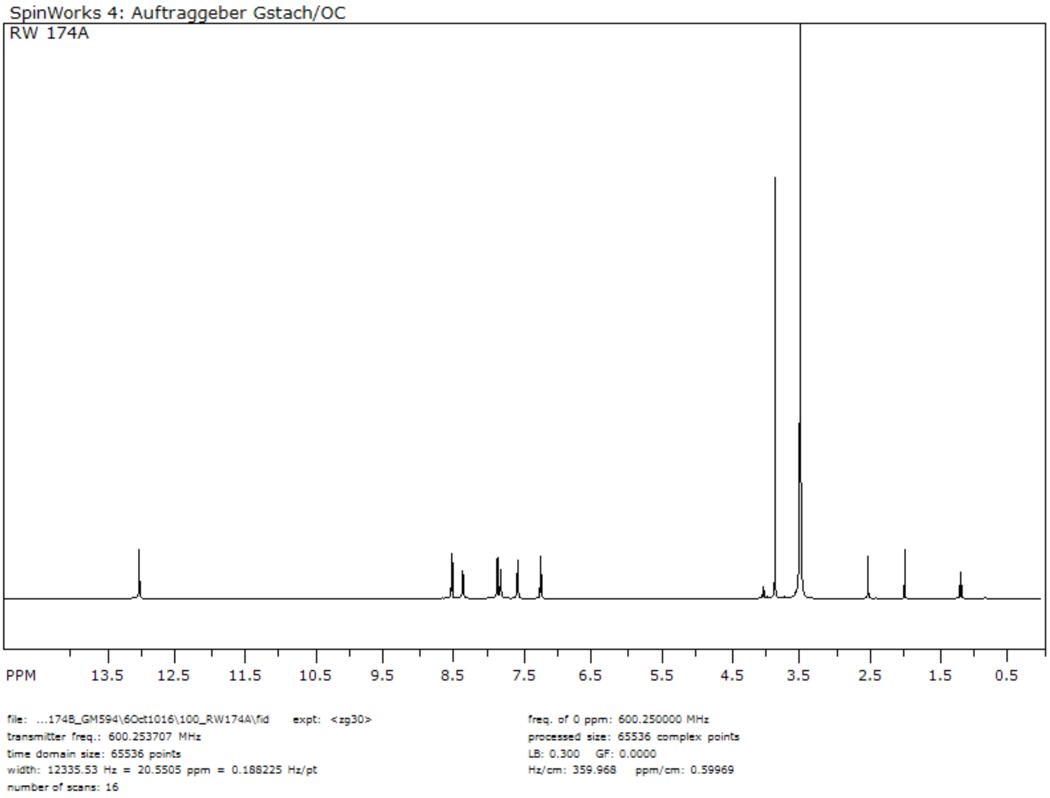

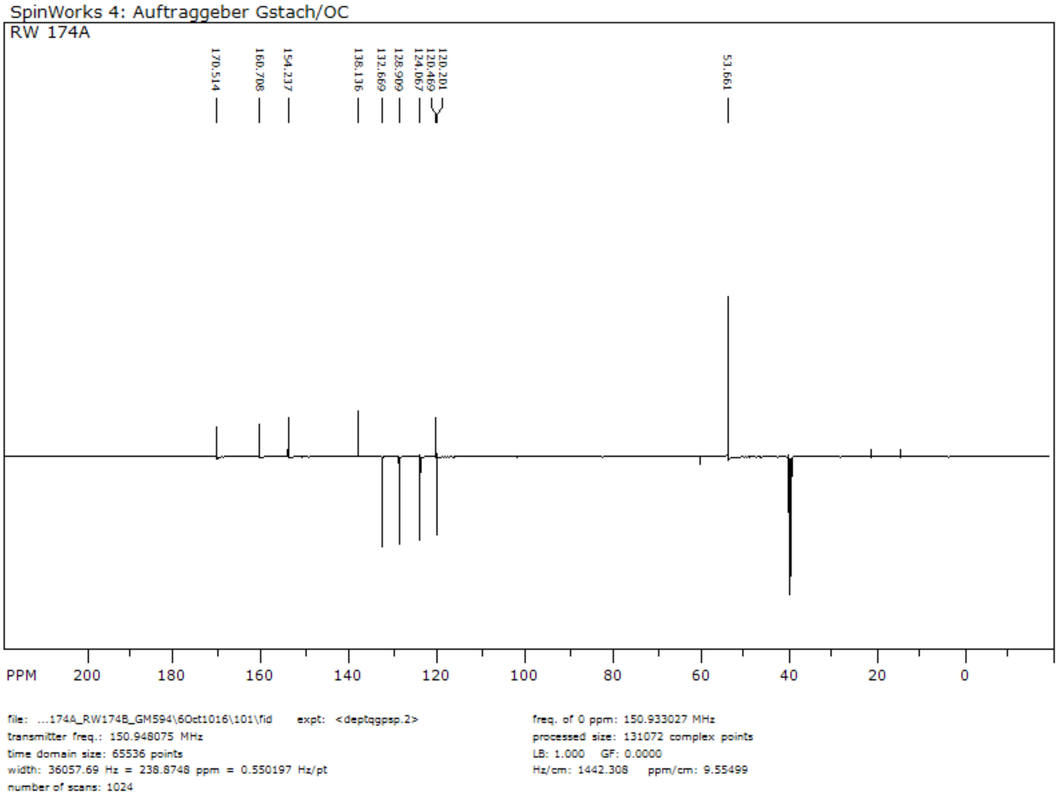

Methyl 4-oxo-1,4-dihydroquinazoline-2-carboxylate (19a)

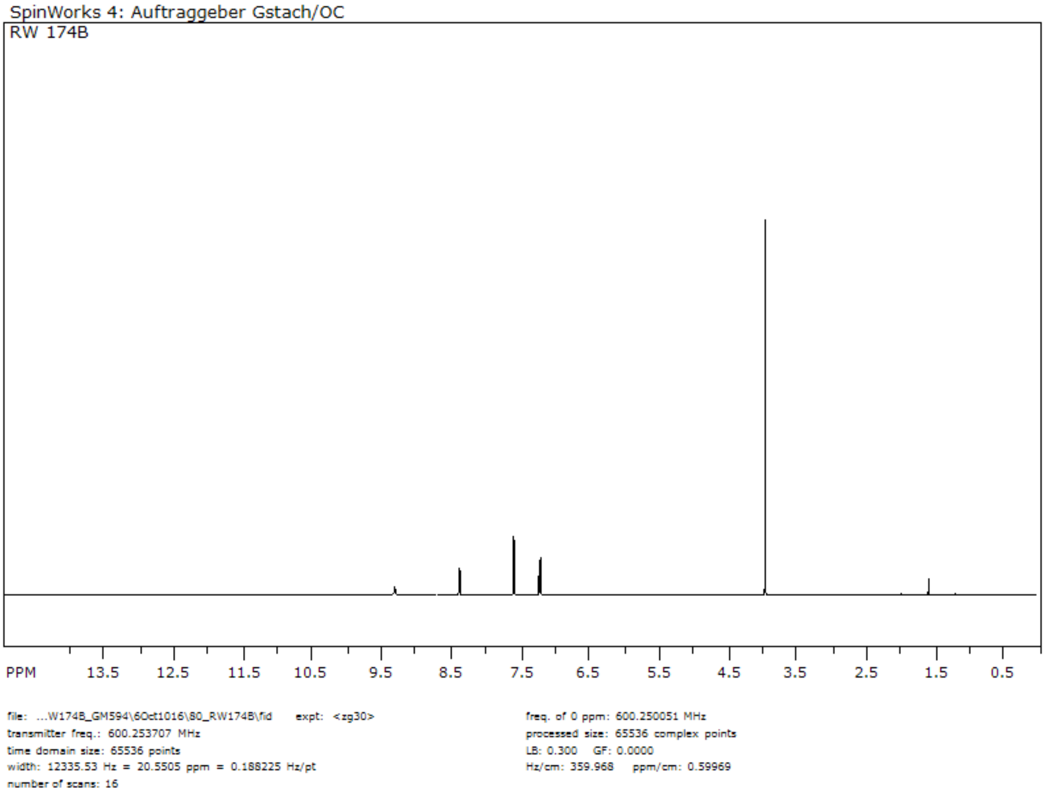

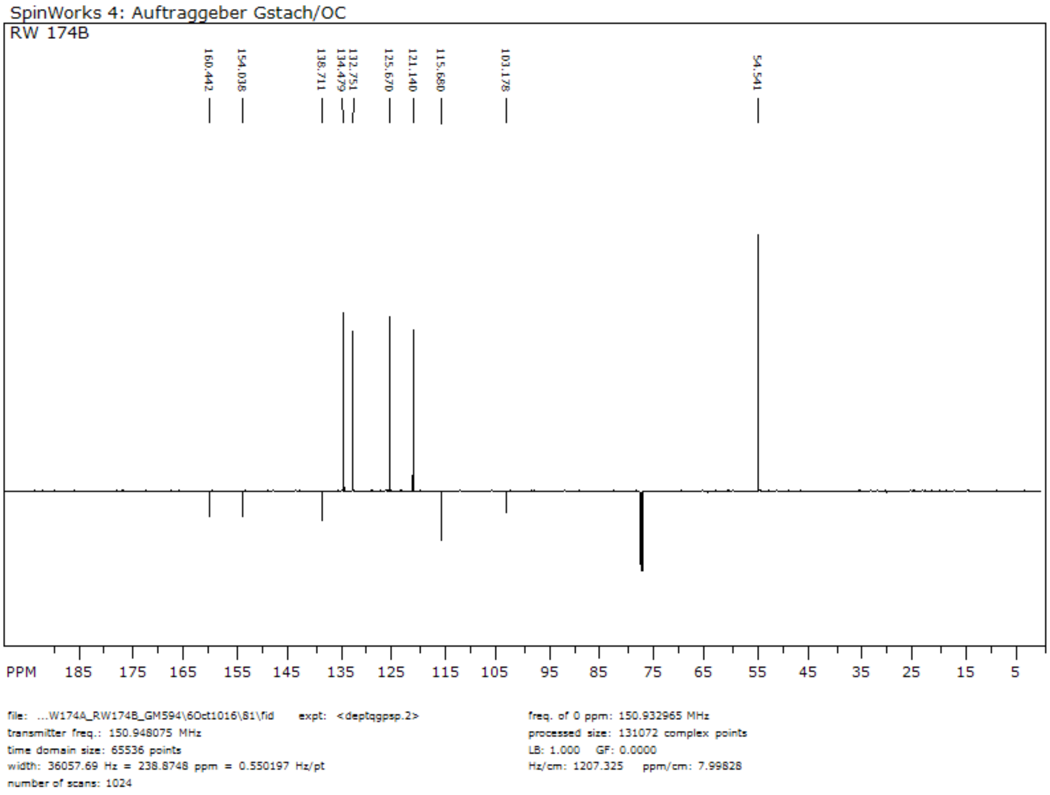

4-oxo-1,4-dihydroquinazoline-2-carboxylic acid (19b)

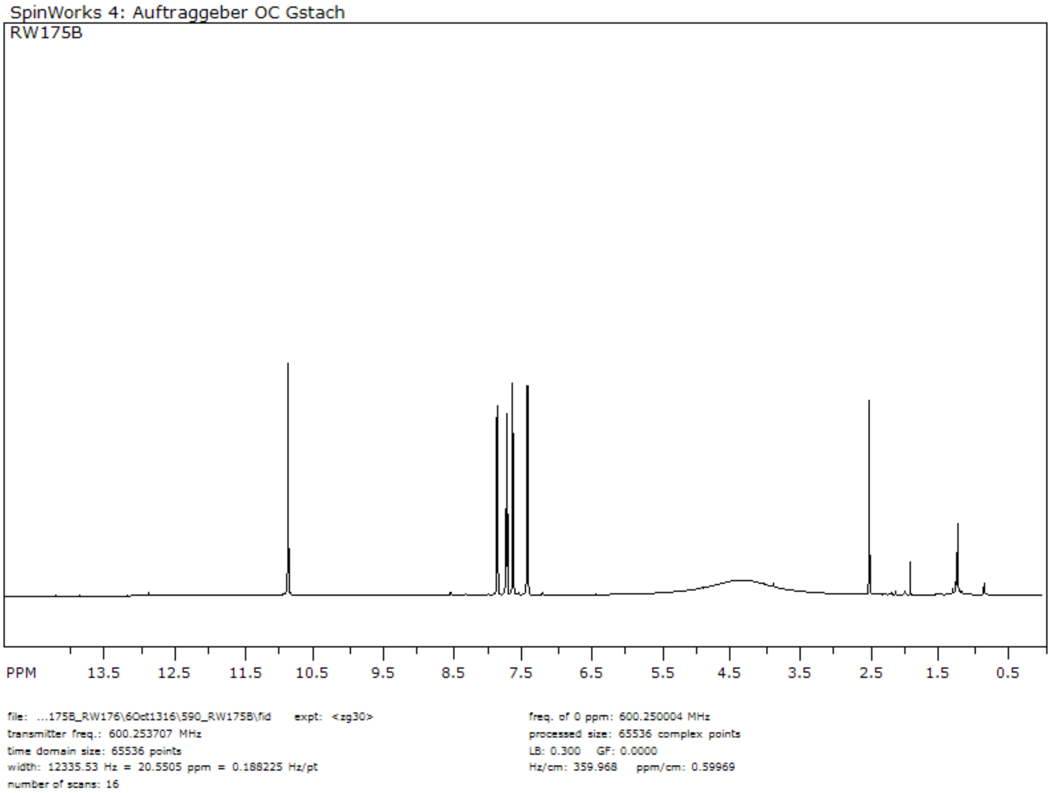

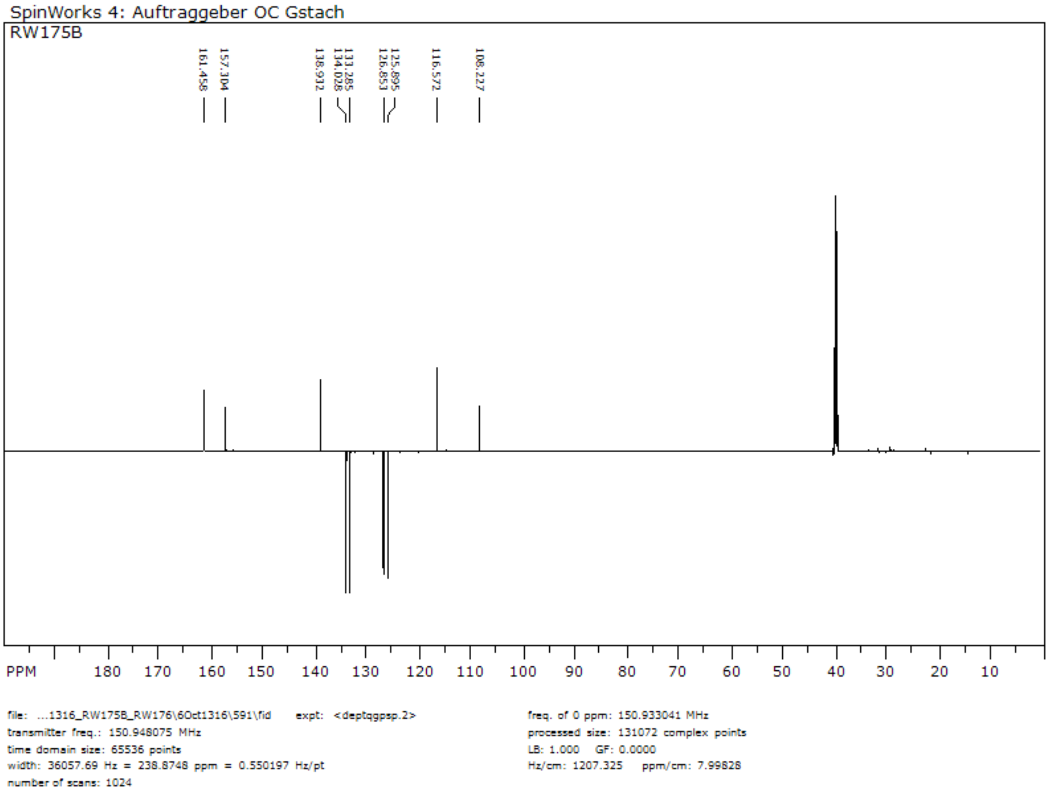

Methyl 2-((2-methoxyphenyl)amino)-2-thioxoacetate (20a)

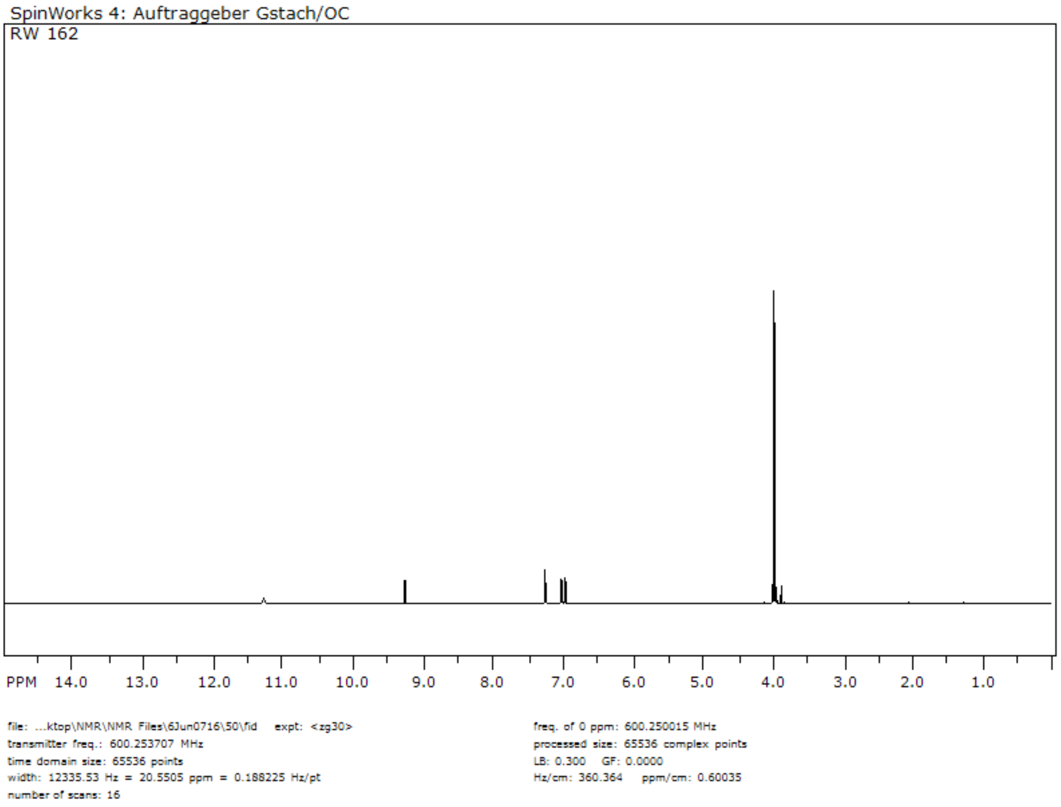

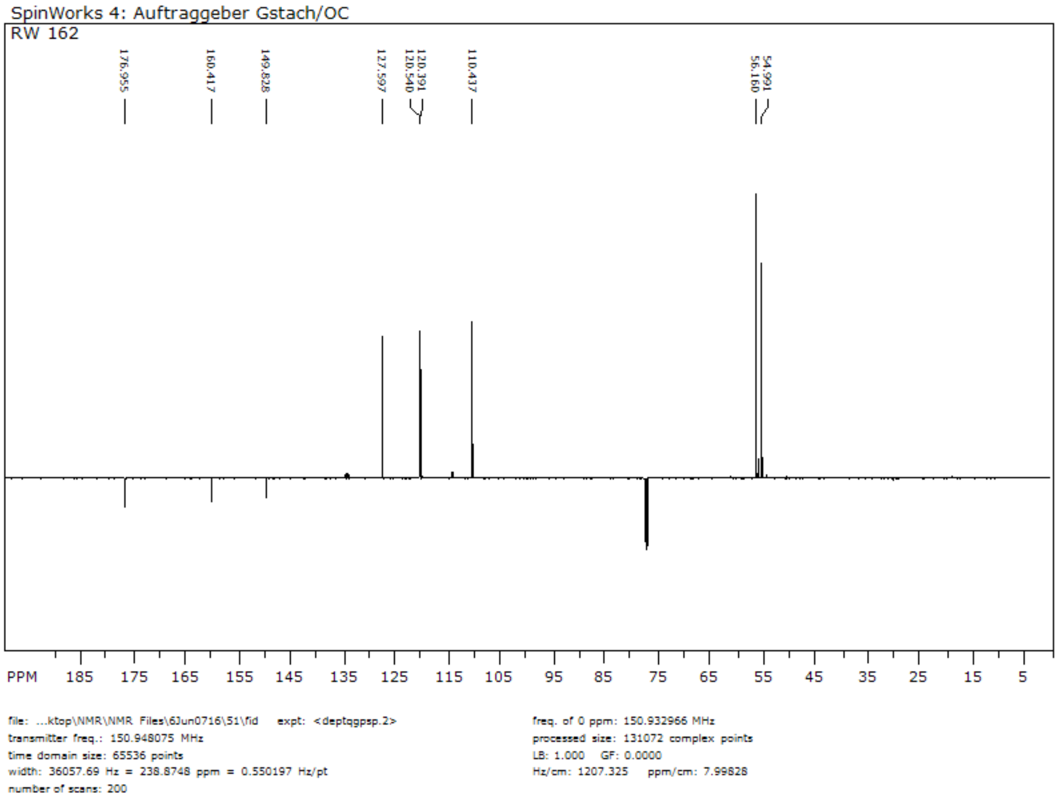

2-((2-methoxyphenyl)amino)-2-thioxoacetic acid (20b)

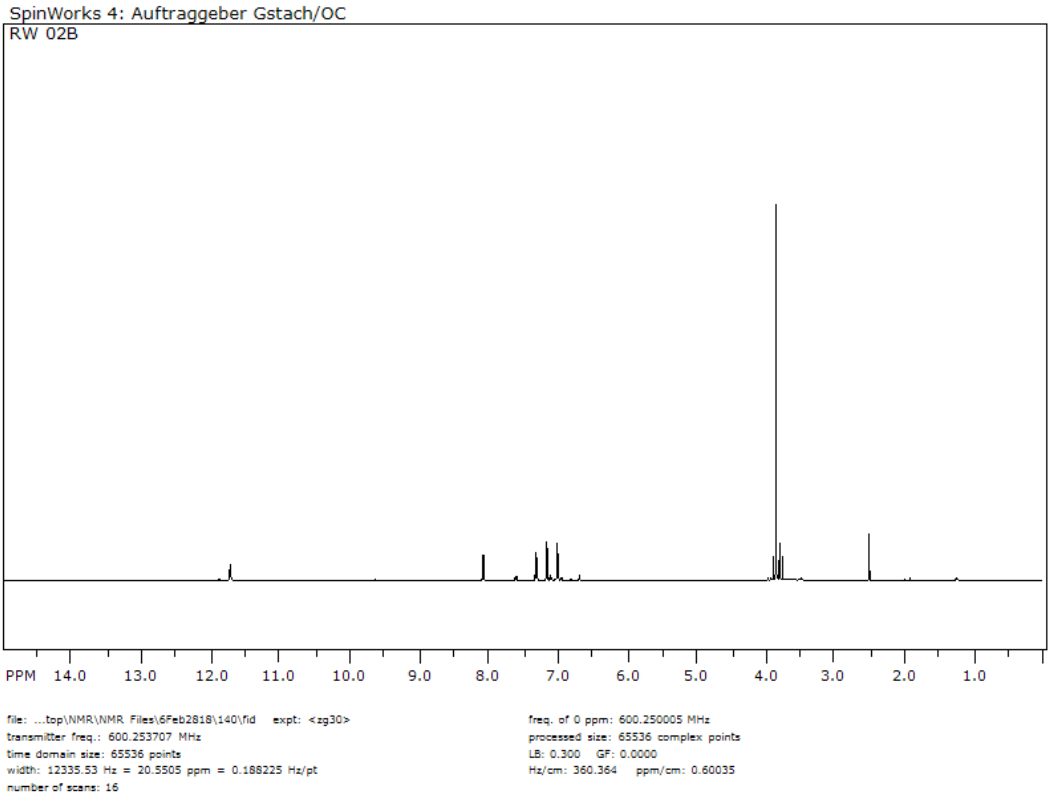

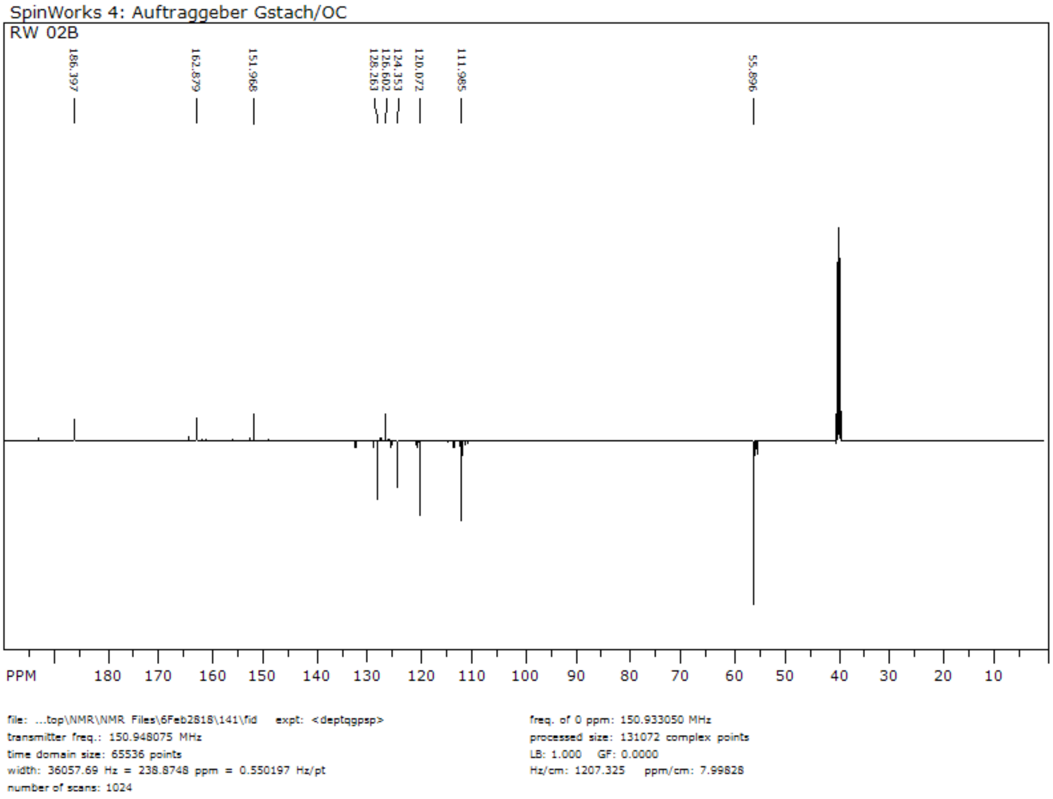

Methyl 2-((3-methoxyphenyl)amino)-2-thioxoacetate (21a)

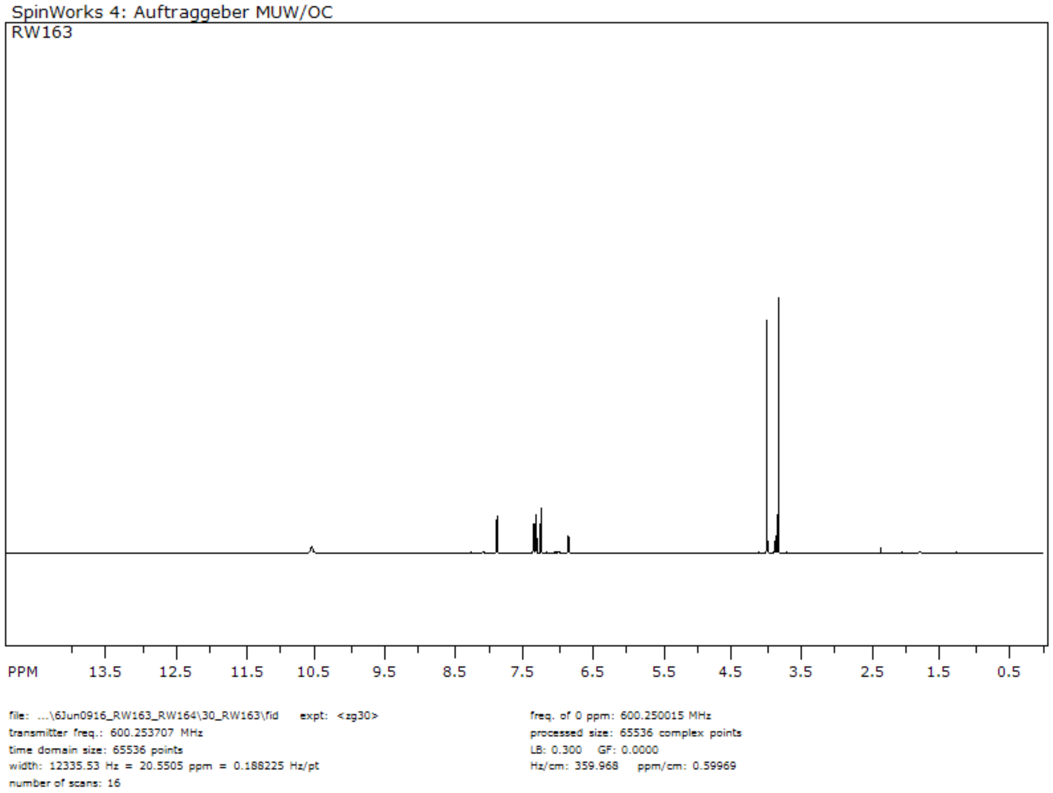

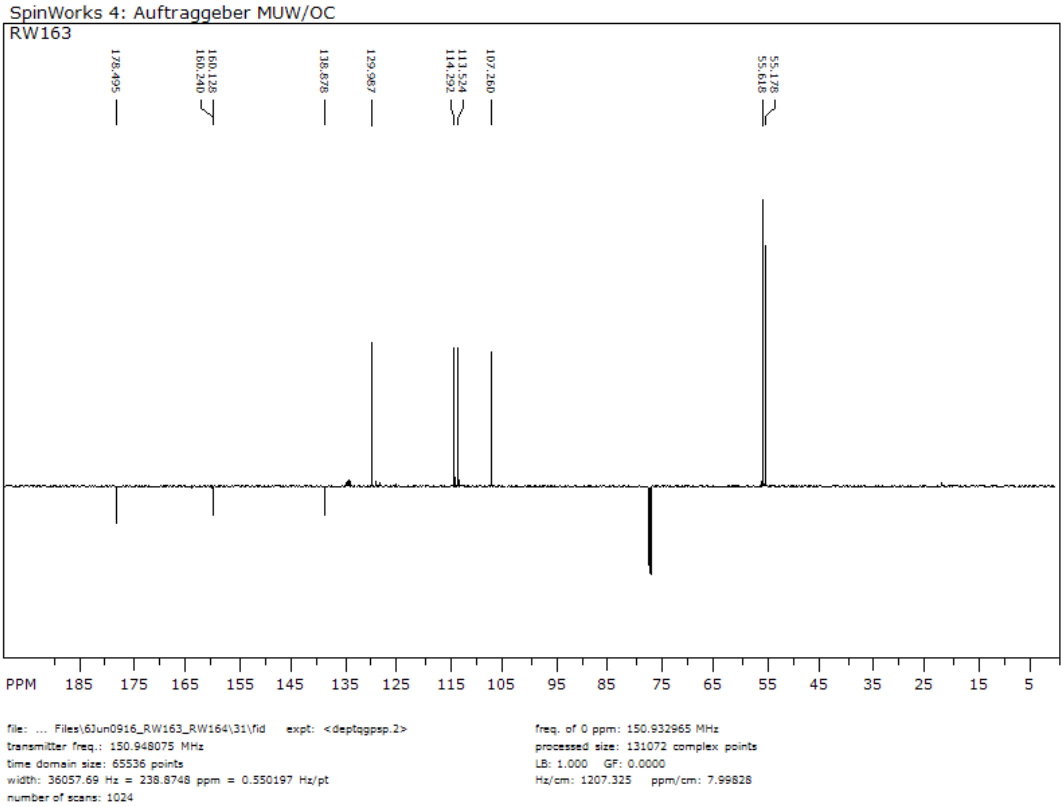

2-((3-methoxyphenyl)amino)-2-thioxoacetic acid (21b)

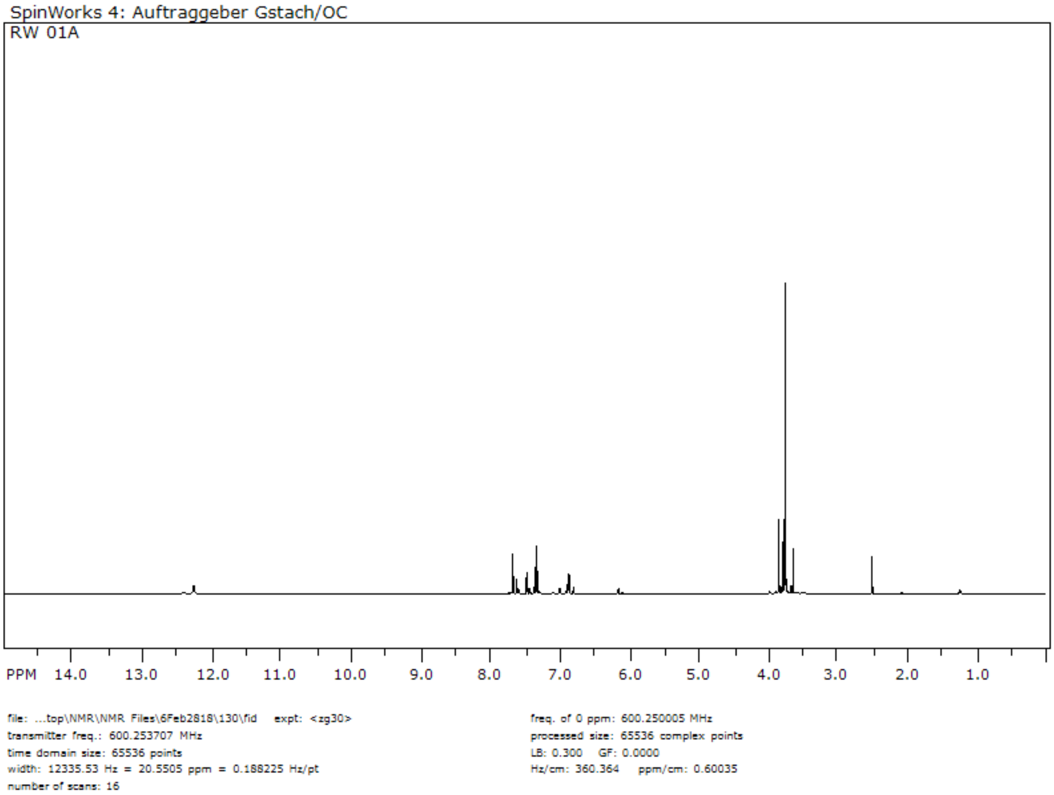

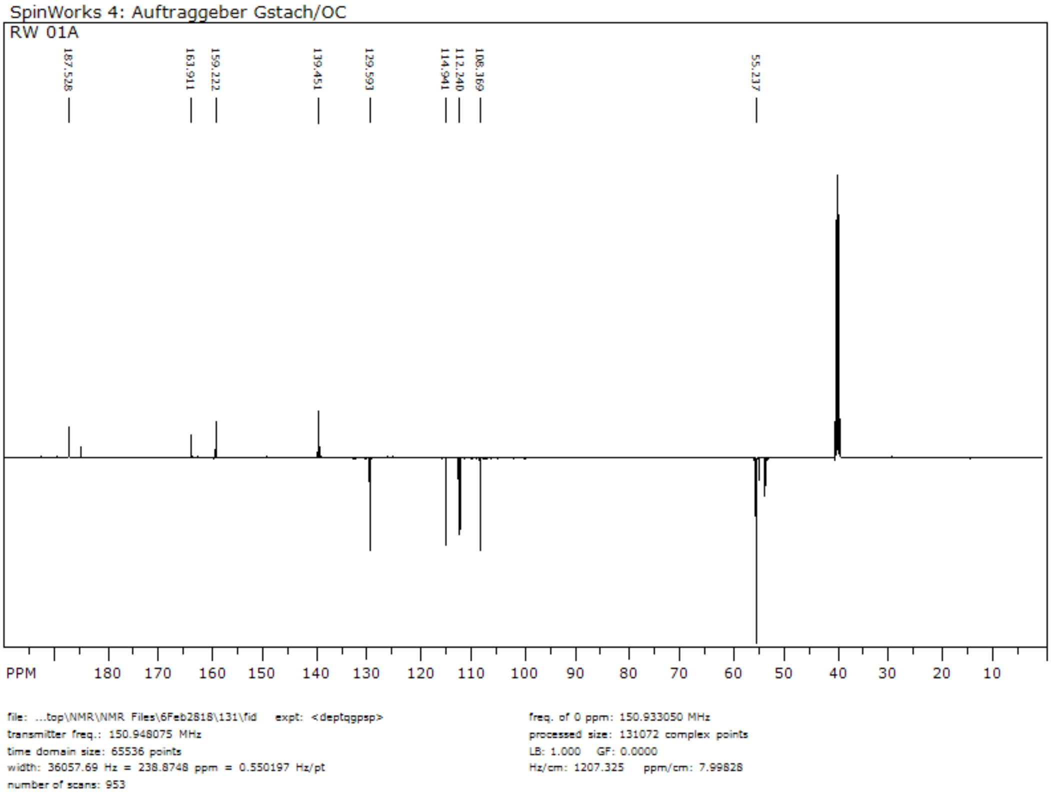

Methyl 2-((4-methoxyphenyl)amino)-2-thioxoacetate (22a)

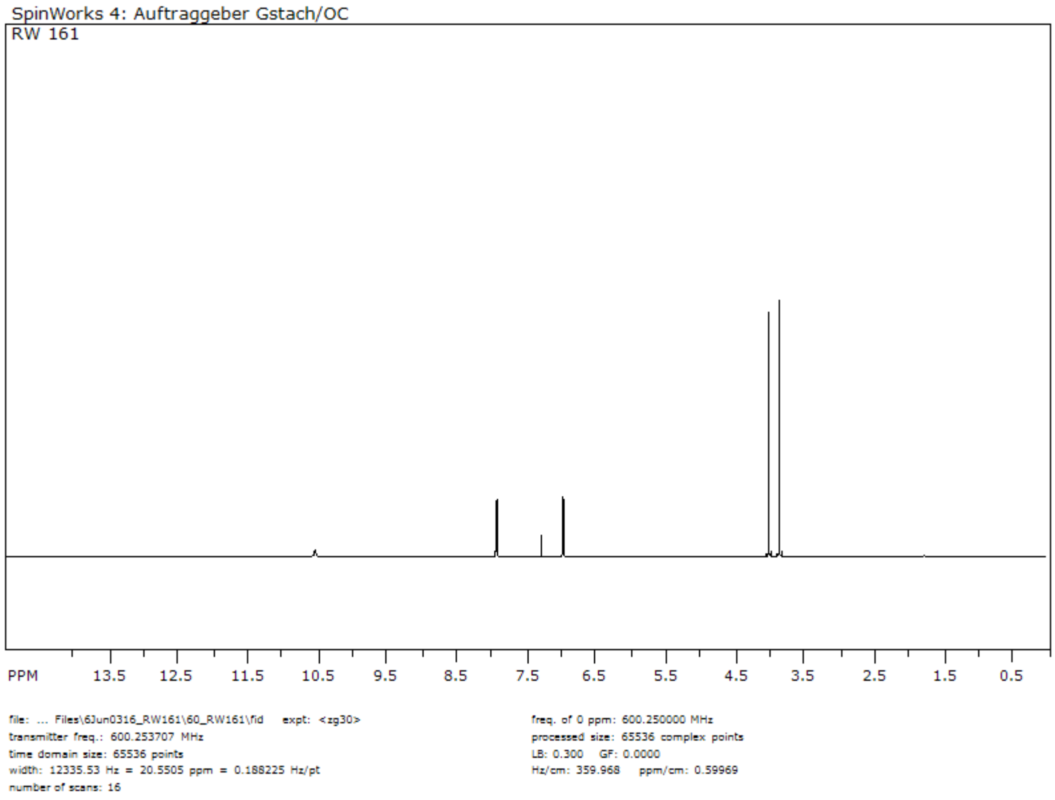

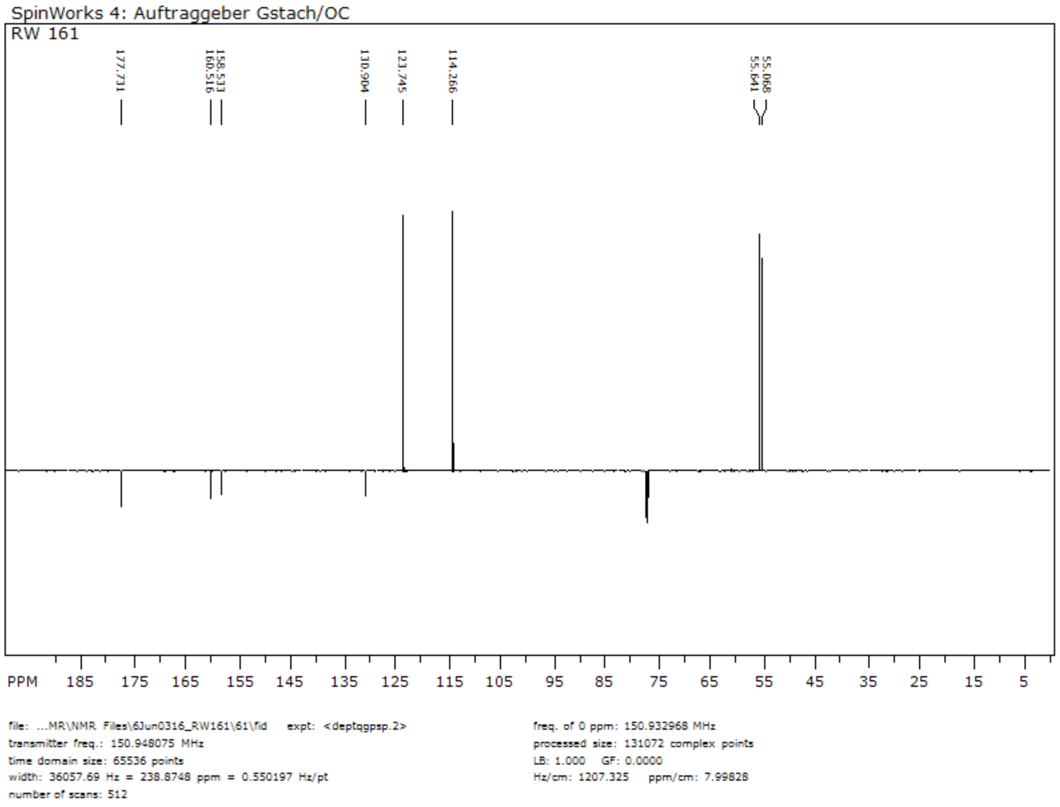

2-((4-methoxyphenyl)amino)-2-thioxoacetic acid (22b)

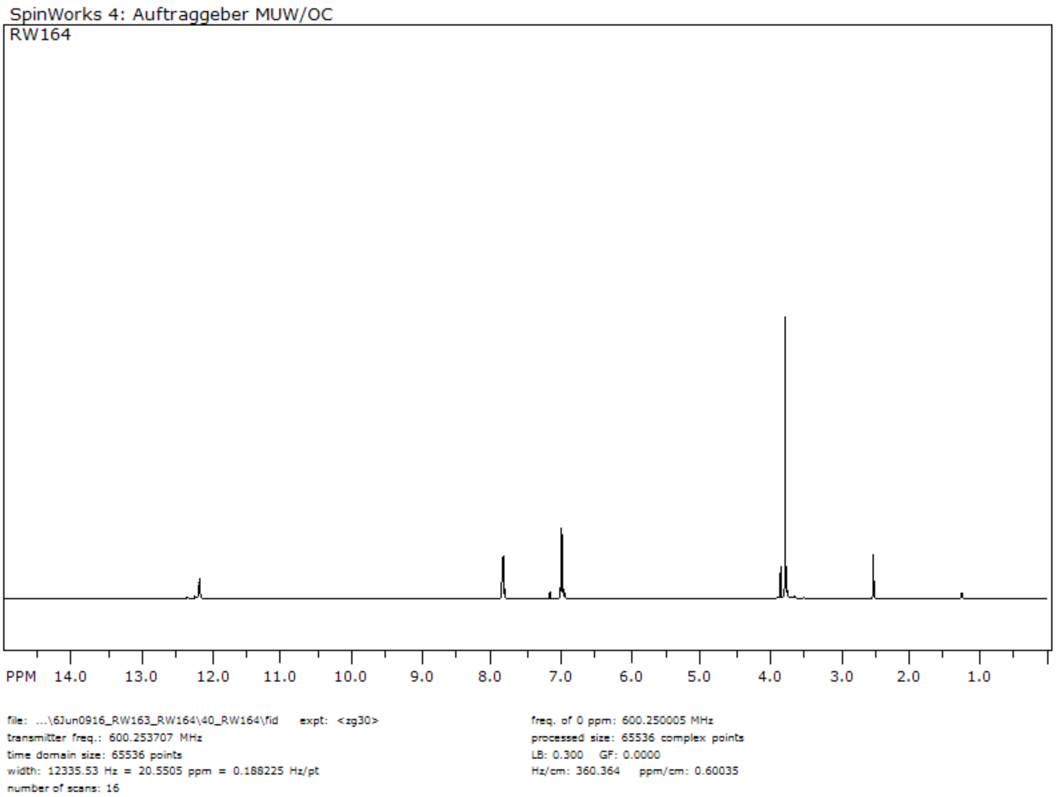

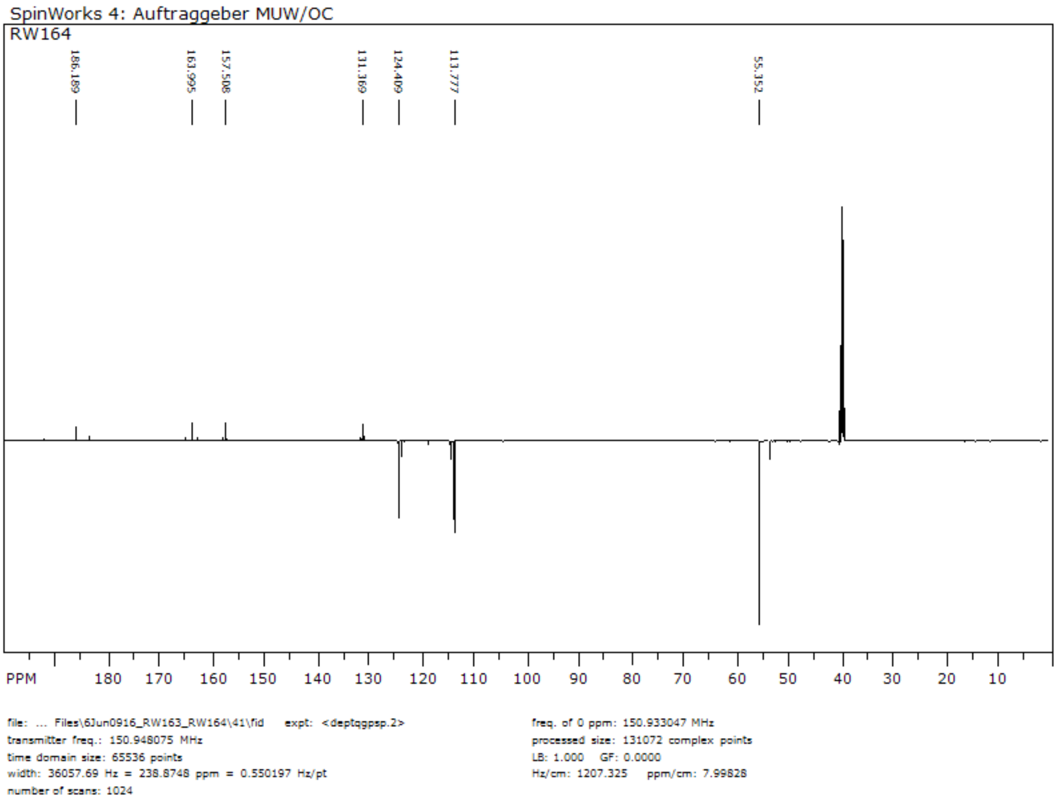

N[1]-(pyridin-2-yl)oxalamide (23)

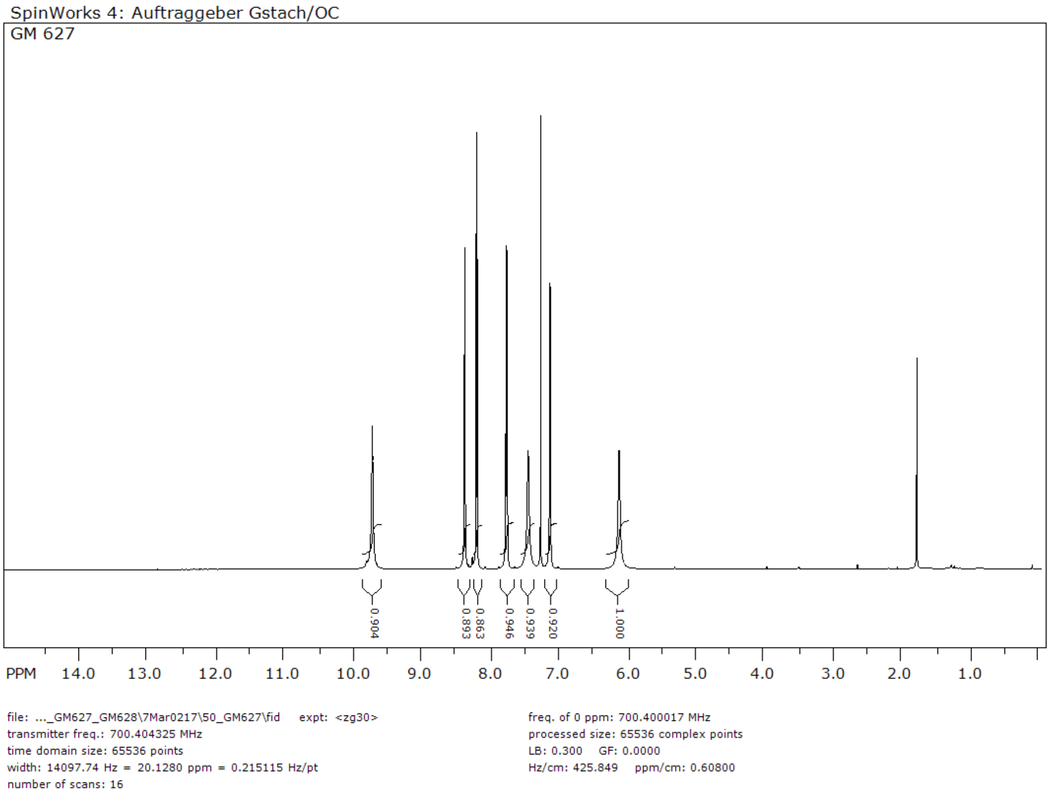

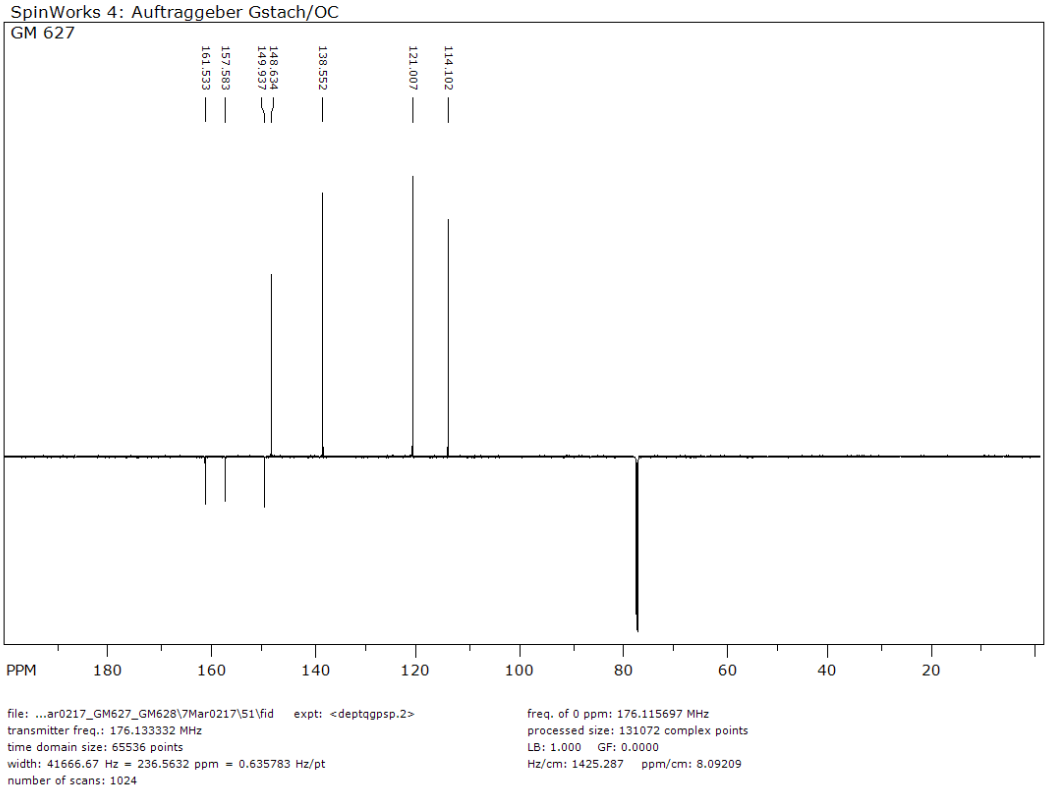

N^1-(naphthalen-2-yl)oxalamide (24)

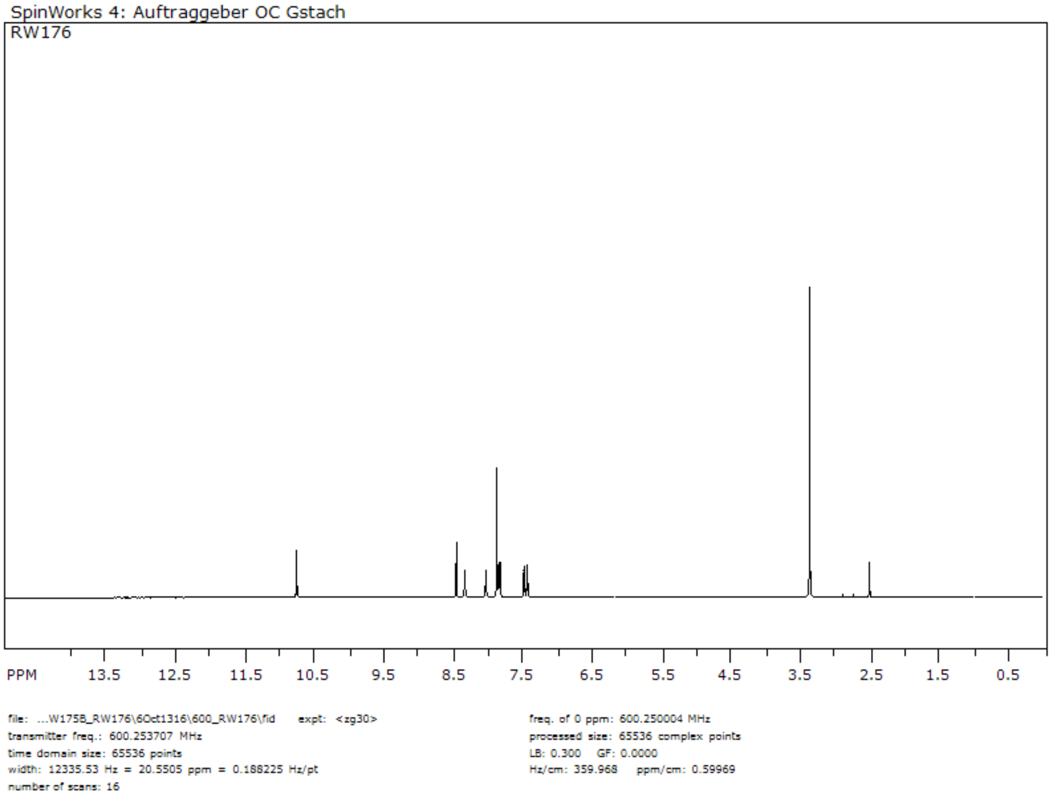

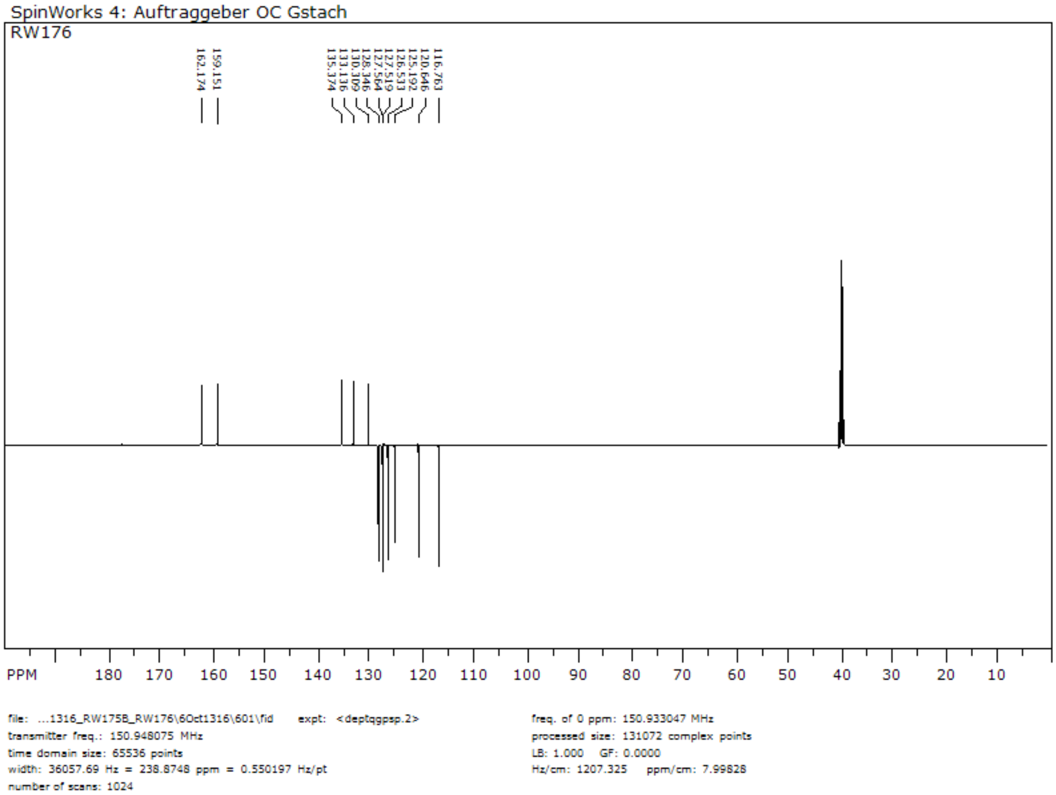

***N*-(6-methylpyridin-2-yl)-2-oxo-2-(pyrrolidin-1-yl)acetamide (25)**

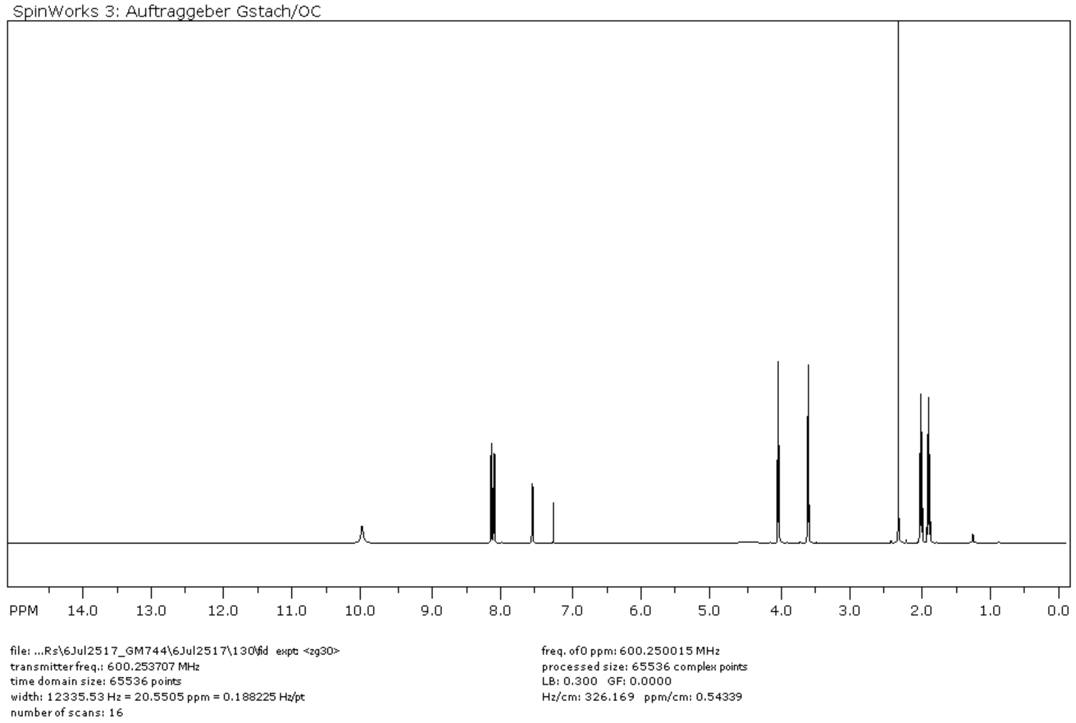

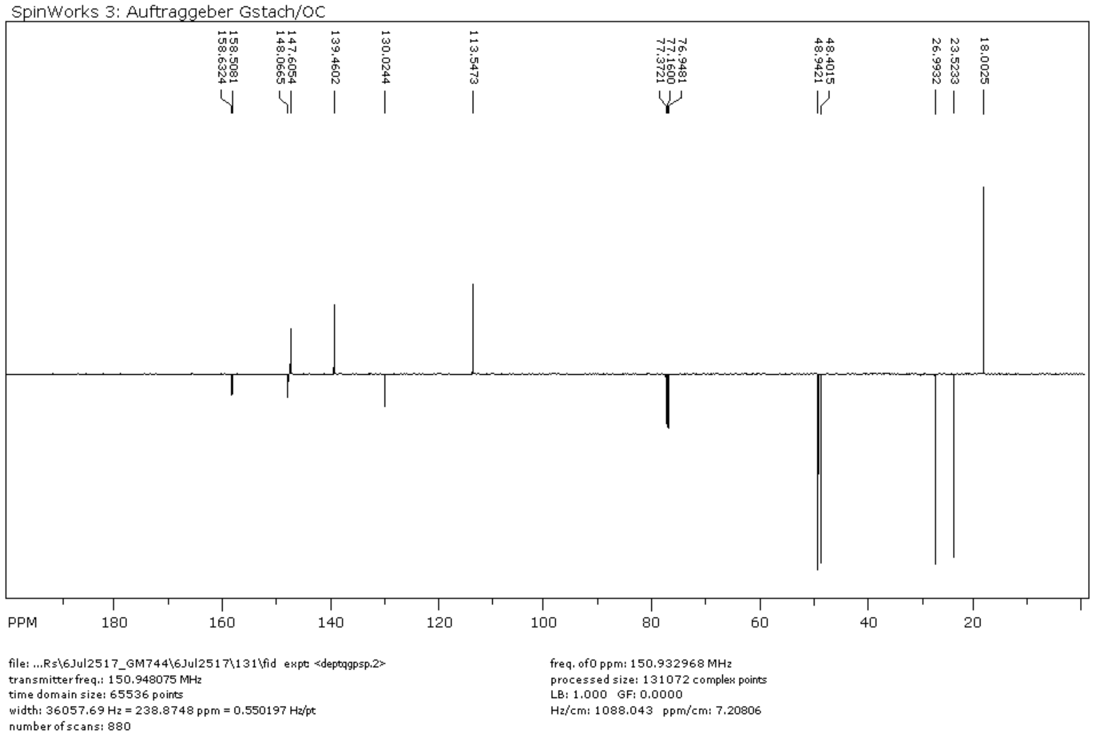

2-(2-*tert*-Butoxy-2-oxoacetamido)benzoic acid (26)

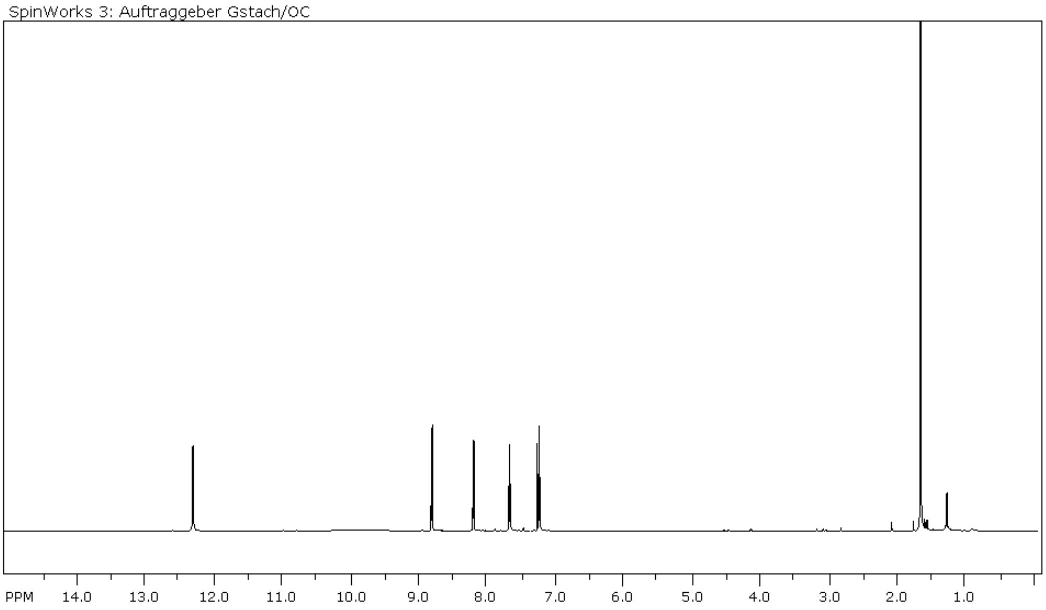

file: ..._GM697_GM698\6Jun0217\70_GM698\fid expt: <zg30>
transmitter freq.: 600.253707 MHz
time domain size: 65536 points
width: 12335.53 Hz = 20.5505 ppm = 0.188225 Hz/pt
number of scans: 16

freq. of 0 ppm: 600.250015 MHz
processed size: 65536 complex points
LB: 0.300 GF: 0.0000
Hz/cm: 325.791 ppm/cm: 0.54276

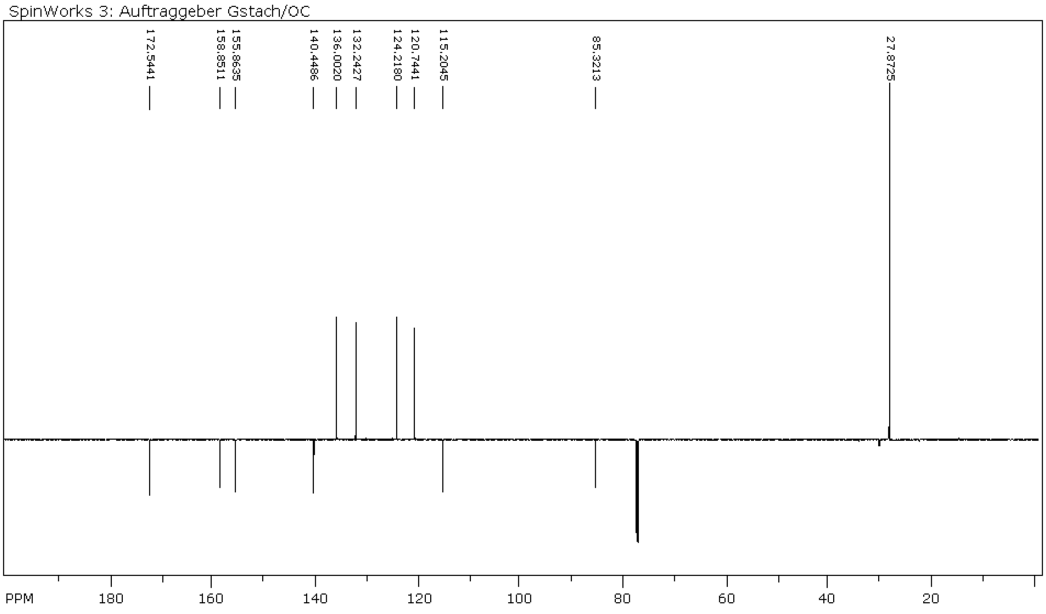

file: ...un0217_GM697_GM698\6Jun0217\71\fid expt: <deptqgpsp.2>
transmitter freq.: 150.948075 MHz
time domain size: 65536 points
width: 36057.69 Hz = 238.8748 ppm = 0.550197 Hz/pt
number of scans: 464

freq. of 0 ppm: 150.932964 MHz
processed size: 131072 complex points
LB: 1.000 GF: 0.0000
Hz/cm: 1091.353 ppm/cm: 7.22999

3-(2-*tert*-Butoxy-2-oxoacetamido)benzoic acid (27)

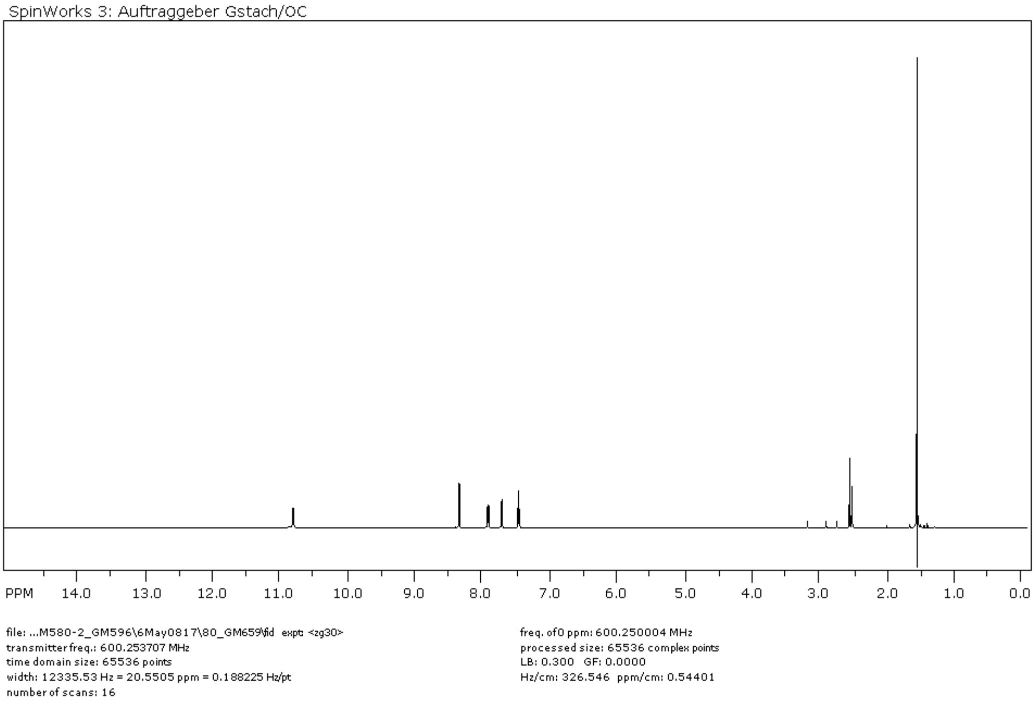

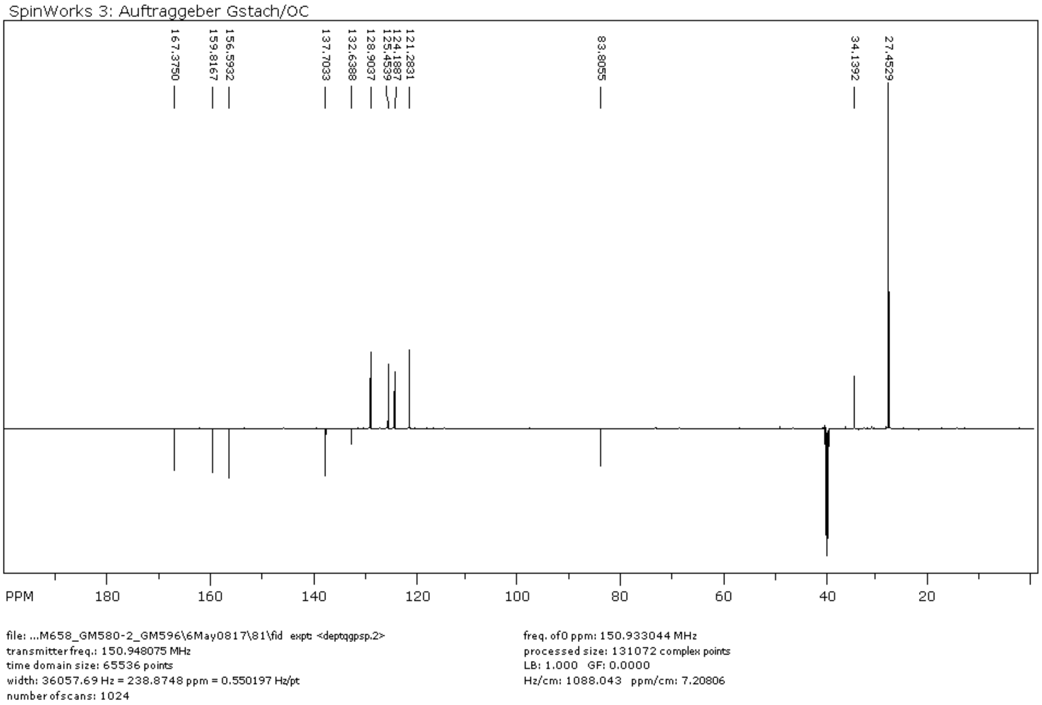

4-(2-*tert*-Butoxy-2-oxoacetamido)benzoic acid (28)

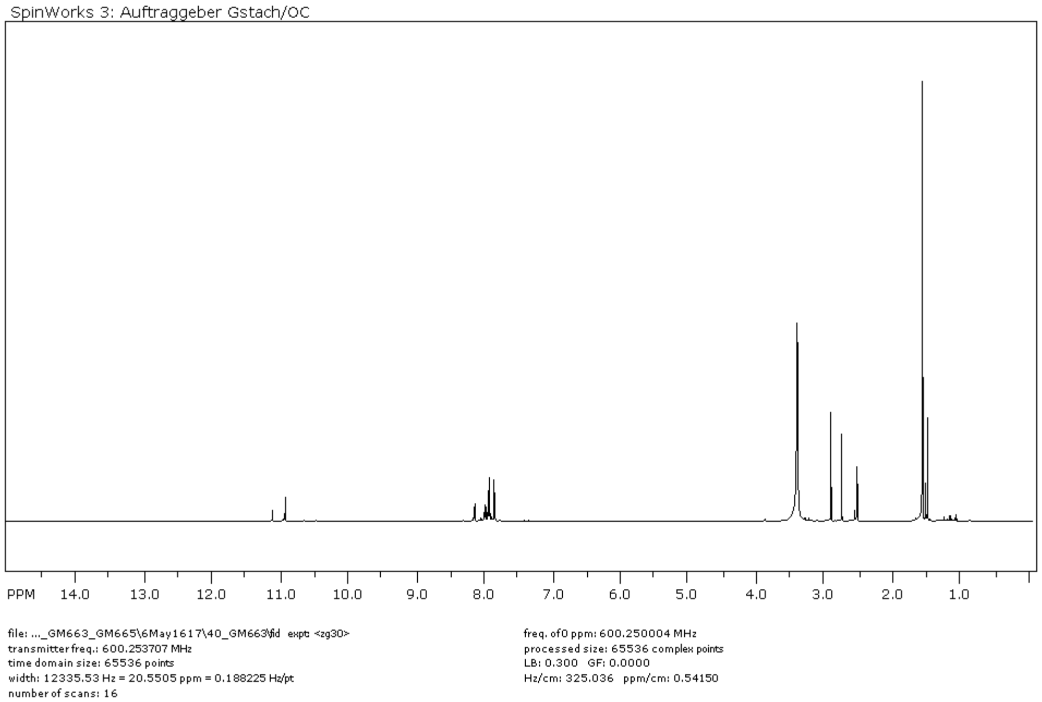

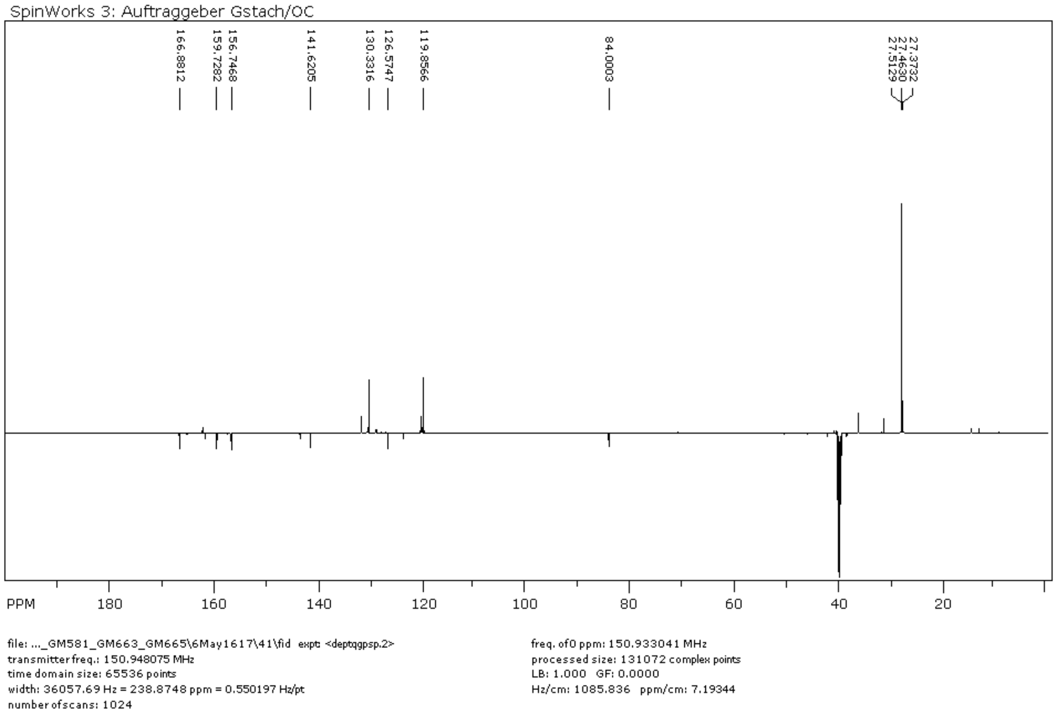

N^1,N^2-bis(5-methylpyridin-2-yl)oxalamide (29)

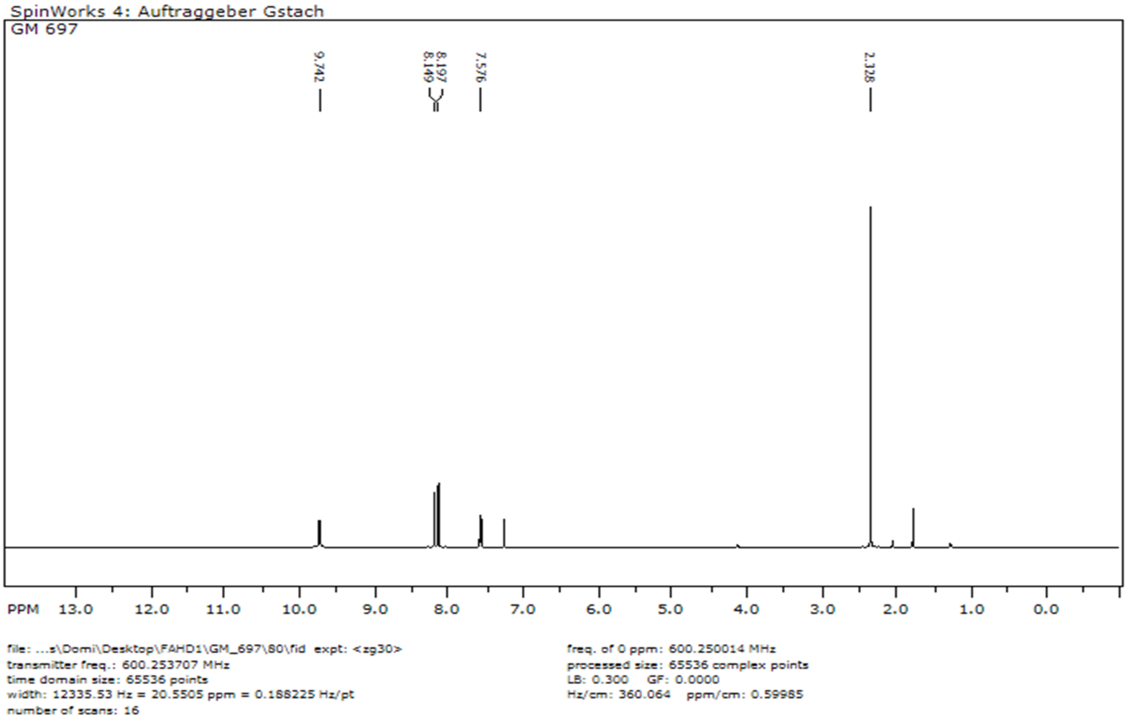

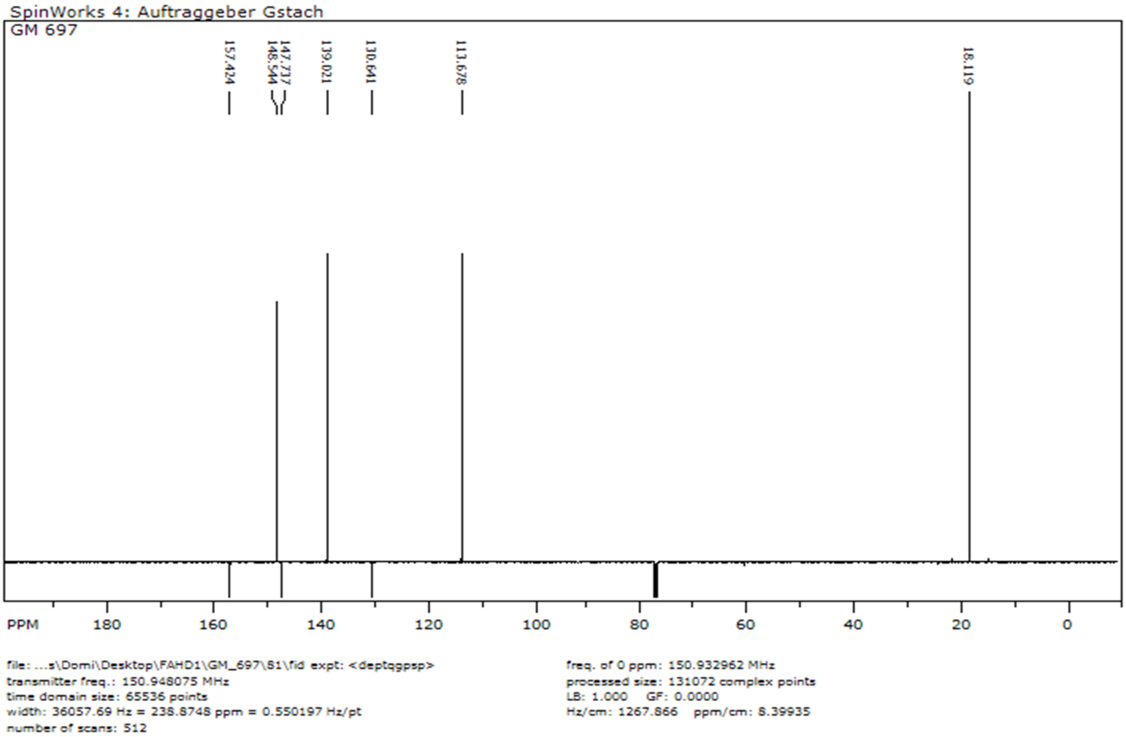

N^1,N^2-di(pyridin-2-yl)oxalamide (30)

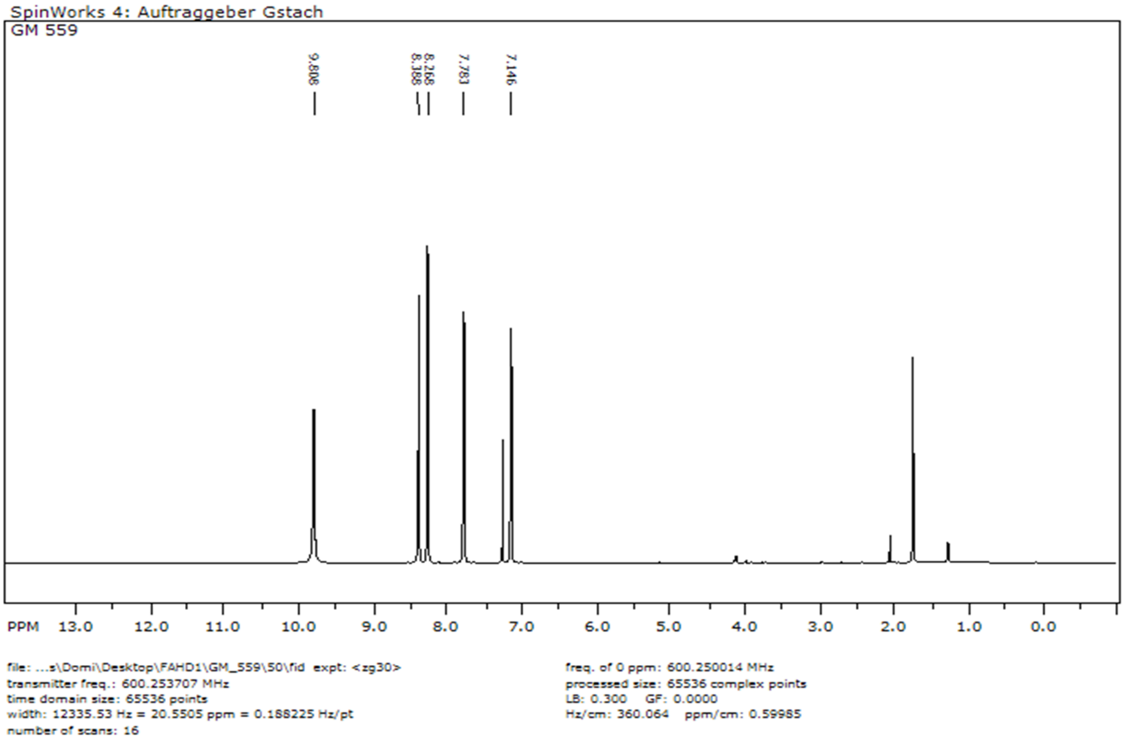

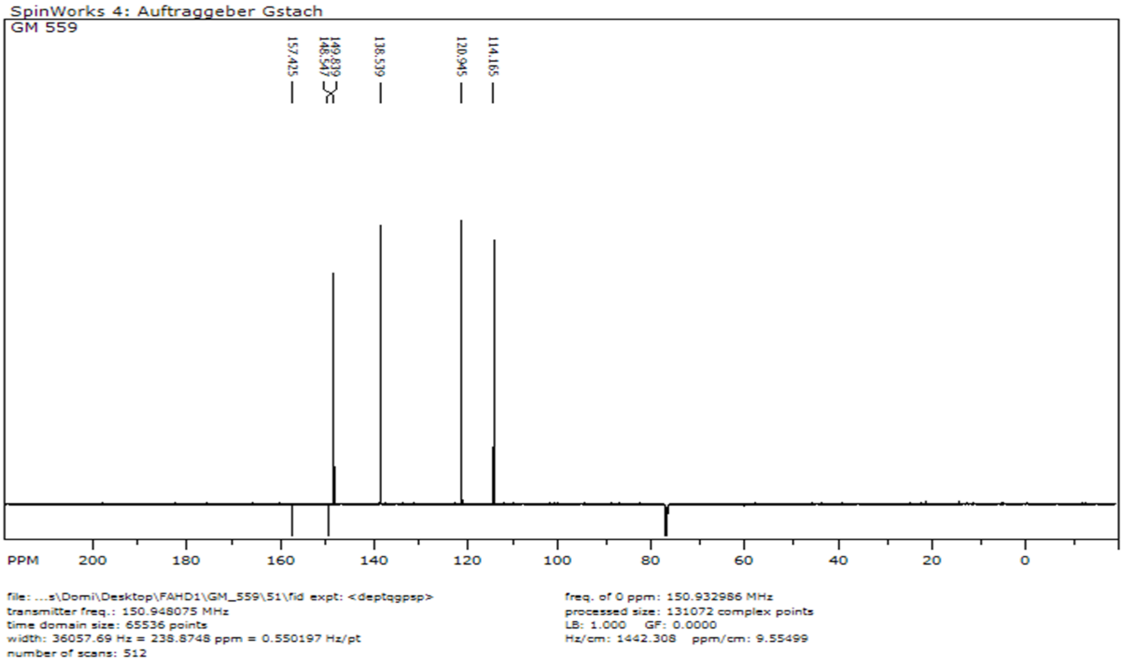

_N,N′_-Di-pyridin-3-yl-oxalamide (31)

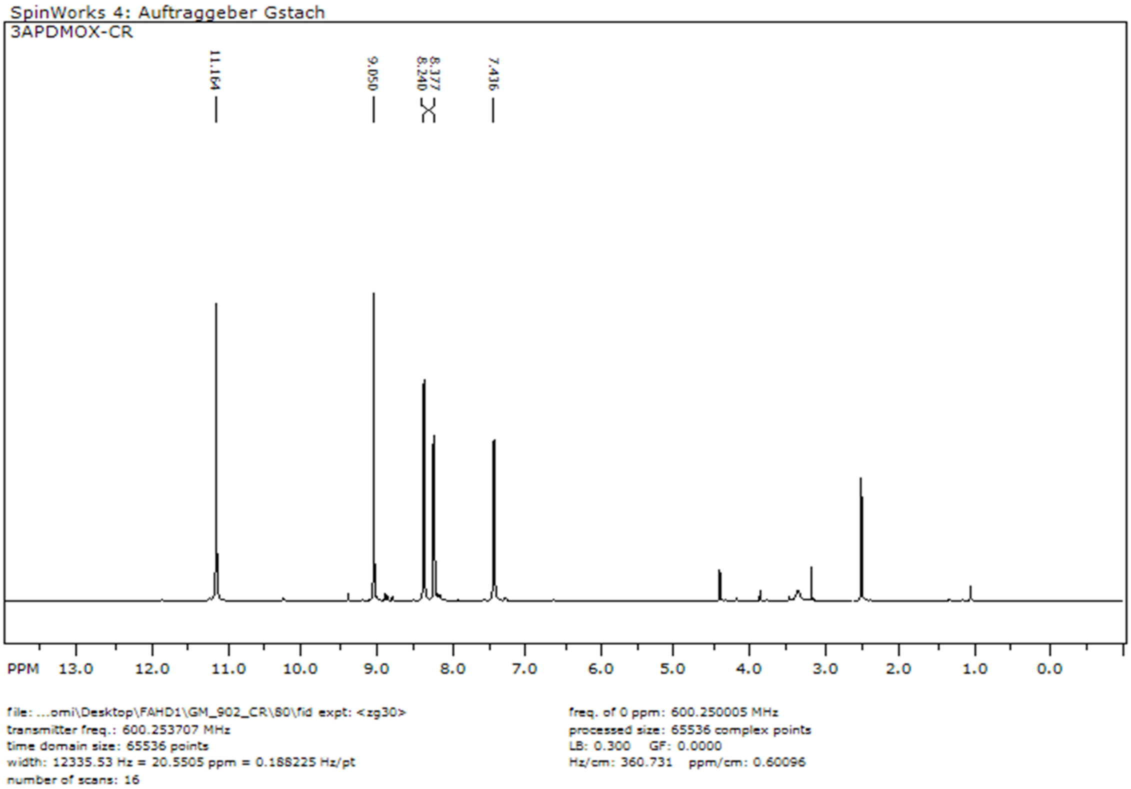

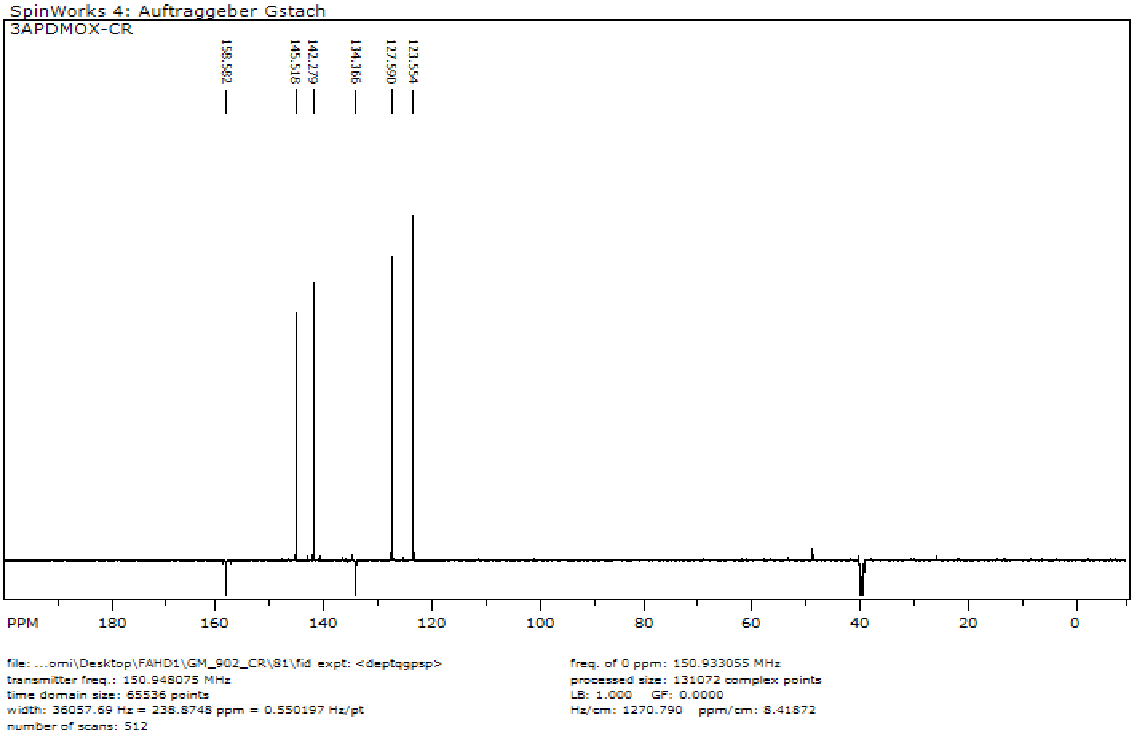

N-Pyridin-2-yl-N′-p-tolyl-oxalamide (32)

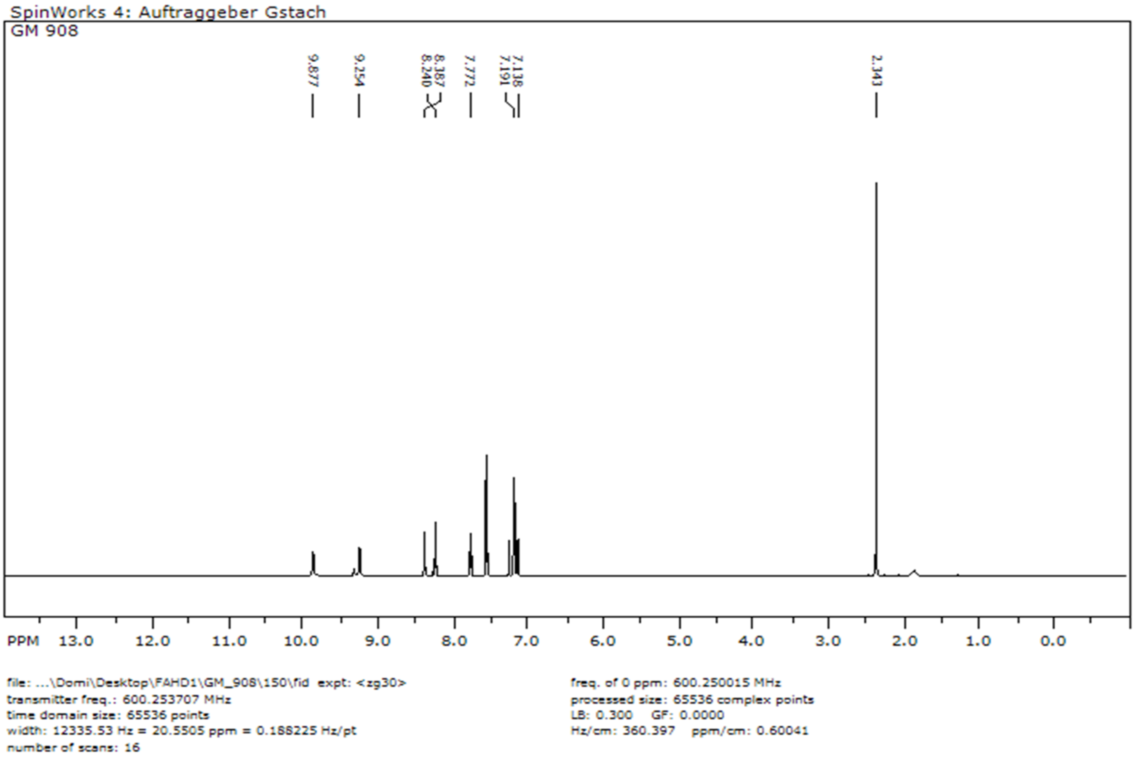

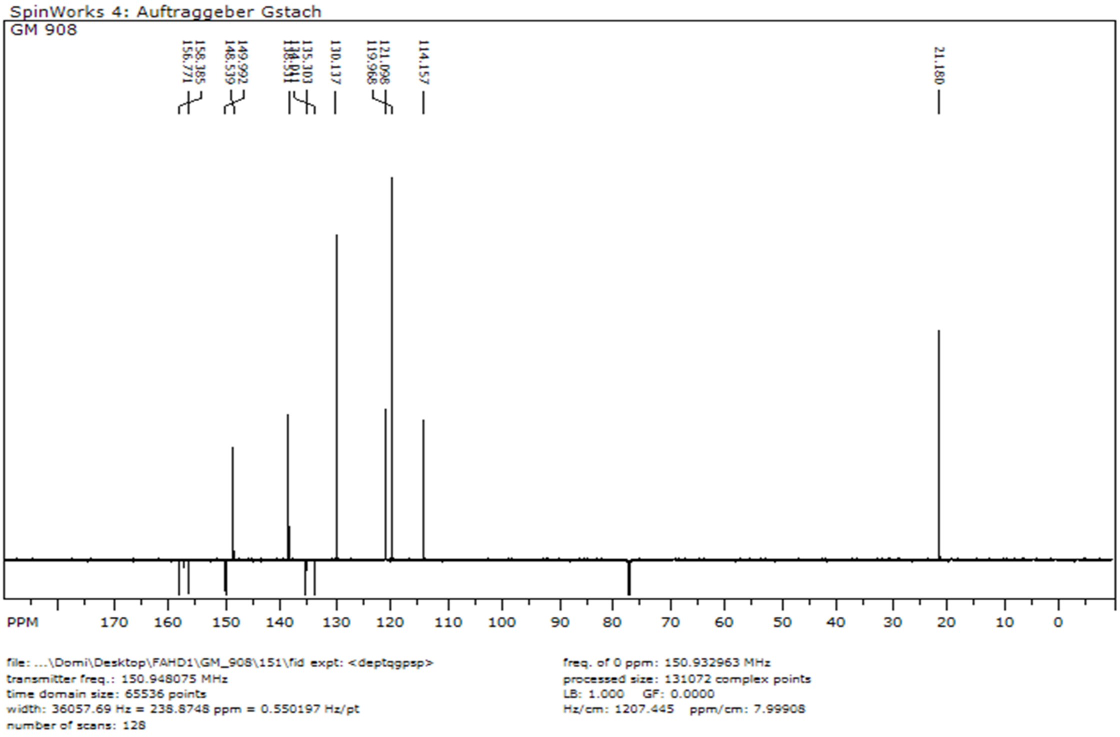

_N_-Pyridin-2-yl-_N′_-_m_-tolyl-oxalamide (33)

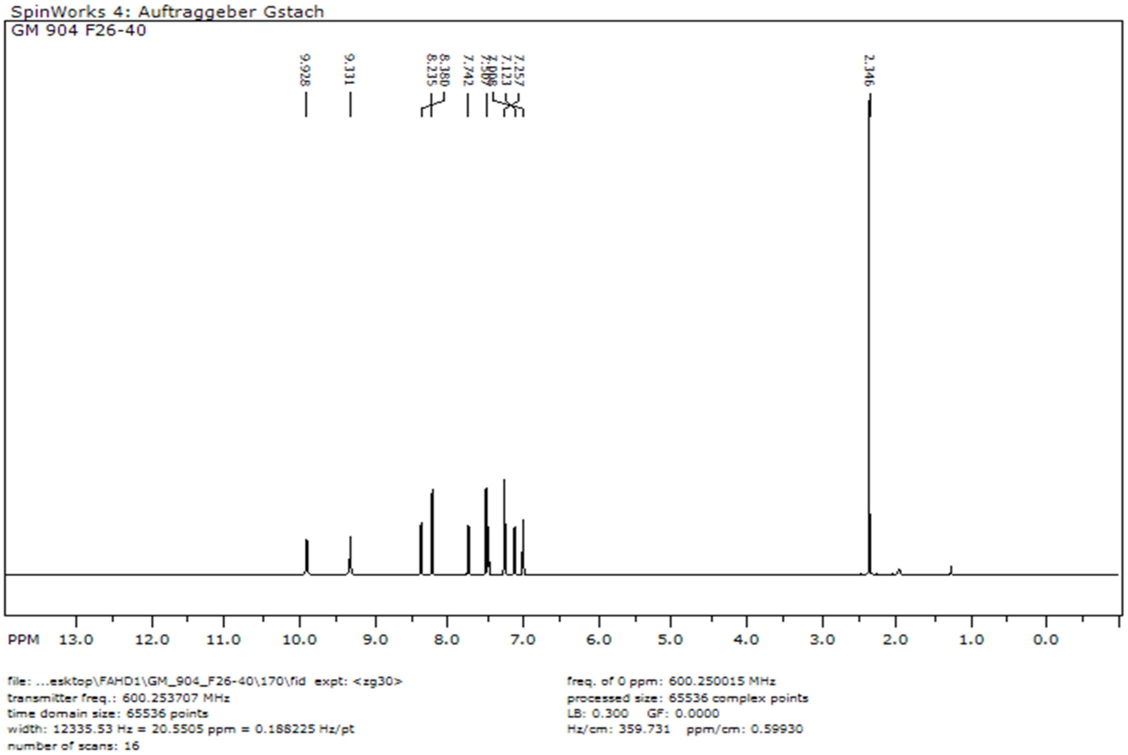

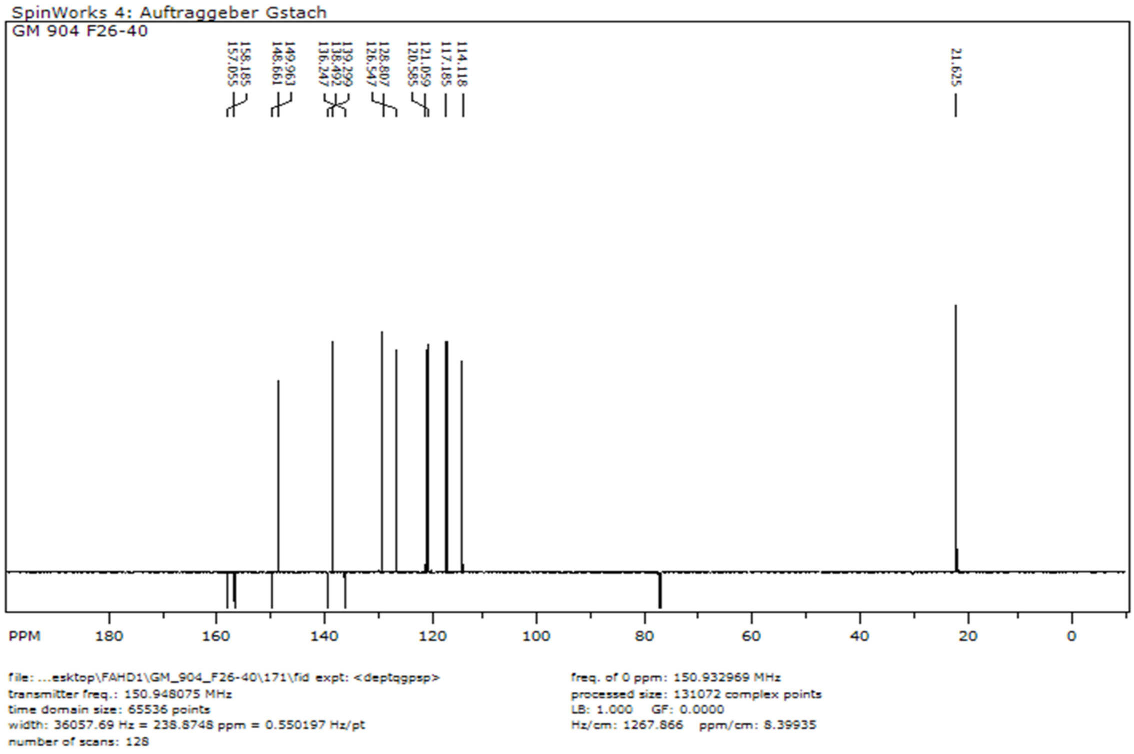

***N*-Pyridin-3-yl-*N′*-pyridin-2-yl-oxalamide (34)**

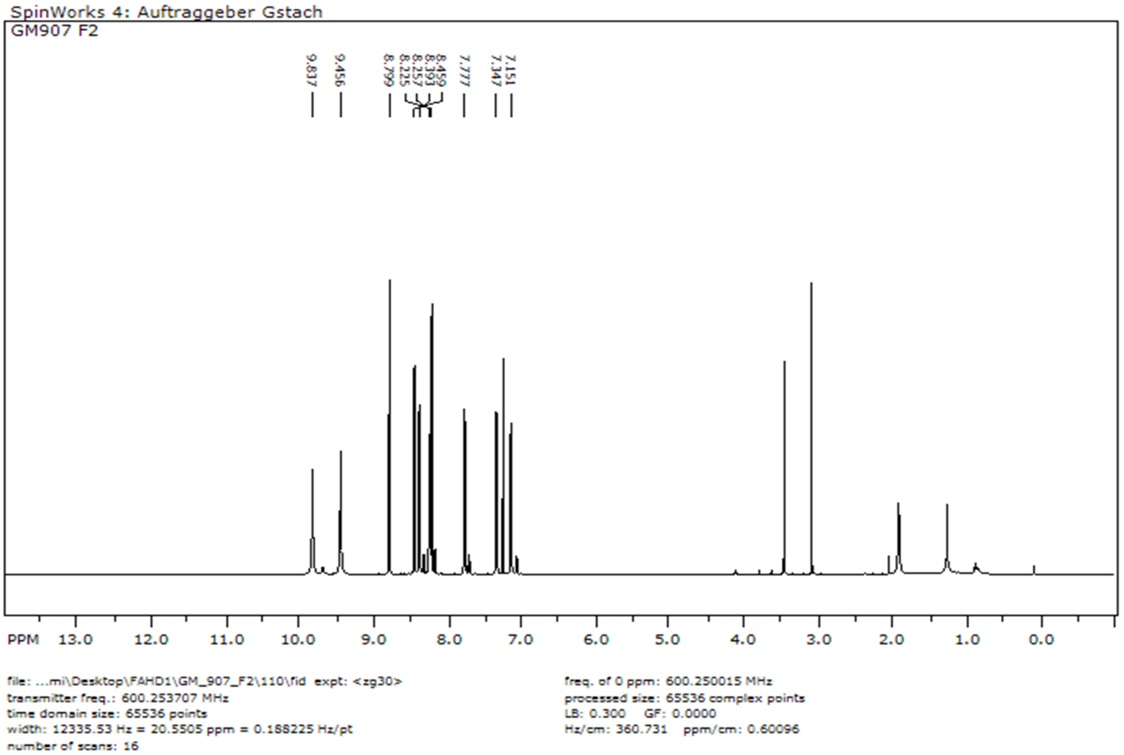

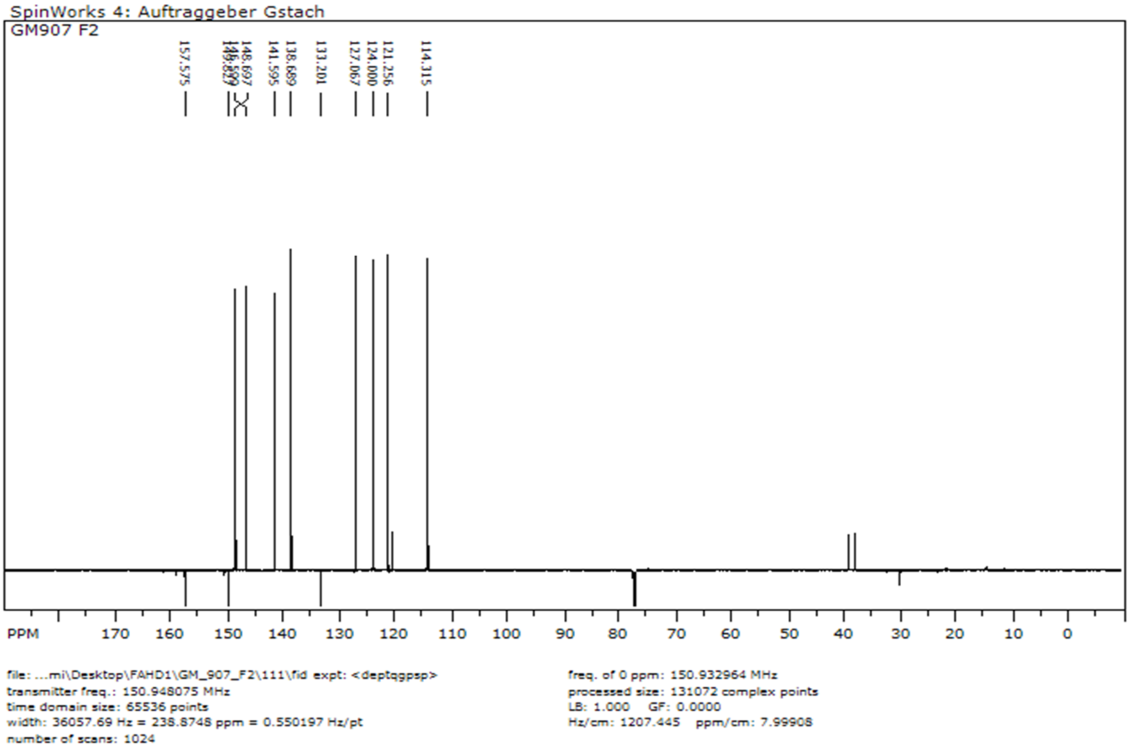

N-(1-Oxy-pyridin-2-yl)-*N′*-*p*-tolyl-oxalamide (35)

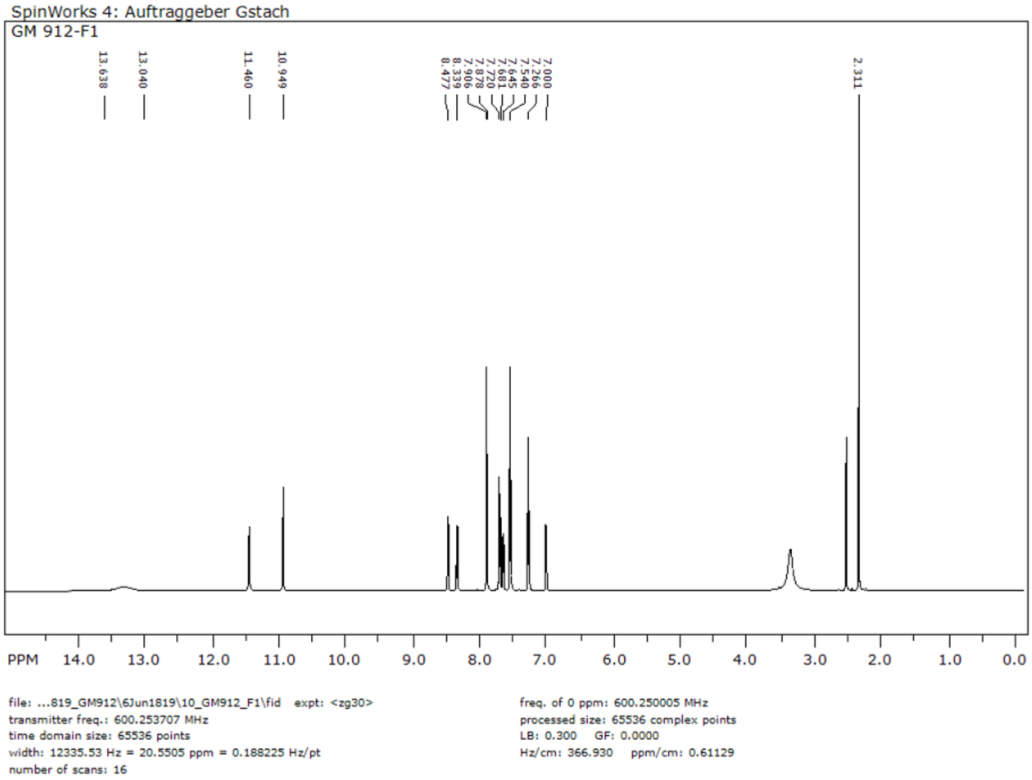

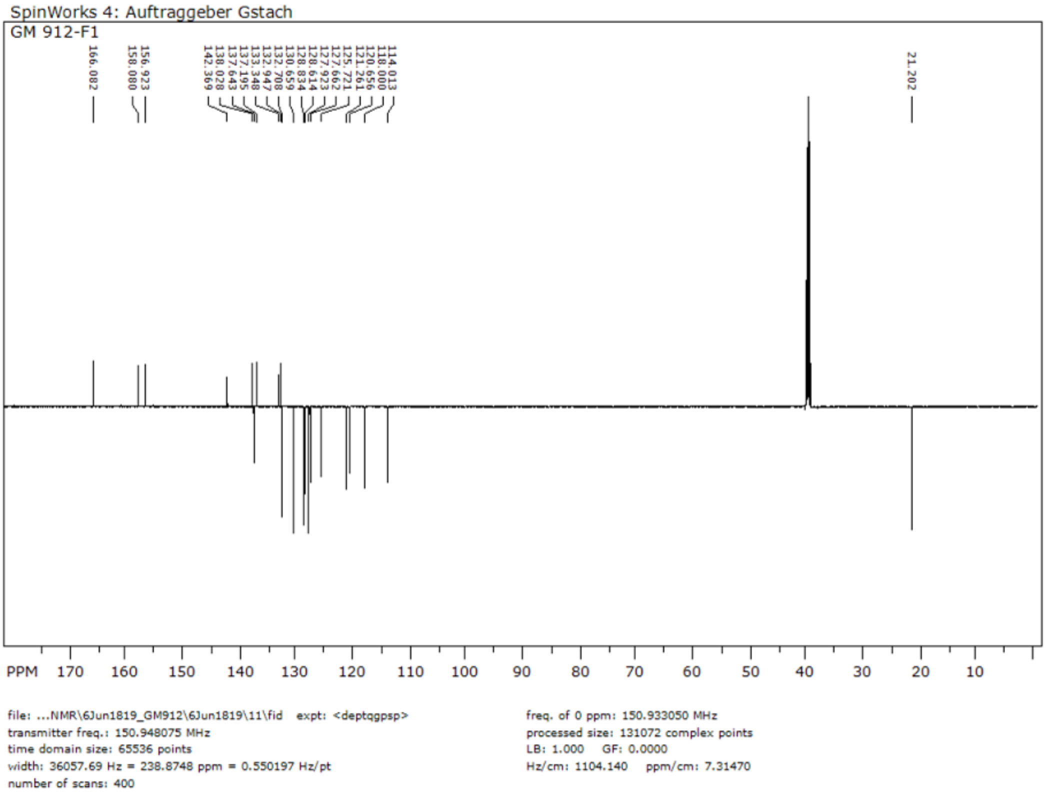

N-(1-Oxy-pyridin-2-yl)-*N′*-*m*-tolyl-oxalamide (36)

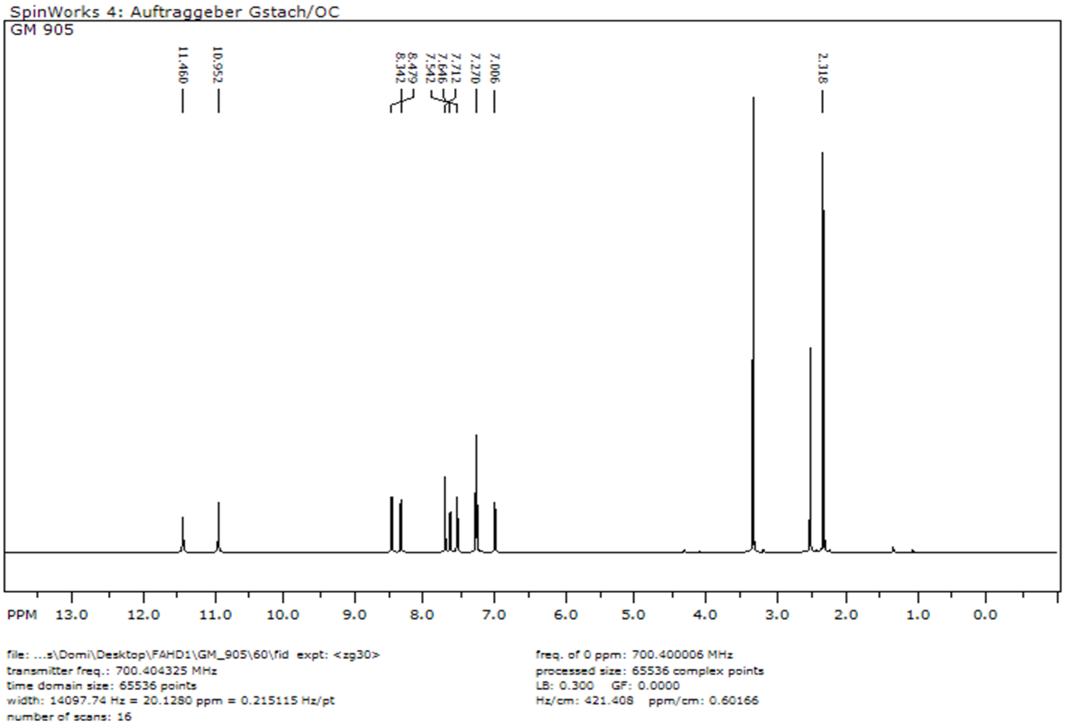

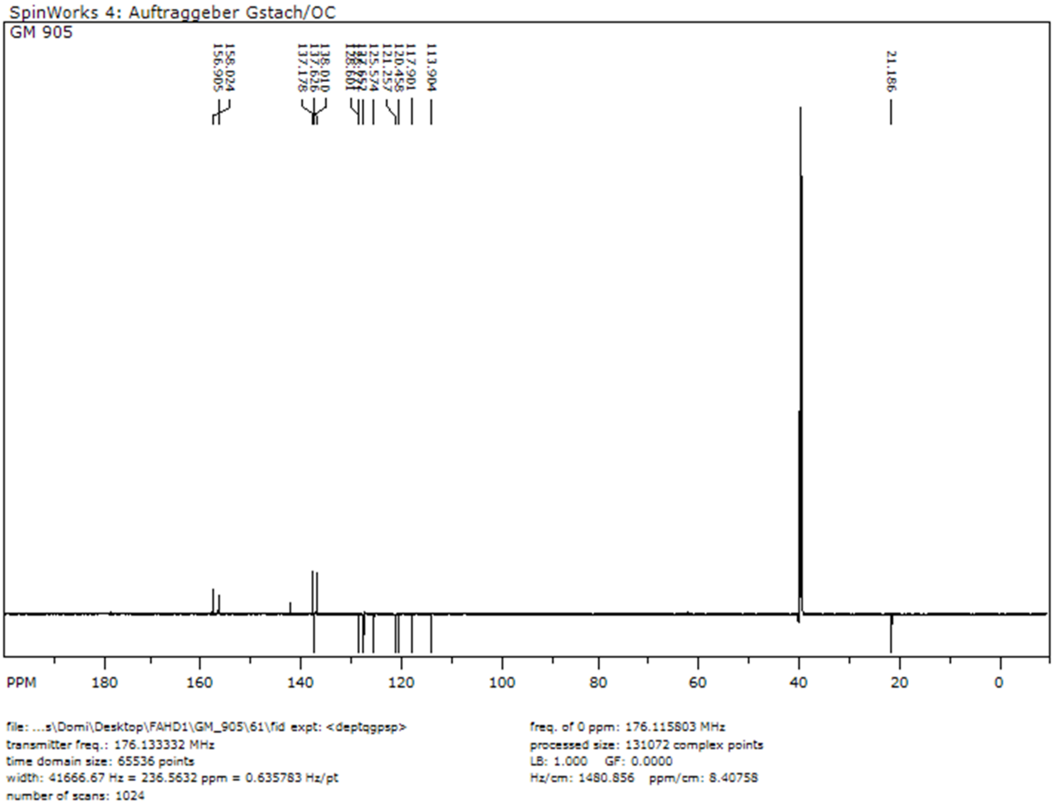

N-(1-Oxy-pyridin-3-yl)-N′-m-tolyl-oxalamide (37)

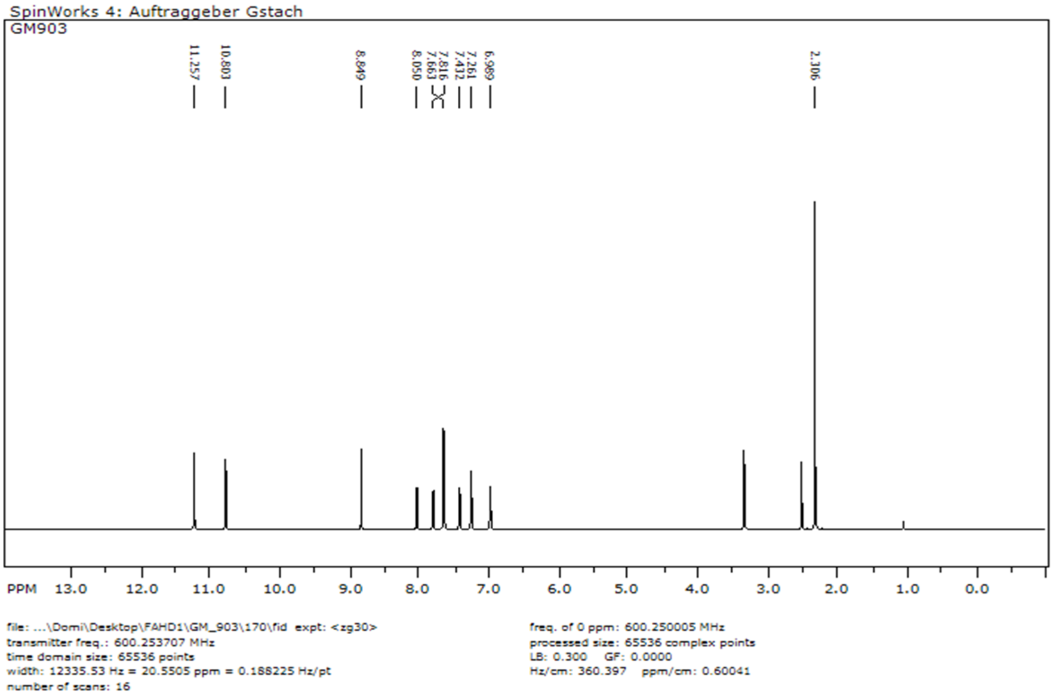

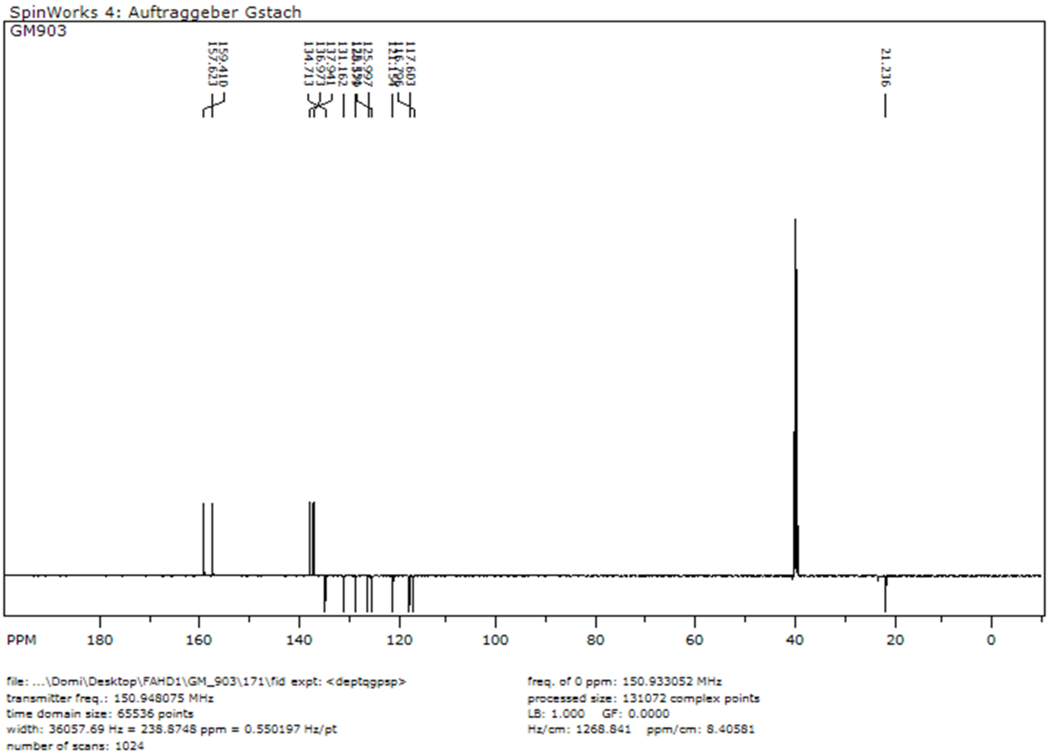

Methyl 2-((6-methylpyridin-2-yl)amino)-2-oxoacetate (S1)

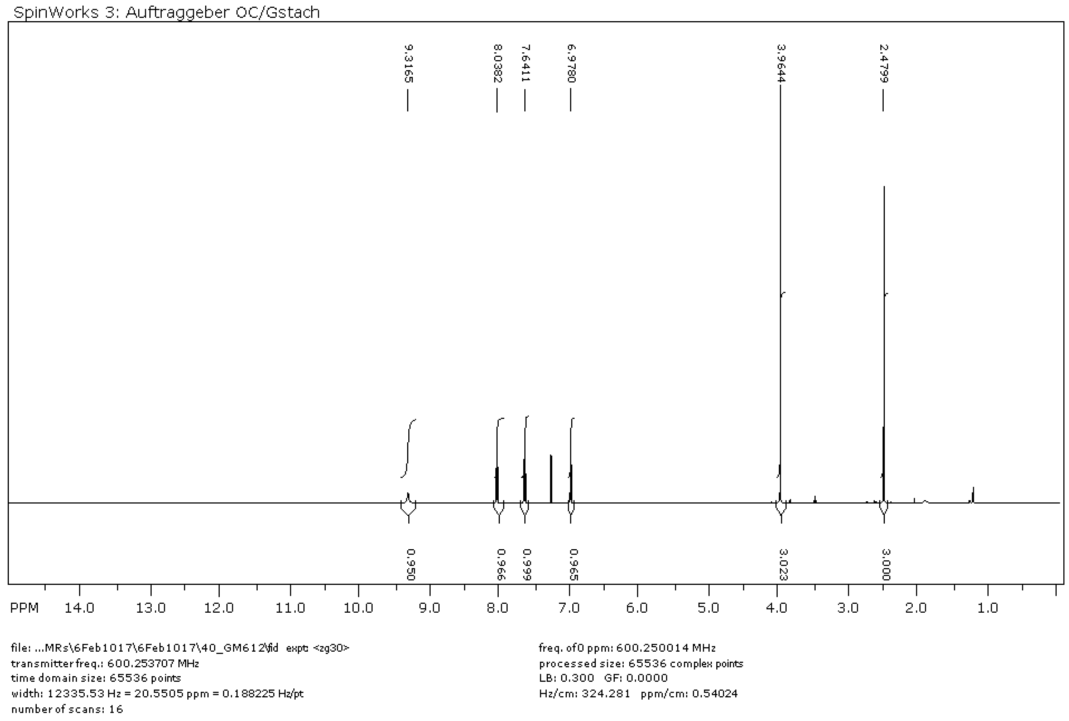

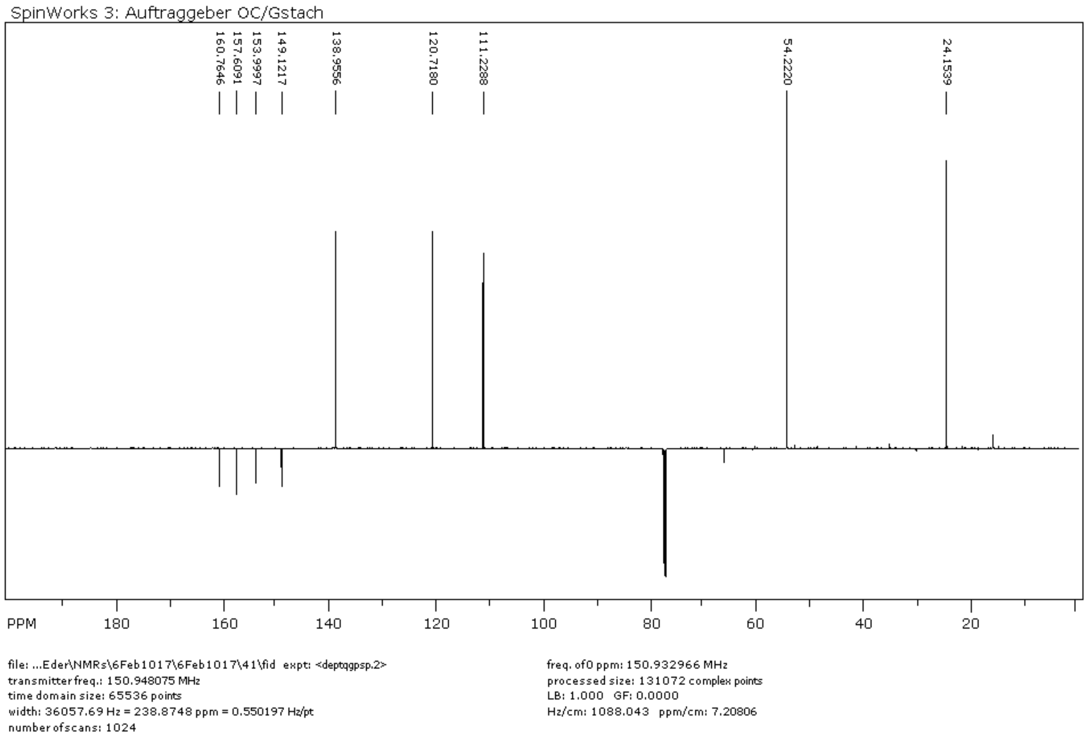

Methyl 2-((2-methoxyphenyl)amino)-2-oxoacetate (S2)

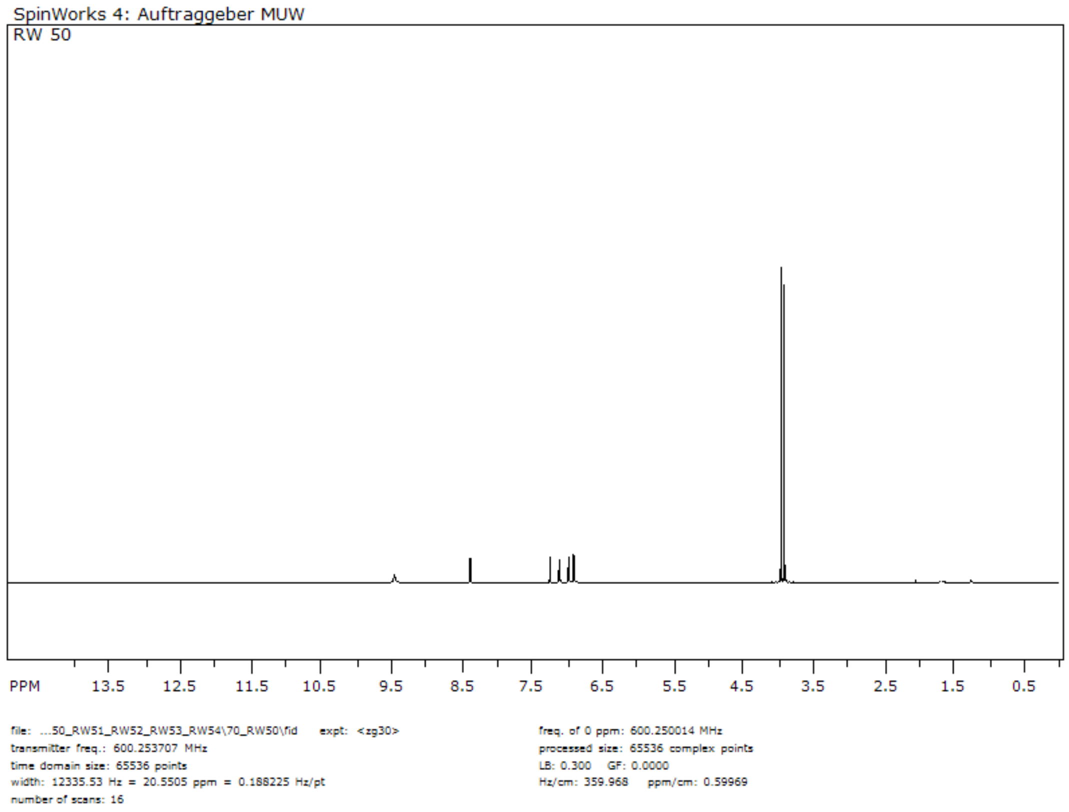

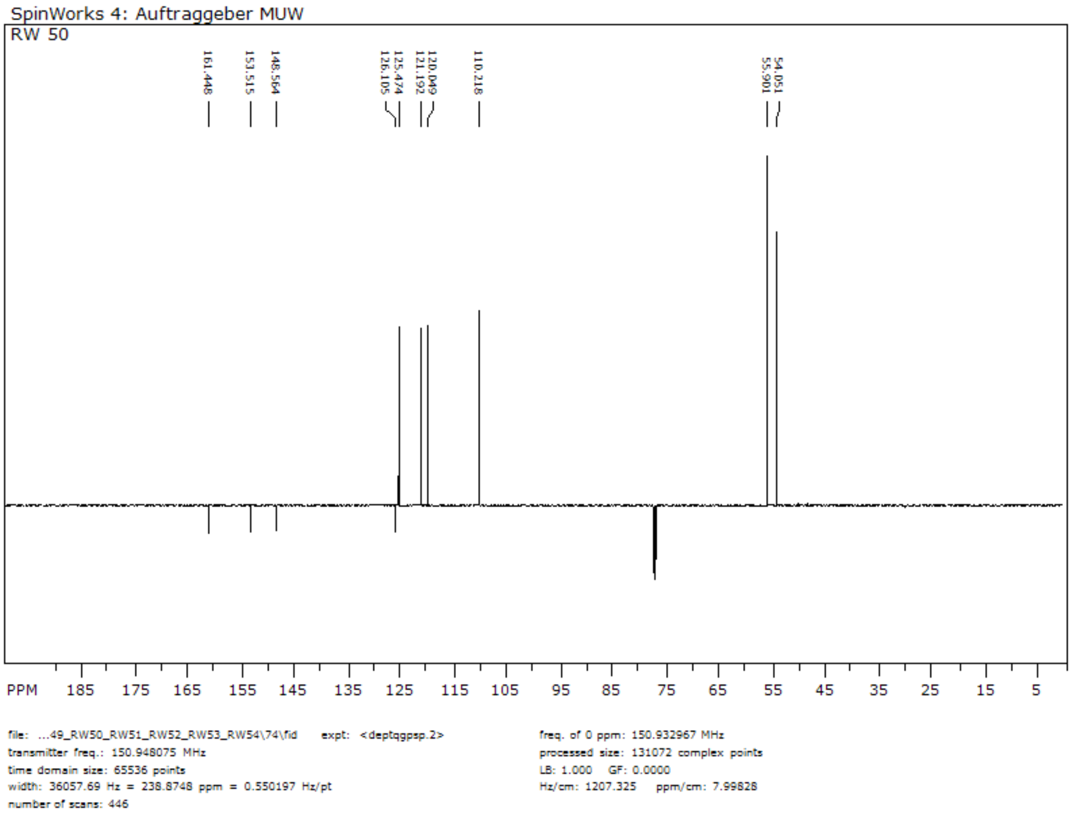

Methyl 2-((3-methoxyphenyl)amino)-2-oxoacetate (S3)

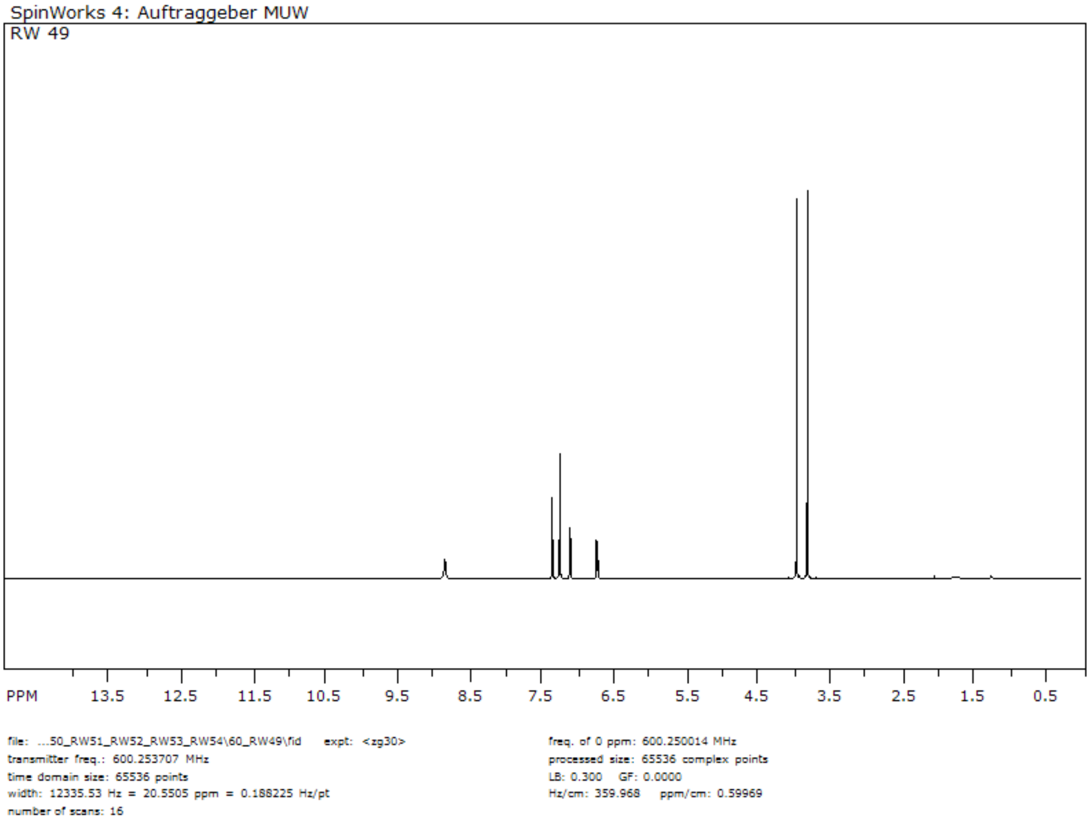

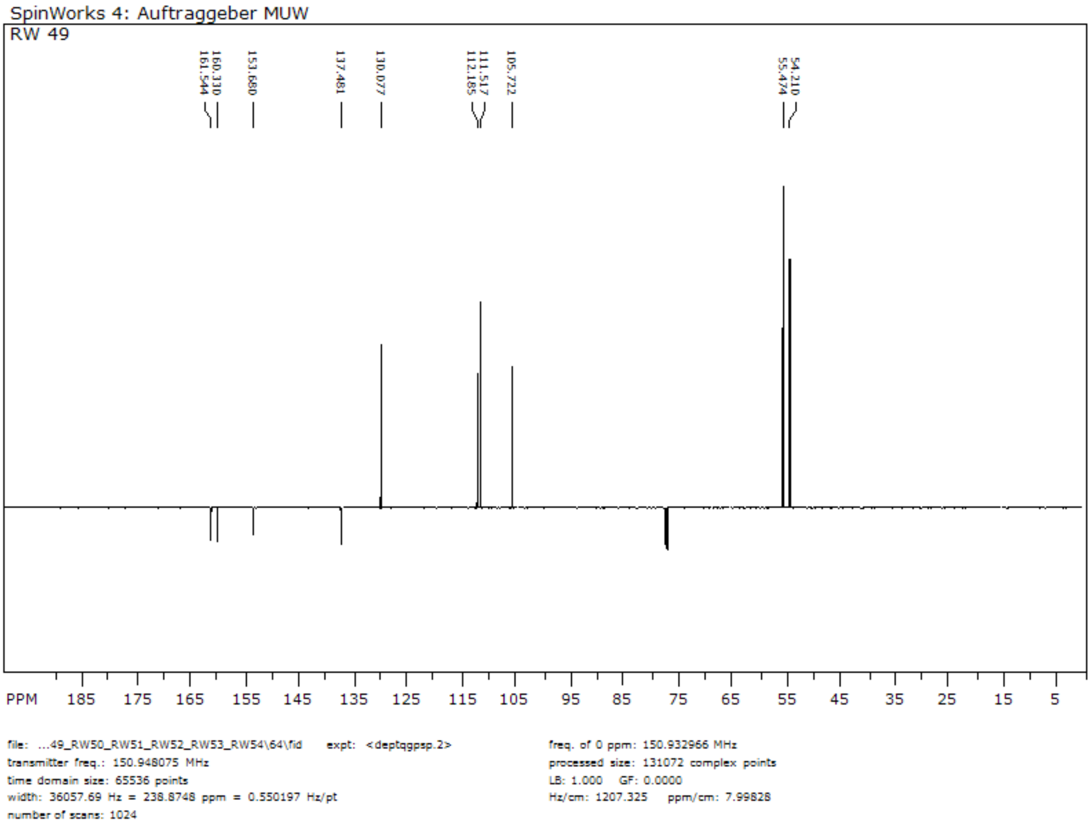

Methyl 2-((4-methoxyphenyl)amino)-2-oxoacetate (S4)

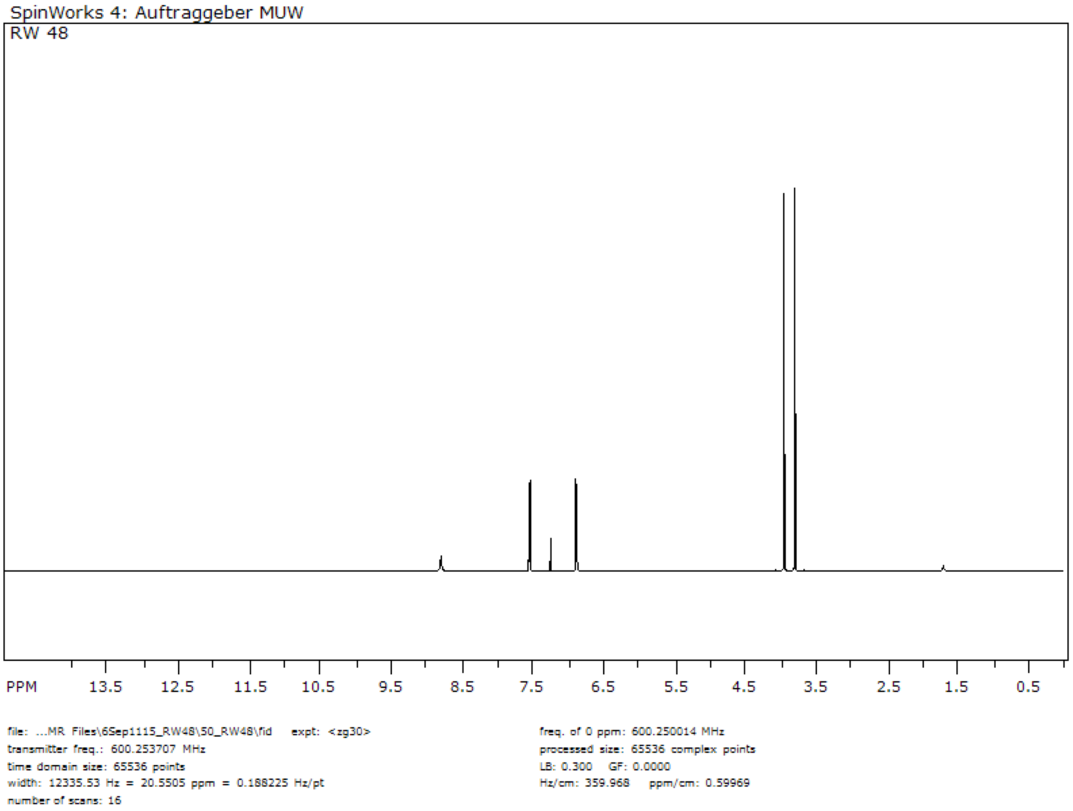

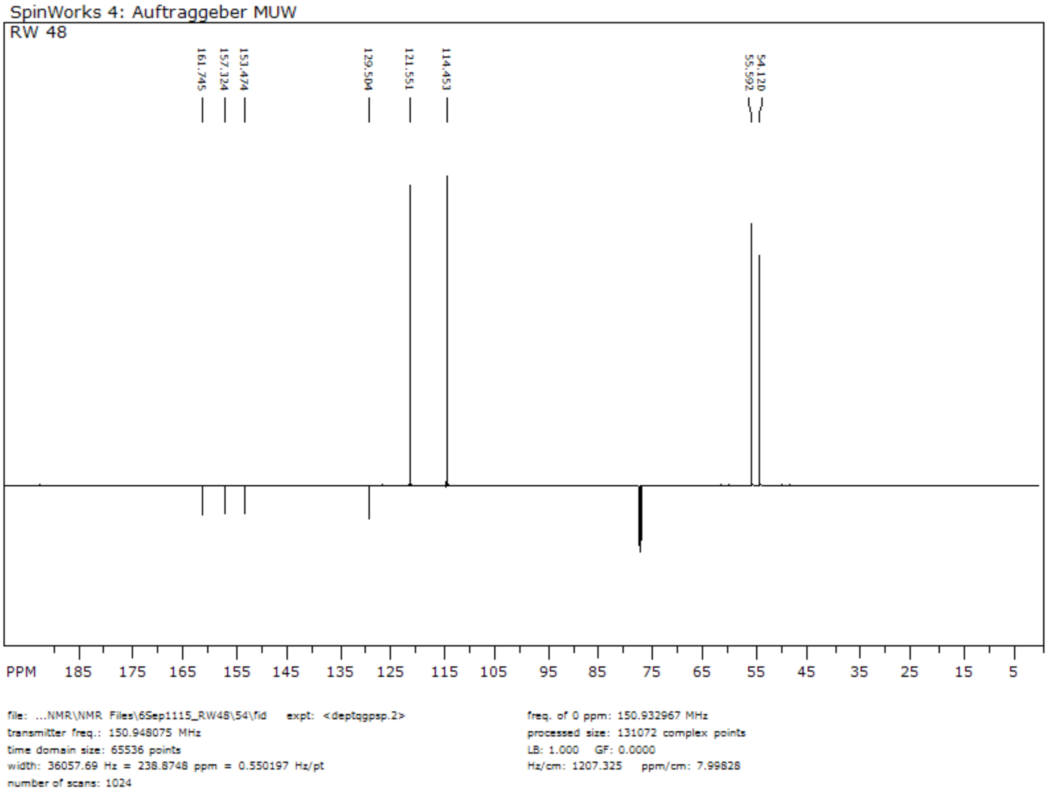

2-oxo-2-(*o*-tolylamino)acetic acid (S5)

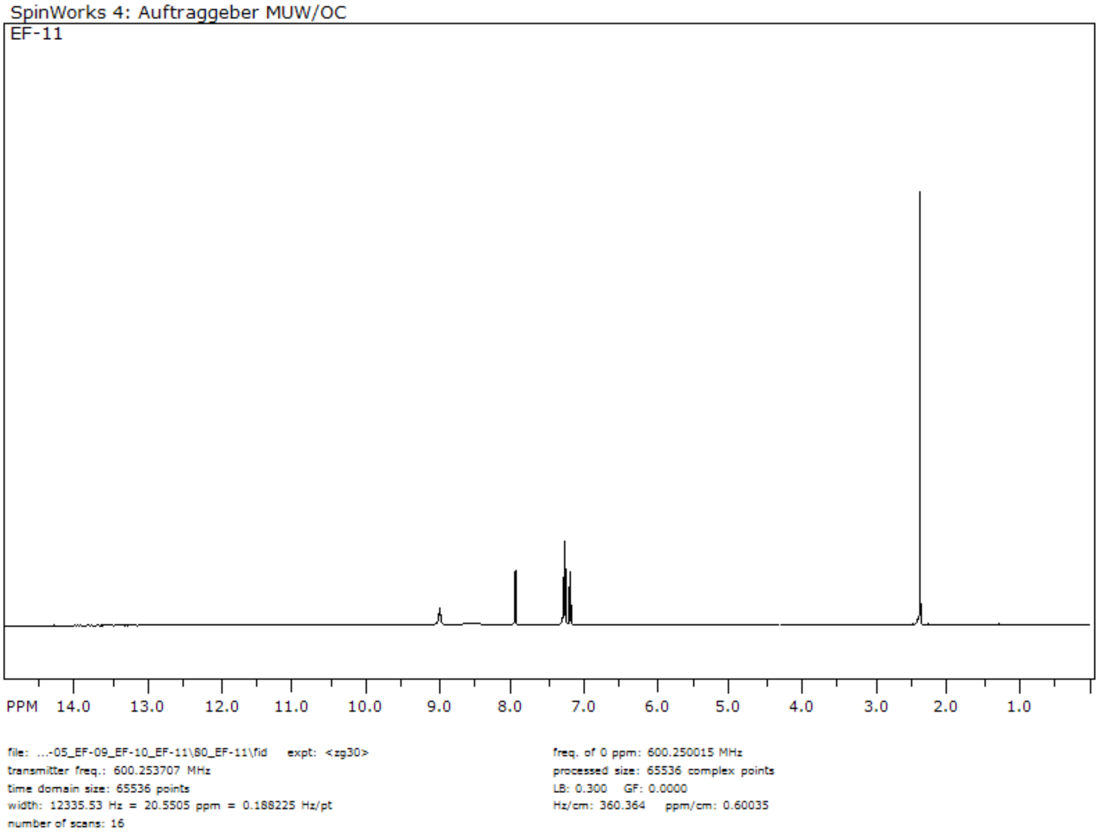

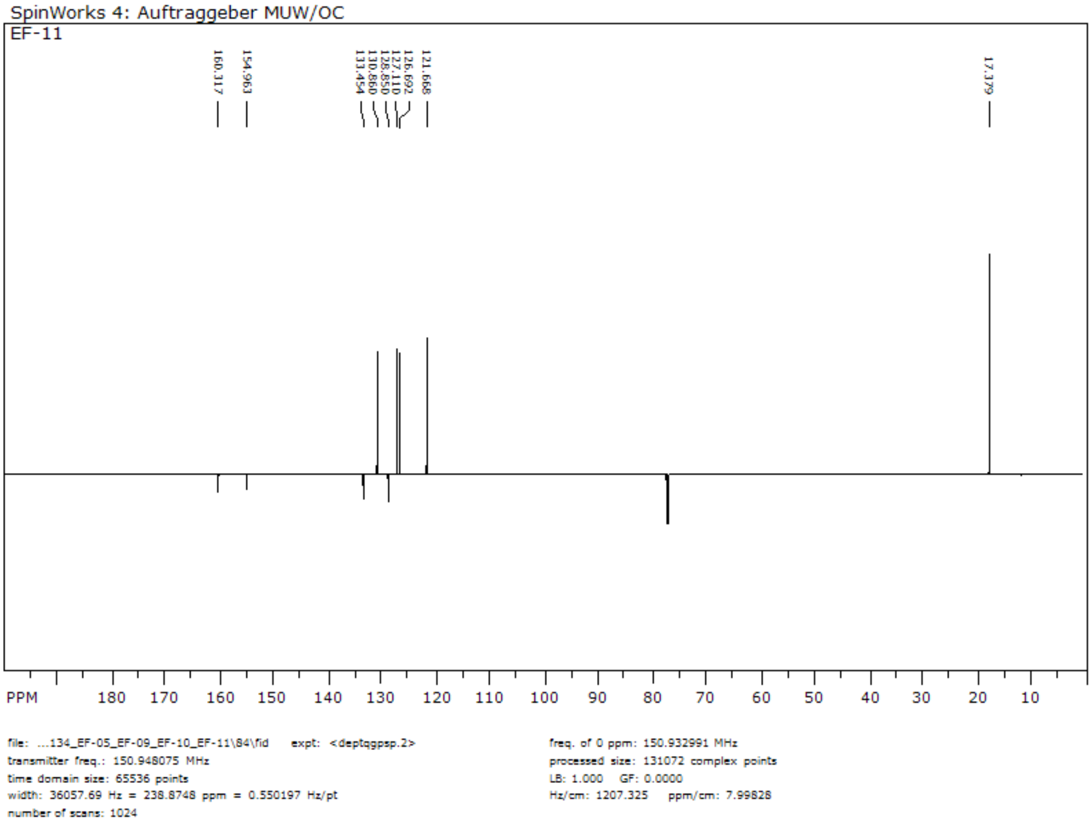

2-(((1*R*,2*S*)-2-hydroxy-2,3-dihydro-1*H*-inden-1-yl)amino)-2-oxoacetic acid (S6)

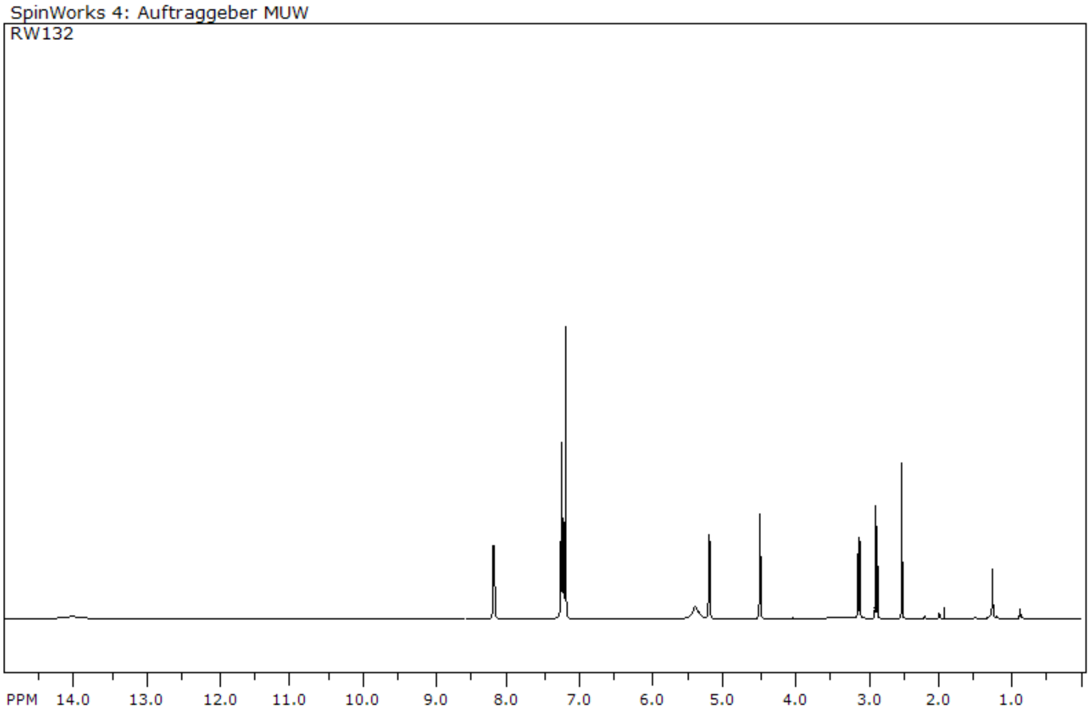

file: ...MR\NMR Files\6Feb1016_RW132\40\fid expt: <zg30>
transmitter freq.: 600.253707 MHz
time domain size: 65536 points
width: 12335.53 Hz = 20.5505 ppm = 0.188225 Hz/pt
number of scans: 16

freq. of 0 ppm: 600.250005 MHz
processed size: 65536 complex points
LB: 0.300 GF: 0.0000
Hz/cm: 360.364 ppm/cm: 0.60035

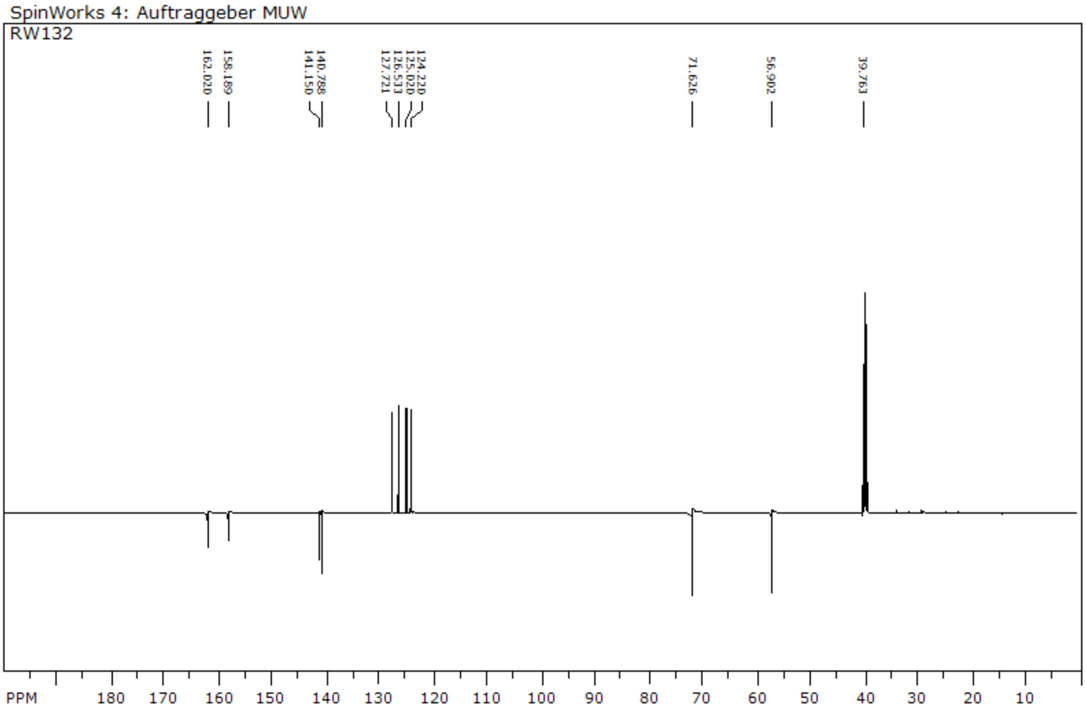

file: ...MR\NMR Files\6Feb1016_RW132\41\fid expt: <deptqgpsp.2>
transmitter freq.: 150.948075 MHz
time domain size: 65536 points
width: 36057.69 Hz = 238.8748 ppm = 0.550197 Hz/pt
number of scans: 1024

freq. of 0 ppm: 150.933048 MHz
processed size: 131072 complex points
LB: 1.000 GF: 0.0000
Hz/cm: 1207.325 ppm/cm: 7.99828

Methyl 2-(*tert*-butylamino)-2-oxoacetate (S7)

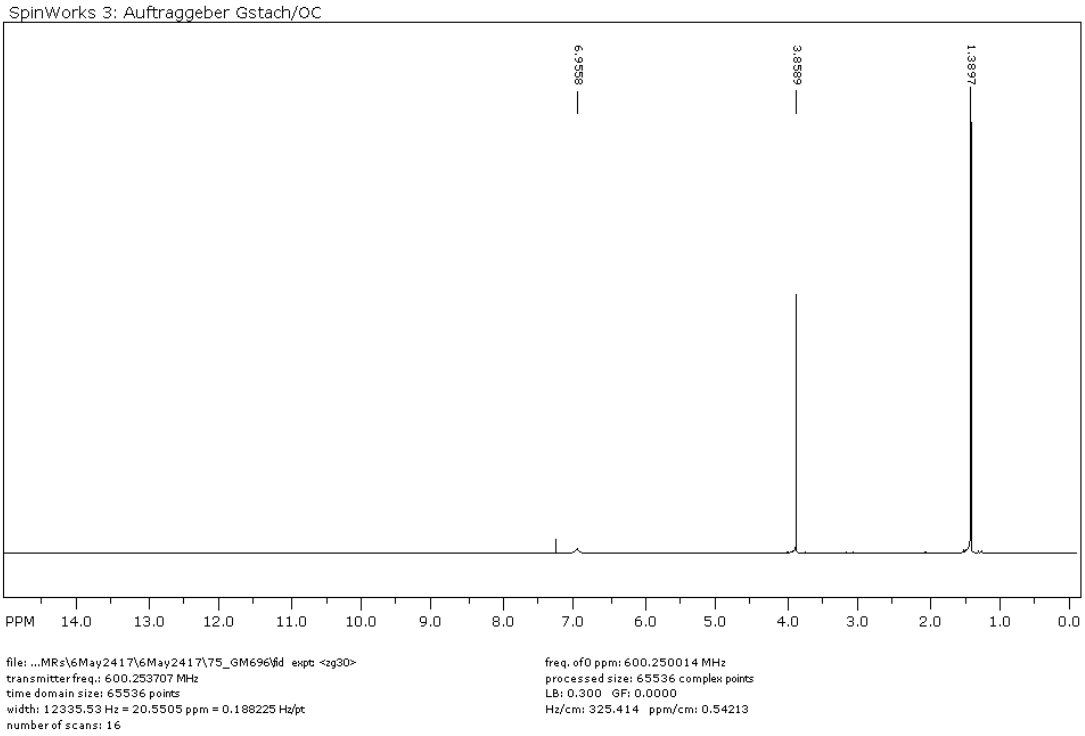

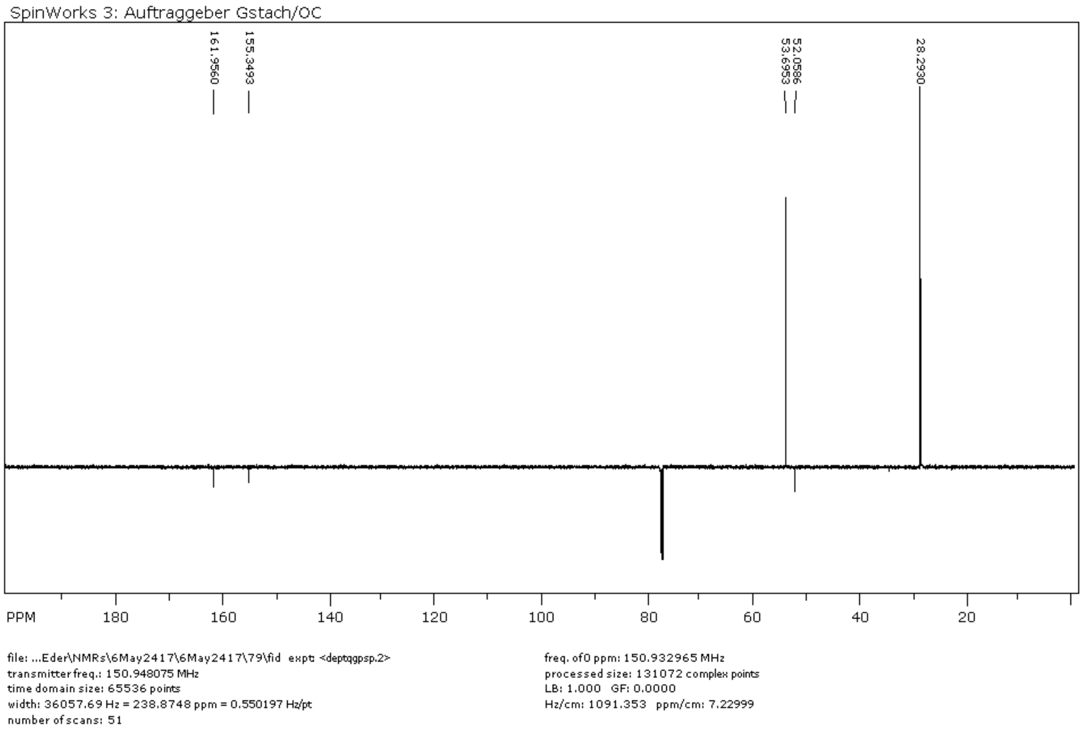

2-(benzylamino)-2-oxoacetic acid (S8)

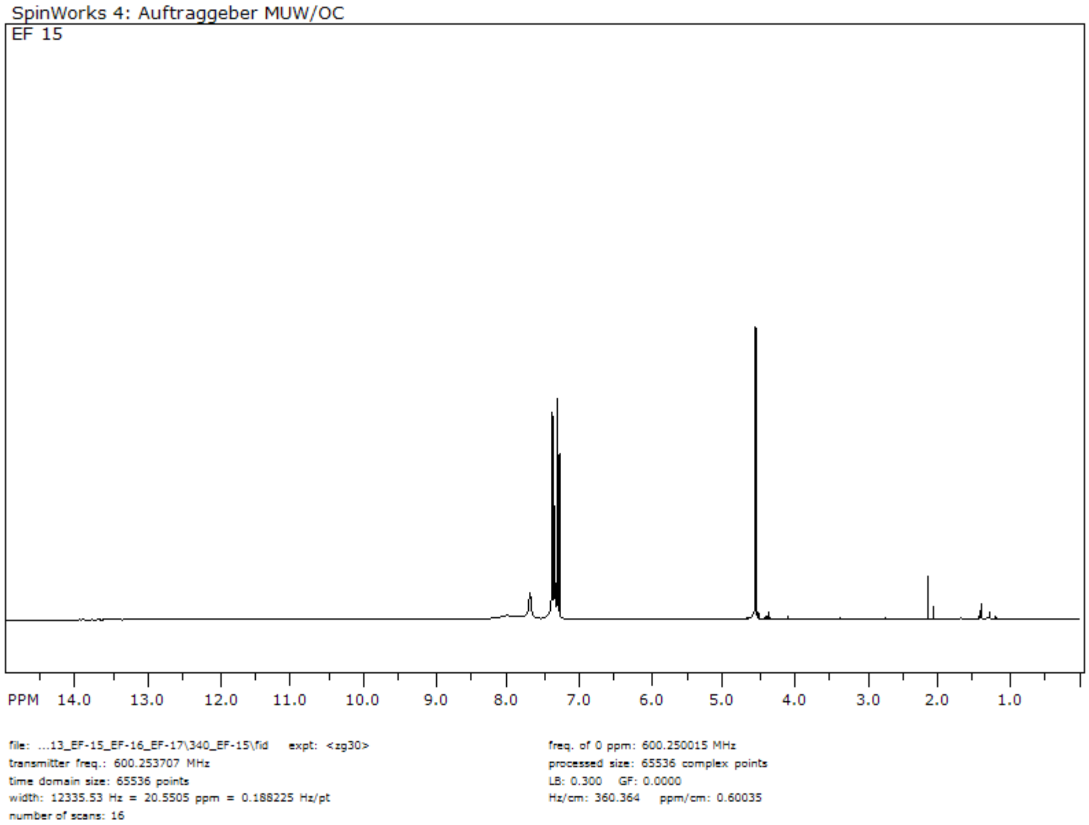

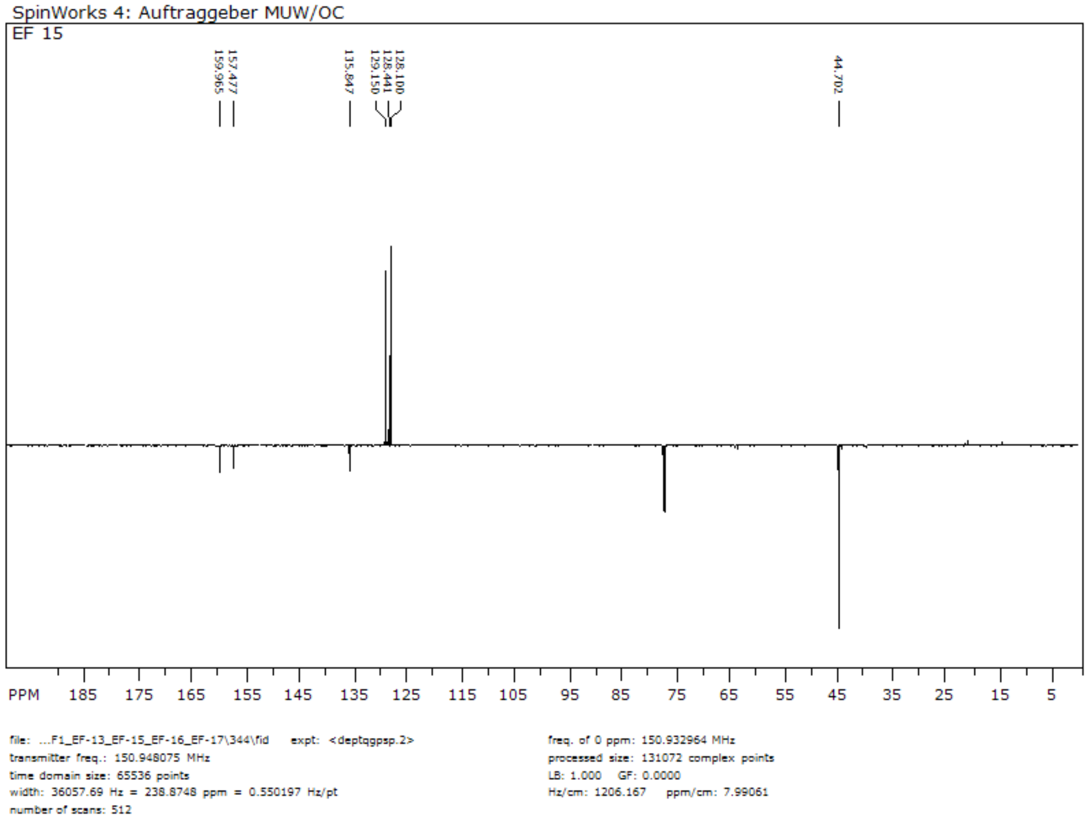

Methyl 2-(((1*S*,2*R*)-1-hydroxy-1-phenylpropan-2-yl)amino)-2-oxoacetate (S9)

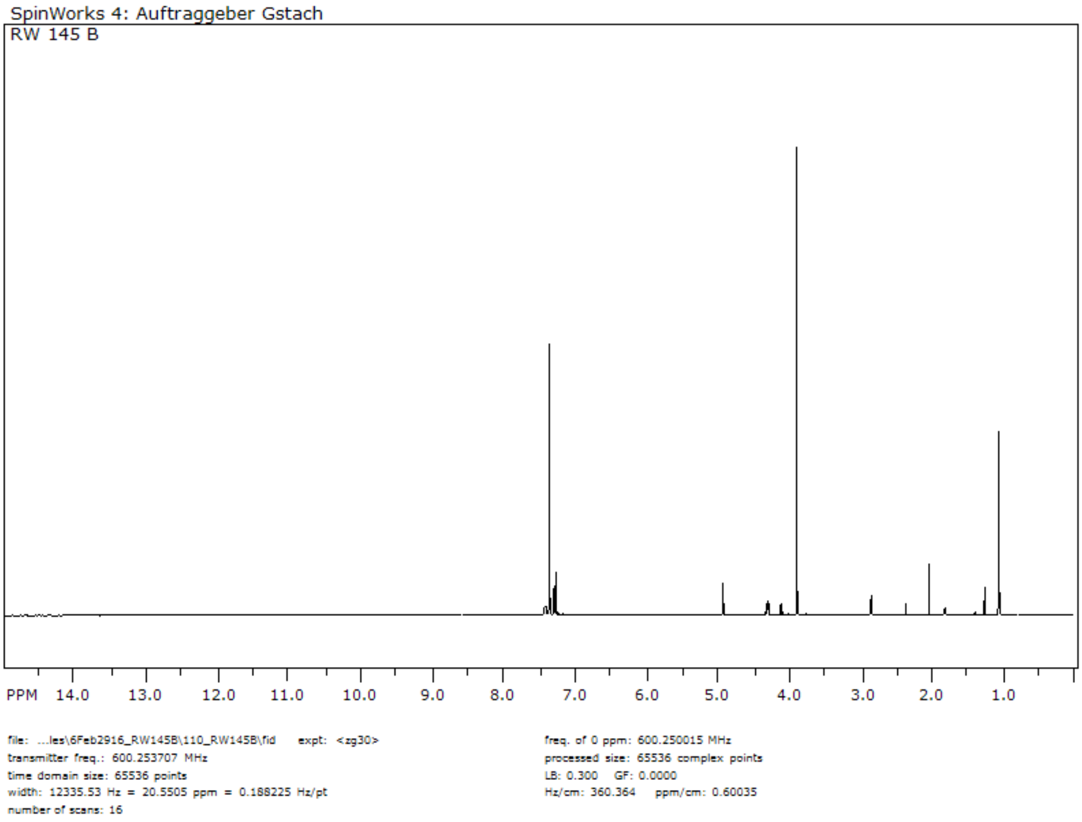

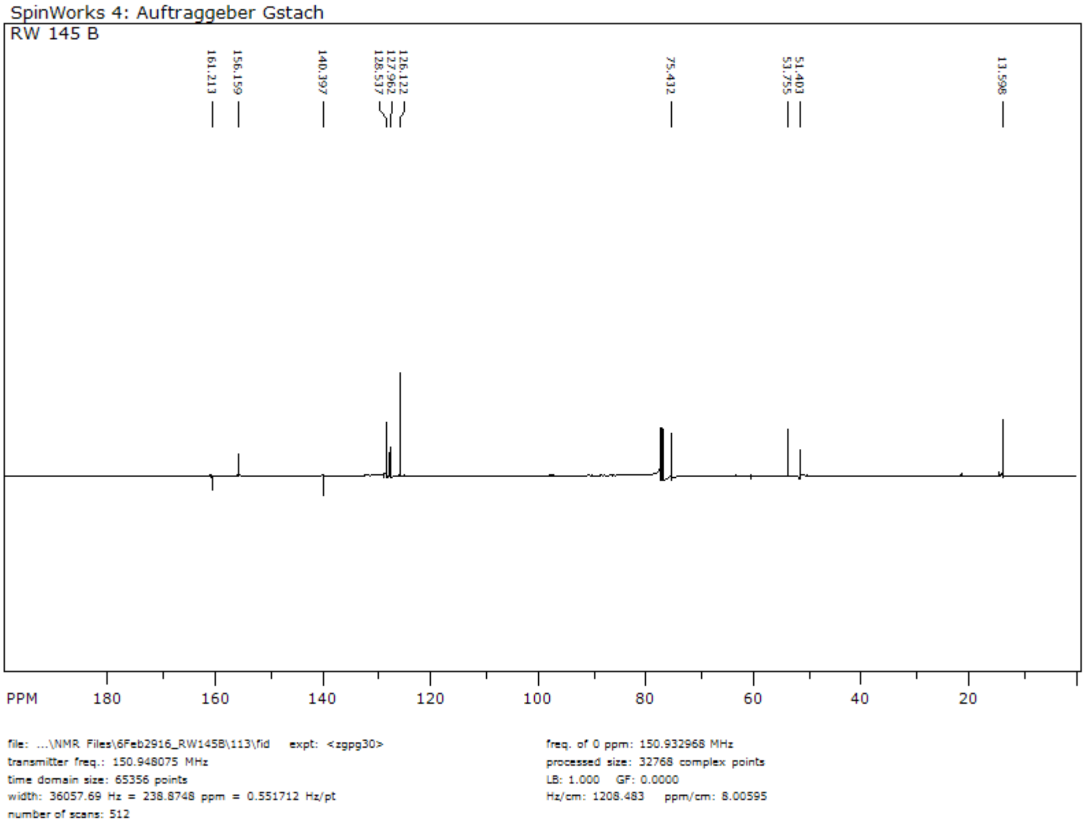

2-((4-fluorophenyl)amino)-2-oxoacetic acid (S10)

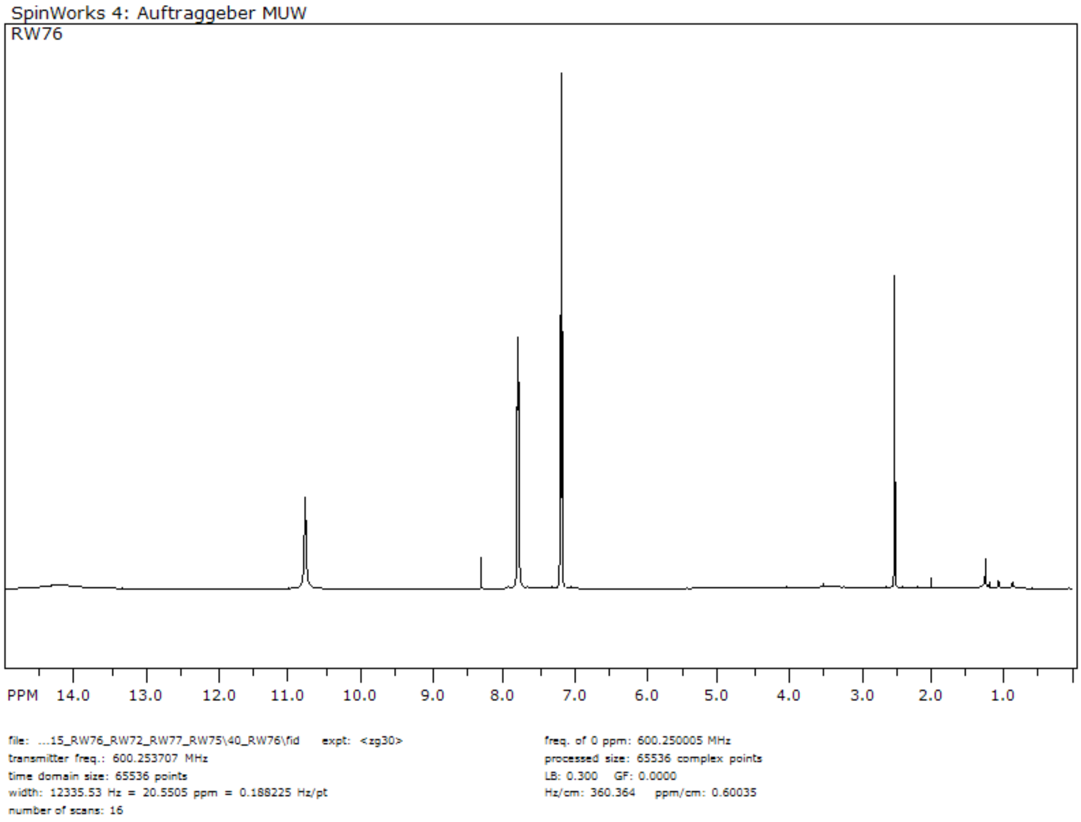

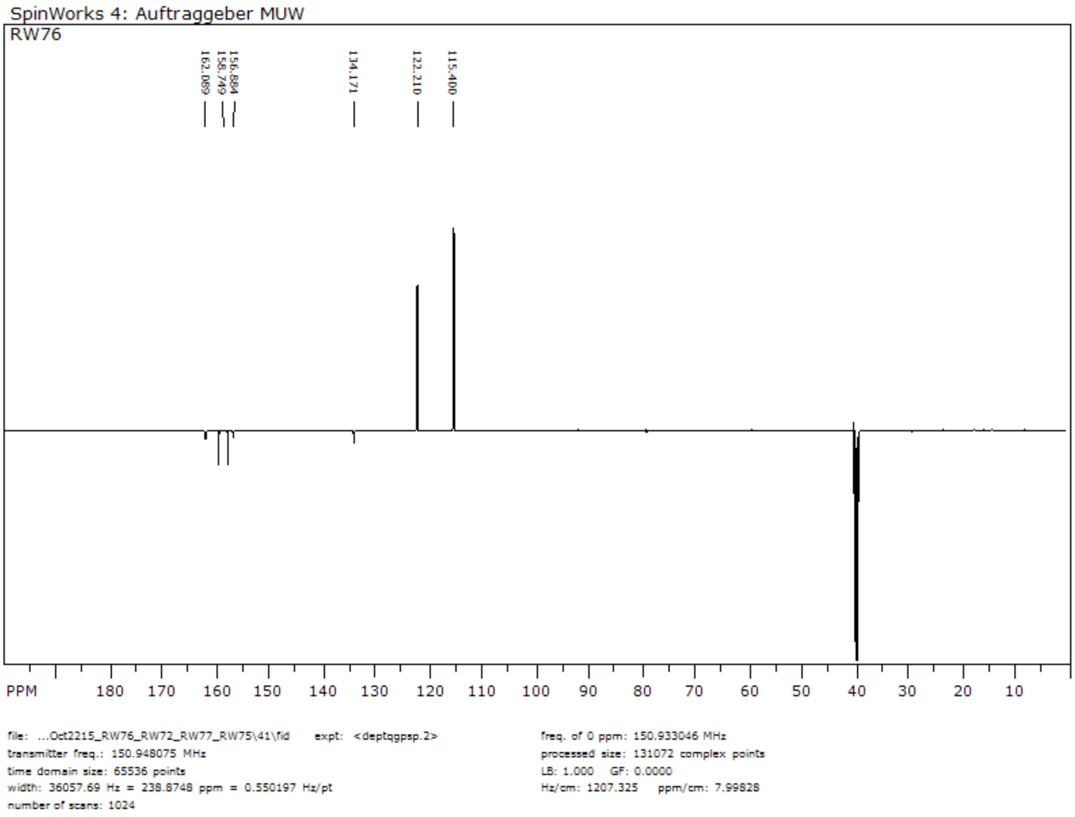

2-((2-fluorophenyl)amino)-2-oxoacetic acid (S11)

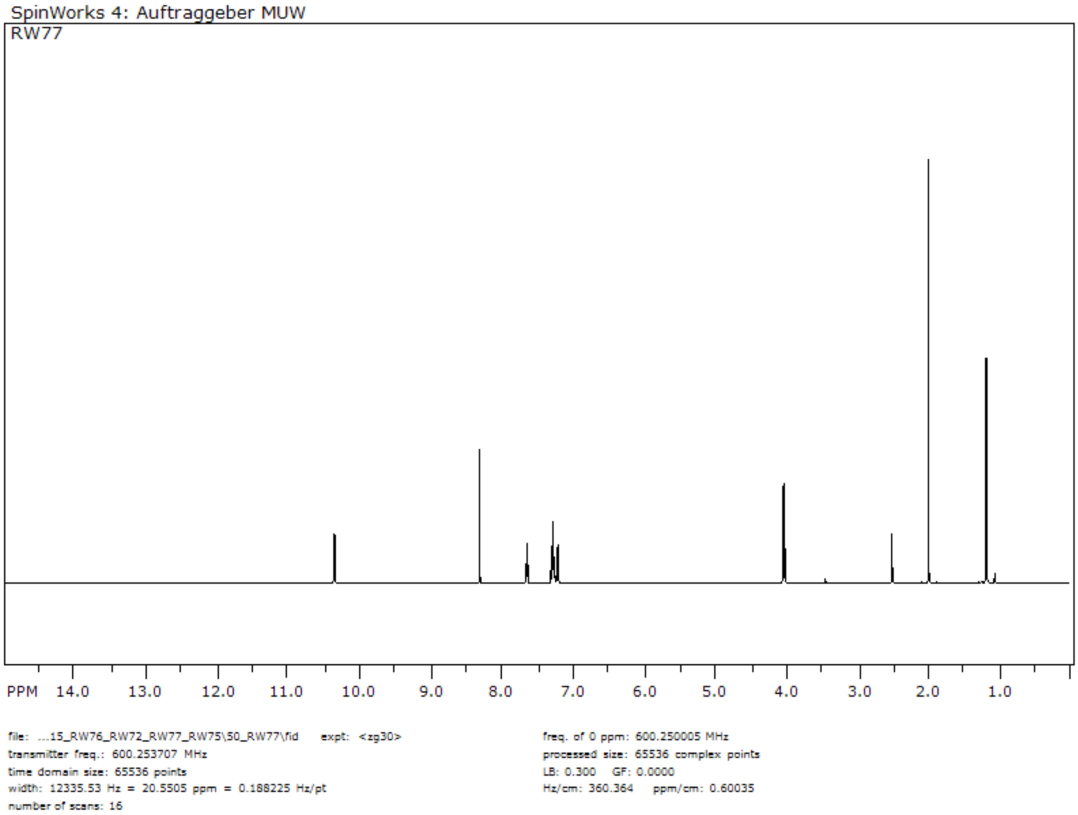

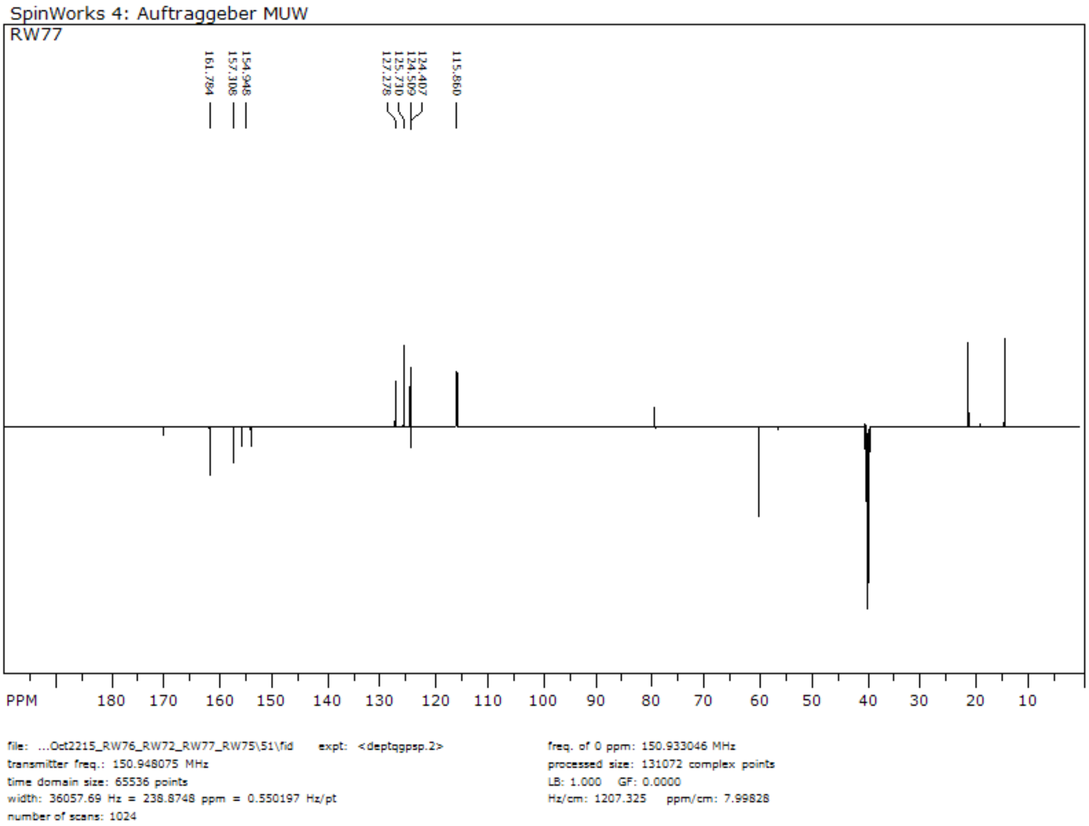

2-((2-bromophenyl)amino)-2-oxoacetic acid (S12)

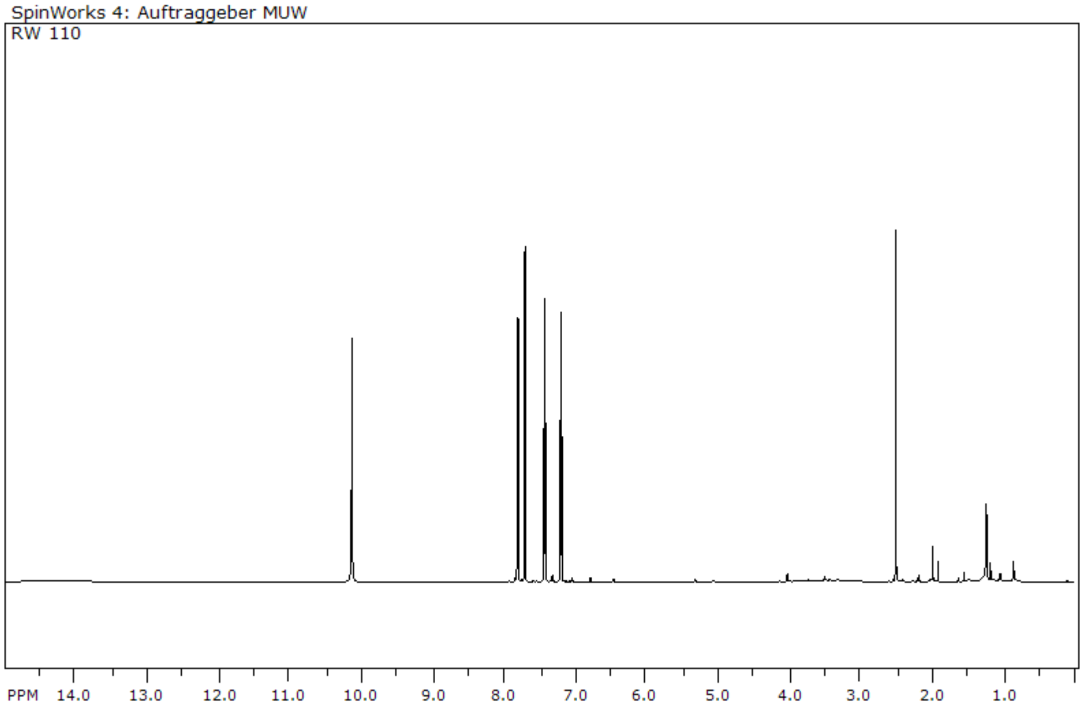

file: ...Files\7Dec0215 RW110_RW_111\40\fid expt: <zg30>
transmitter freq.: 700.404325 MHz
time domain size: 65536 points
width: 14097.74 Hz = 20.1280 ppm = 0.215115 Hz/pt
number of scans: 16

freq. of 0 ppm: 700.400005 MHz
processed size: 65536 complex points
LB: 0.300 GF: 0.0000
Hz/cm: 420.443 ppm/cm: 0.60029

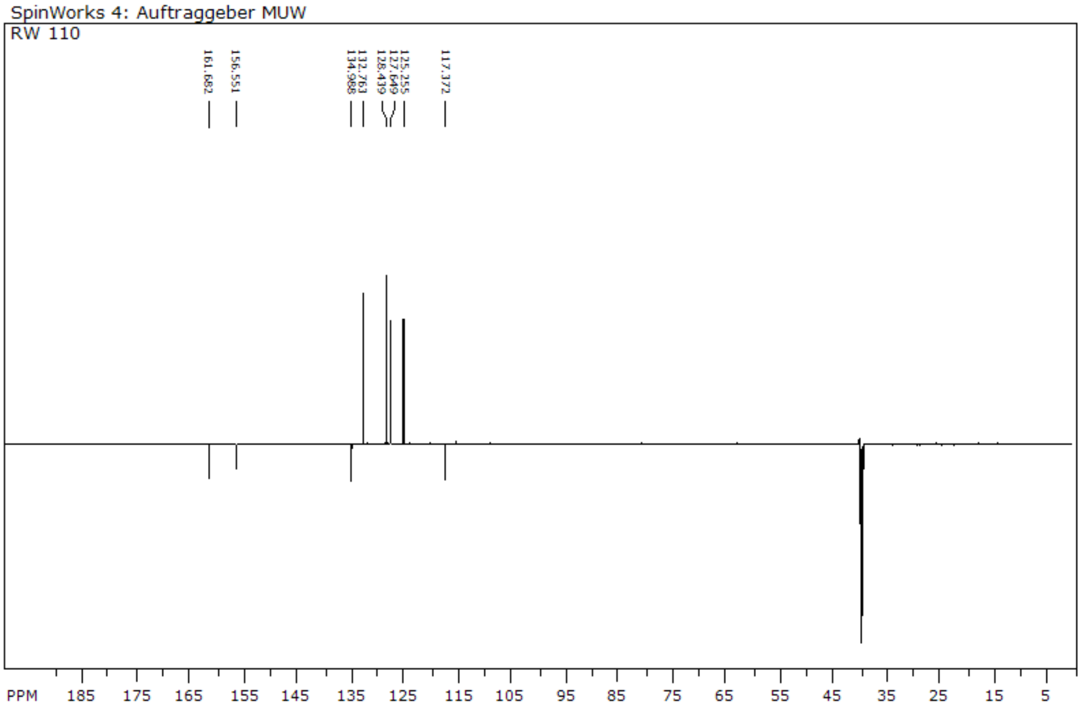

file: ...Files\7Dec0215 RW110_RW_111\44\fid expt: <deptqgpsp.2>
transmitter freq.: 176.133332 MHz
time domain size: 65536 points
width: 41666.67 Hz = 236.5632 ppm = 0.635783 Hz/pt
number of scans: 1024

freq. of 0 ppm: 176.115798 MHz
processed size: 131072 complex points
LB: 1.000 GF: 0.0000
Hz/cm: 1408.507 ppm/cm: 7.99682

2-((3-bromophenyl)amino)-2-oxoacetic acid (S13)

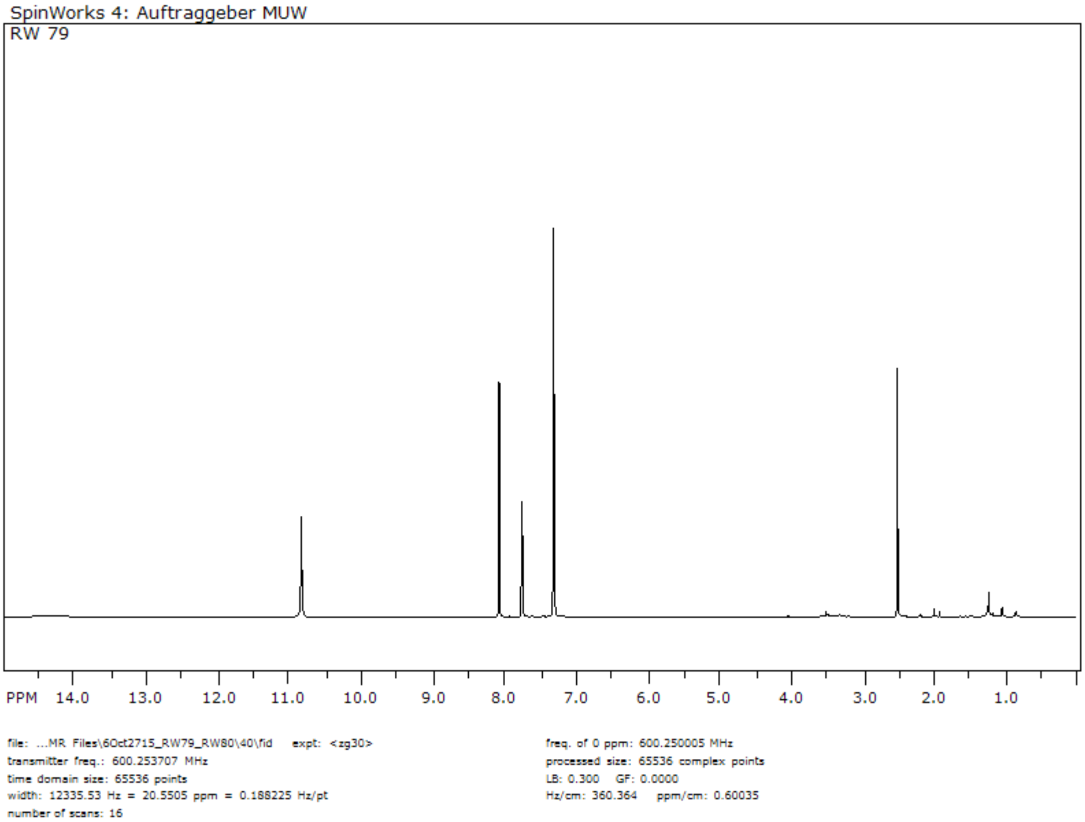

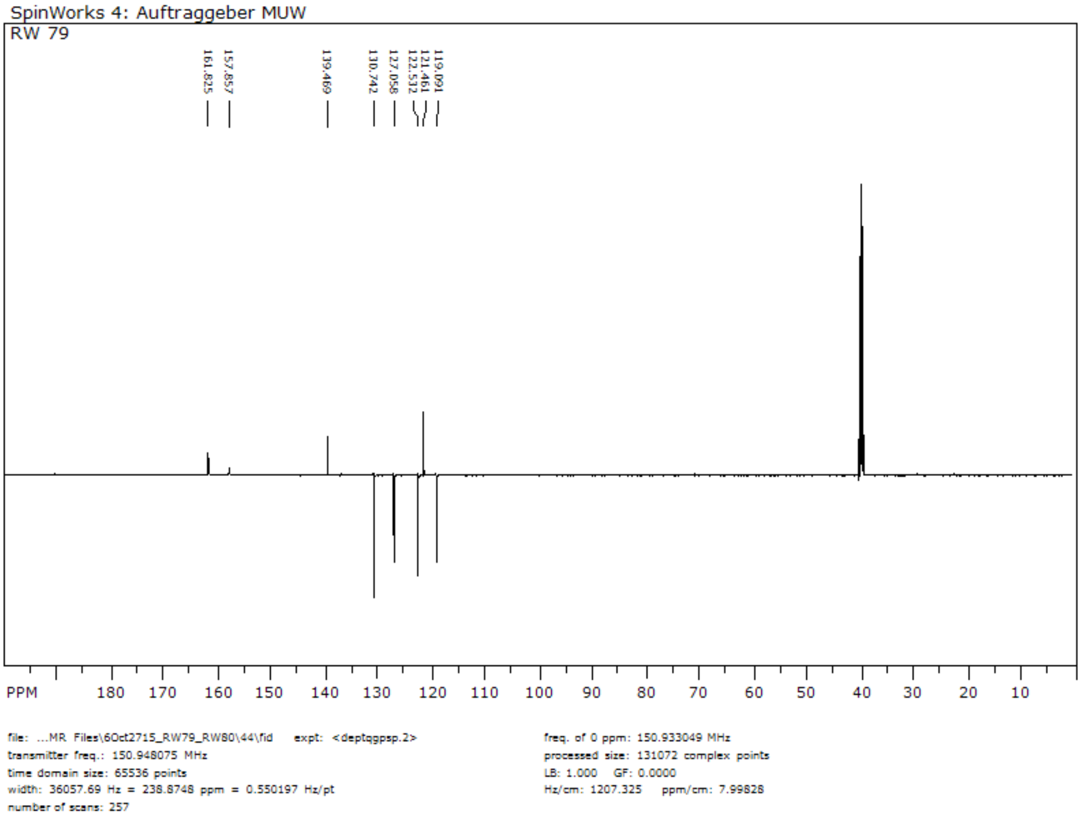

2-((2,4-dichlorophenyl)amino)-2-oxoacetic acid (S14)

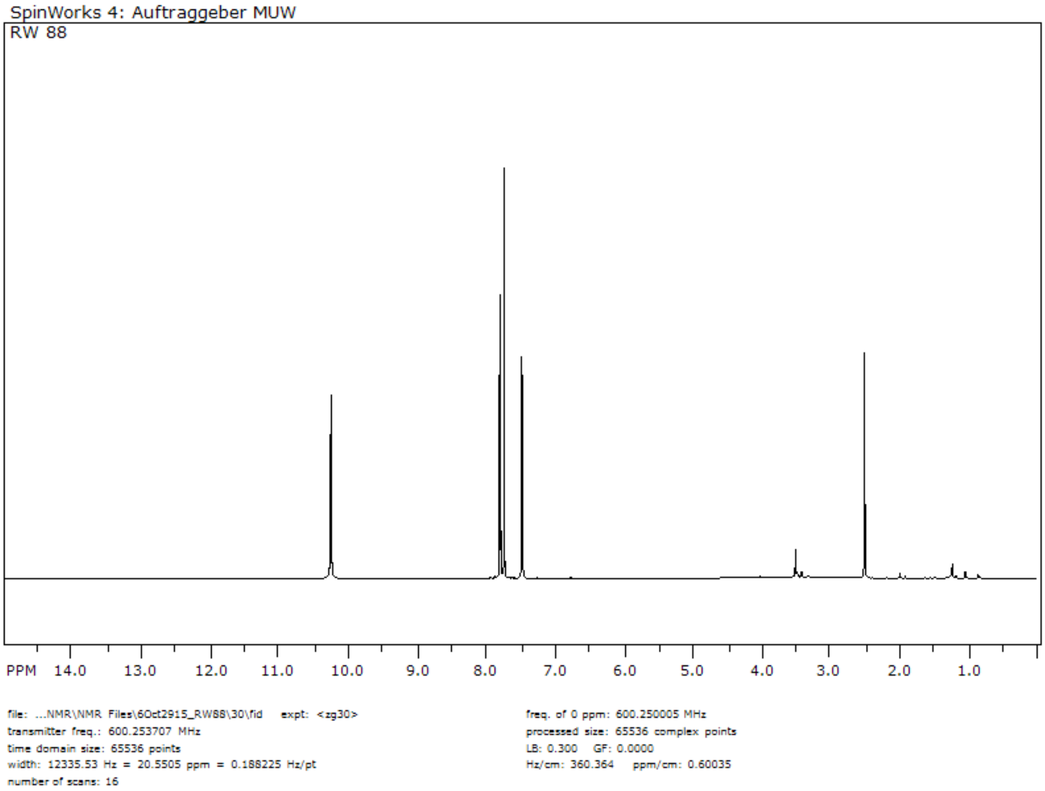

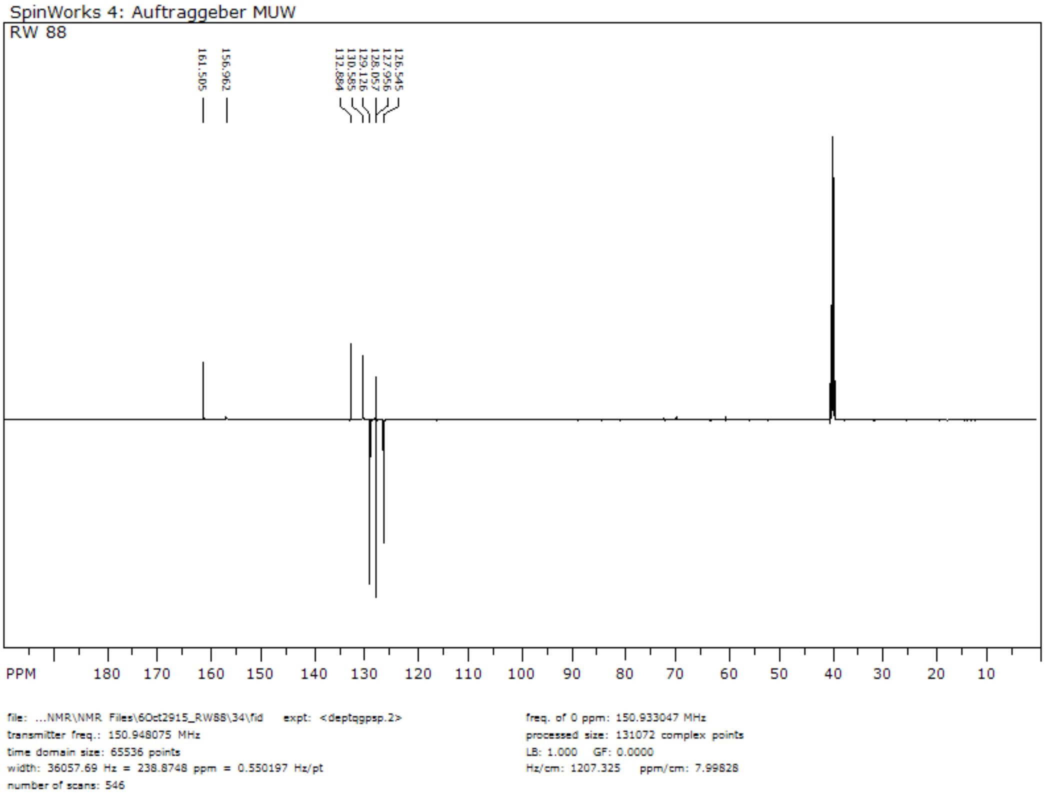

2-((3-hydroxyphenyl)amino)-2-oxoacetic acid (S15)

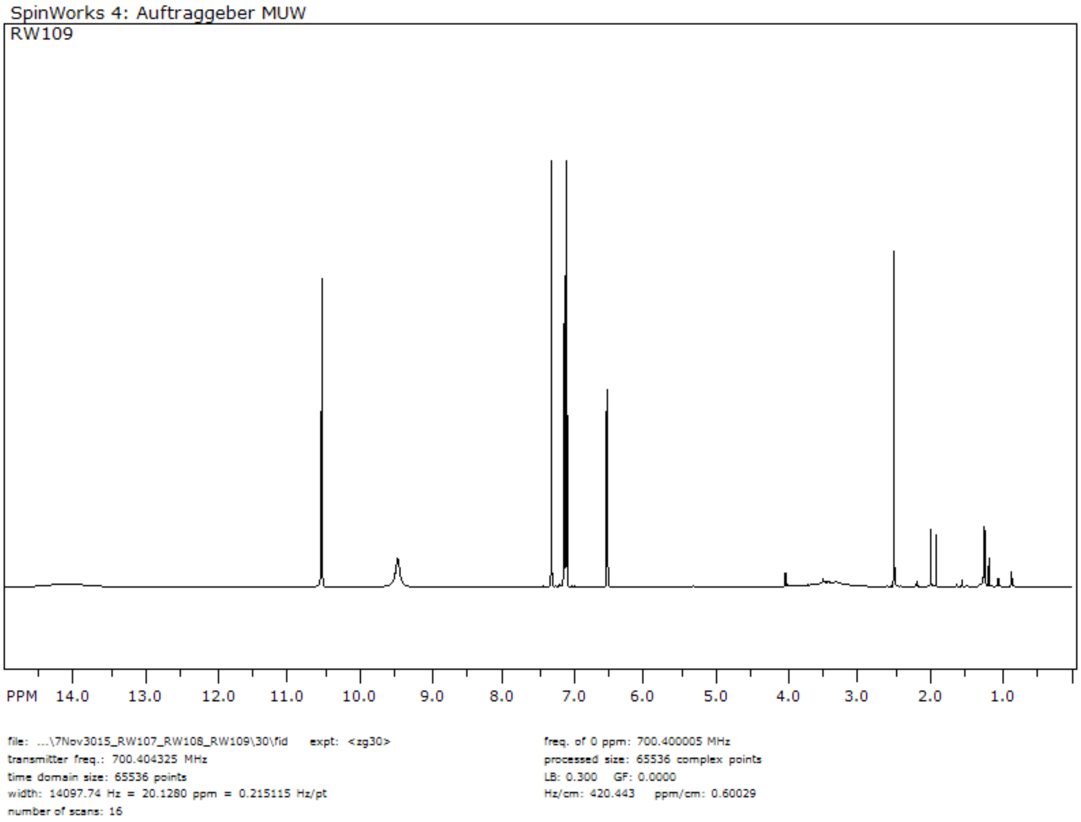

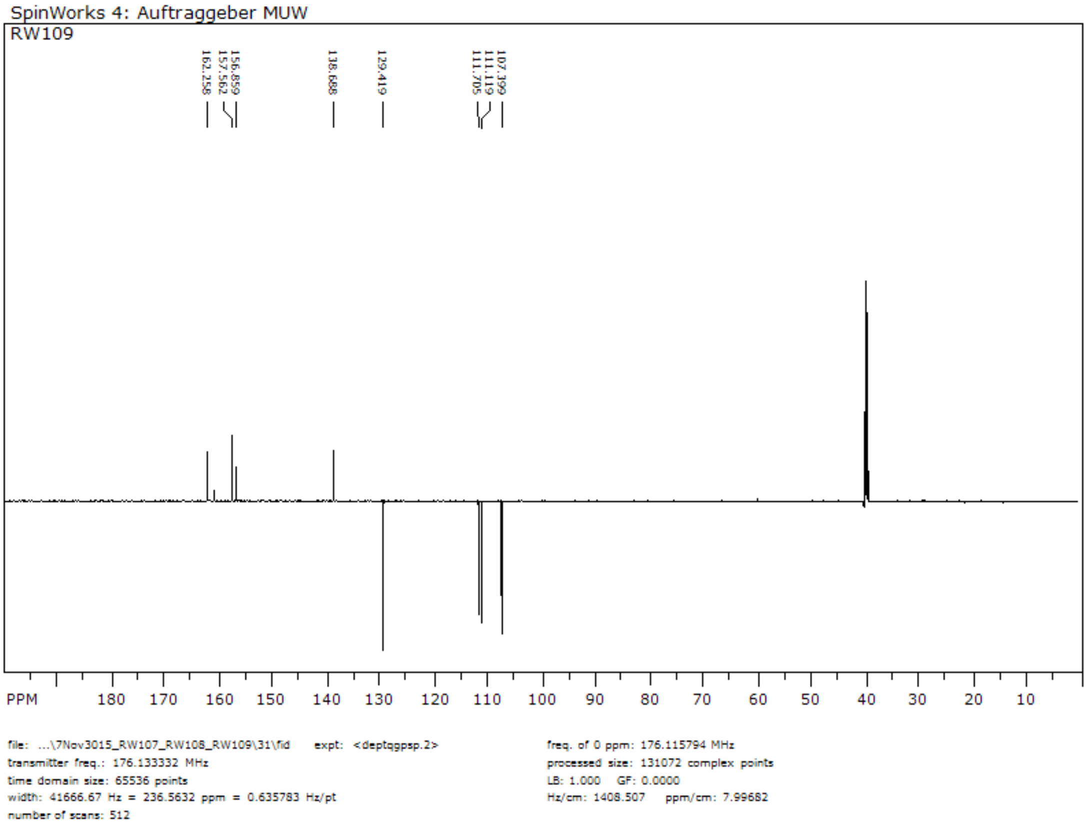

2-((2-hydroxyphenyl)amino)-2-oxoacetic acid (S16)

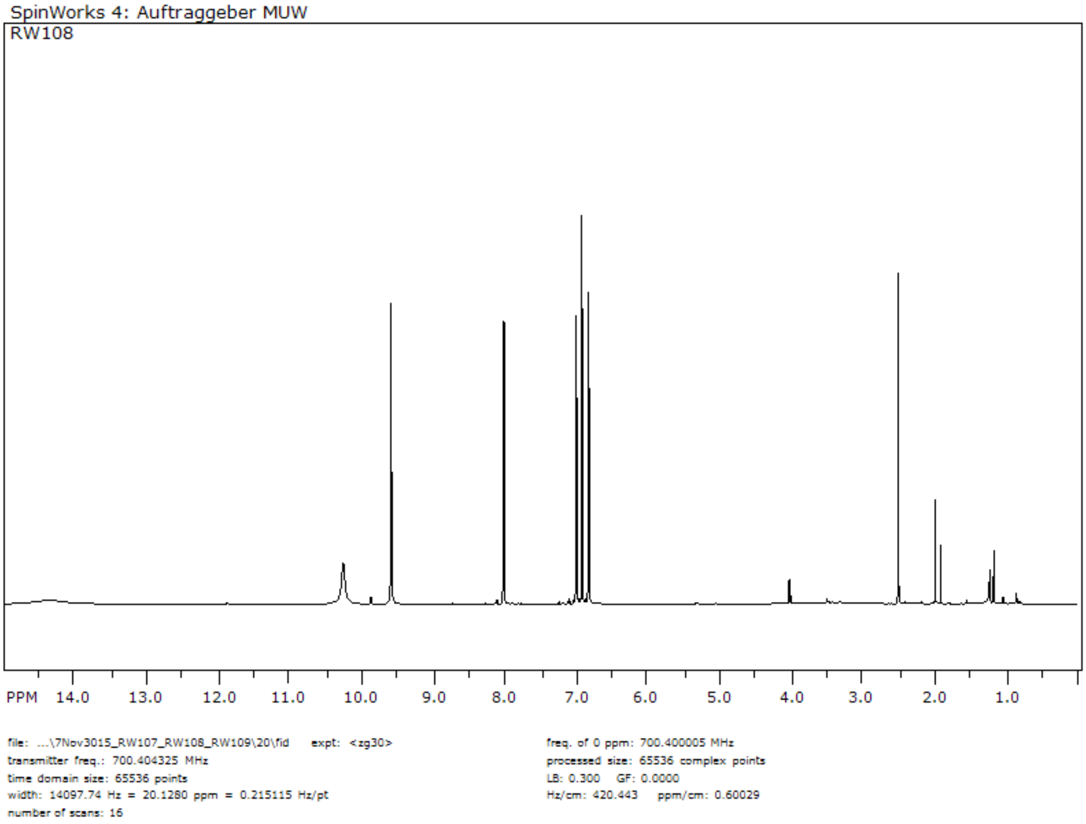

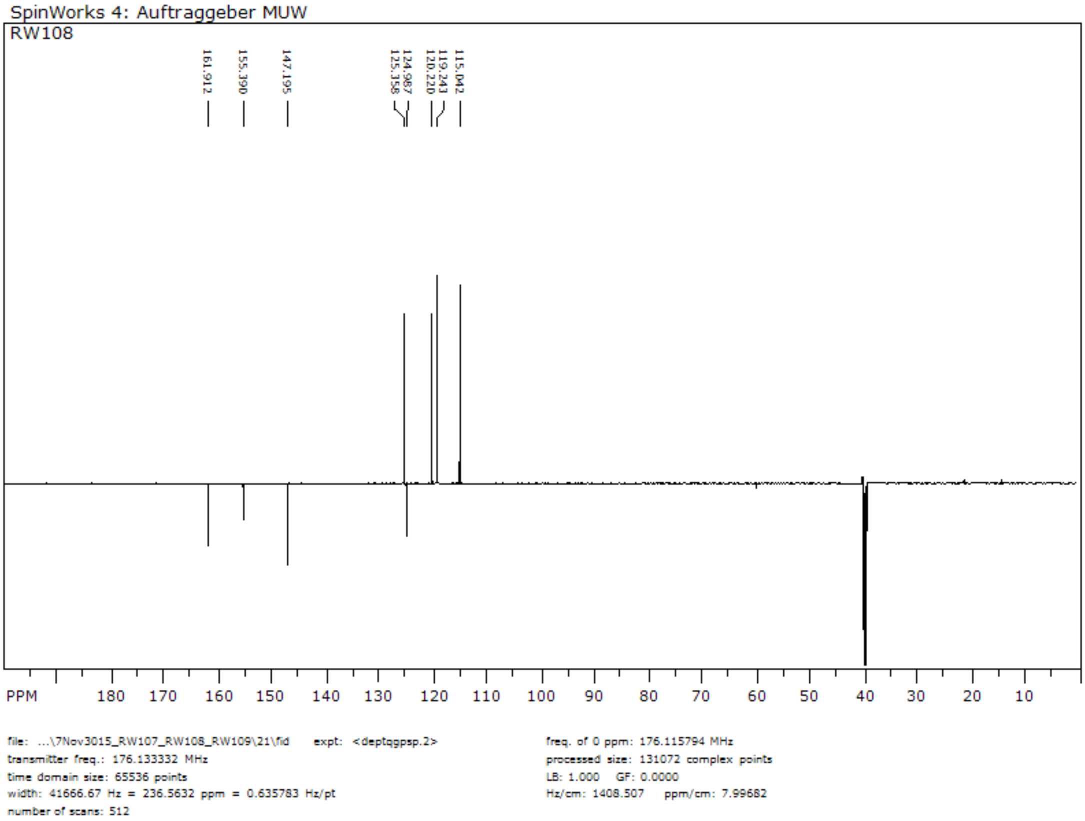

2-((2-methoxyphenyl)amino)-2-oxoacetic acid (S17)

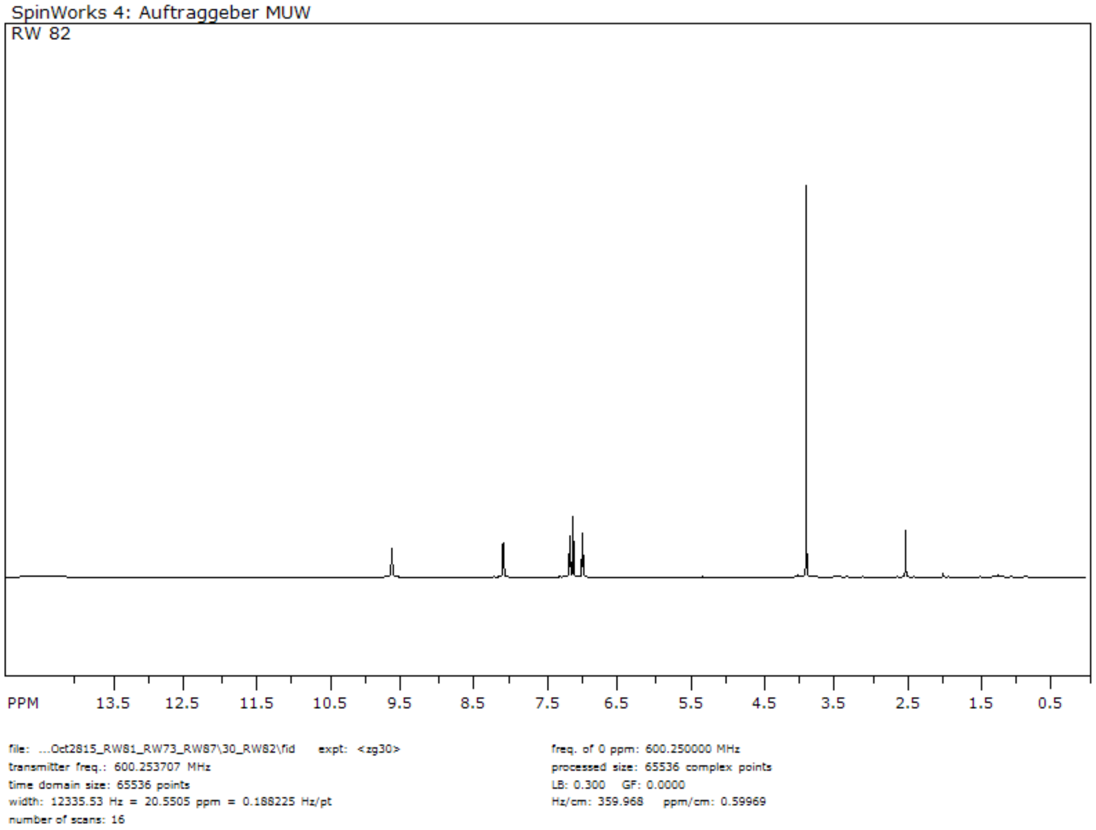

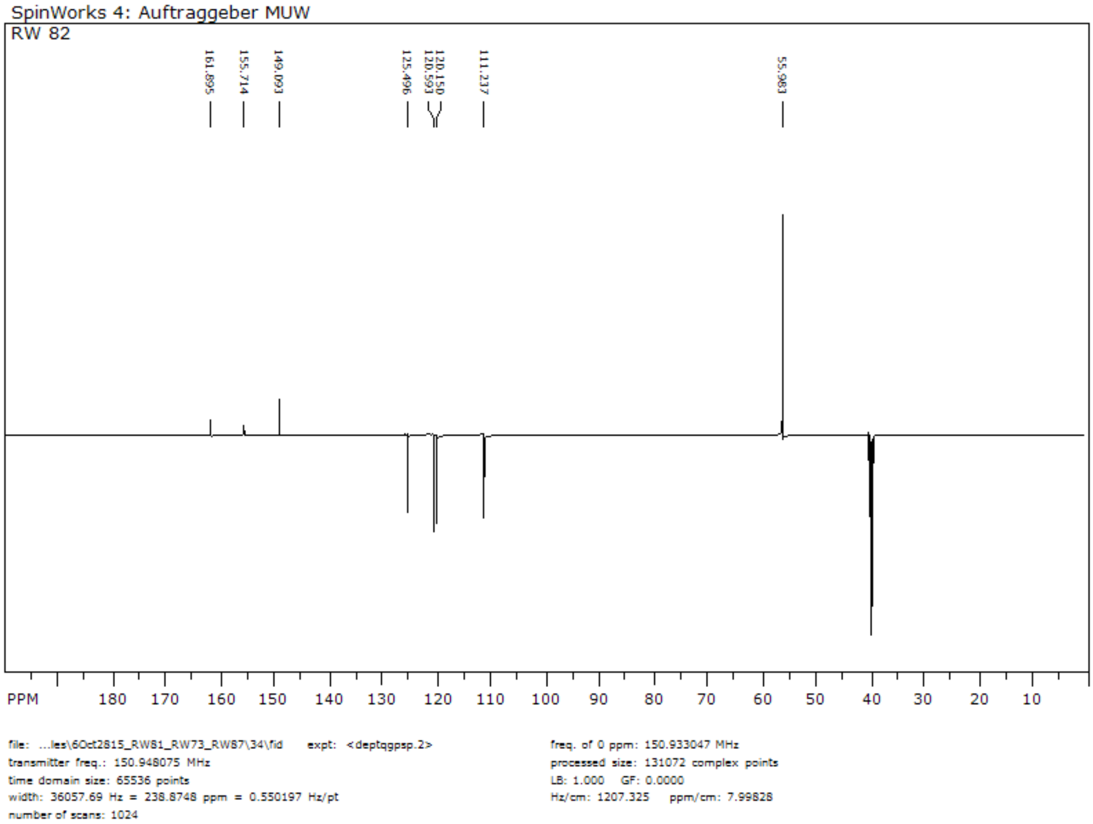

2-((4-methoxyphenyl)amino)-2-oxoacetic acid (S18)

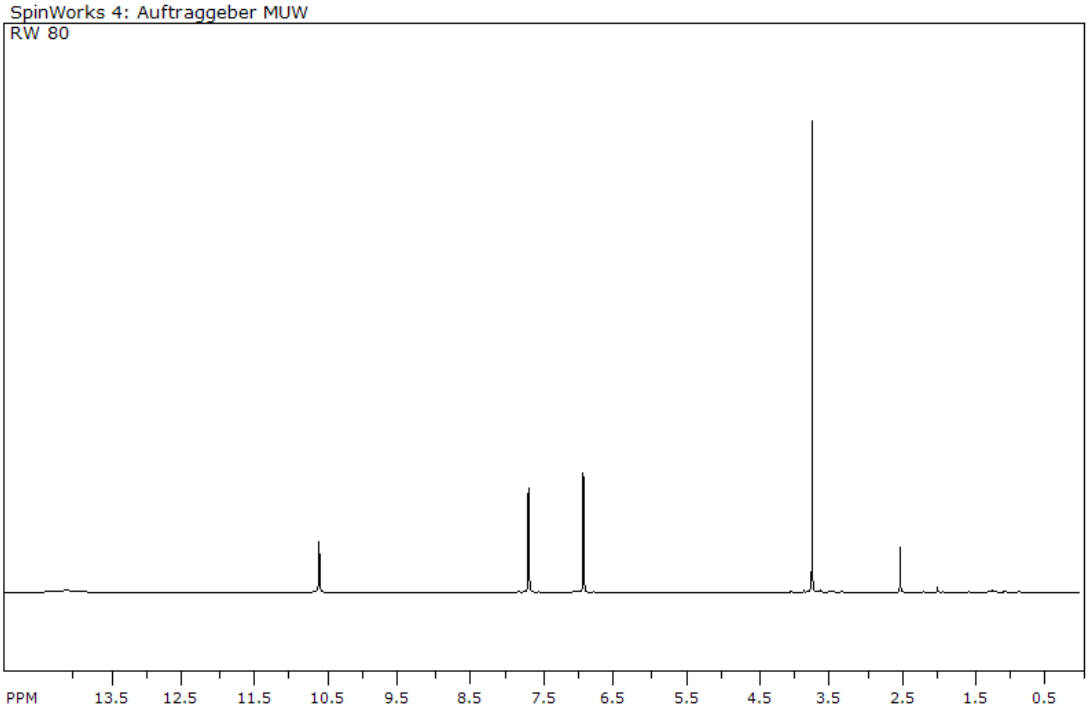

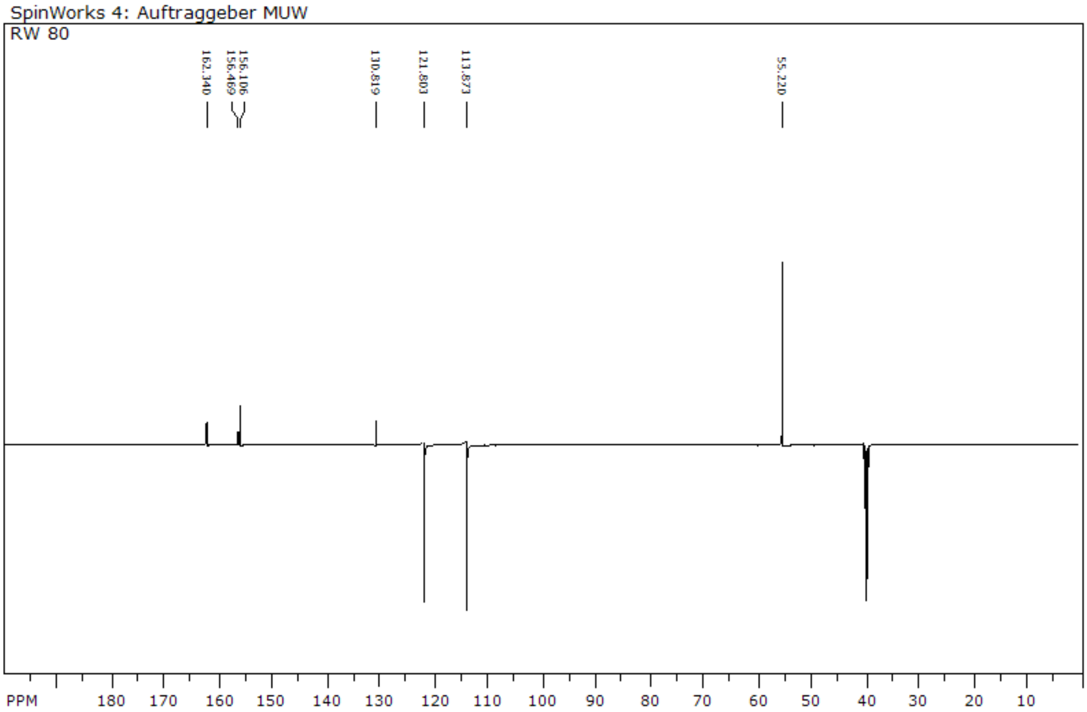

2-(2-(*tert*-butoxy)-2-oxoacetamido)-5-methylpyridine 1-oxide (S19)

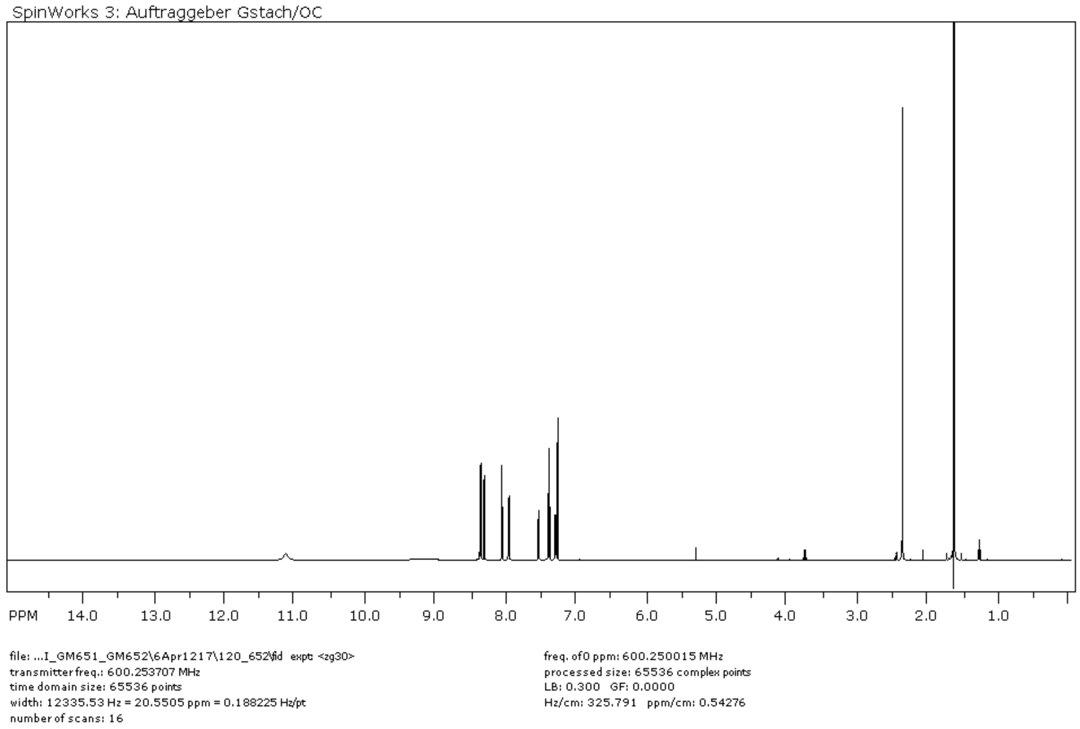

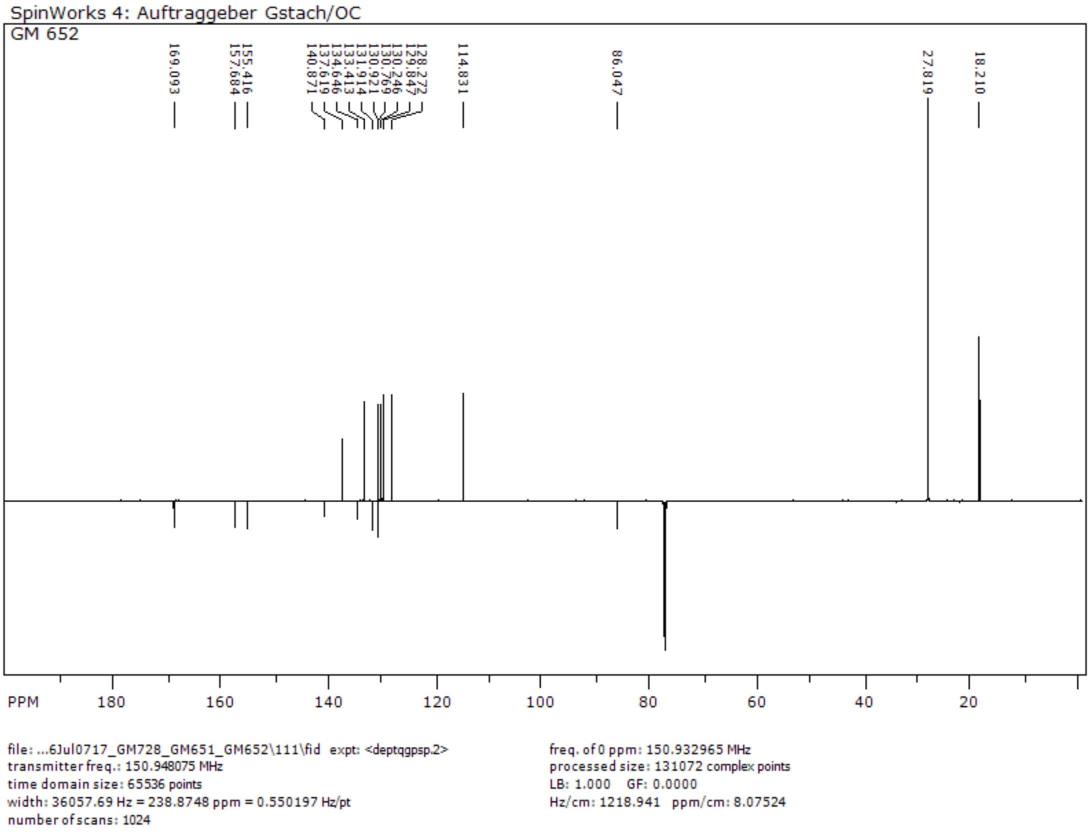

***tert*-butyl 2-((5-methylpyridin-2-yl)amino)-2-oxoacetate (S20)**

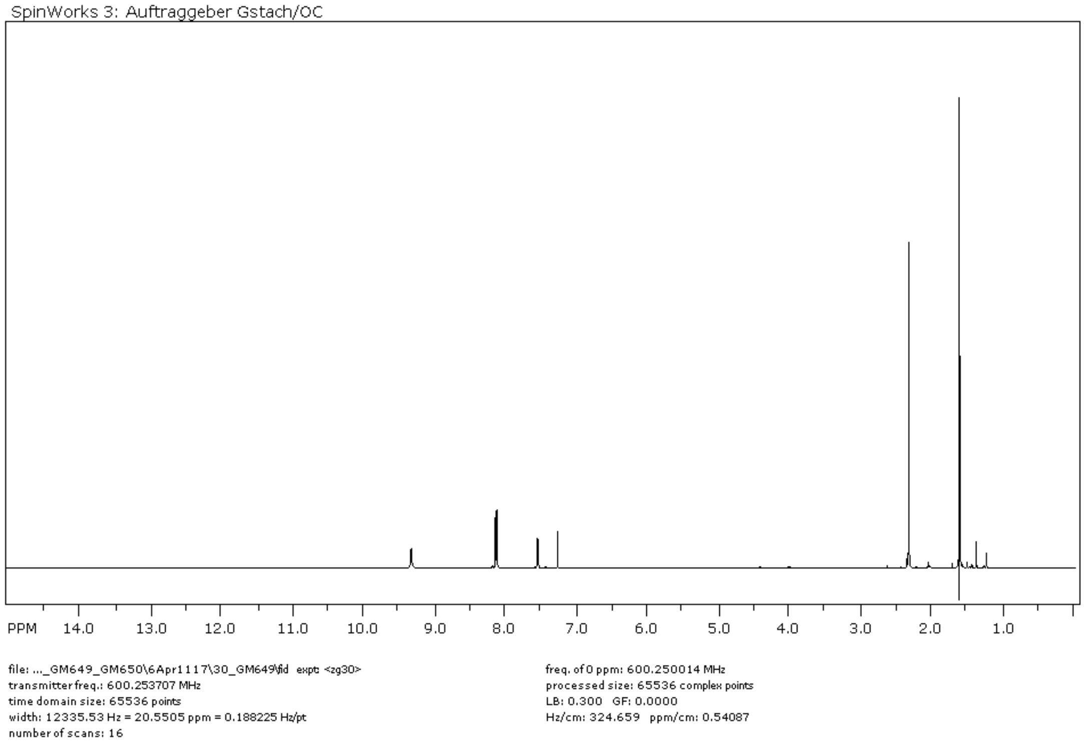

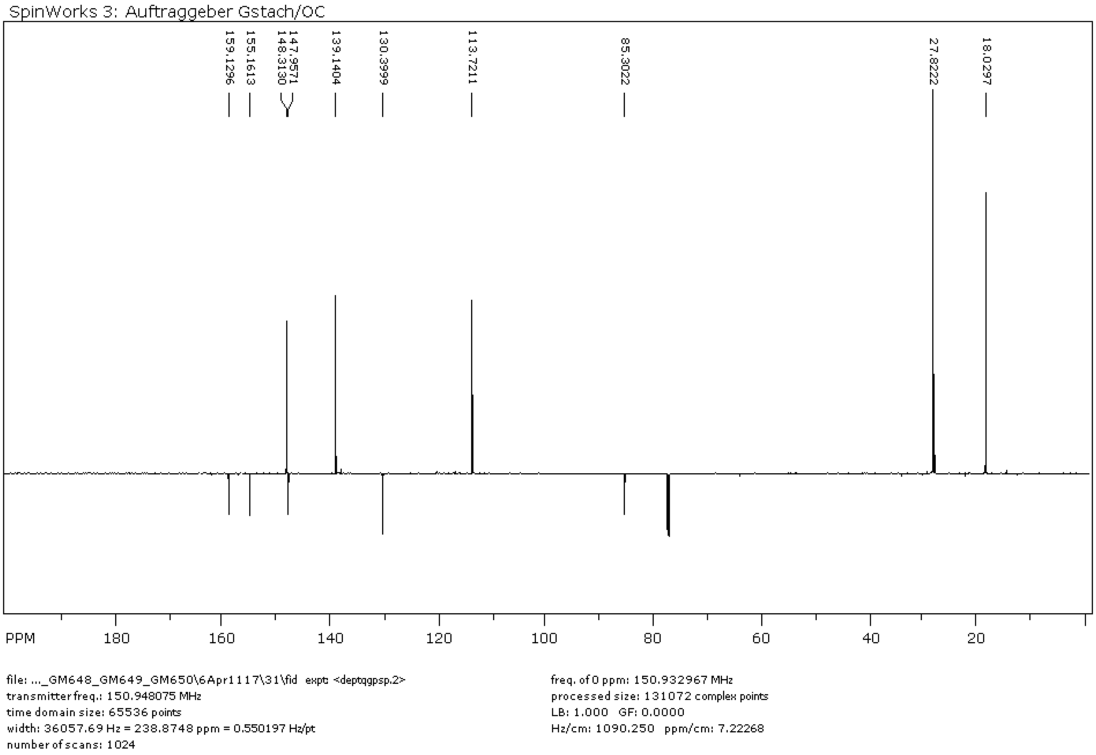

5-bromo-2-(2-(*tert*-butoxy)-2-oxoacetamido)pyridine 1-oxide (S21)

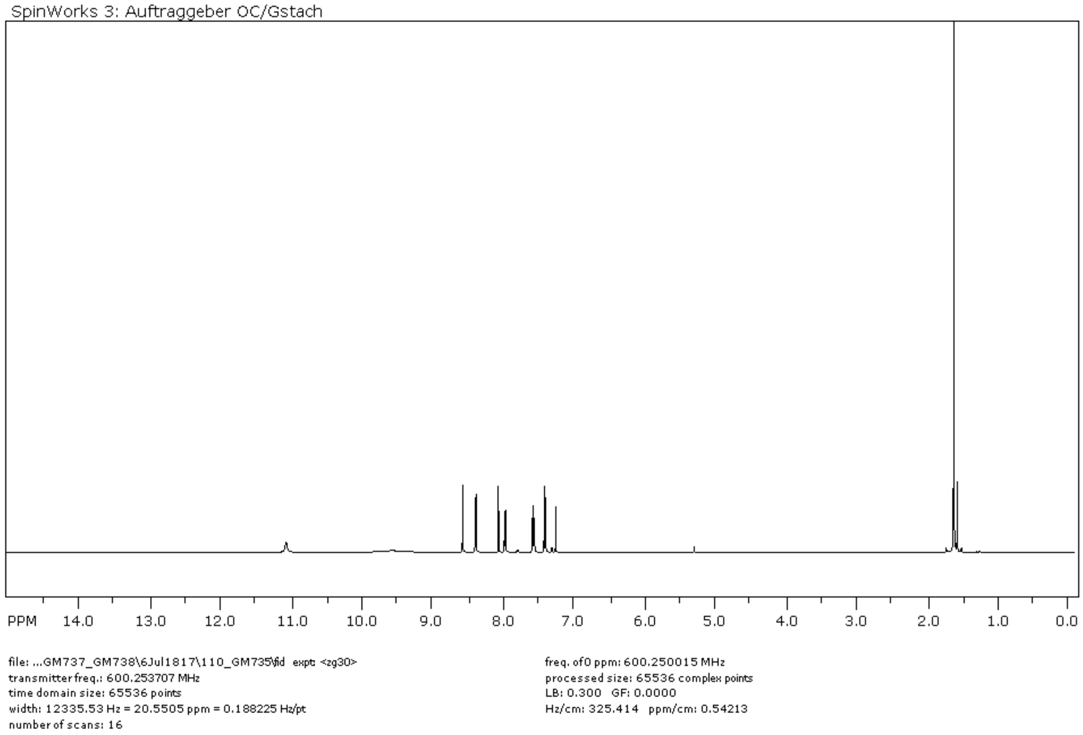

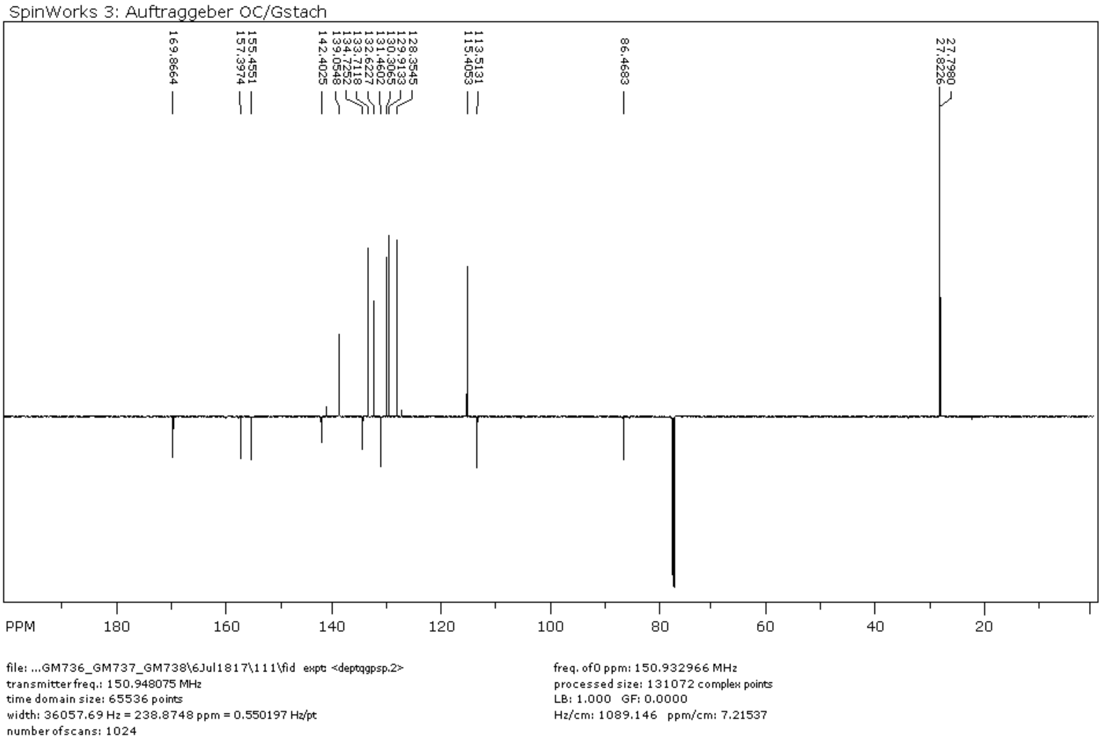

2-(2-(*tert*-butoxy)-2-oxoacetamido)-6-(methoxycarbonyl)pyridine 1-oxide (S22)

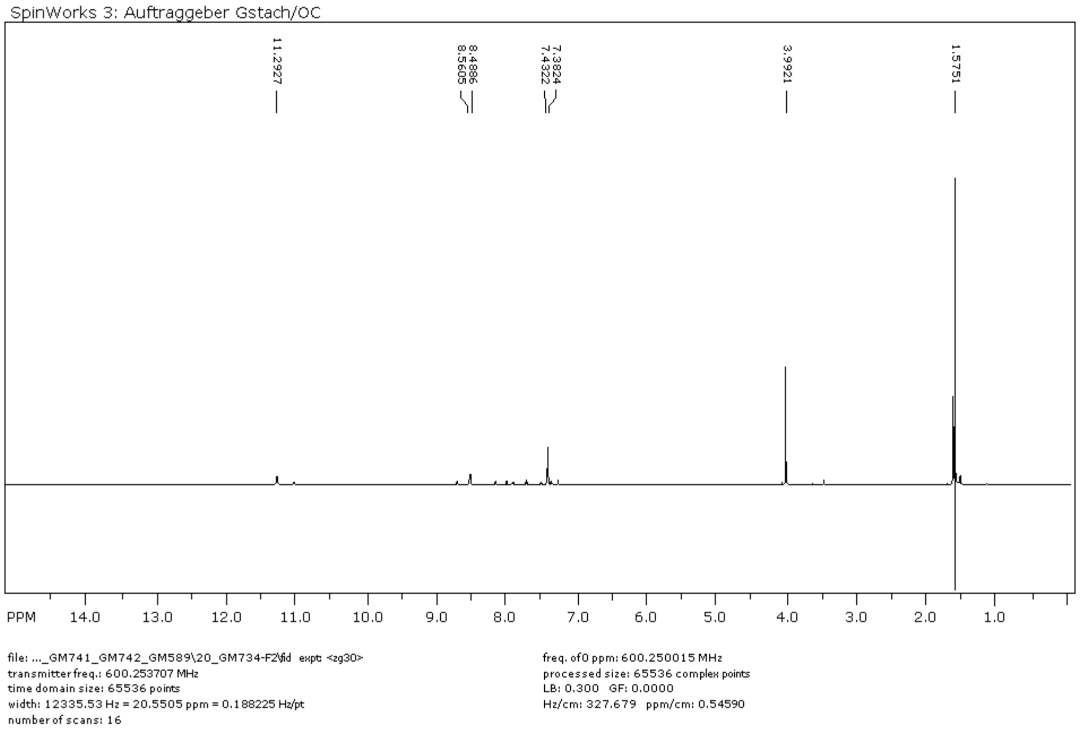

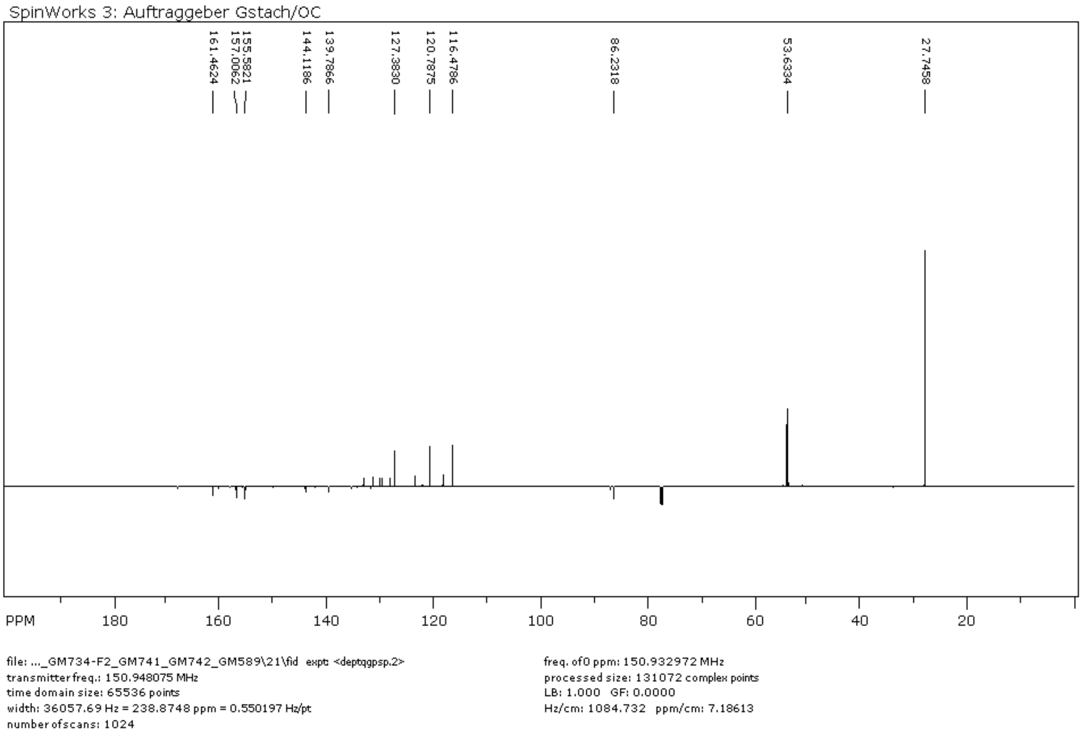

- FAHD1-inhibitor titration curves

1a

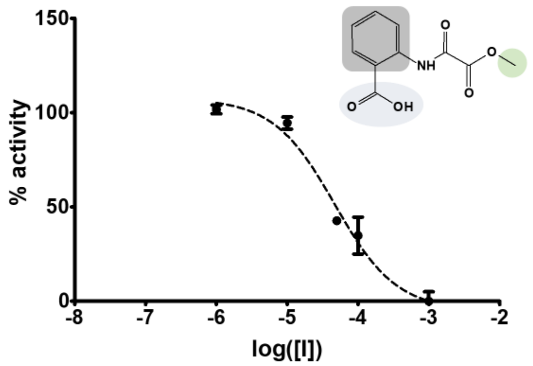

1b

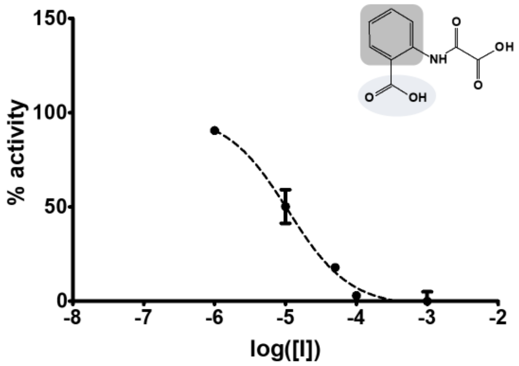

2a

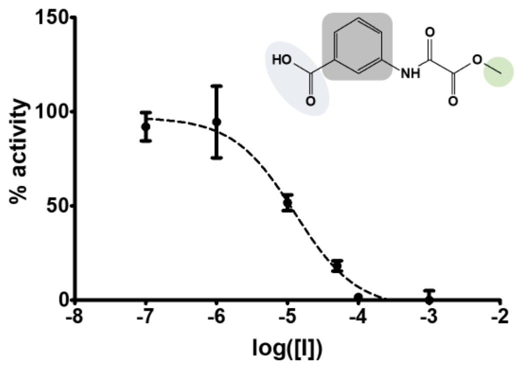

2b

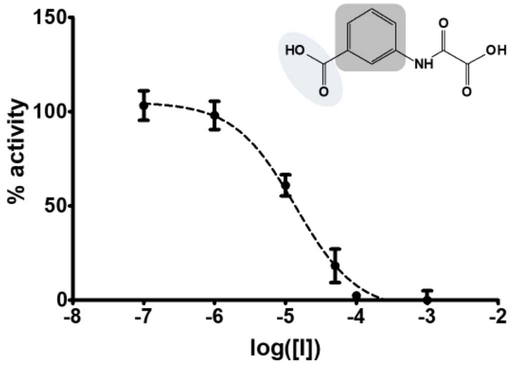

3a

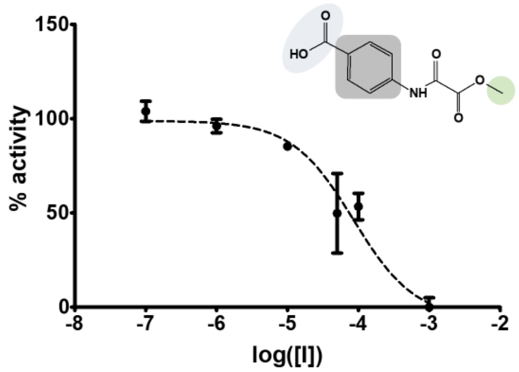

3b

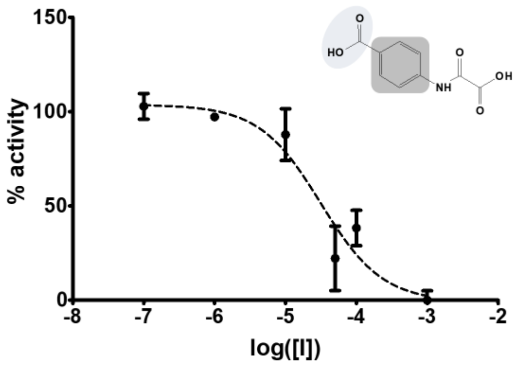

4b

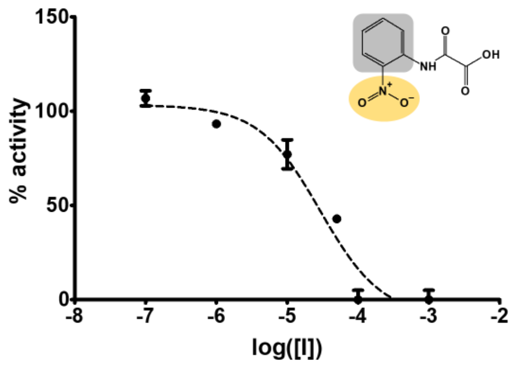

5b

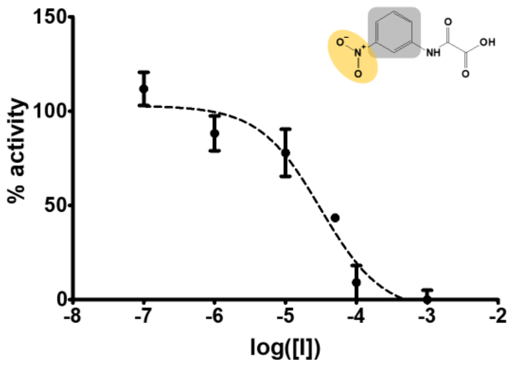

6b

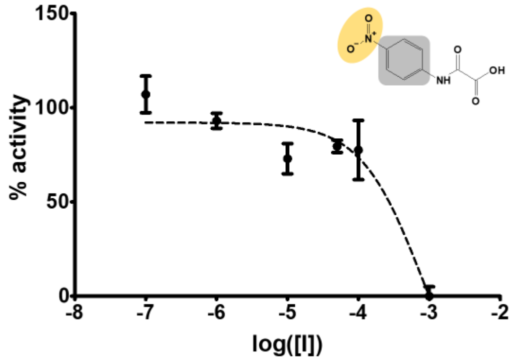

7a

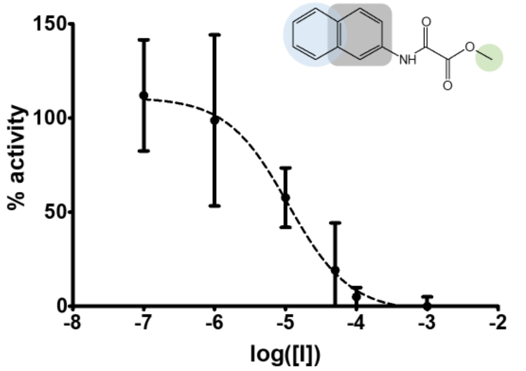

7b

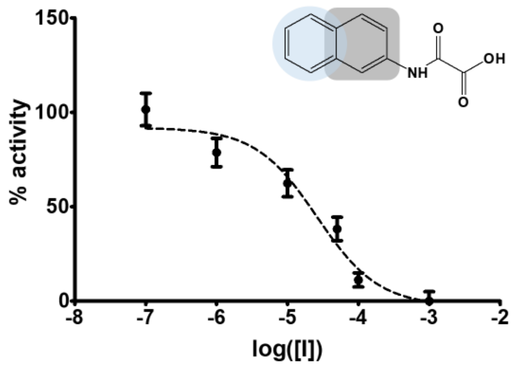

8a

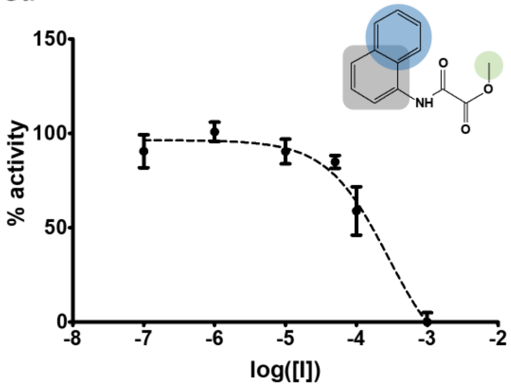

8b

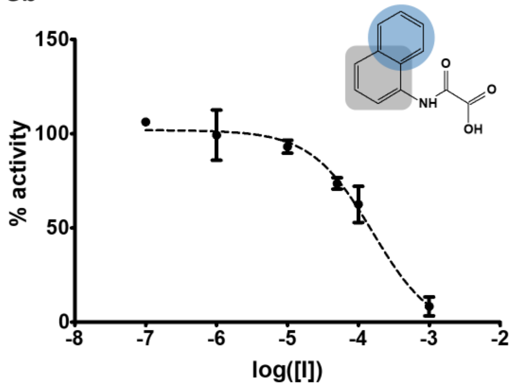

9a

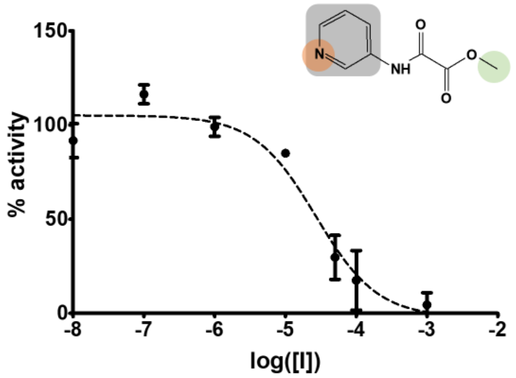

10a

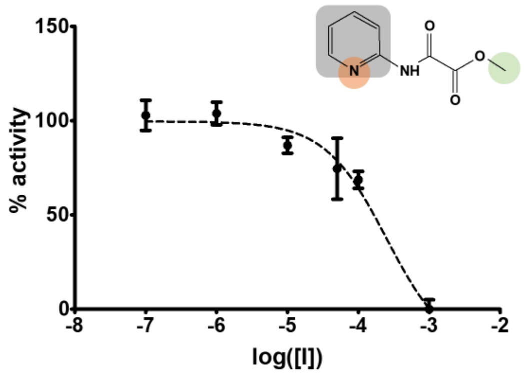

11a

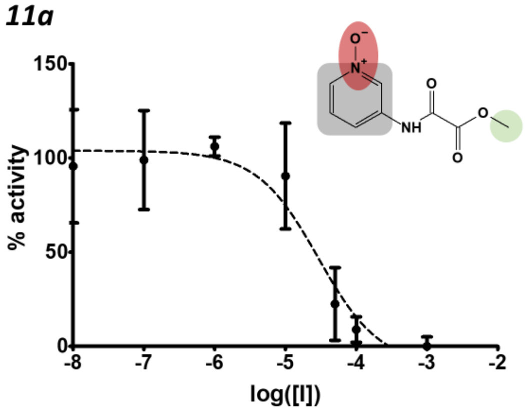

11b

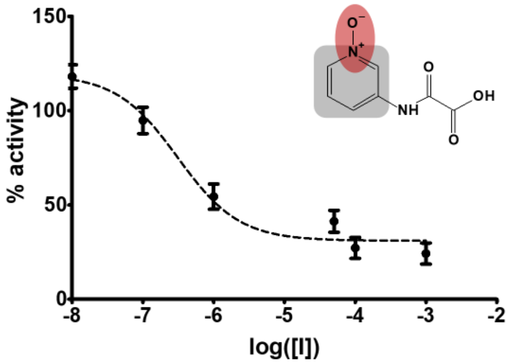

12a

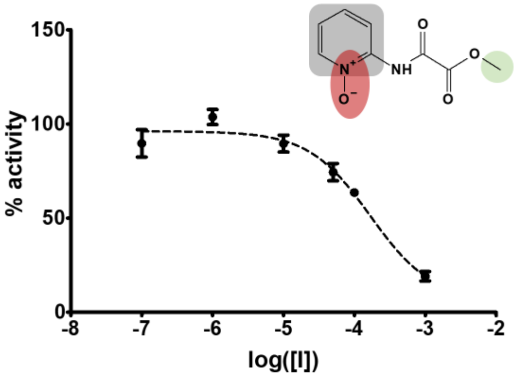

12b

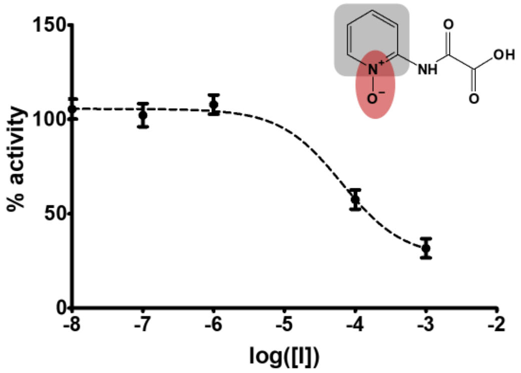

13a

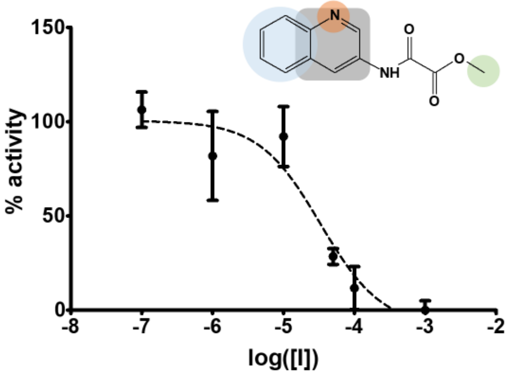

14a

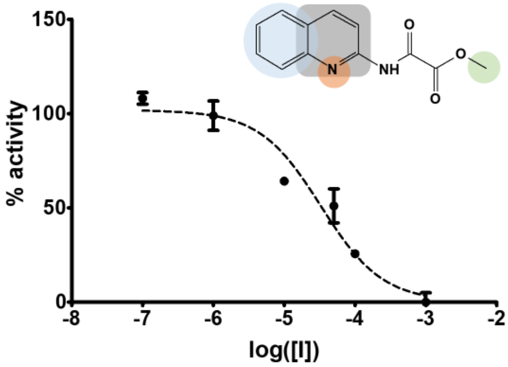

15a

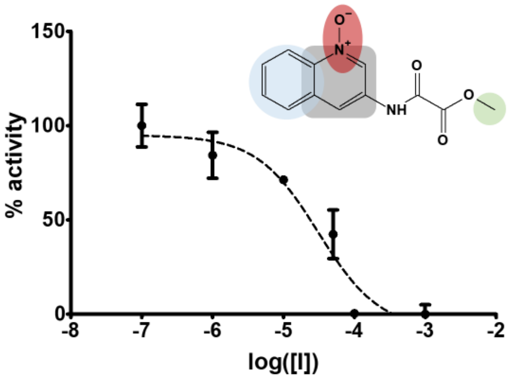

15b

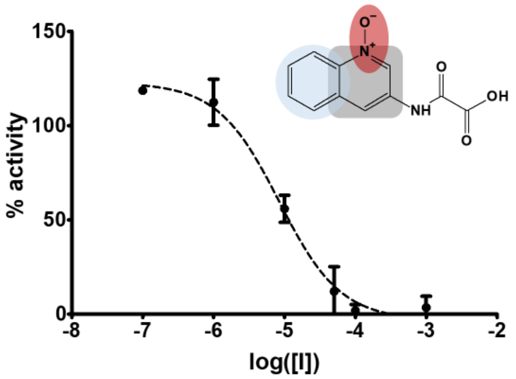

16a

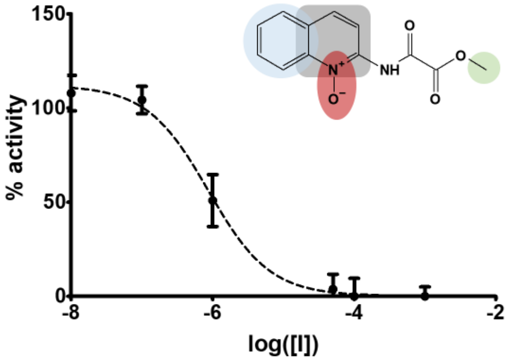

16b

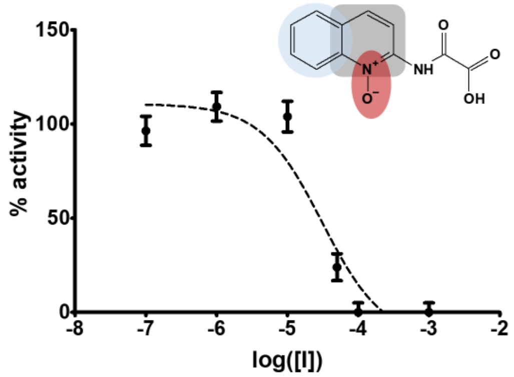

17a

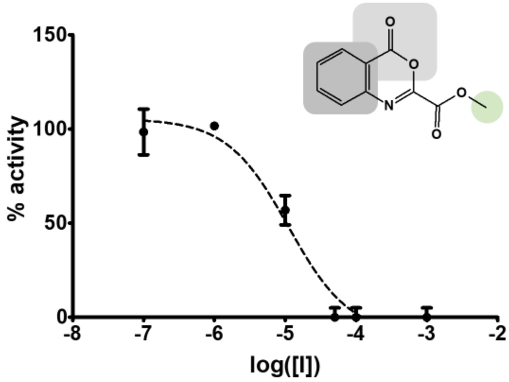

18a

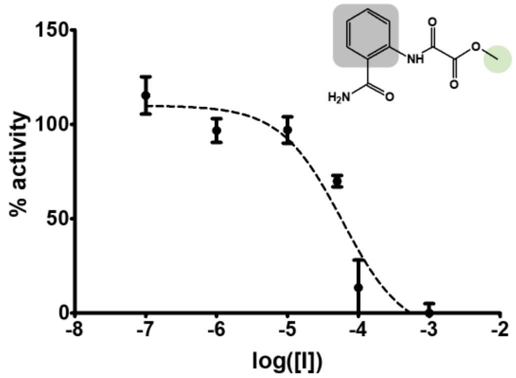

19a

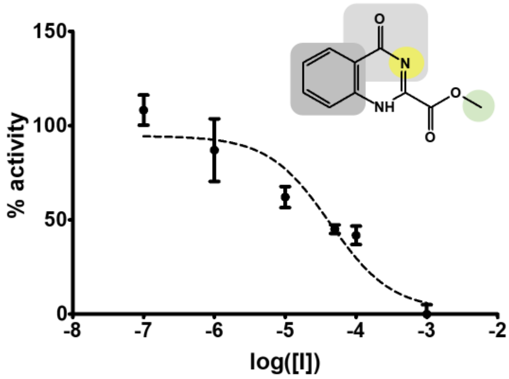

19b

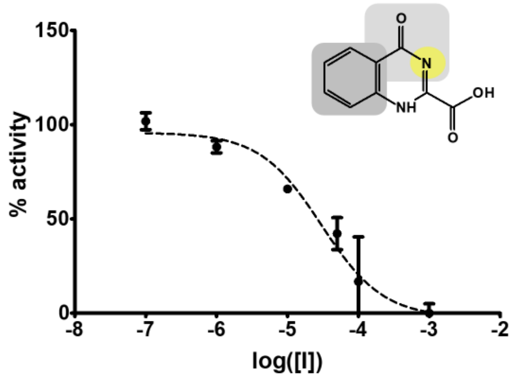

20b

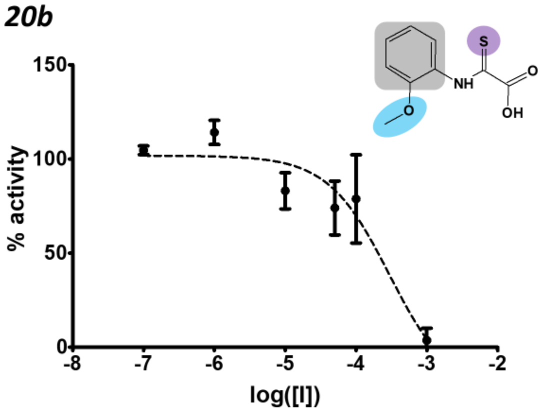

21a

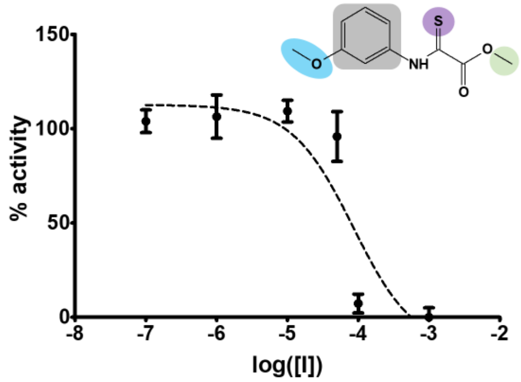

21b

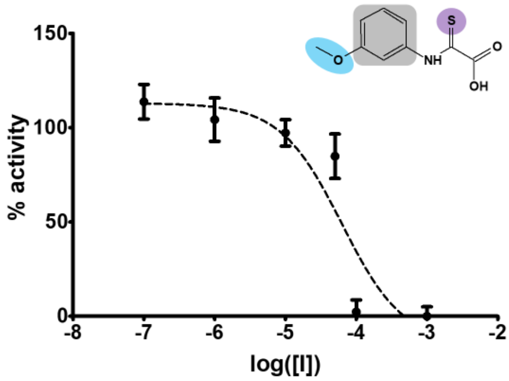

22a

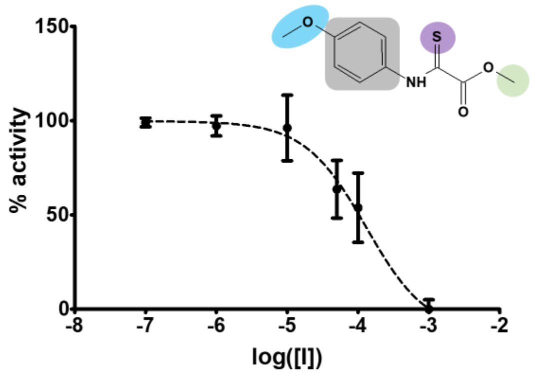

22b

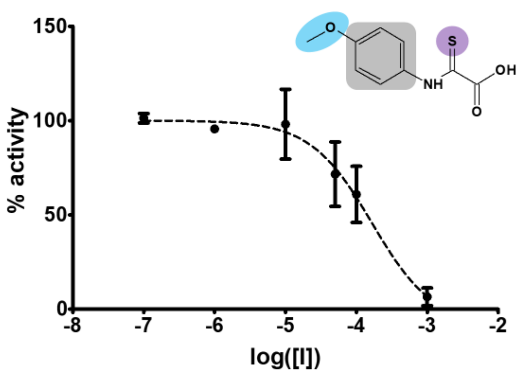

23

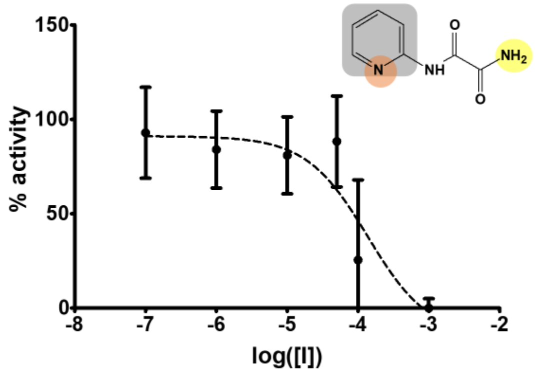

24

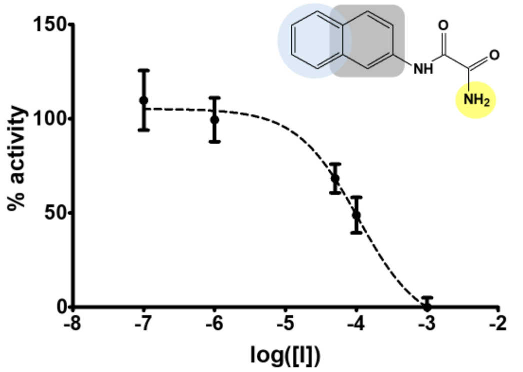

25

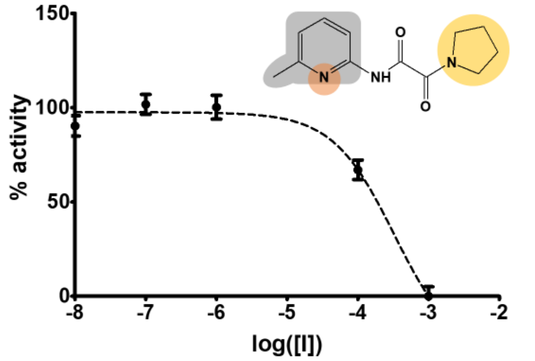

26

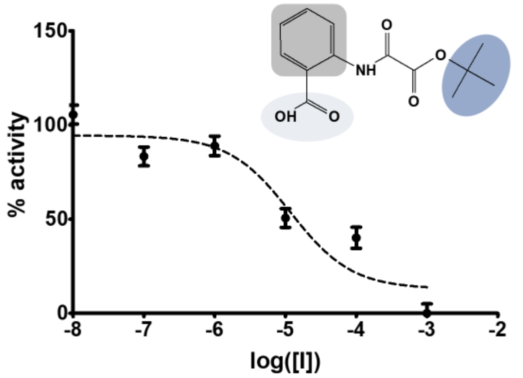

27

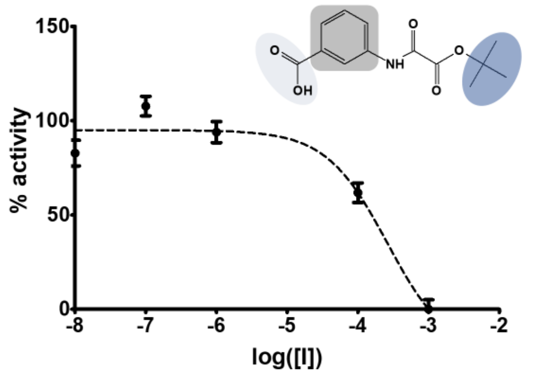

28

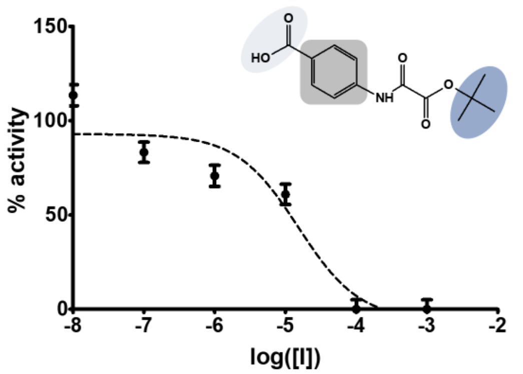

29

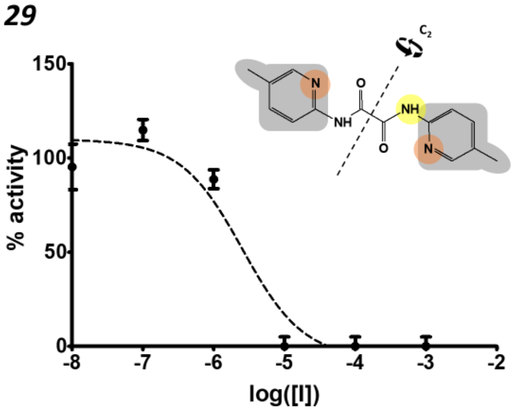

30

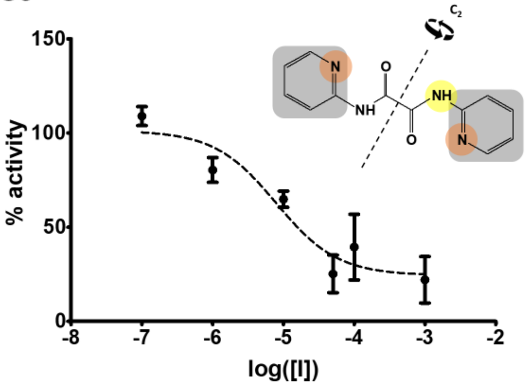

31

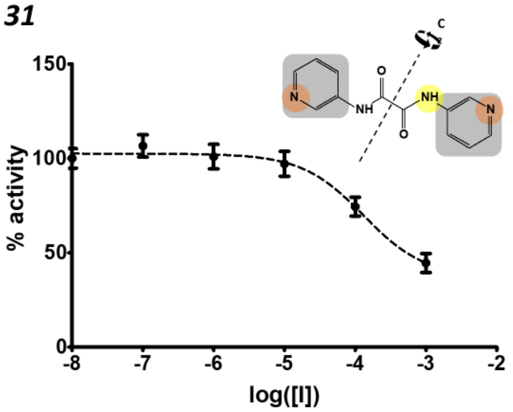

33

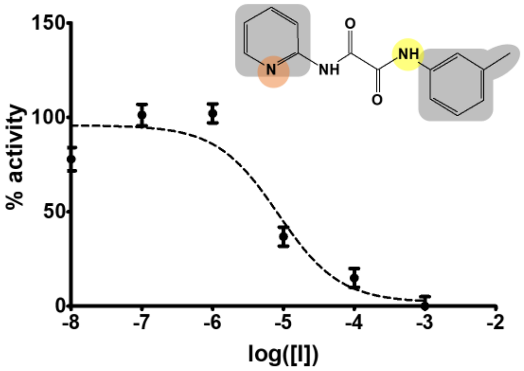

34

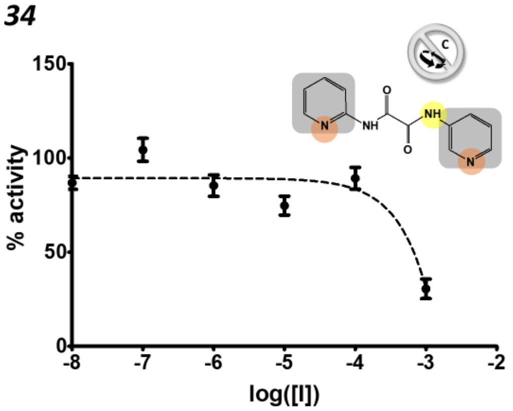

32

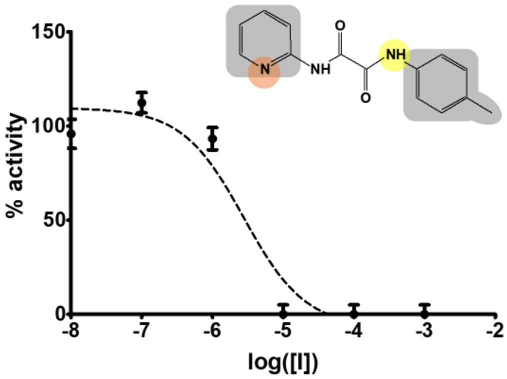

35

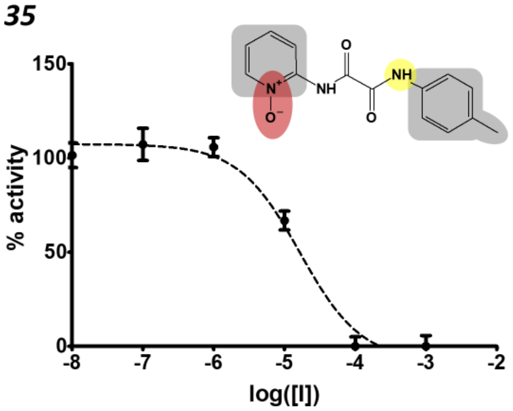

36

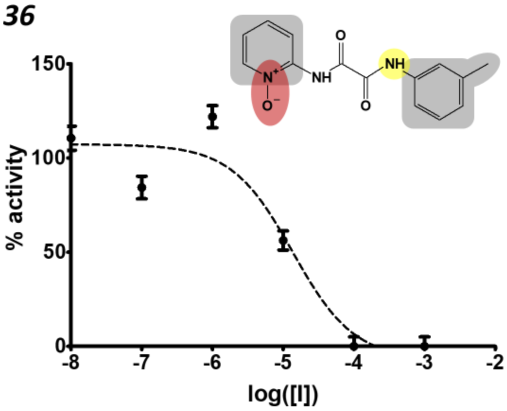

37

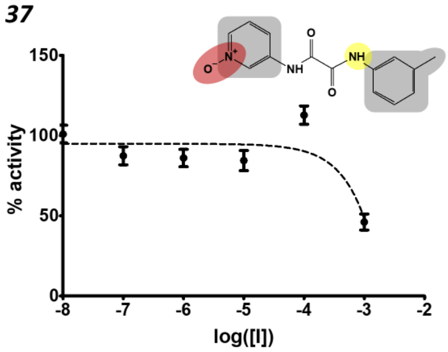

150
100
50
0
% activity
-8
-7
-6
-5
-4
-3
-2
log([I])
NH
NH
O
O

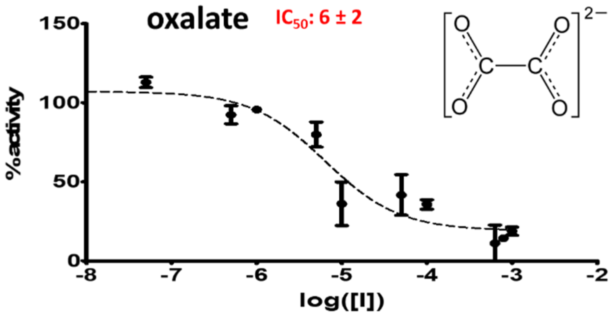

oxalate
IC50: 6 ± 2
150
100
50
0
%activity
-8
-7
-6
-5
-4
-3
-2
log([I])

- **References**

1. Llopis, J.; Mccaffery, J.M.; Miyawaki, A.; Farquhar, M.G.; Tsien, R.Y. Measurement of cytosolic, mitochondrial, and Golgi pH in single living cells with green fluorescent proteins. *Cell Biol.* **1998**, *95*, 6803–6808.

2. Flint, D.H.; Nudelman, A.; Calabrese, J.C.; Gottlieb, H.E. Enol oxalacetic acid exists in the Z form in the crystalline state and in solution. *J. Org. Chem.* **1992**, *57*, 7270–7274, doi:10.1021/jo00052a049.

3. Gelles, E. 925. Kinetics of the decarboxylation of oxaloacetic acid. *J. Chem. Soc.* **1956**, *0*, 4736, doi:10.1039/jr9560004736.

4. Kokesh, F.C. The determination by proton nuclear magnetic resonance of the enol, hydrate and keto forms of oxaloacetic acid and its anions. *J. Org. Chem.* **1976**, *41*, 3593–3599, doi:10.1021/jo00884a025.

5. Pogson, C.I.I.; Wolfe, R.G.G. Oxaloacetic acid tautomeric and hydrated forms in solution. *Biochem. Biophys. Res. Commun.* **1972**, *46*, 1048–1054, doi:10.1016/S0006-291X(72)80078-0.

6. Tate, S.S.; Grzybowski, A.K.; Datta, S.P. 265. The acid dissociations of the keto and enol isomers of oxaloacetic acid at 25°. *J. Chem. Soc.* **1964**, *0*, 1372–1380, doi:10.1039/JR9640001372.

7. Pedersen, K.J. Uncatalyzed and the metal-ion-catalyzed decarboxylation of oxaloacetic acid. *Acta Chem. Scand.* **1952**, *6*, 285.

8. Tate, S.S.; Grzybowski, A.K.; Datta, S.P. 266. The stability constants of the magnesium complexes of the keto and enol isomers of oxaloacetic acid at 25°. *J. Chem. Soc.* **1964**, *0*, 1381–1389, doi:10.1039/JR9640001381.

9. Steinberger, R.; Westheimer, F.H. THE METAL ION CATALYZED DECARBOXYLATION OF DIMETHYLOXALOACETIC ACID. *J. Am. Chem. Soc.* **1949**, *71*, 4158–4159, doi:10.1021/ja01180a517.

10. Steinberger, R.; Westheimer, F.H. Metal Ion-catalyzed Decarboxylation: A Model for an Enzyme System 1. *J. Am. Chem. Soc.* **1951**, *73*, 429–435, doi:10.1021/ja01145a139.

11. Gelles, E.; Hay, R.W. 736. The interaction of transition-metal ions with oxaloacetic acid. Part I. The role of chelate compounds in the decarboxylation. *J. Chem. Soc.* **1958**, 3673–3683, doi:10.1039/JR9580003673.
